# Supplementary material for: A time-resolved proteomic and prognostic map of COVID-19
Source: Cell Syst. 2021 Aug 18;12(8):780–794.e7. doi: 10.1016/j.cels.2021.05.005 (PMC8201874; doi:10.1016/j.cels.2021.05.005)
Supplement: Document S1. Figures S1–S18 and Tables S1 and S2 [file mmc1.pdf]

Cell Systems, Volume *12*

Supplemental information

A time-resolved proteomic

and prognostic map of COVID-19

Vadim Demichev, Pinkus Tober-Lau, Oliver Lemke, Tatiana Nazarenko, Charlotte Thibeault, Harry Whitwell, Annika Röhl, Anja Freiwald, Lukasz Szyrwiel, Daniela Ludwig, Clara Correia-Melo, Simran Kaur Aulakh, Elisa T. Helbig, Paula Stubbemann, Lena J. Lippert, Nana-Maria Grüning, Oleg Blyuss, Spyros Vernardis, Matthew White, Christoph B. Messner, Michael Joannidis, Thomas Sonnweber, Sebastian J. Klein, Alex Pizzini, Yvonne Wohlfarter, Sabina Sahanic, Richard Hilbe, Benedikt Schaefer, Sonja Wagner, Mirja Mittermaier, Felix Machleidt, Carmen Garcia, Christoph Ruwwe-Glösenkamp, Tilman Lingscheid, Laure Bosquillon de Jarcy, Miriam S. Stegemann, Moritz Pfeiffer, Linda Jürgens, Sophy Denker, Daniel Zickler, Philipp Enghard, Aleksej Zelezniak, Archie Campbell, Caroline Hayward, David J. Porteous, Riccardo E. Marioni, Alexander Uhrig, Holger Müller-Redetzky, Heinz Zoller, Judith Löffler-Ragg, Markus A. Keller, Ivan Tancevski, John F. Timms, Alexey Zaikin, Stefan Hippenstiel, Michael Ramharter, Martin Witzenrath, Norbert Suttorp, Kathryn Lilley, Michael Mülleder, Leif Erik Sander, PA-COVID-19 Study group, Markus Ralser, and Florian Kurth

Patient state	Descriptor	Score
Uninfected	No clinical or virological evidence of infection	0
Ambulatory	No limitation of activities	1
	Limitation of activities	2
Hospitalised - mild disease	No oxygen therapy	3
	Oxygen by mask or nasal prongs	4
Hospitalised - severe disease	Non-invasive ventilation or high-flow oxygen	5
	Intubation and mechanical ventilation	6
	Ventilation + additional organ support (vasopressors, RRT, ECMO)	7
Dead	Death	8

Supplementary Table 1. **WHO ordinal scale for clinical improvement in COVID-19 as used in the study** (World Health Organisation 2020).

	All patients		No invasive mechanical ventilation						Invasive mechanical ventilation				Deceased**	
	All patients		Max WHO 3 No supplemental oxygen		Max WHO 4 Supplemental low flow oxygen		Max WHO 5 Supplemental high flow oxygen		Max WHO 6 Invasive mechanical ventilation		Max WHO 7 additional organ replacement therapy			
**Number of patients**	139	100%	23	17%	33	24%	14	10%	6	4%	63	45%	17	12%
**Sex**														
Female	44	32%	13	57%	8	24%	3	21%	1	17%	19	30%	3	18%
Male	95	68%	10	43%	25	76%	11	79%	5	83%	44	70%	14	82%
**Age, years** (Median, IQR)	61	50 - 71	49	35 - 70	63	48 - 71	62	49 - 85	64	59 - 71	62	53 - 72	69	55 - 77
≥65	57	41%	6	26%	16	48%	6	43%	3	50%	26	41%	11	65%
**BMI** (kg/m^2, Median, IQR)	27.8	24.7 - 31.9	25.4	23.0 - 30.6	27.2	23.3 - 30.3	27.1	23.3 - 34.9	26.6	24.6 - 28.1	29.4	25.7 - 34.1	29.0	24.8 - 31.0
< 25 kg/m^2	40	29%	10	43%	13	39%	3	21%	2	33%	12	20%	4	24%
≥ 25 kg/m^2	97	71%	13	57%	20	61%	11	79%	4	66%	49	80%	13	76%
**Pre-existing conditions**														
CCI (Median, IQR)	3	1 - 4	1	0 - 3	4	1 - 5	3	1 - 6	2.5	2 - 5	3	1 - 4	3	3 - 6
<3	65	47%	16	70%	13	39%	7	50%	3	50%	26	41%	2	12%
≥3	74	53%	7	30%	20	61%	7	50%	3	50%	37	59%	15	88%
Hypertension	78	56%	6	26%	21	64%	7	50%	3	50%	41	65%	15	88%
Congestive heart failure	7	5%	0	0%	3	9%	0	0%	1	17%	3	5%	1	6%
h/o myocardial infarction	13	9%	1	4%	2	6%	1	7%	1	17%	8	13%	1	6%
Chronic lung disease	22	16%	2	9%	6	18%	1	7%	2	33%	11	17%	3	18%
Asthma	7	5%	1	4%	2	6%	0	0%	0	0%	4	6%	1	6%
COPD	15	11%	1	4%	5	15%	0	0%	1	17%	8	13%	2	12%
on home oxygen therapy	6	4%	0	0%	1	3%	0	0%	1	17%	4	6%	2	12%
Diabetes	31	22%	1	4%	9	27%	4	29%	1	17%	16	25%	3	18%
Type II diabetes (% of diabetes)	30	97%	1	100%	8	89%	4	100%	1	100%	16	100%	3	100%
Oral hypoglycaemic drugs	16	12%	0	0%	5	15%	2	14%	0	0%	9	14%	2	12%
Insulin therapy	11	8%	0	0%	6	18%	2	14%	0	0%	3	5%	0	0%
Chronic kidney disease	19	14%	2	9%	8	24%	3	21%	0	0%	6	10%	2	12%
Requiring RRT	2	2%	0	0%	1	3%	0	0%	0	0%	1	2%	1	6%
Chronic liver disease	8	6%	2	9%	3	9%	1	7%	0	0%	2	3%	1	6%
Lipid metabolism disorder	21	15%	4	17%	9	27%	1	7%	1	17%	6	10%	3	18%
h/o stroke	7	5%	0	0%	1	3%	1	7%	1	17%	4	6%	1	6%
Ischemic (% of stroke)	6	86%	0	0%	1	100%	1	100%	1	100%	3	75%	0	0%
Active malignancy	3	2%	0	0%	0	0%	0	0%	1	17%	2	3%	1	6%
h/o solid organ transplant	3	2%	0	0%	2	6%	0	0%	0	0%	1	2%	1	6%
h/o immunosuppressive therapy within past 3 months	13	9%	2	9%	4	12%	2	14%	0	0%	5	8%	3	18%
h/o smoking	33	24%	6	26%	14	42%	2	14%	2	33%	9	14%	2	12%
Current smoker	5	4%	1	4%	2	6%	0	0%	1	17%	1	2%	0	0%
**Outpatient medication**														
Total amount (Median, IQR)	2	1 - 3	1	0 - 2	3	2 - 5	1	0 - 2	5	2 - 8	2	1 - 4	2	1 - 3
ARB	24	17%	2	9%	6	18%	2	14%	0	0%	14	22%	2	12%
ACE-I	22	22%	3	13%	8	24%	1	7%	0	0%	10	16%	2	12%
Lipid lowering agents	25	25%	2	9%	11	33%	0	0%	1	17%	11	17%	3	18%

**Duration of hospital course* (days)**	20	9 - 48	7	4 - 10	14	9 - 17	20	13 - 28	38	24 - 71	46	32 - 75	28	16 - 46
Proning**	51	38%	-	-	-	-	-	-	2	33%	49	78%	13	76%
Mechanical ventilation**	69	50%	-	-	-	-	-	-	6	100%	63	100%	17	100%
RRT	46	33%	0	0%	1	3%	0	0%	1	17%	44	70%	15	88%
ECMO**	22	16%	-	-	-		-	-	-	-	22	35%	8	47%
ARDS	76	55%	0	0%	3	9%	7	50%	4	67%	62	98%	17	100%
Sepsis	41	29%	0	0%	2	6%	3	21%	2	33%	34	54%	12	71%
Thromboembolic event	32	23%	0	0%	1	3%	2	14%	1	17%	28	44%	8	47%
Cardiopulmonary resuscitation**	7	6%	0	0%	0	0%	0	0%	0	0%	7	12%	2	12%
**Outcome**														
Deceased (incl. DNI/DNR)	20***	14%	0	0%	1	3%	2	14%	0	0%	17	27%	-	-
DNI/DNR	3	2%	0	0%	1	3%	2	14%	0	0%	0	0%	-	-
Secondary DNI/DNR	4	3%	0	0%	0	0%	0	0%	0	0%	4	6%	4	24%
Requiring new oxygen therapy after discharge*	12	10%	0	0%	0	0%	1	10%	0	0%	11	24%	-	-
Requiring new RRT after discharge*	5	4%	0	0%	0	0%	0	0%	0	0%	5	11%	-	-

Data are shown in n (%) unless otherwise indicated. IQR – interquartile range, CCI – Charlson's comorbidity index; CKD – chronic kidney disease; ACE-I – angiotensin-converting enzyme inhibitor, ARB - Angiotensin II receptor blockers, RRT – renal replacement therapy, ECMO – extracorporeal membrane oxygenation, DNI/DNR – do not intubate/do not resuscitate, i.e. patient's wish not to receive mechanical ventilation, organ replacement therapy, or cardiopulmonary resuscitation - secondary DNR - secondary limitation of therapy in situation of probable unfavourable outcome and according to the presumed patient's wish

* deceased patients not included

** patients with DNI/DNR not included

*** one patient died of non-COVID-19 related cause

Supplementary Table 2. **Baseline, treatment and outcome characteristics of patient cohort with COVID-19 at Charité - University hospital Berlin**. Patients are stratified according to the maximum grade on WHO ordinal scale.

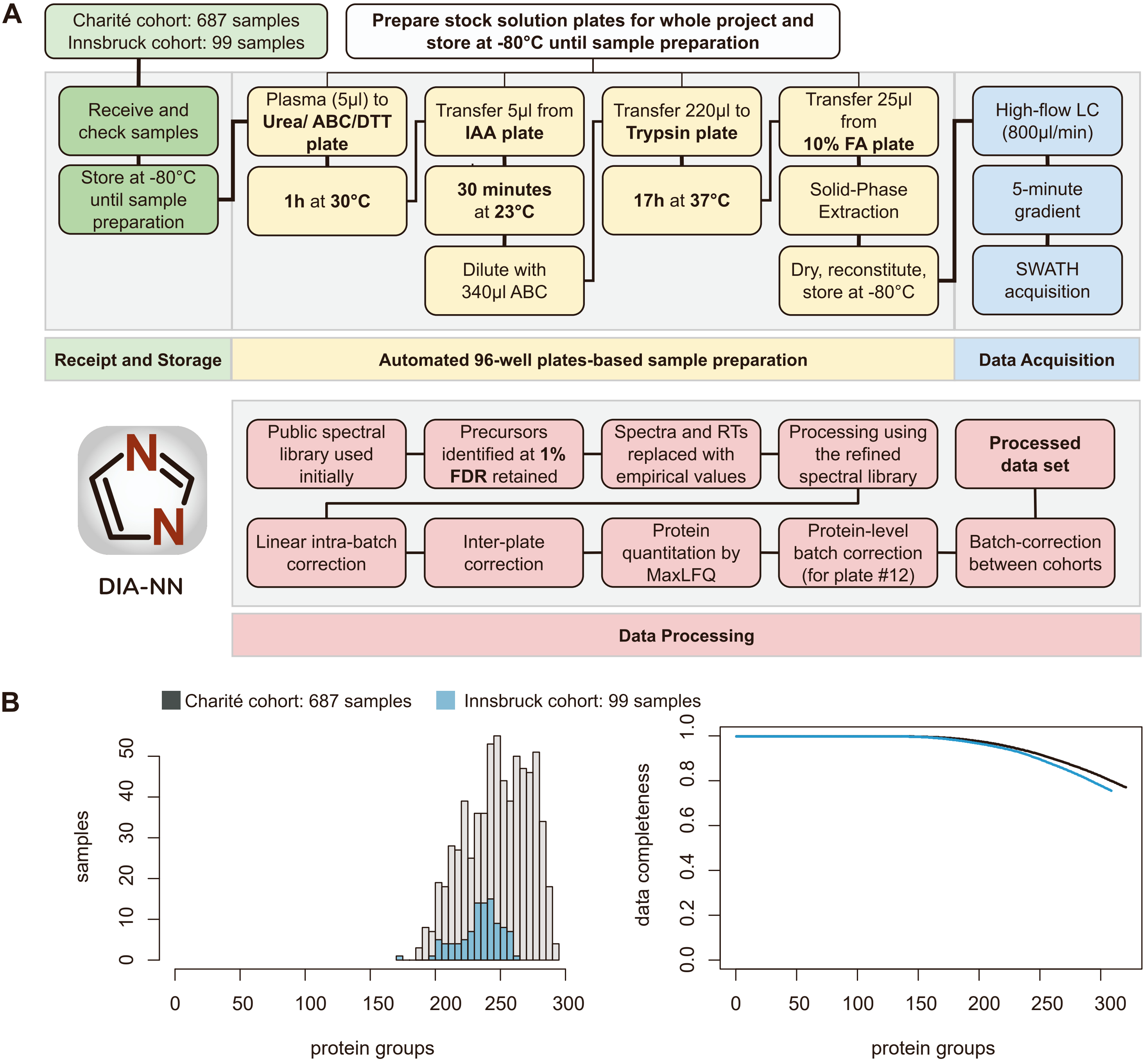

Figure S1. **Plasma proteomics workflow. A** The schematic of the 96-well plates-based sample preparation workflow (Messner et al., 2020), followed by mass spectrometry and raw data processing. The workflow results in normalised and batch-corrected protein quantities. Imputation of missing values is not performed per se, but might be required for certain downstream analyses. All statistical tests in the present work were applied without imputation, while imputation was performed for machine learning (Methods). **B** Distribution of the numbers of quantified protein groups across different samples in two cohorts (left) and the data completeness plotted against the number of protein groups (right).

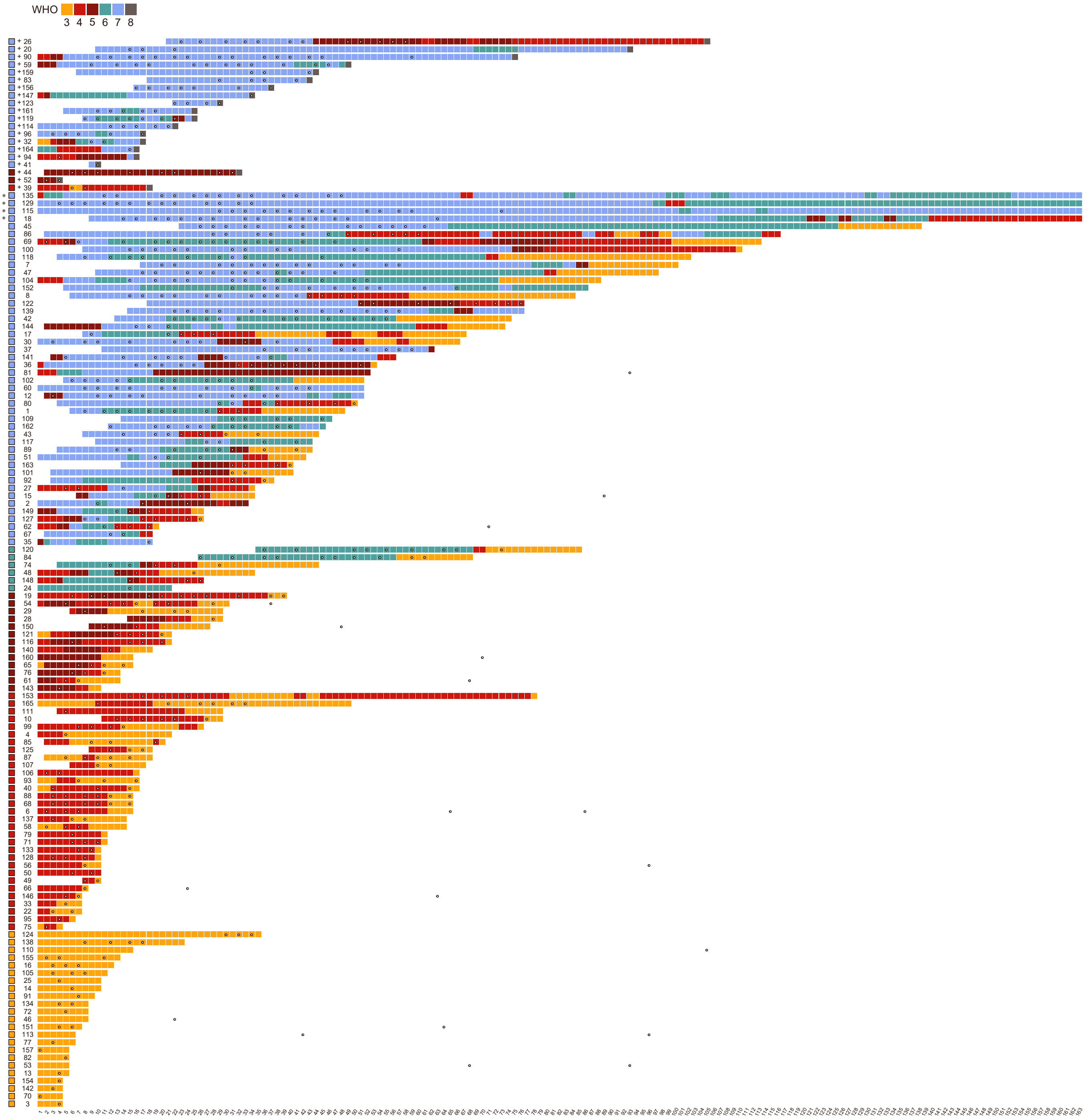

Figure S2. **Disease trajectories from hospital admission onward**. Patient IDs are given on the y-axis, number of days since admission on the x-axis, WHO severity grade is color-coded, starting with the day the patient was admitted to Charite or transferred from another hospital. Proteomic samples (including follow-up visits after discharge) are indicated with white points.

+ deceased

* still in hospital on 30 August 2020

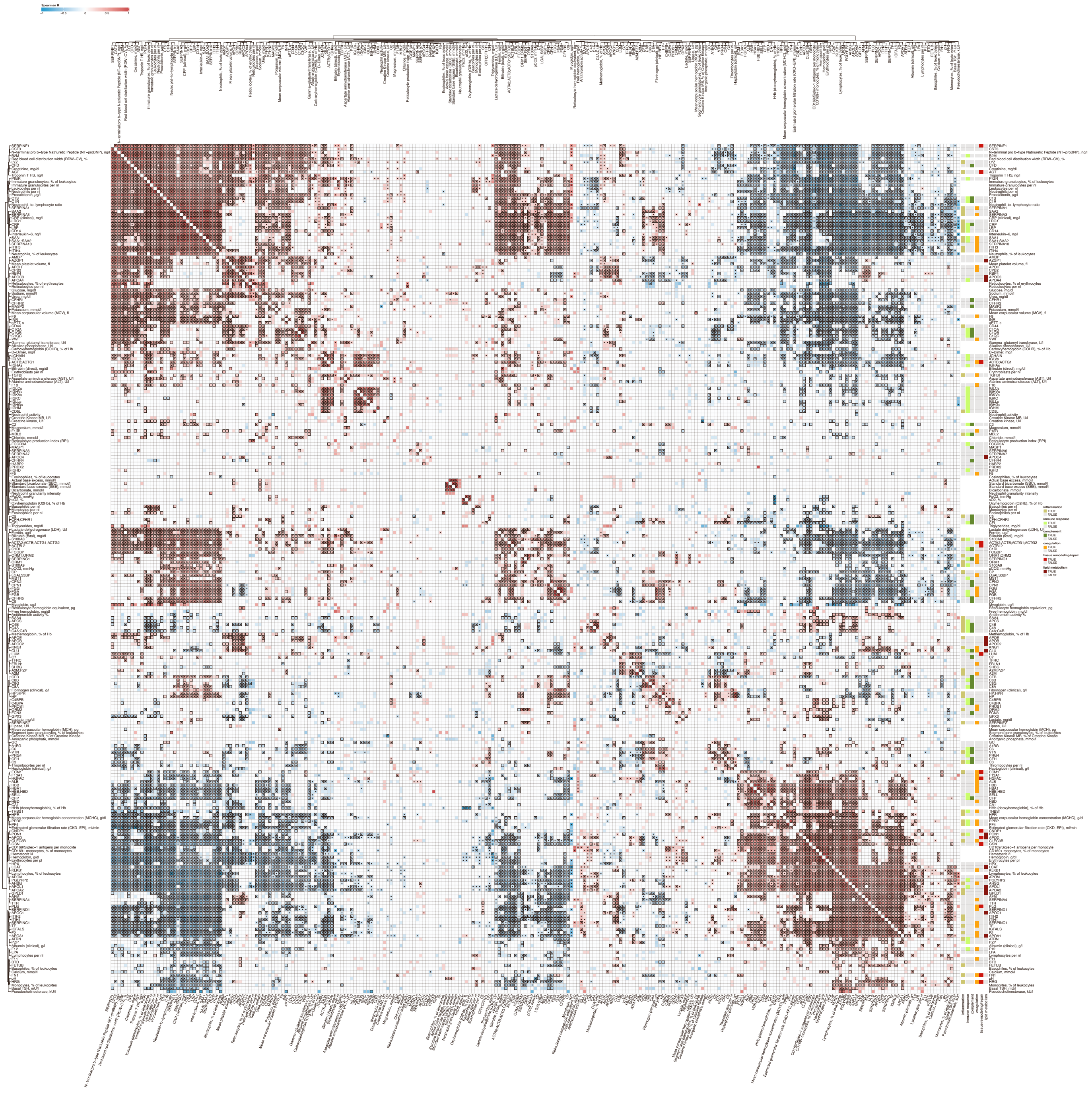

Figure S3. **Map of all correlations between omics measurements**

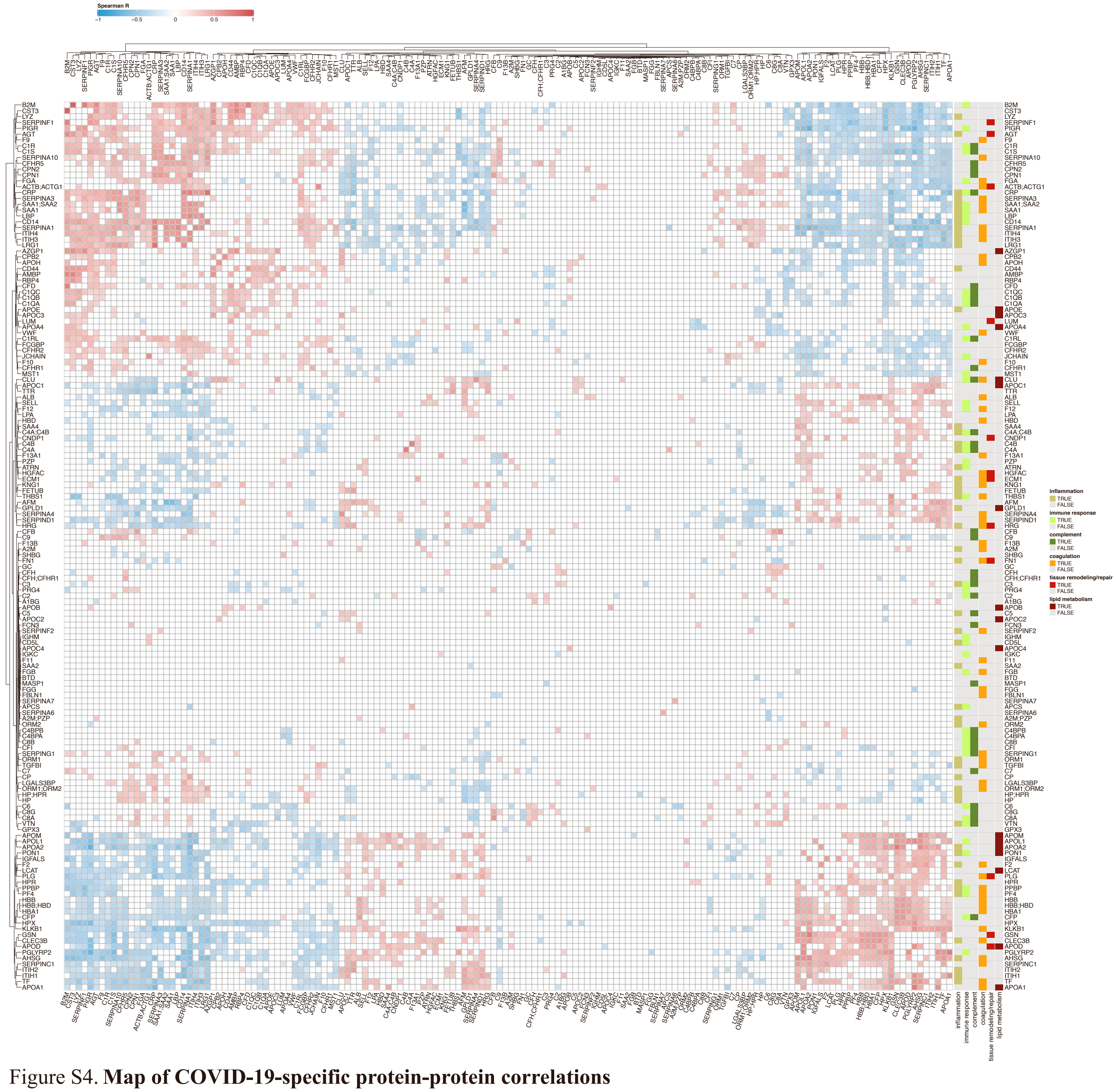

Figure S4. **Map of COVID-19-specific protein-protein correlations**

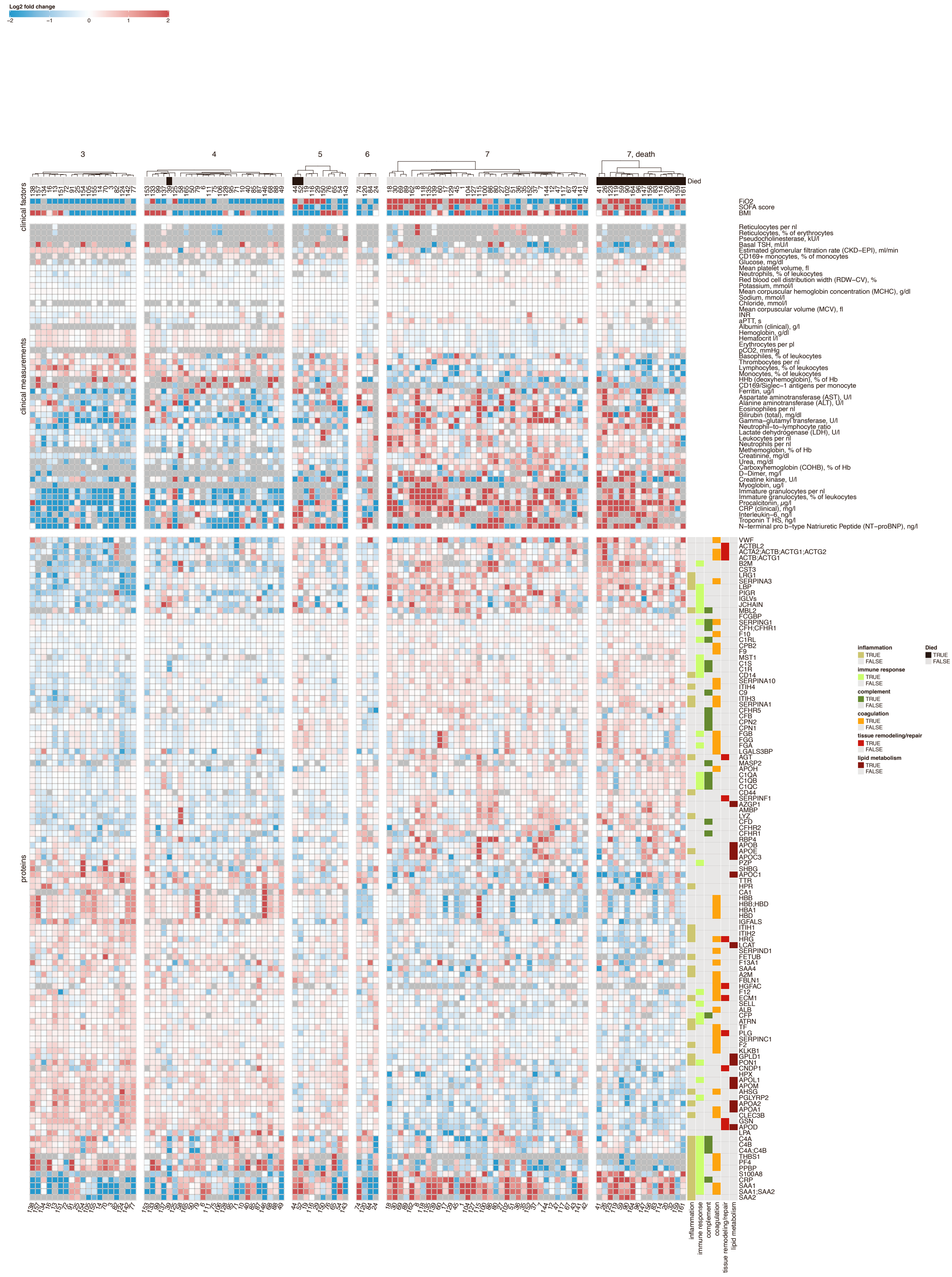

Figure S5. **Map of omics response depending on COVID-19 severity**

Figure S6 (following 5 pages). **Omics features significantly regulated depending on COVID-19 severity**

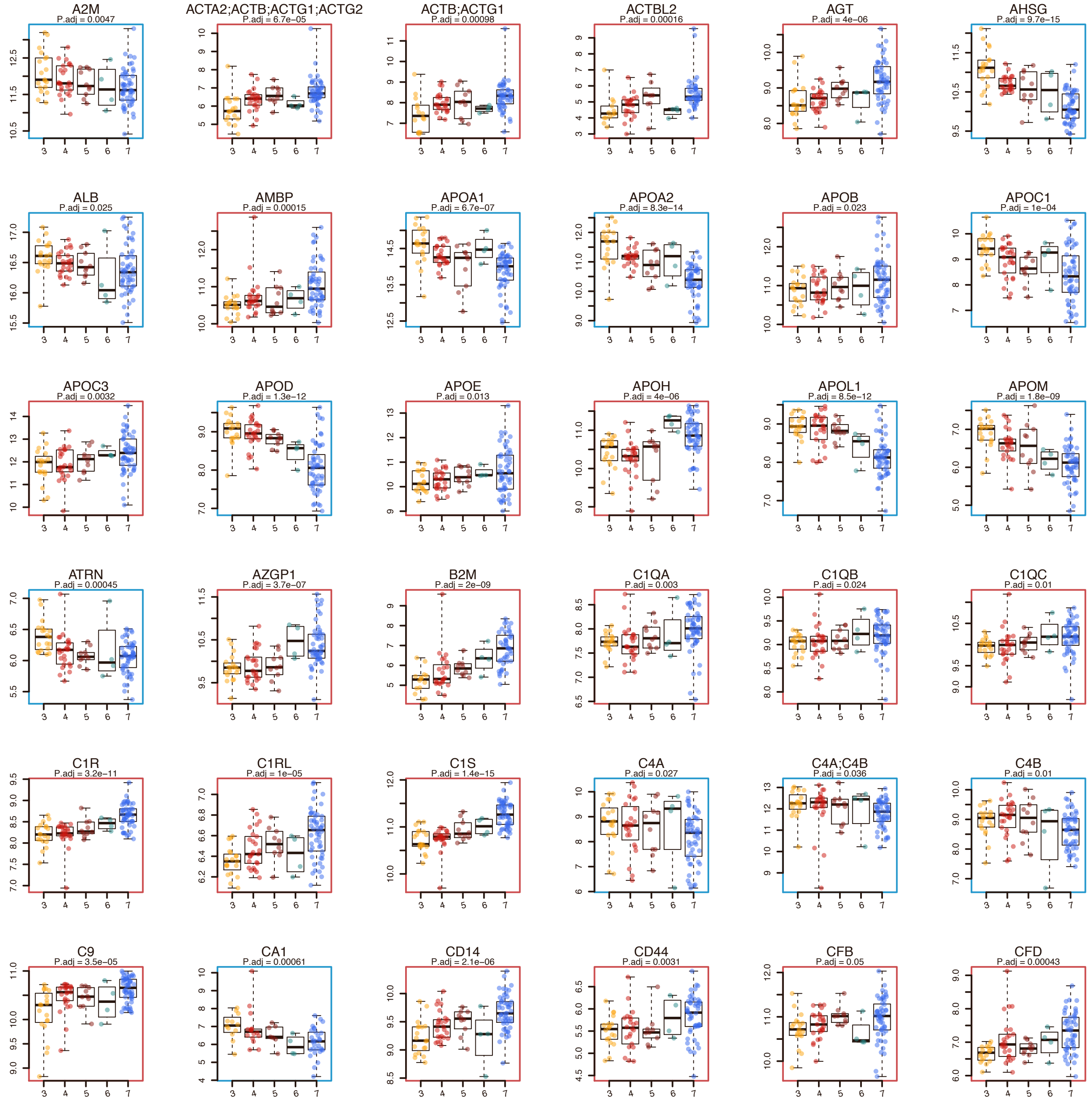

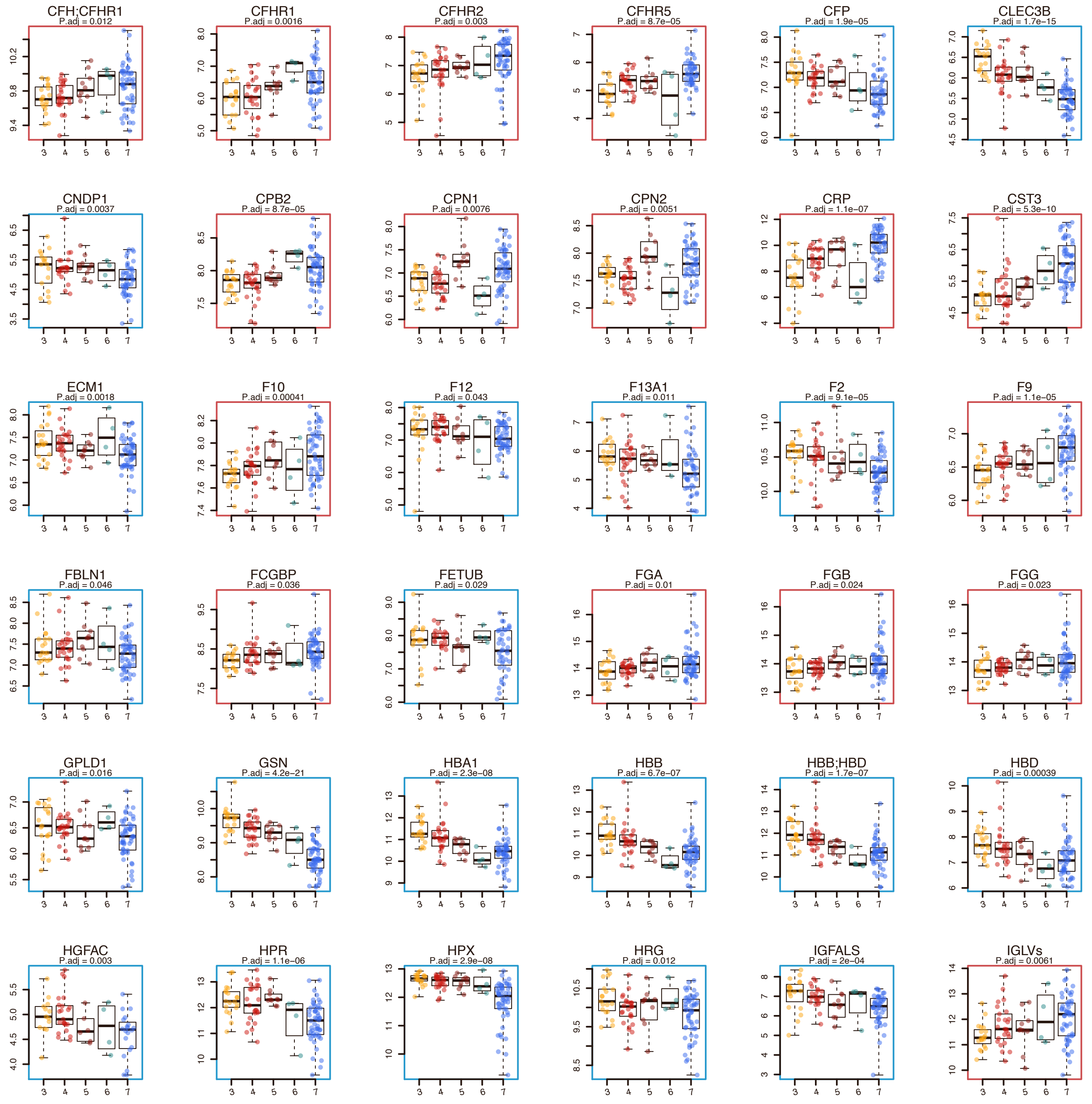
CFH;CFHR1
P.adj = 0.012
CFHR1
P.adj = 0.0016
CFHR2
P.adj = 0.003
CFHR5
P.adj = 8.7e−05
CFP
P.adj = 1.9e−05
CLEC3B
P.adj = 1.7e−15
CNDP1
P.adj = 0.0037
CPB2
P.adj = 8.7e−05
CPN1
P.adj = 0.0076
CPN2
P.adj = 0.0051
CRP
P.adj = 1.1e−07
CST3
P.adj = 5.3e−10
ECM1
P.adj = 0.0018
F10
P.adj = 0.00041
F12
P.adj = 0.043
F13A1
P.adj = 0.011
F2
P.adj = 9.1e−05
F9
P.adj = 1.1e−05
FBLN1
P.adj = 0.046
FCGBP
P.adj = 0.036
FETUB
P.adj = 0.029
FGA
P.adj = 0.01
FGB
P.adj = 0.024
FGG
P.adj = 0.023
GPLD1
P.adj = 0.016
GSN
P.adj = 4.2e−21
HBA1
P.adj = 2.3e−08
HBB
P.adj = 6.7e−07
HBB;HBD
P.adj = 1.7e−07
HBD
P.adj = 0.00039
HGFAC
P.adj = 0.003
HPR
P.adj = 1.1e−06
HPX
P.adj = 2.9e−08
HRG
P.adj = 0.012
IGFALS
P.adj = 2e−04
IGLVs
P.adj = 0.0061

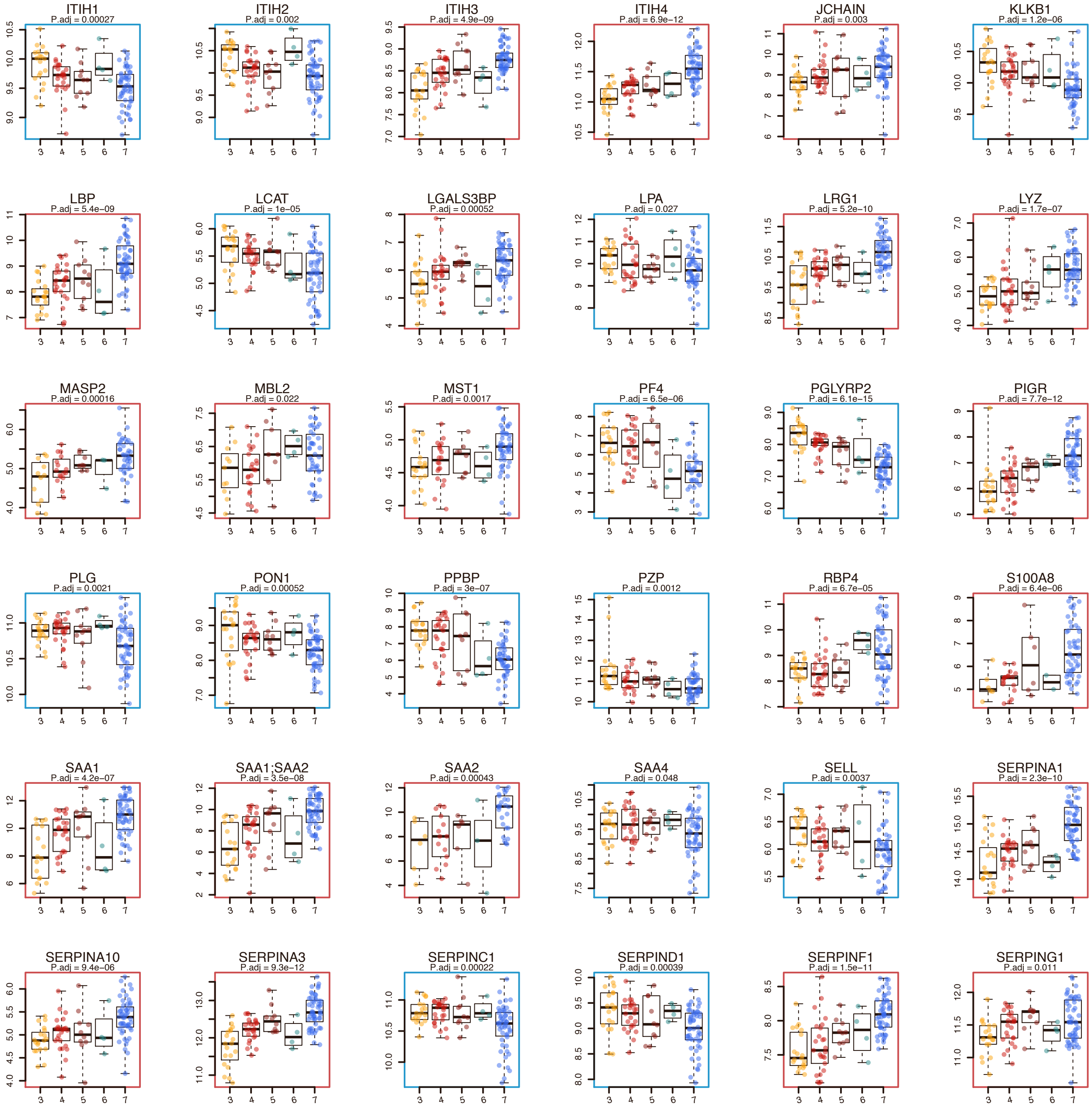

ITIH1
P.adj = 0.00027
ITIH2
P.adj = 0.002
ITIH3
P.adj = 4.9e−09
ITIH4
P.adj = 6.9e−12
JCHAIN
P.adj = 0.003
KLKB1
P.adj = 1.2e−06
LBP
P.adj = 5.4e−09
LCAT
P.adj = 1e−05
LGALS3BP
P.adj = 0.00052
LPA
P.adj = 0.027
LRG1
P.adj = 5.2e−10
LYZ
P.adj = 1.7e−07
MASP2
P.adj = 0.00016
MBL2
P.adj = 0.022
MST1
P.adj = 0.0017
PF4
P.adj = 6.5e−06
PGLYRP2
P.adj = 6.1e−15
PIGR
P.adj = 7.7e−12
PLG
P.adj = 0.0021
PON1
P.adj = 0.00052
PPBP
P.adj = 3e−07
PZP
P.adj = 0.0012
RBP4
P.adj = 6.7e−05
S100A8
P.adj = 6.4e−06
SAA1
P.adj = 4.2e−07
SAA1;SAA2
P.adj = 3.5e−08
SAA2
P.adj = 0.00043
SAA4
P.adj = 0.048
SELL
P.adj = 0.0037
SERPINA1
P.adj = 2.3e−10
SERPINA10
P.adj = 9.4e−06
SERPINA3
P.adj = 9.3e−12
SERPINC1
P.adj = 0.00022
SERPIND1
P.adj = 0.00039
SERPINF1
P.adj = 1.5e−11
SERPING1
P.adj = 0.011

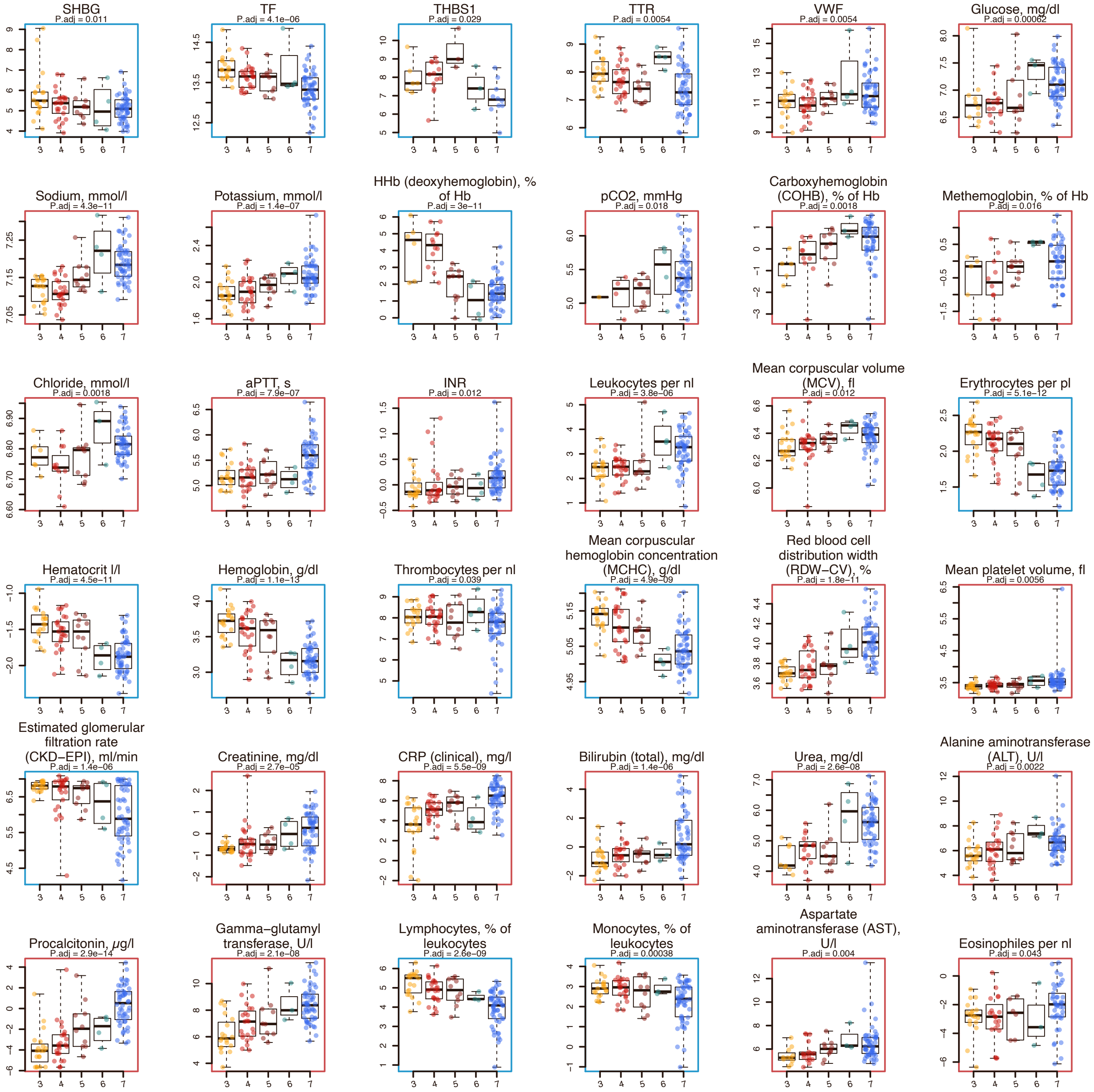

SHBG
P.adj = 0.011
TF
P.adj = 4.1e−06
THBS1
P.adj = 0.029
TTR
P.adj = 0.0054
VWF
P.adj = 0.0054
Glucose, mg/dl
P.adj = 0.00062
Sodium, mmol/l
P.adj = 4.3e−11
Potassium, mmol/l
P.adj = 1.4e−07
HHb (deoxyhemoglobin), % of Hb
P.adj = 3e−11
pCO2, mmHg
P.adj = 0.018
Carboxyhemoglobin (COHB), % of Hb
P.adj = 0.0018
Methemoglobin, % of Hb
P.adj = 0.016
Chloride, mmol/l
P.adj = 0.0018
aPTT, s
P.adj = 7.9e−07
INR
P.adj = 0.012
Leukocytes per nl
P.adj = 3.8e−06
Mean corpuscular volume (MCV), fl
P.adj = 0.012
Erythrocytes per pl
P.adj = 5.1e−12
Hematocrit l/l
P.adj = 4.5e−11
Hemoglobin, g/dl
P.adj = 1.1e−13
Thrombocytes per nl
P.adj = 0.039
Mean corpuscular hemoglobin concentration (MCHC), g/dl
P.adj = 4.9e−09
Red blood cell distribution width (RDW−CV), %
P.adj = 1.8e−11
Mean platelet volume, fl
P.adj = 0.0056
Estimated glomerular filtration rate (CKD−EPI), ml/min
P.adj = 1.4e−06
Creatinine, mg/dl
P.adj = 2.7e−05
CRP (clinical), mg/l
P.adj = 5.5e−09
Bilirubin (total), mg/dl
P.adj = 1.4e−06
Urea, mg/dl
P.adj = 2.6e−08
Alanine aminotransferase (ALT), U/l
P.adj = 0.0022
Procalcitonin, µg/l
P.adj = 2.9e−14
Gamma−glutamyl transferase, U/l
P.adj = 2.1e−08
Lymphocytes, % of leukocytes
P.adj = 2.6e−09
Monocytes, % of leukocytes
P.adj = 0.00038
Aspartate aminotransferase (AST), U/l
P.adj = 0.004
Eosinophiles per nl
P.adj = 0.043

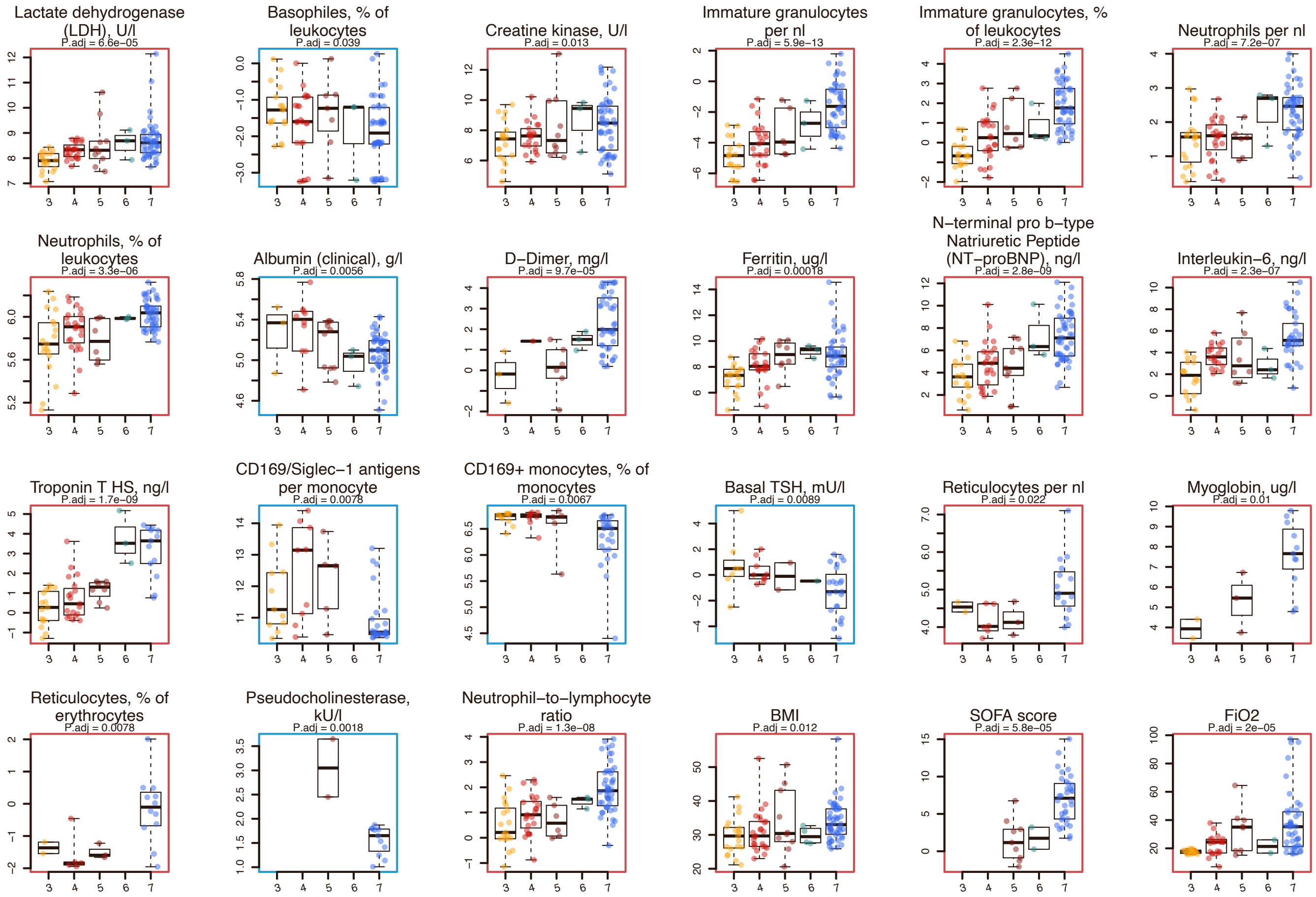

Lactate dehydrogenase (LDH), U/l
P.adj = 6.6e−05
Basophiles, % of leukocytes
P.adj = 0.039
Creatine kinase, U/l
P.adj = 0.013
Immature granulocytes per nl
P.adj = 5.9e−13
Immature granulocytes, % of leukocytes
P.adj = 2.3e−12
Neutrophils per nl
P.adj = 7.2e−07
Neutrophils, % of leukocytes
P.adj = 3.3e−06
Albumin (clinical), g/l
P.adj = 0.0056
D−Dimer, mg/l
P.adj = 9.7e−05
Ferritin, ug/l
P.adj = 0.00018
N−terminal pro b−type Natriuretic Peptide (NT−proBNP), ng/l
P.adj = 2.8e−09
Interleukin−6, ng/l
P.adj = 2.3e−07
Troponin T HS, ng/l
P.adj = 1.7e−09
CD169/Siglec−1 antigens per monocyte
P.adj = 0.0078
CD169+ monocytes, % of monocytes
P.adj = 0.0067
Basal TSH, mU/l
P.adj = 0.0089
Reticulocytes per nl
P.adj = 0.022
Myoglobin, ug/l
P.adj = 0.01
Reticulocytes, % of erythrocytes
P.adj = 0.0078
Pseudocholinesterase, kU/l
P.adj = 0.0018
Neutrophil−to−lymphocyte ratio
P.adj = 1.3e−08
BMI
P.adj = 0.012
SOFA score
P.adj = 5.8e−05
FiO2
P.adj = 2e−05

Figure S7. **Omics features significantly regulated upon RRT**

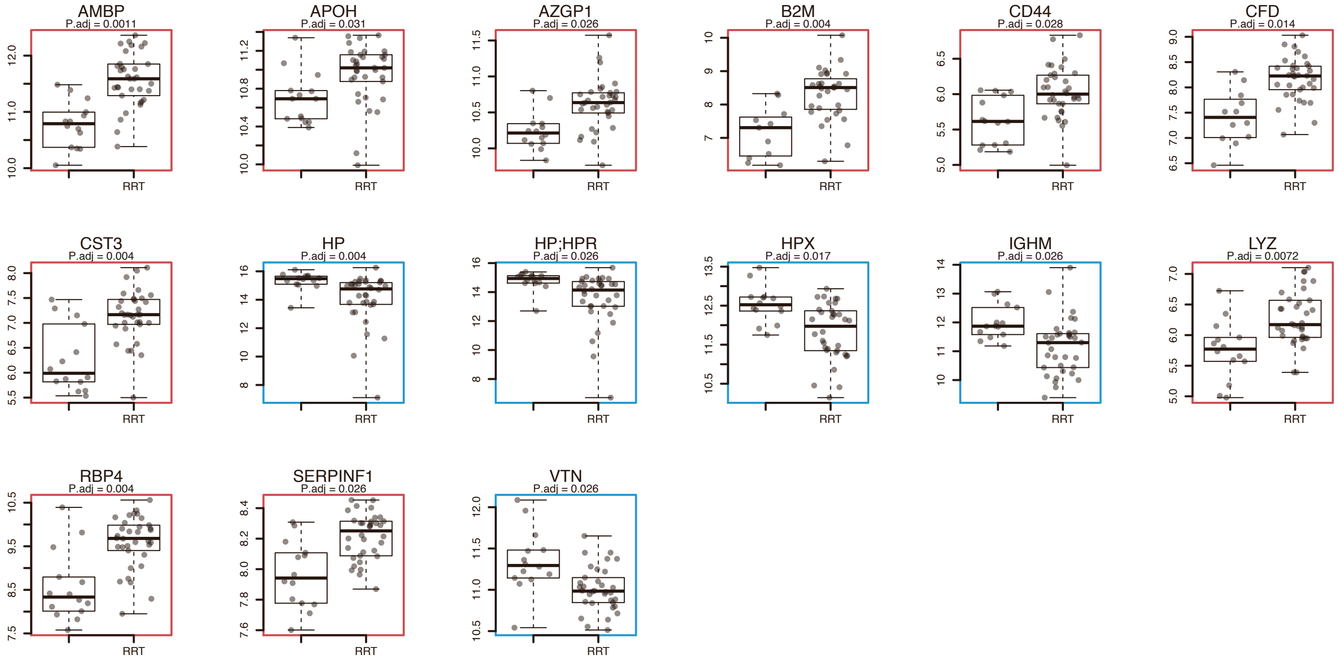

Figure S8. **Omics features significantly regulated upon ECMO**

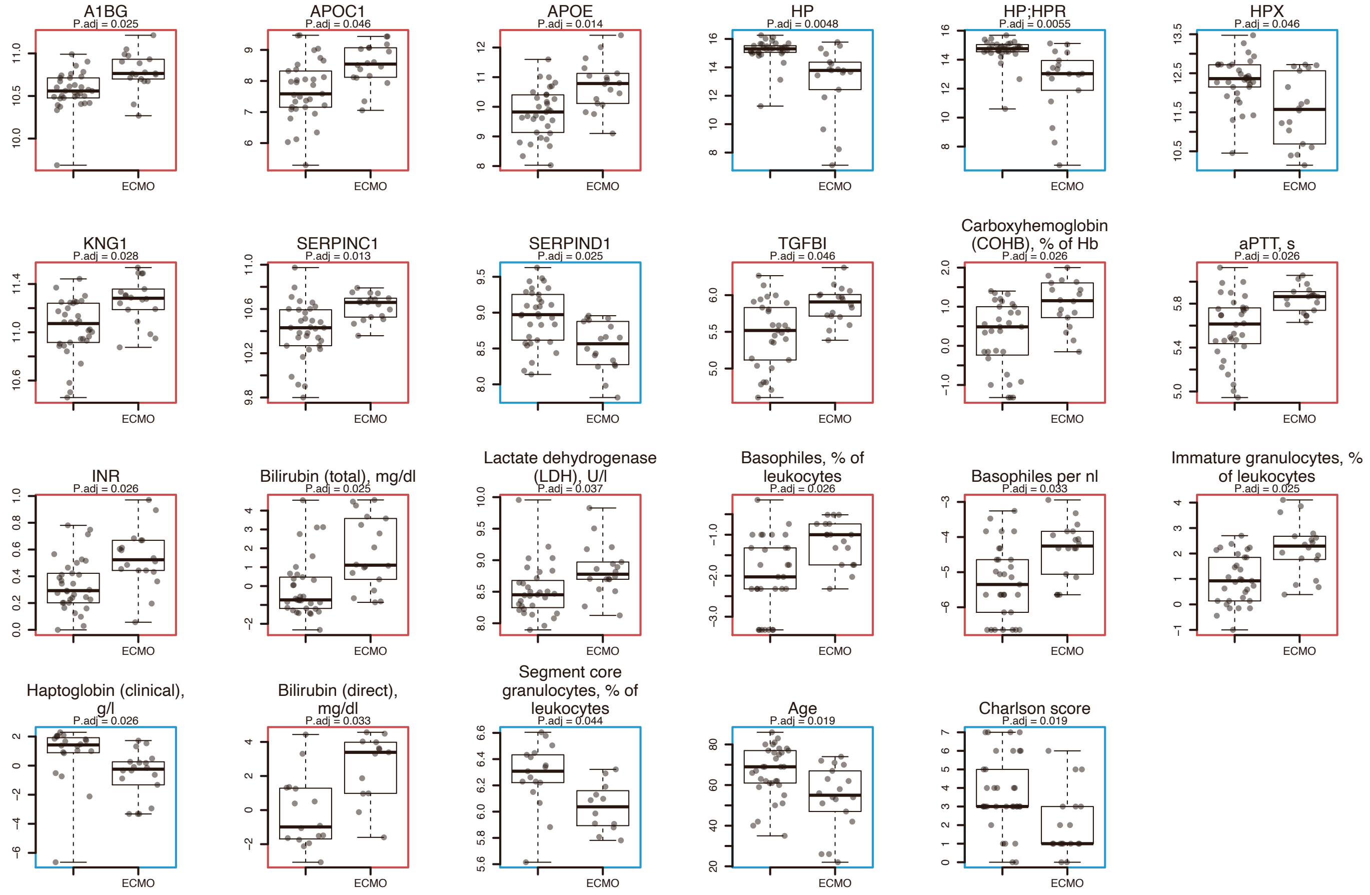

Figure S9 (following 3 pages). **Omics features changing with age - test without accounting for the WHO grade as a covariate**

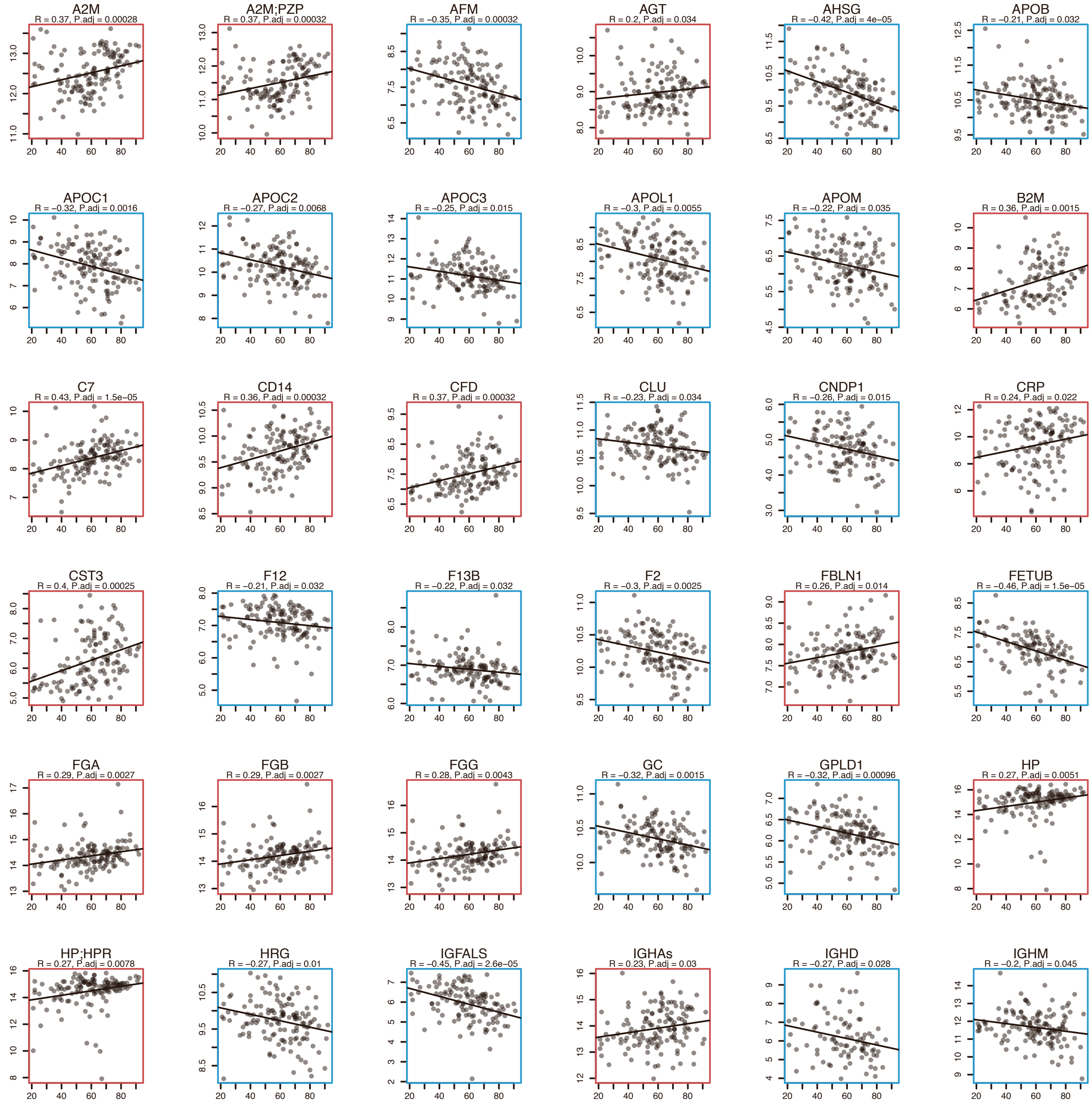

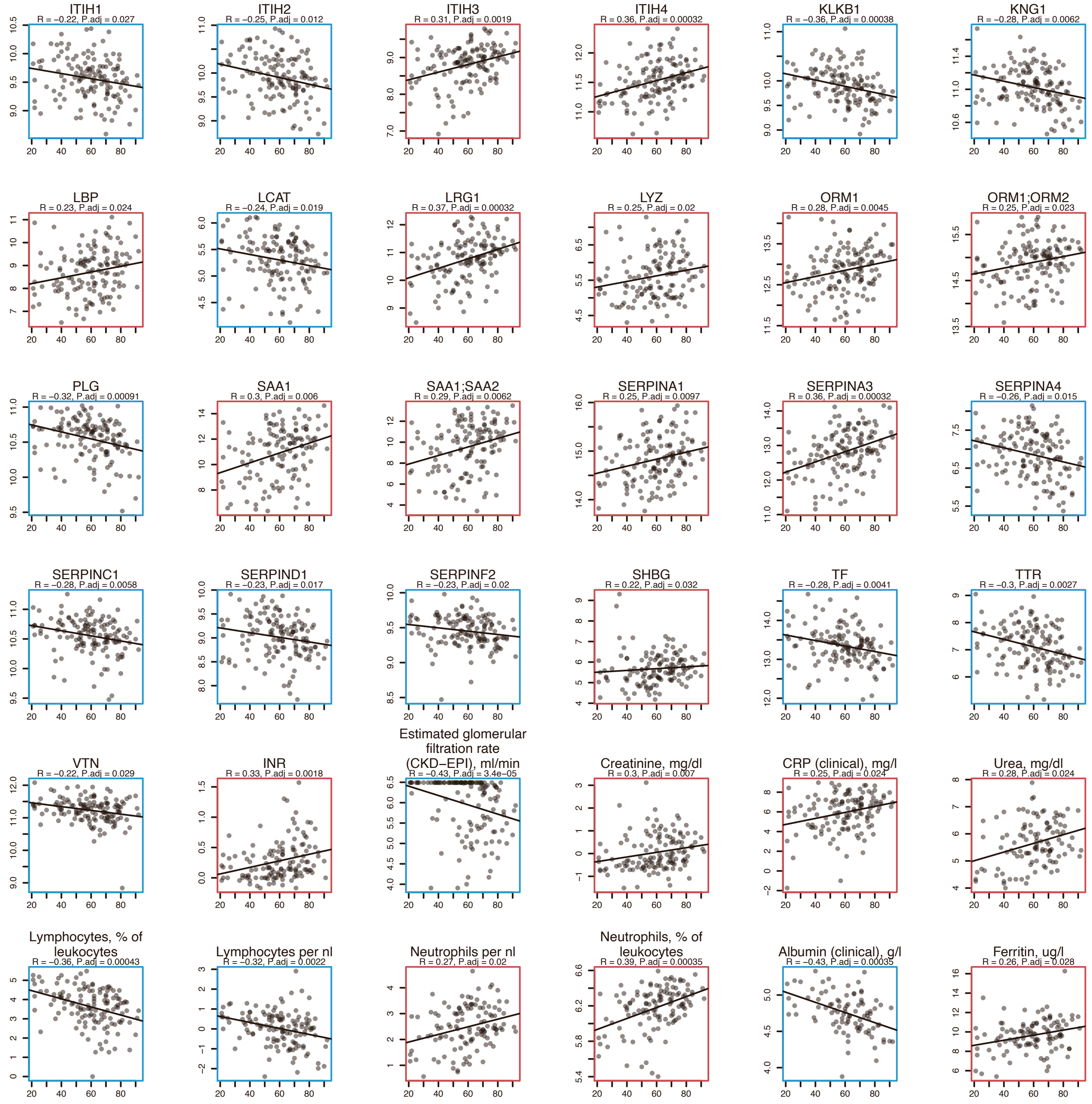
ITIH1
R = −0.22, P.adj = 0.027
ITIH2
R = −0.25, P.adj = 0.012
ITIH3
R = 0.31, P.adj = 0.0019
ITIH4
R = 0.36, P.adj = 0.00032
KLKB1
R = −0.36, P.adj = 0.00038
KNG1
R = −0.28, P.adj = 0.0062
LBP
R = 0.23, P.adj = 0.024
LCAT
R = −0.24, P.adj = 0.019
LRG1
R = 0.37, P.adj = 0.00032
LYZ
R = 0.25, P.adj = 0.02
ORM1
R = 0.28, P.adj = 0.0045
ORM1;ORM2
R = 0.25, P.adj = 0.023
PLG
R = −0.32, P.adj = 0.00091
SAA1
R = 0.3, P.adj = 0.006
SAA1;SAA2
R = 0.29, P.adj = 0.0062
SERPINA1
R = 0.25, P.adj = 0.0097
SERPINA3
R = 0.36, P.adj = 0.00032
SERPINA4
R = −0.26, P.adj = 0.015
SERPINC1
R = −0.28, P.adj = 0.0058
SERPIND1
R = −0.23, P.adj = 0.017
SERPINF2
R = −0.23, P.adj = 0.02
SHBG
R = 0.22, P.adj = 0.032
TF
R = −0.28, P.adj = 0.0041
TTR
R = −0.3, P.adj = 0.0027
VTN
R = −0.22, P.adj = 0.029
INR
R = 0.33, P.adj = 0.0018
Estimated glomerular filtration rate (CKD−EPI), ml/min
R = −0.43, P.adj = 3.4e−05
Creatinine, mg/dl
R = 0.3, P.adj = 0.007
CRP (clinical), mg/l
R = 0.25, P.adj = 0.024
Urea, mg/dl
R = 0.28, P.adj = 0.024
Lymphocytes, % of leukocytes
R = −0.36, P.adj = 0.00043
Lymphocytes per nl
R = −0.32, P.adj = 0.0022
Neutrophils per nl
R = 0.27, P.adj = 0.02
Neutrophils, % of leukocytes
R = 0.39, P.adj = 0.00035
Albumin (clinical), g/l
R = −0.43, P.adj = 0.00035
Ferritin, ug/l
R = 0.26, P.adj = 0.028

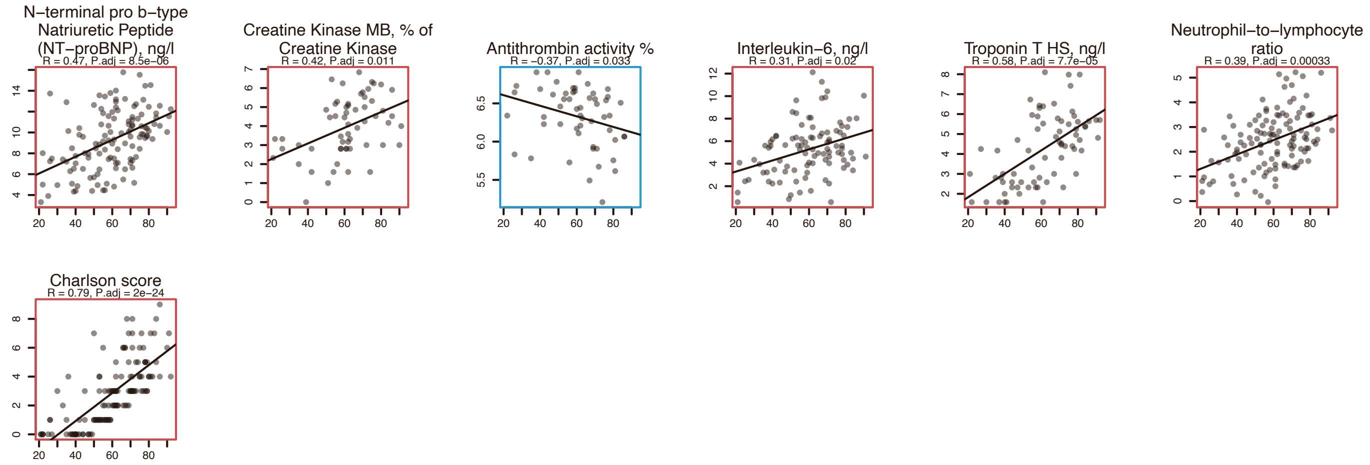

N–terminal pro b–type Natriuretic Peptide (NT–proBNP), ng/l
R = 0.47, P.adj = 8.5e–06
Creatine Kinase MB, % of Creatine Kinase
R = 0.42, P.adj = 0.011
Antithrombin activity %
R = −0.37, P.adj = 0.033
Interleukin–6, ng/l
R = 0.31, P.adj = 0.02
Troponin T HS, ng/l
R = 0.58, P.adj = 7.7e–05
Neutrophil–to–lymphocyte ratio
R = 0.39, P.adj = 0.00033
Charlson score
R = 0.79, P.adj = 2e–24

Figure S10 (following 9 pages). **Plasma protein levels vs age in general population based on Generation Scotland cohort**

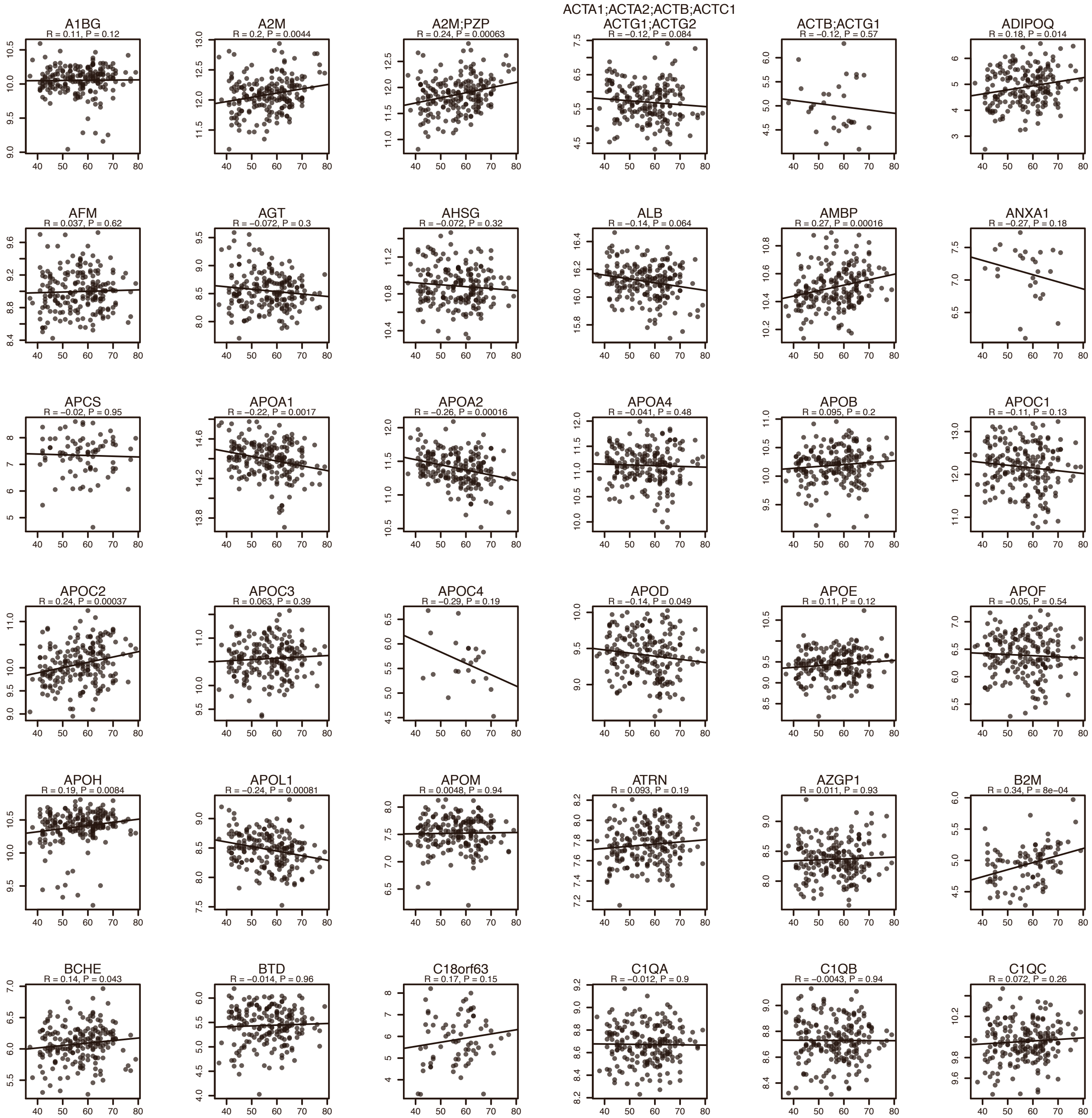

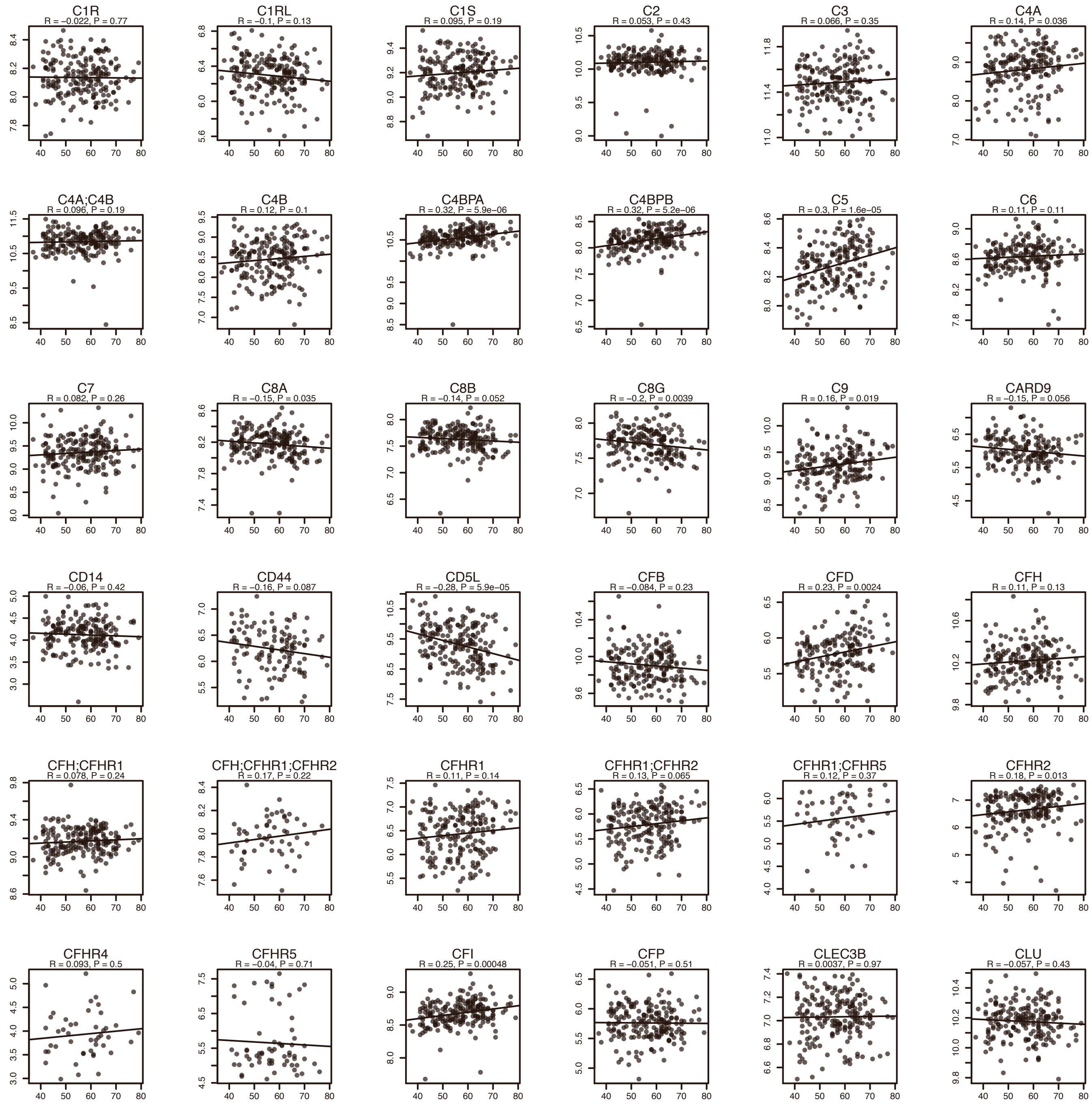
C1R
R = −0.022, P = 0.77
C1RL
R = −0.1, P = 0.13
C1S
R = 0.095, P = 0.19
C2
R = 0.053, P = 0.43
C3
R = 0.066, P = 0.35
C4A
R = 0.14, P = 0.036
C4A;C4B
R = 0.096, P = 0.19
C4B
R = 0.12, P = 0.1
C4BPA
R = 0.32, P = 5.9e−06
C4BPB
R = 0.32, P = 5.2e−06
C5
R = 0.3, P = 1.6e−05
C6
R = 0.11, P = 0.11
C7
R = 0.082, P = 0.26
C8A
R = −0.15, P = 0.035
C8B
R = −0.14, P = 0.052
C8G
R = −0.2, P = 0.0039
C9
R = 0.16, P = 0.019
CARD9
R = −0.15, P = 0.056
CD14
R = −0.06, P = 0.42
CD44
R = −0.16, P = 0.087
CD5L
R = −0.28, P = 5.9e−05
CFB
R = −0.084, P = 0.23
CFD
R = 0.23, P = 0.0024
CFH
R = 0.11, P = 0.13
CFH;CFHR1
R = 0.078, P = 0.24
CFH;CFHR1;CFHR2
R = 0.17, P = 0.22
CFHR1
R = 0.11, P = 0.14
CFHR1;CFHR2
R = 0.13, P = 0.065
CFHR1;CFHR5
R = 0.12, P = 0.37
CFHR2
R = 0.18, P = 0.013
CFHR4
R = 0.093, P = 0.5
CFHR5
R = −0.04, P = 0.71
CFI
R = 0.25, P = 0.00048
CFP
R = −0.051, P = 0.51
CLEC3B
R = 0.0037, P = 0.97
CLU
R = −0.057, P = 0.43

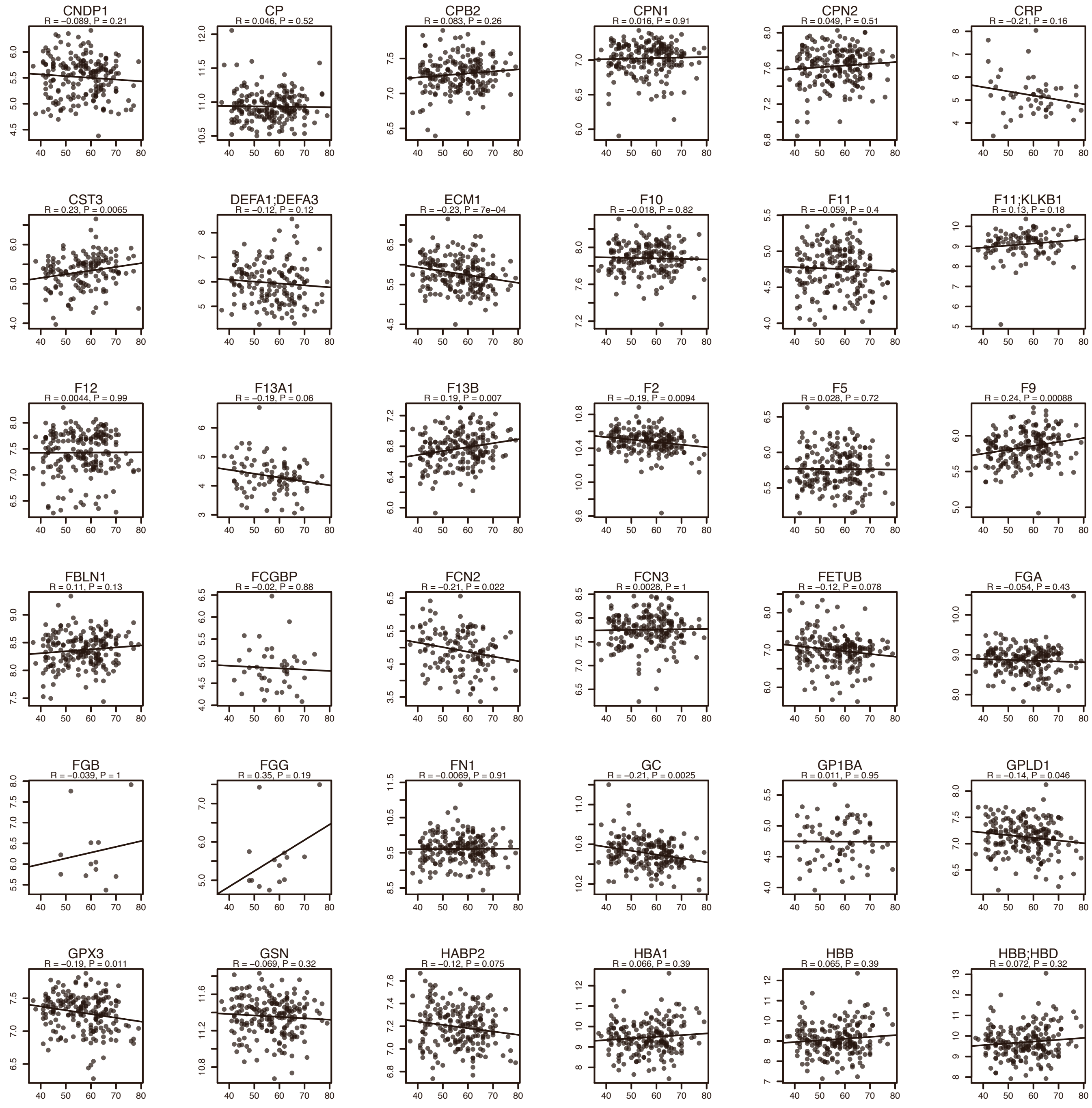
CNDP1
R = −0.089, P = 0.21
CP
R = 0.046, P = 0.52
CPB2
R = 0.083, P = 0.26
CPN1
R = 0.016, P = 0.91
CPN2
R = 0.049, P = 0.51
CRP
R = −0.21, P = 0.16
CST3
R = 0.23, P = 0.0065
DEFA1;DEFA3
R = −0.12, P = 0.12
ECM1
R = −0.23, P = 7e−04
F10
R = −0.018, P = 0.82
F11
R = −0.059, P = 0.4
F11;KLKB1
R = 0.13, P = 0.18
F12
R = 0.0044, P = 0.99
F13A1
R = −0.19, P = 0.06
F13B
R = 0.19, P = 0.007
F2
R = −0.19, P = 0.0094
F5
R = 0.028, P = 0.72
F9
R = 0.24, P = 0.00088
FBLN1
R = 0.11, P = 0.13
FCGBP
R = −0.02, P = 0.88
FCN2
R = −0.21, P = 0.022
FCN3
R = 0.0028, P = 1
FETUB
R = −0.12, P = 0.078
FGA
R = −0.054, P = 0.43
FGB
R = −0.039, P = 1
FGG
R = 0.35, P = 0.19
FN1
R = −0.0069, P = 0.91
GC
R = −0.21, P = 0.0025
GP1BA
R = 0.011, P = 0.95
GPLD1
R = −0.14, P = 0.046
GPX3
R = −0.19, P = 0.011
GSN
R = −0.069, P = 0.32
HABP2
R = −0.12, P = 0.075
HBA1
R = 0.066, P = 0.39
HBB
R = 0.065, P = 0.39
HBB;HBD
R = 0.072, P = 0.32

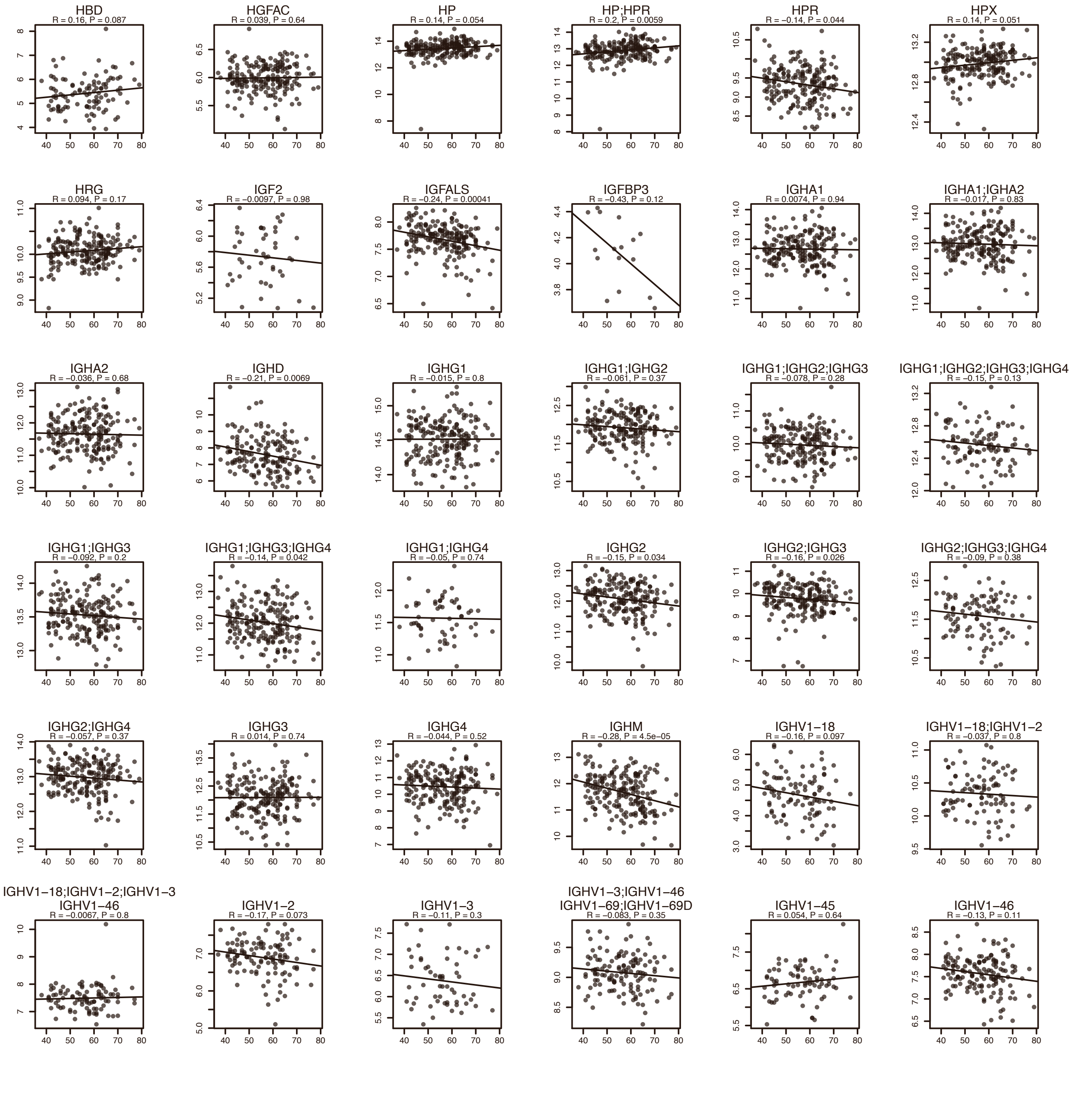
HBD
R = 0.16, P = 0.087
HGFAC
R = 0.039, P = 0.64
HP
R = 0.14, P = 0.054
HP;HPR
R = 0.2, P = 0.0059
HPR
R = −0.14, P = 0.044
HPX
R = 0.14, P = 0.051
HRG
R = 0.094, P = 0.17
IGF2
R = −0.0097, P = 0.98
IGFALS
R = −0.24, P = 0.00041
IGFBP3
R = −0.43, P = 0.12
IGHA1
R = 0.0074, P = 0.94
IGHA1;IGHA2
R = −0.017, P = 0.83
IGHA2
R = −0.036, P = 0.68
IGHD
R = −0.21, P = 0.0069
IGHG1
R = −0.015, P = 0.8
IGHG1;IGHG2
R = −0.061, P = 0.37
IGHG1;IGHG2;IGHG3
R = −0.078, P = 0.28
IGHG1;IGHG2;IGHG3;IGHG4
R = −0.15, P = 0.13
IGHG1;IGHG3
R = −0.092, P = 0.2
IGHG1;IGHG3;IGHG4
R = −0.14, P = 0.042
IGHG1;IGHG4
R = −0.05, P = 0.74
IGHG2
R = −0.15, P = 0.034
IGHG2;IGHG3
R = −0.16, P = 0.026
IGHG2;IGHG3;IGHG4
R = −0.09, P = 0.38
IGHG2;IGHG4
R = −0.057, P = 0.37
IGHG3
R = 0.014, P = 0.74
IGHG4
R = −0.044, P = 0.52
IGHM
R = −0.28, P = 4.5e−05
IGHV1−18
R = −0.16, P = 0.097
IGHV1−18;IGHV1−2
R = −0.037, P = 0.8
IGHV1−18;IGHV1−2;IGHV1−3
IGHV1−46
R = −0.0067, P = 0.8
IGHV1−2
R = −0.17, P = 0.073
IGHV1−3
R = −0.11, P = 0.3
IGHV1−3;IGHV1−46
IGHV1−69;IGHV1−69D
R = −0.083, P = 0.35
IGHV1−45
R = 0.054, P = 0.64
IGHV1−46
R = −0.13, P = 0.11

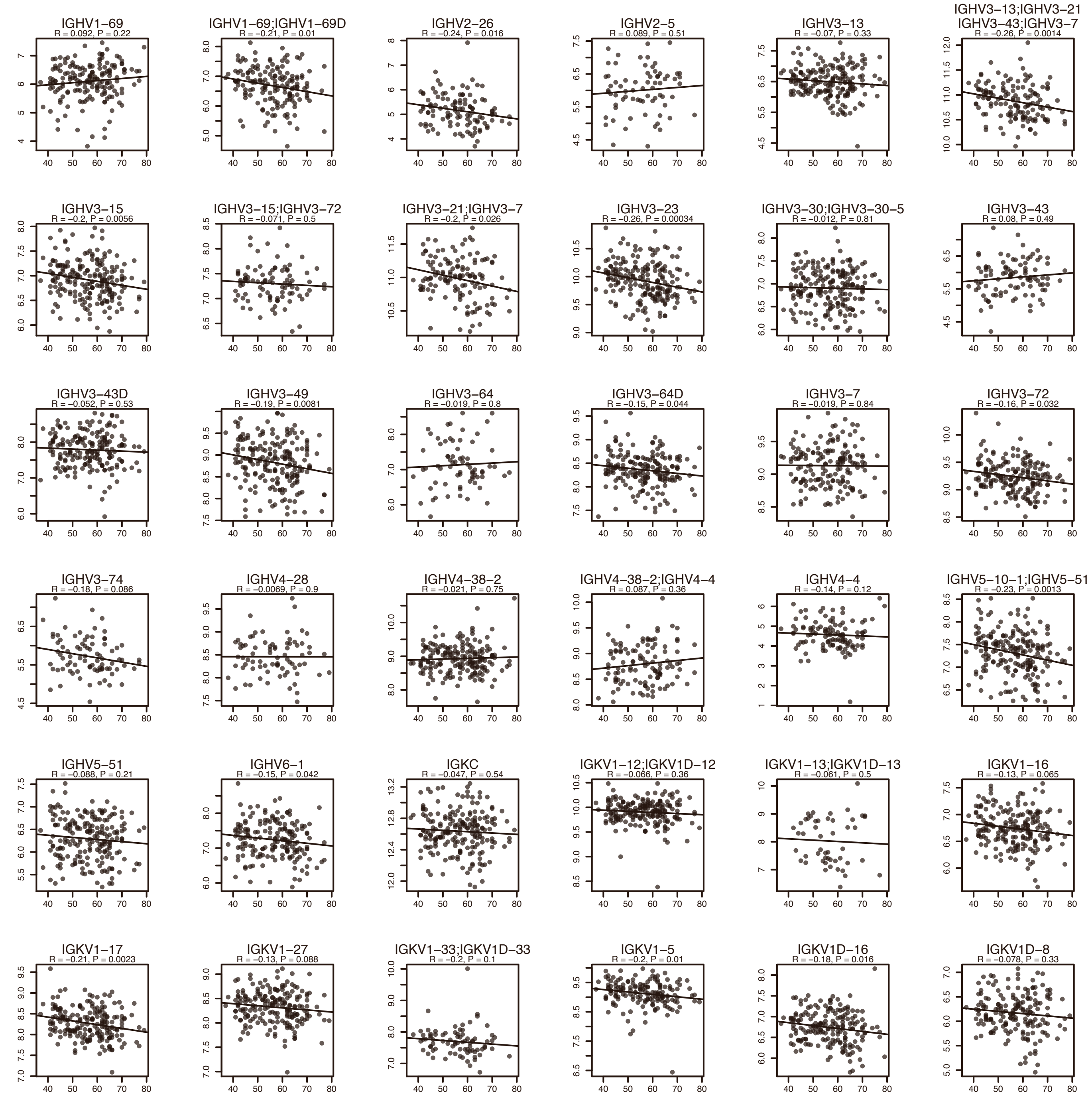
IGHV1−69
R = 0.092, P = 0.22
IGHV1−69;IGHV1−69D
R = −0.21, P = 0.01
IGHV2−26
R = −0.24, P = 0.016
IGHV2−5
R = 0.089, P = 0.51
IGHV3−13
R = −0.07, P = 0.33
IGHV3−13;IGHV3−21
IGHV3−43;IGHV3−7
R = −0.26, P = 0.0014
IGHV3−15
R = −0.2, P = 0.0056
IGHV3−15;IGHV3−72
R = −0.071, P = 0.5
IGHV3−21;IGHV3−7
R = −0.2, P = 0.026
IGHV3−23
R = −0.26, P = 0.00034
IGHV3−30;IGHV3−30−5
R = −0.012, P = 0.81
IGHV3−43
R = 0.08, P = 0.49
IGHV3−43D
R = −0.052, P = 0.53
IGHV3−49
R = −0.19, P = 0.0081
IGHV3−64
R = −0.019, P = 0.8
IGHV3−64D
R = −0.15, P = 0.044
IGHV3−7
R = −0.019, P = 0.84
IGHV3−72
R = −0.16, P = 0.032
IGHV3−74
R = −0.18, P = 0.086
IGHV4−28
R = −0.0069, P = 0.9
IGHV4−38−2
R = −0.021, P = 0.75
IGHV4−38−2;IGHV4−4
R = 0.087, P = 0.36
IGHV4−4
R = −0.14, P = 0.12
IGHV5−10−1;IGHV5−51
R = −0.23, P = 0.0013
IGHV5−51
R = −0.088, P = 0.21
IGHV6−1
R = −0.15, P = 0.042
IGKC
R = −0.047, P = 0.54
IGKV1−12;IGKV1D−12
R = −0.066, P = 0.36
IGKV1−13;IGKV1D−13
R = −0.061, P = 0.5
IGKV1−16
R = −0.13, P = 0.065
IGKV1−17
R = −0.21, P = 0.0023
IGKV1−27
R = −0.13, P = 0.088
IGKV1−33;IGKV1D−33
R = −0.2, P = 0.1
IGKV1−5
R = −0.2, P = 0.01
IGKV1D−16
R = −0.18, P = 0.016
IGKV1D−8
R = −0.078, P = 0.33

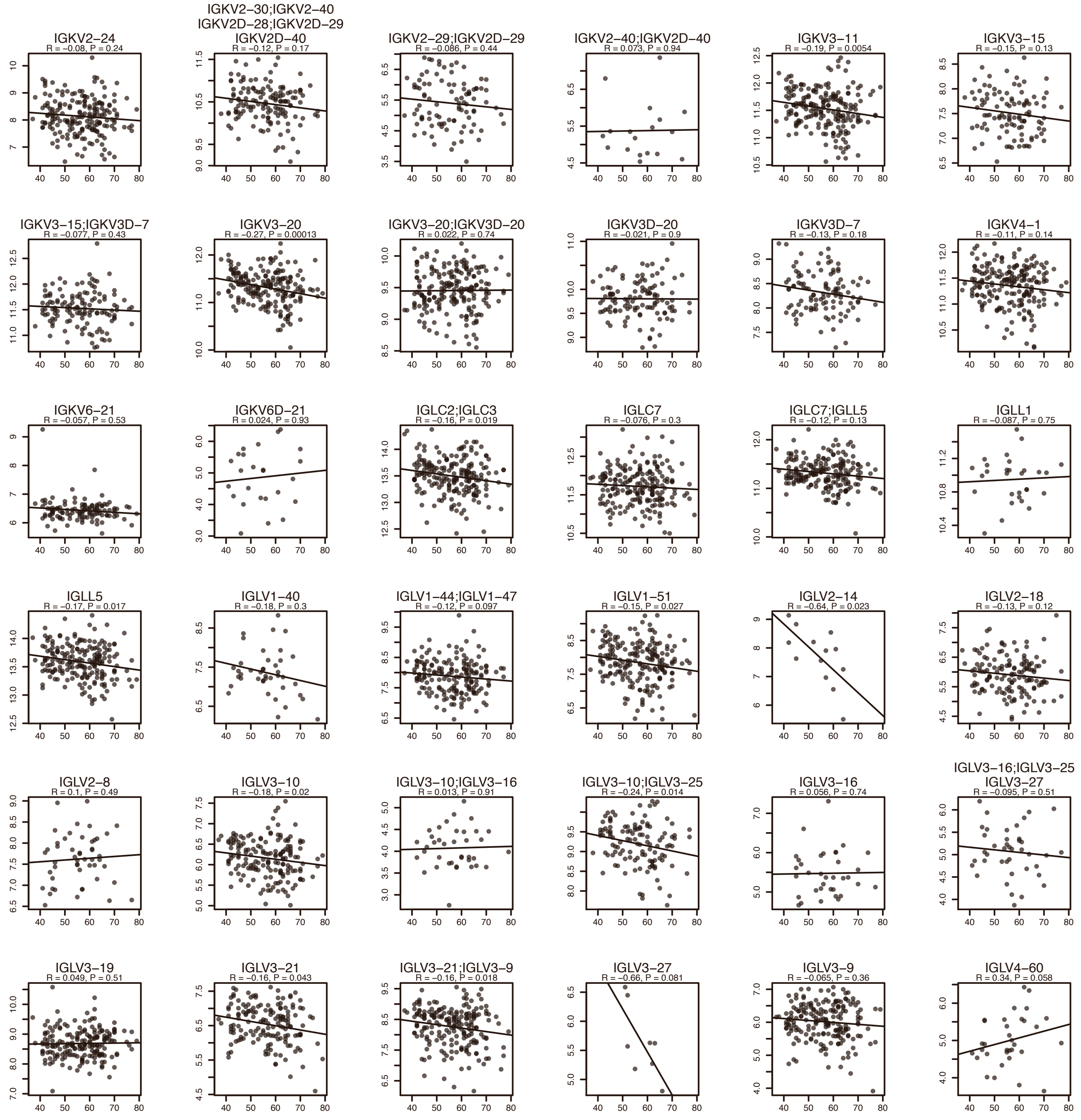
IGKV2-24
R = -0.08, P = 0.24
IGKV2-30;IGKV2-40
IGKV2D-28;IGKV2D-29
IGKV2D-40
R = -0.12, P = 0.17
IGKV2-29;IGKV2D-29
R = -0.086, P = 0.44
IGKV2-40;IGKV2D-40
R = 0.073, P = 0.94
IGKV3-11
R = -0.19, P = 0.0054
IGKV3-15
R = -0.15, P = 0.13
IGKV3-15;IGKV3D-7
R = -0.077, P = 0.43
IGKV3-20
R = -0.27, P = 0.00013
IGKV3-20;IGKV3D-20
R = 0.022, P = 0.74
IGKV3D-20
R = -0.021, P = 0.9
IGKV3D-7
R = -0.13, P = 0.18
IGKV4-1
R = -0.11, P = 0.14
IGKV6-21
R = -0.057, P = 0.53
IGKV6D-21
R = 0.024, P = 0.93
IGLC2;IGLC3
R = -0.16, P = 0.019
IGLC7
R = -0.076, P = 0.3
IGLC7;IGLL5
R = -0.12, P = 0.13
IGLL1
R = -0.087, P = 0.75
IGLL5
R = -0.17, P = 0.017
IGLV1-40
R = -0.18, P = 0.3
IGLV1-44;IGLV1-47
R = -0.12, P = 0.097
IGLV1-51
R = -0.15, P = 0.027
IGLV2-14
R = -0.64, P = 0.023
IGLV2-18
R = -0.13, P = 0.12
IGLV2-8
R = 0.1, P = 0.49
IGLV3-10
R = -0.18, P = 0.02
IGLV3-10;IGLV3-16
R = 0.013, P = 0.91
IGLV3-10;IGLV3-25
R = -0.24, P = 0.014
IGLV3-16
R = 0.056, P = 0.74
IGLV3-16;IGLV3-25
IGLV3-27
R = -0.095, P = 0.51
IGLV3-19
R = 0.049, P = 0.51
IGLV3-21
R = -0.16, P = 0.043
IGLV3-21;IGLV3-9
R = -0.16, P = 0.018
IGLV3-27
R = -0.66, P = 0.081
IGLV3-9
R = -0.065, P = 0.36
IGLV4-60
R = 0.34, P = 0.058

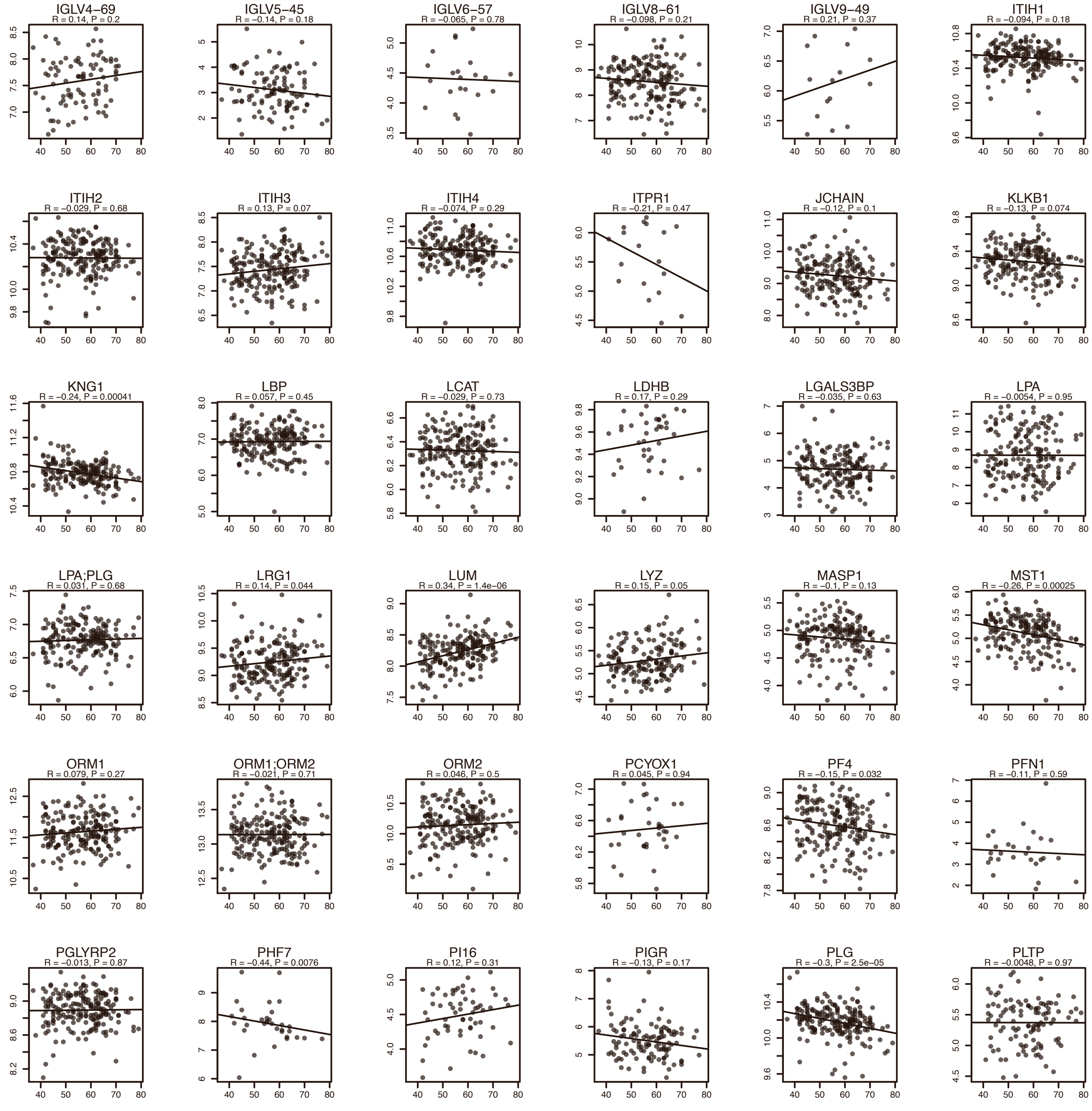
IGLV4−69
R = 0.14, P = 0.2
IGLV5−45
R = −0.14, P = 0.18
IGLV6−57
R = −0.065, P = 0.78
IGLV8−61
R = −0.098, P = 0.21
IGLV9−49
R = 0.21, P = 0.37
ITIH1
R = −0.094, P = 0.18
ITIH2
R = −0.029, P = 0.68
ITIH3
R = 0.13, P = 0.07
ITIH4
R = −0.074, P = 0.29
ITPR1
R = −0.21, P = 0.47
JCHAIN
R = −0.12, P = 0.1
KLKB1
R = −0.13, P = 0.074
KNG1
R = −0.24, P = 0.00041
LBP
R = 0.057, P = 0.45
LCAT
R = −0.029, P = 0.73
LDHB
R = 0.17, P = 0.29
LGALS3BP
R = −0.035, P = 0.63
LPA
R = −0.0054, P = 0.95
LPA;PLG
R = 0.031, P = 0.68
LRG1
R = 0.14, P = 0.044
LUM
R = 0.34, P = 1.4e−06
LYZ
R = 0.15, P = 0.05
MASP1
R = −0.1, P = 0.13
MST1
R = −0.26, P = 0.00025
ORM1
R = 0.079, P = 0.27
ORM1;ORM2
R = −0.021, P = 0.71
ORM2
R = 0.046, P = 0.5
PCYOX1
R = 0.045, P = 0.94
PF4
R = −0.15, P = 0.032
PFN1
R = −0.11, P = 0.59
PGLYRP2
R = −0.013, P = 0.87
PHF7
R = −0.44, P = 0.0076
PI16
R = 0.12, P = 0.31
PIGR
R = −0.13, P = 0.17
PLG
R = −0.3, P = 2.5e−05
PLTP
R = −0.0048, P = 0.97

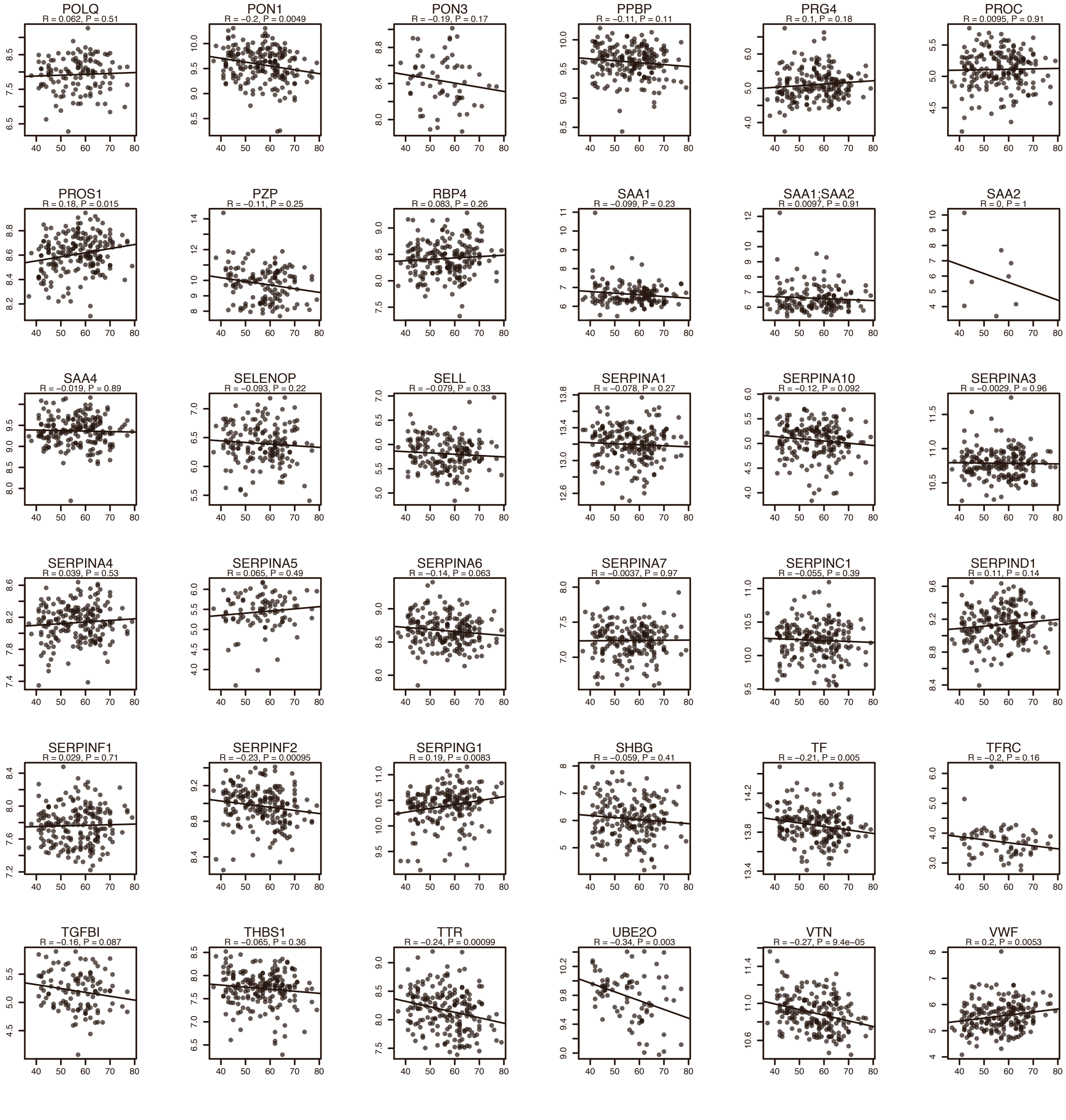

POLQ
R = 0.062, P = 0.51
PON1
R = −0.2, P = 0.0049
PON3
R = −0.19, P = 0.17
PPBP
R = −0.11, P = 0.11
PRG4
R = 0.1, P = 0.18
PROC
R = 0.0095, P = 0.91
PROS1
R = 0.18, P = 0.015
PZP
R = −0.11, P = 0.25
RBP4
R = 0.083, P = 0.26
SAA1
R = −0.099, P = 0.23
SAA1;SAA2
R = 0.0097, P = 0.91
SAA2
R = 0, P = 1
SAA4
R = −0.019, P = 0.89
SELENOP
R = −0.093, P = 0.22
SELL
R = −0.079, P = 0.33
SERPINA1
R = −0.078, P = 0.27
SERPINA10
R = −0.12, P = 0.092
SERPINA3
R = −0.0029, P = 0.96
SERPINA4
R = 0.039, P = 0.53
SERPINA5
R = 0.065, P = 0.49
SERPINA6
R = −0.14, P = 0.063
SERPINA7
R = −0.0037, P = 0.97
SERPINC1
R = −0.055, P = 0.39
SERPIND1
R = 0.11, P = 0.14
SERPINF1
R = 0.029, P = 0.71
SERPINF2
R = −0.23, P = 0.00095
SERPING1
R = 0.19, P = 0.0083
SHBG
R = −0.059, P = 0.41
TF
R = −0.21, P = 0.005
TFRC
R = −0.2, P = 0.16
TGFBI
R = −0.16, P = 0.087
THBS1
R = −0.065, P = 0.36
TTR
R = −0.24, P = 0.00099
UBE2O
R = −0.34, P = 0.003
VTN
R = −0.27, P = 9.4e−05
VWF
R = 0.2, P = 0.0053

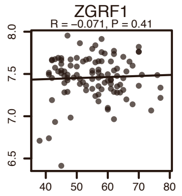

ZGRF1
R = −0.071, P = 0.41
8.0
7.5
7.0
6.5
40
50
60
70
80

Figure S11 (following 2 pages). **Omics features significantly regulated depending on age**

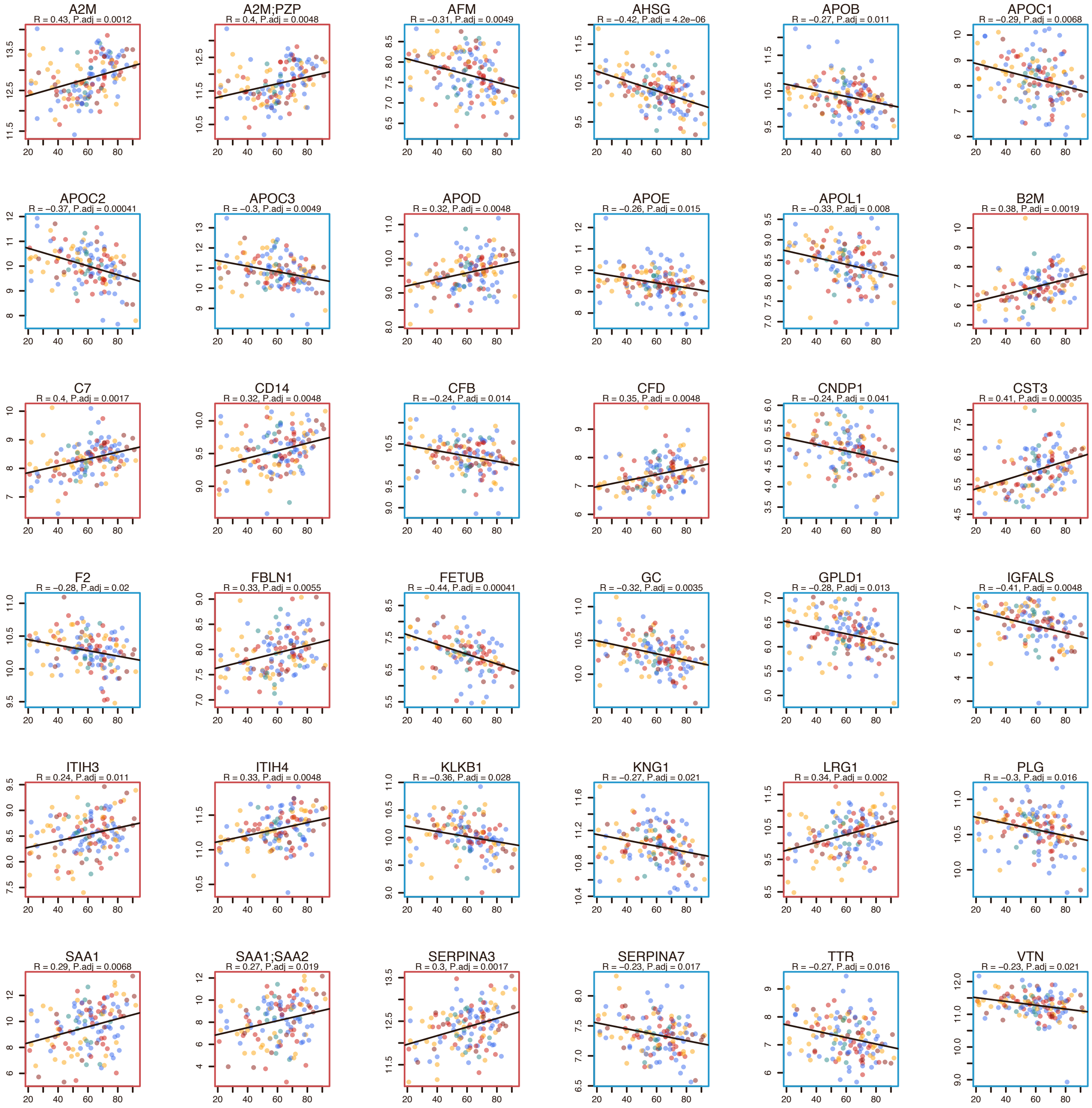

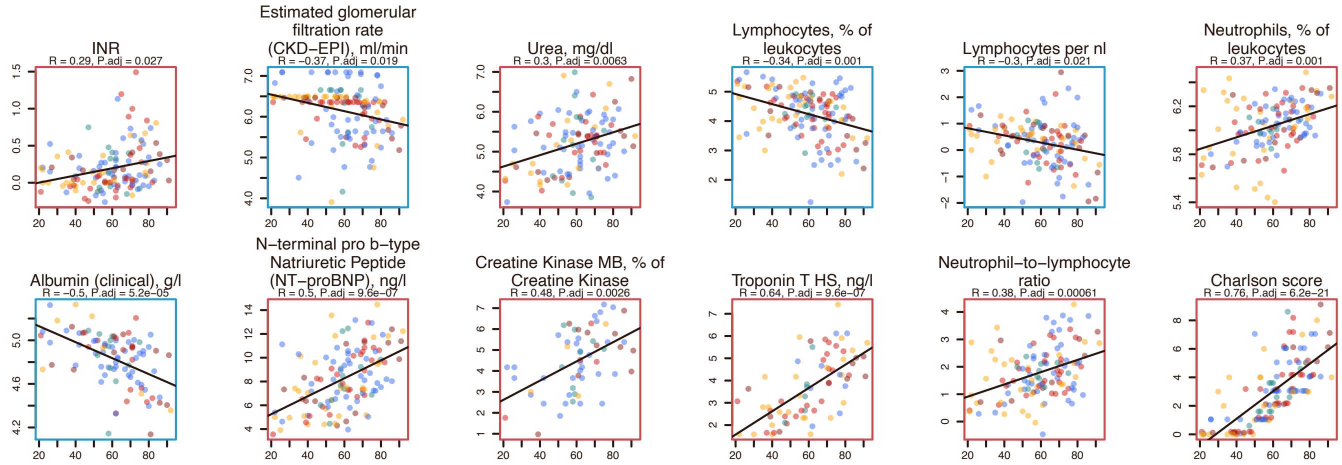

INR
R = 0.29, P.adj = 0.027
Estimated glomerular filtration rate (CKD–EPI), ml/min
R = −0.37, P.adj = 0.019
Urea, mg/dl
R = 0.3, P.adj = 0.0063
Lymphocytes, % of leukocytes
R = −0.34, P.adj = 0.001
Lymphocytes per nl
R = −0.3, P.adj = 0.021
Neutrophils, % of leukocytes
R = 0.37, P.adj = 0.001
Albumin (clinical), g/l
R = −0.5, P.adj = 5.2e−05
N–terminal pro b–type Natriuretic Peptide (NT–proBNP), ng/l
R = 0.5, P.adj = 9.6e−07
Creatine Kinase MB, % of Creatine Kinase
R = 0.48, P.adj = 0.0026
Troponin T HS, ng/l
R = 0.64, P.adj = 9.6e−07
Neutrophil–to–lymphocyte ratio
R = 0.38, P.adj = 0.00061
Charlson score
R = 0.76, P.adj = 6.2e−21

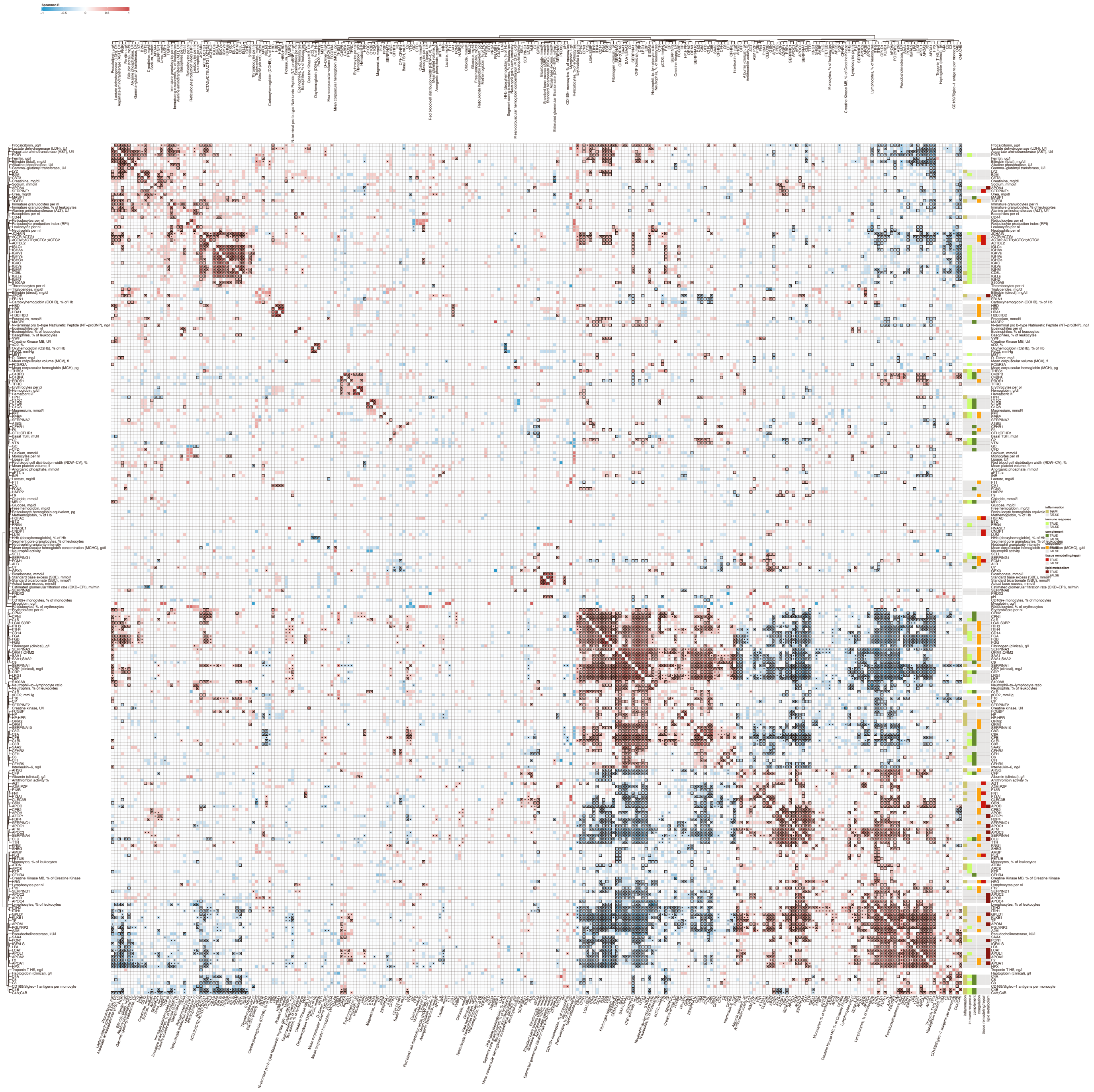

Figure S12. **Map of correlations between dynamic changes in omics measurements during the peak period of the disease**

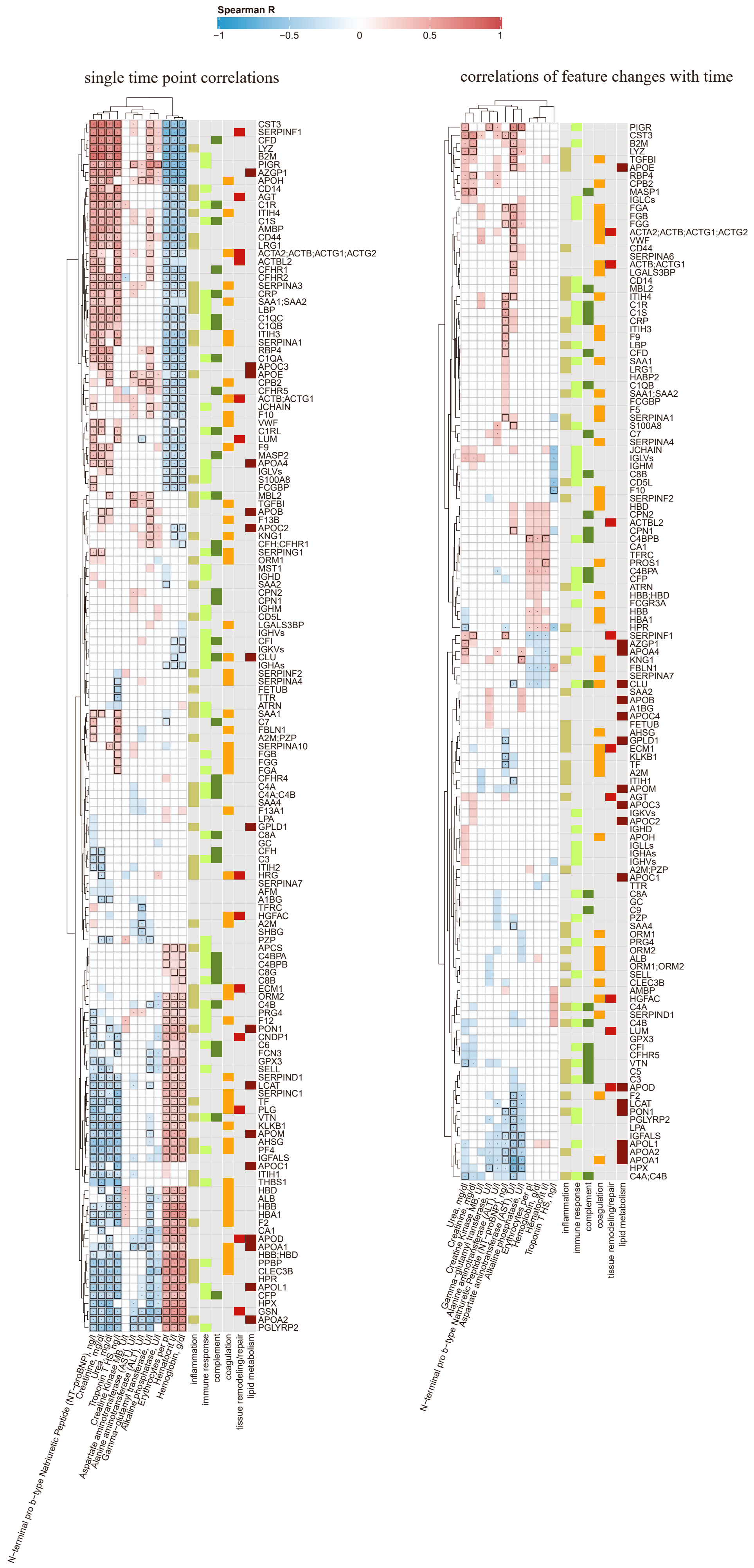

Figure S13. **Covariation of organ function markers and plasma proteins**

Figure S14 (following 4 pages). **Omics features changing during the peak of the disease**

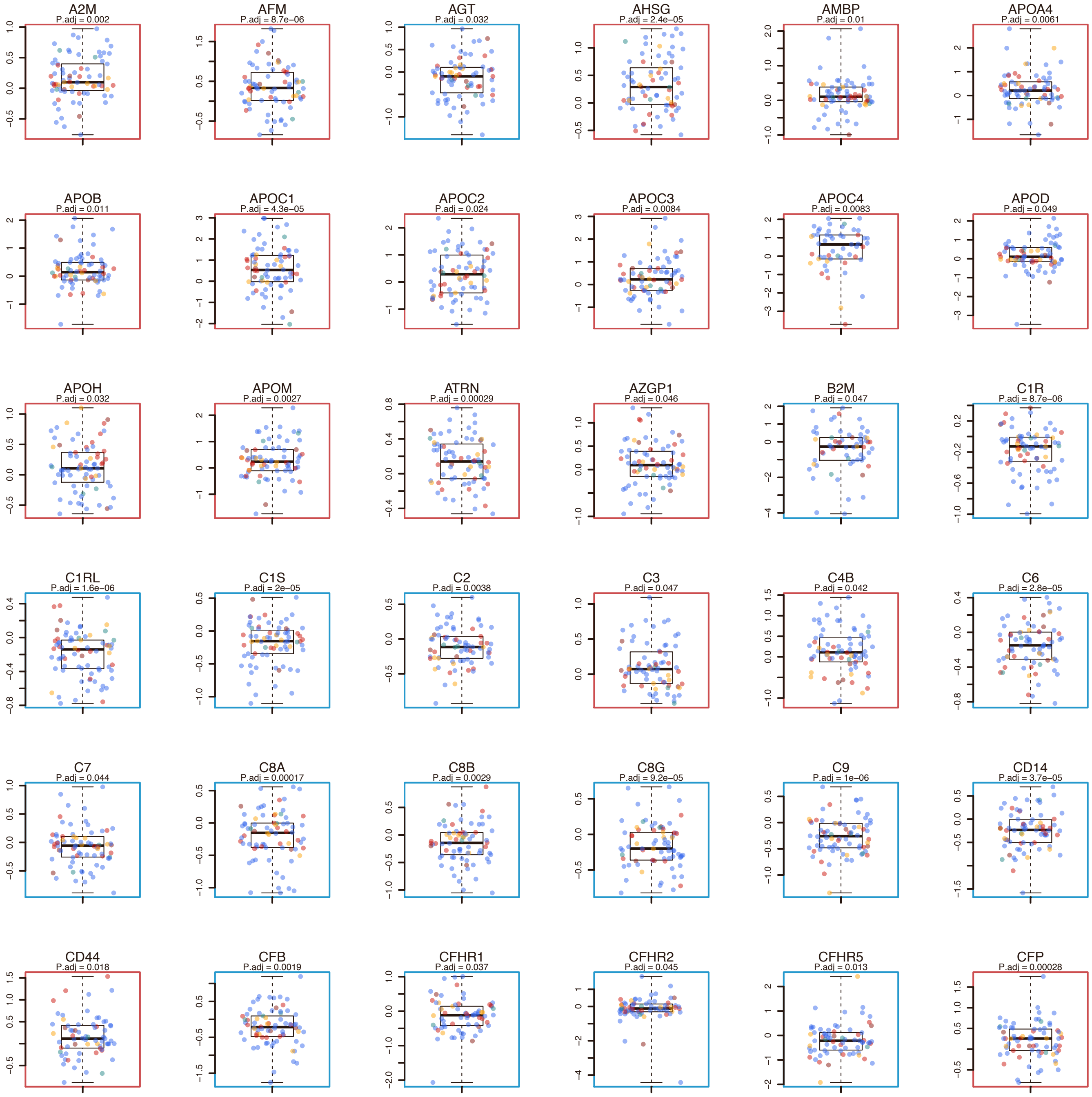

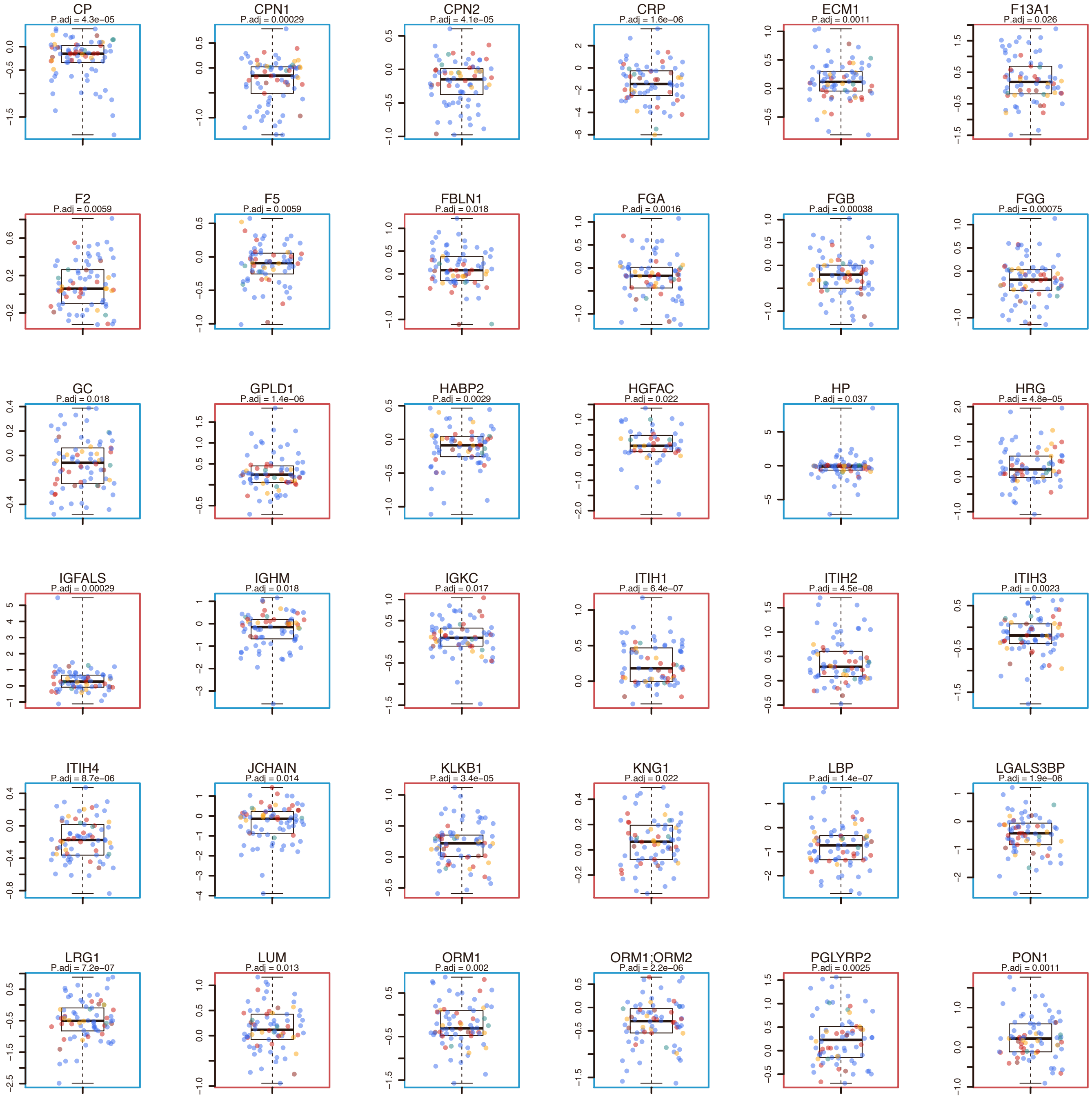

CP
P.adj = 4.3e−05
CPN1
P.adj = 0.00029
CPN2
P.adj = 4.1e−05
CRP
P.adj = 1.6e−06
ECM1
P.adj = 0.0011
F13A1
P.adj = 0.026
F2
P.adj = 0.0059
F5
P.adj = 0.0059
FBLN1
P.adj = 0.018
FGA
P.adj = 0.0016
FGB
P.adj = 0.00038
FGG
P.adj = 0.00075
GC
P.adj = 0.018
GPLD1
P.adj = 1.4e−06
HABP2
P.adj = 0.0029
HGFAC
P.adj = 0.022
HP
P.adj = 0.037
HRG
P.adj = 4.8e−05
IGFALS
P.adj = 0.00029
IGHM
P.adj = 0.018
IGKC
P.adj = 0.017
ITIH1
P.adj = 6.4e−07
ITIH2
P.adj = 4.5e−08
ITIH3
P.adj = 0.0023
ITIH4
P.adj = 8.7e−06
JCHAIN
P.adj = 0.014
KLKB1
P.adj = 3.4e−05
KNG1
P.adj = 0.022
LBP
P.adj = 1.4e−07
LGALS3BP
P.adj = 1.9e−06
LRG1
P.adj = 7.2e−07
LUM
P.adj = 0.013
ORM1
P.adj = 0.002
ORM1;ORM2
P.adj = 2.2e−06
PGLYRP2
P.adj = 0.0025
PON1
P.adj = 0.0011

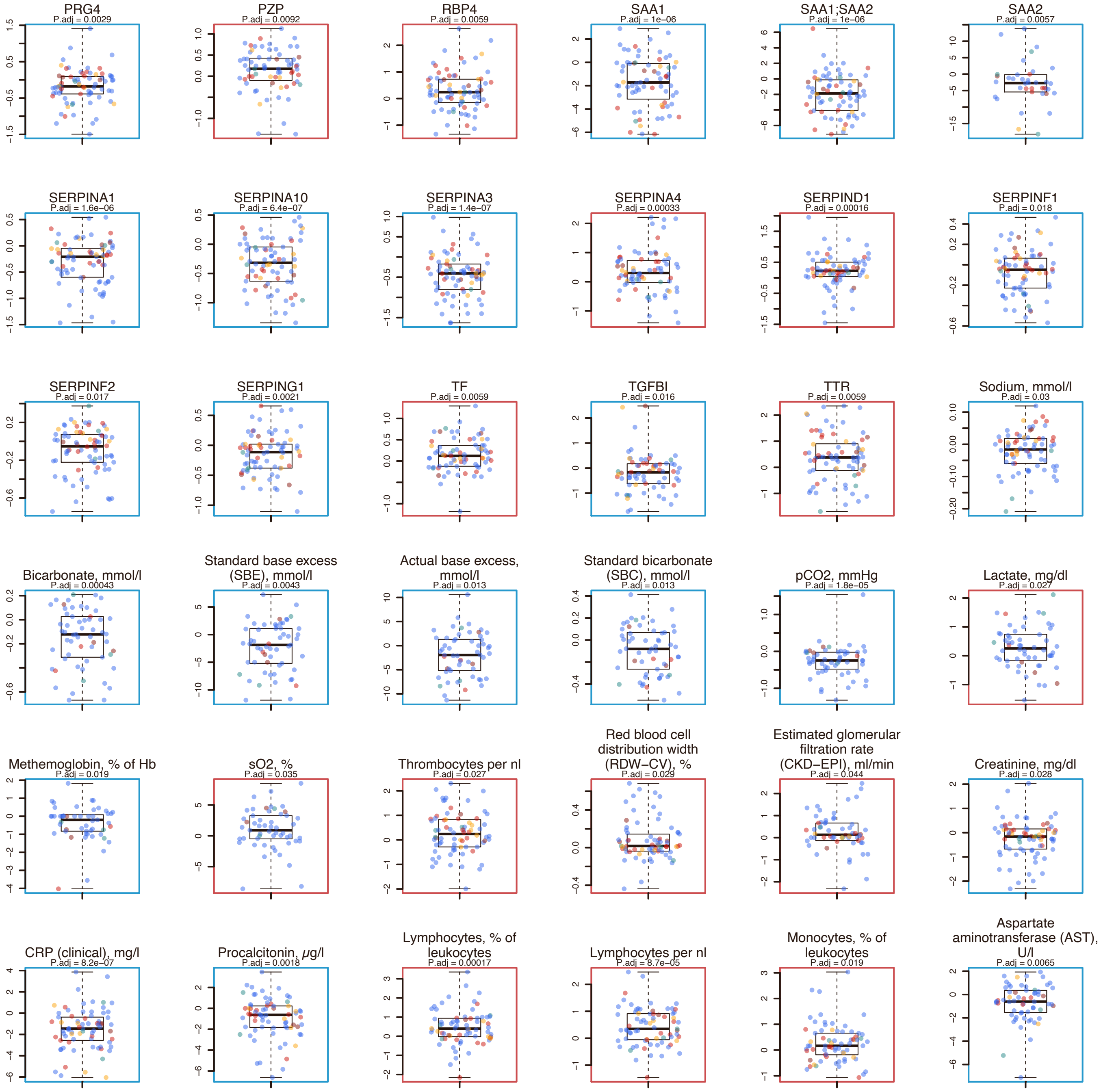
PRG4
P.adj = 0.0029
PZP
P.adj = 0.0092
RBP4
P.adj = 0.0059
SAA1
P.adj = 1e−06
SAA1;SAA2
P.adj = 1e−06
SAA2
P.adj = 0.0057
SERPINA1
P.adj = 1.6e−06
SERPINA10
P.adj = 6.4e−07
SERPINA3
P.adj = 1.4e−07
SERPINA4
P.adj = 0.00033
SERPIND1
P.adj = 0.00016
SERPINF1
P.adj = 0.018
SERPINF2
P.adj = 0.017
SERPING1
P.adj = 0.0021
TF
P.adj = 0.0059
TGFBI
P.adj = 0.016
TTR
P.adj = 0.0059
Sodium, mmol/l
P.adj = 0.03
Bicarbonate, mmol/l
P.adj = 0.00043
Standard base excess (SBE), mmol/l
P.adj = 0.0043
Actual base excess, mmol/l
P.adj = 0.013
Standard bicarbonate (SBC), mmol/l
P.adj = 0.013
pCO2, mmHg
P.adj = 1.8e−05
Lactate, mg/dl
P.adj = 0.027
Methemoglobin, % of Hb
P.adj = 0.019
sO2, %
P.adj = 0.035
Thrombocytes per nl
P.adj = 0.027
Red blood cell distribution width (RDW−CV), %
P.adj = 0.029
Estimated glomerular filtration rate (CKD−EPI), ml/min
P.adj = 0.044
Creatinine, mg/dl
P.adj = 0.028
CRP (clinical), mg/l
P.adj = 8.2e−07
Procalcitonin, µg/l
P.adj = 0.0018
Lymphocytes, % of leukocytes
P.adj = 0.00017
Lymphocytes per nl
P.adj = 8.7e−05
Monocytes, % of leukocytes
P.adj = 0.019
Aspartate aminotransferase (AST), U/l
P.adj = 0.0065

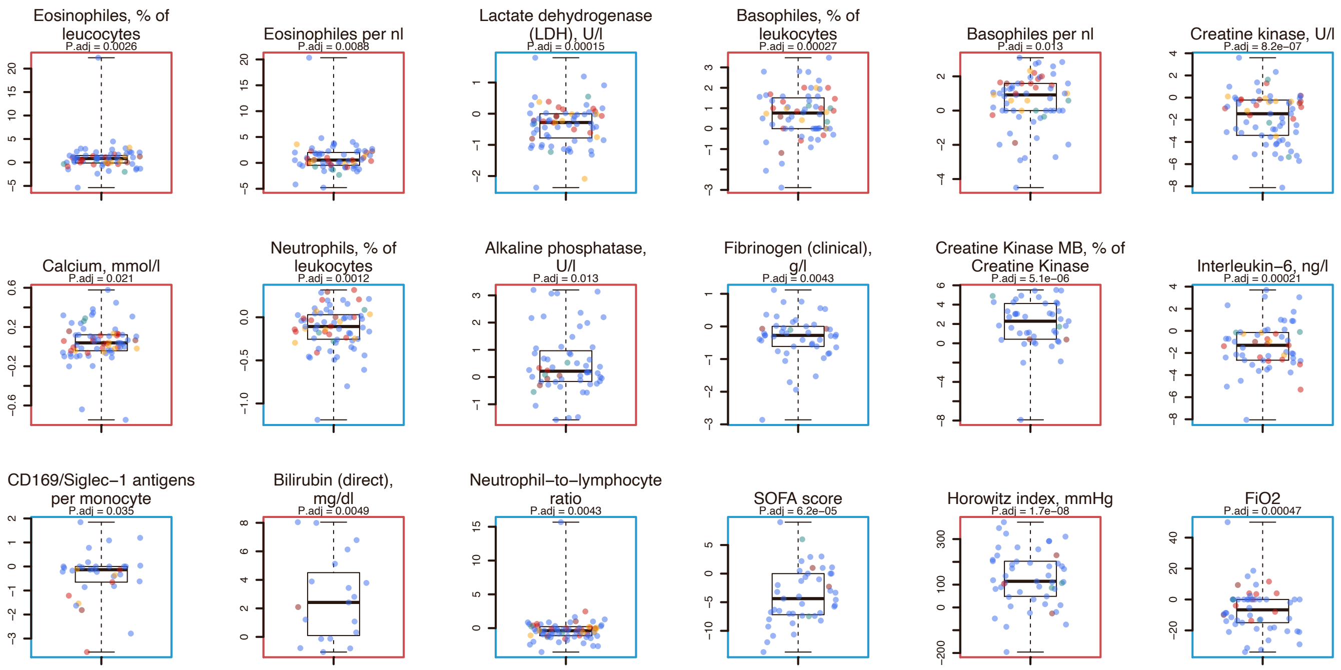

Eosinophiles, % of leucocytes
P.adj = 0.0026
Eosinophiles per nl
P.adj = 0.0088
Lactate dehydrogenase (LDH), U/l
P.adj = 0.00015
Basophiles, % of leukocytes
P.adj = 0.00027
Basophiles per nl
P.adj = 0.013
Creatine kinase, U/l
P.adj = 8.2e−07
Calcium, mmol/l
P.adj = 0.021
Neutrophils, % of leukocytes
P.adj = 0.0012
Alkaline phosphatase, U/l
P.adj = 0.013
Fibrinogen (clinical), g/l
P.adj = 0.0043
Creatine Kinase MB, % of Creatine Kinase
P.adj = 5.1e−06
Interleukin−6, ng/l
P.adj = 0.00021
CD169/Siglec−1 antigens per monocyte
P.adj = 0.035
Bilirubin (direct), mg/dl
P.adj = 0.0049
Neutrophil−to−lymphocyte ratio
P.adj = 0.0043
SOFA score
P.adj = 6.2e−05
Horowitz index, mmHg
P.adj = 1.7e−08
FiO2
P.adj = 0.00047

Figure S15. **Omics features differentially changing during the peak of the disease depending on the severity**

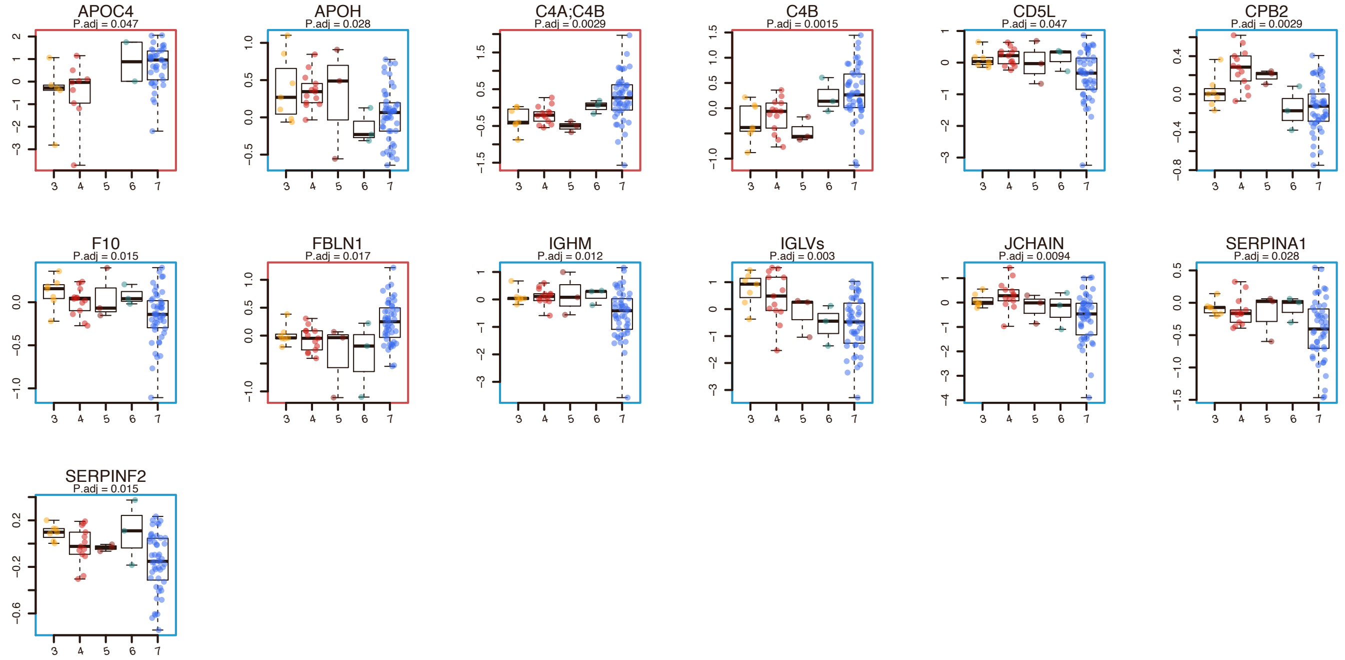

Figure S16 (following 9 pages). **Trajectories at the peak of the disease**

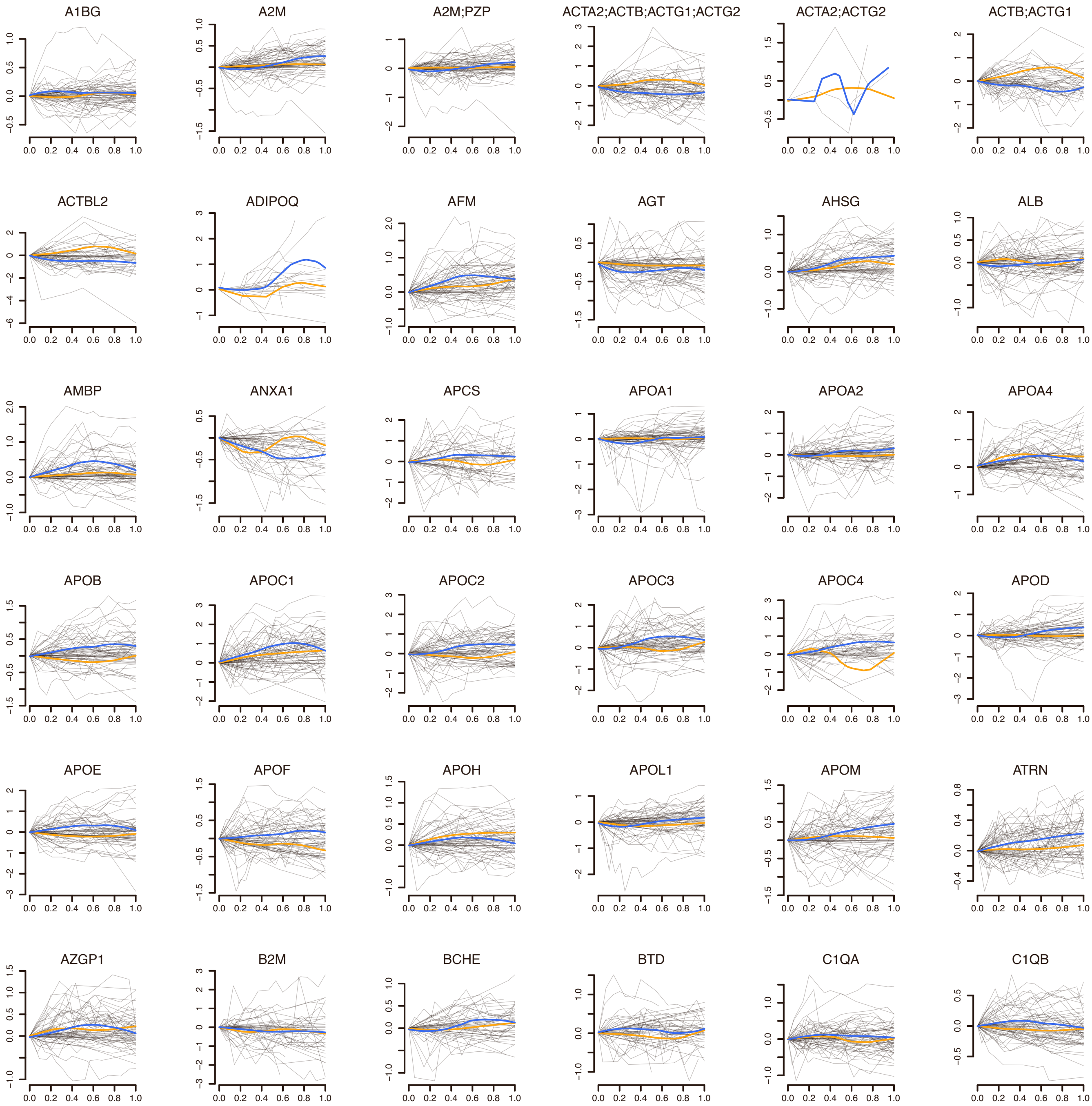

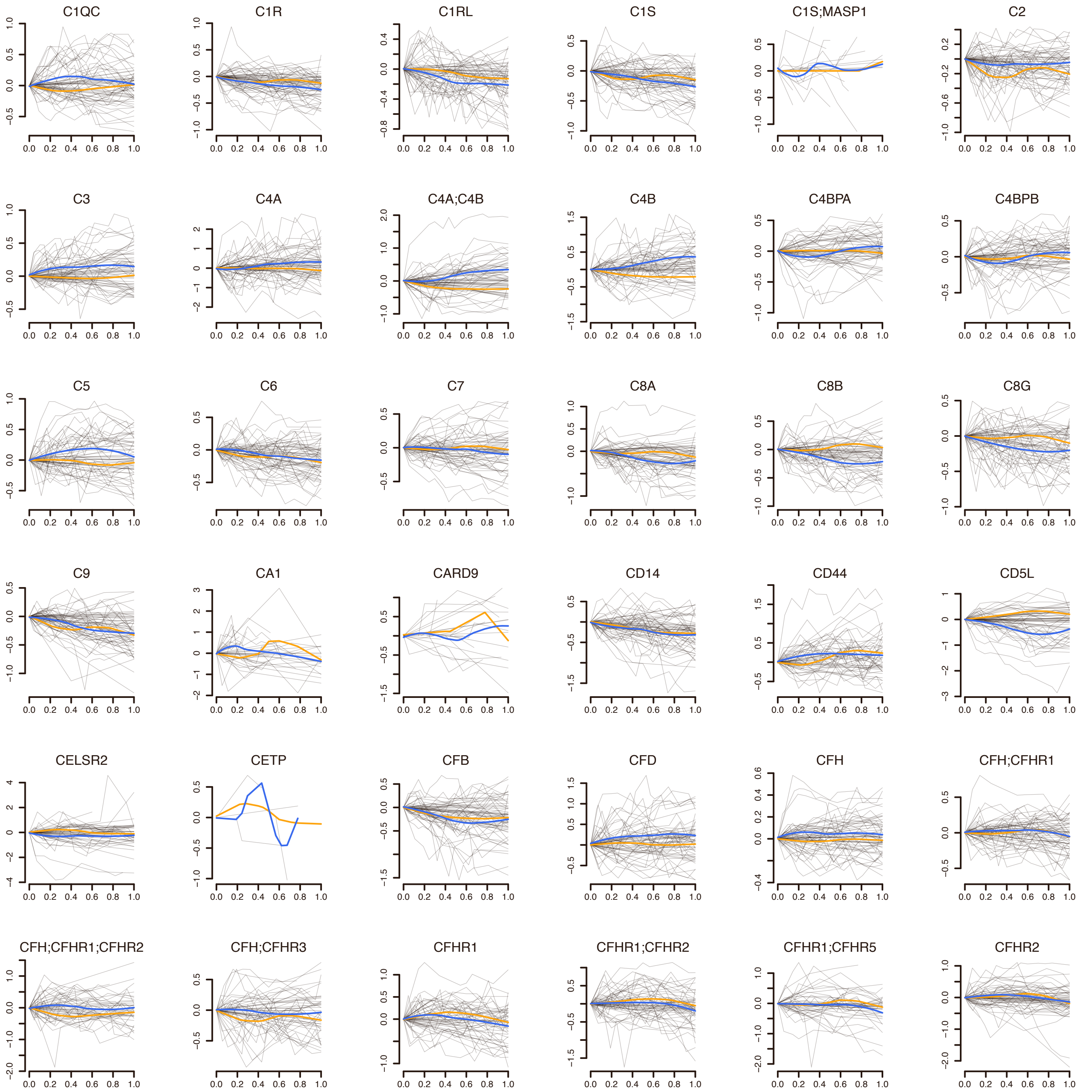
C1QC
C1R
C1RL
C1S
C1S;MASP1
C2
C3
C4A
C4A;C4B
C4B
C4BPA
C4BPB
C5
C6
C7
C8A
C8B
C8G
C9
CA1
CARD9
CD14
CD44
CD5L
CELSR2
CETP
CFB
CFD
CFH
CFH;CFHR1
CFH;CFHR1;CFHR2
CFH;CFHR3
CFHR1
CFHR1;CFHR2
CFHR1;CFHR5
CFHR2

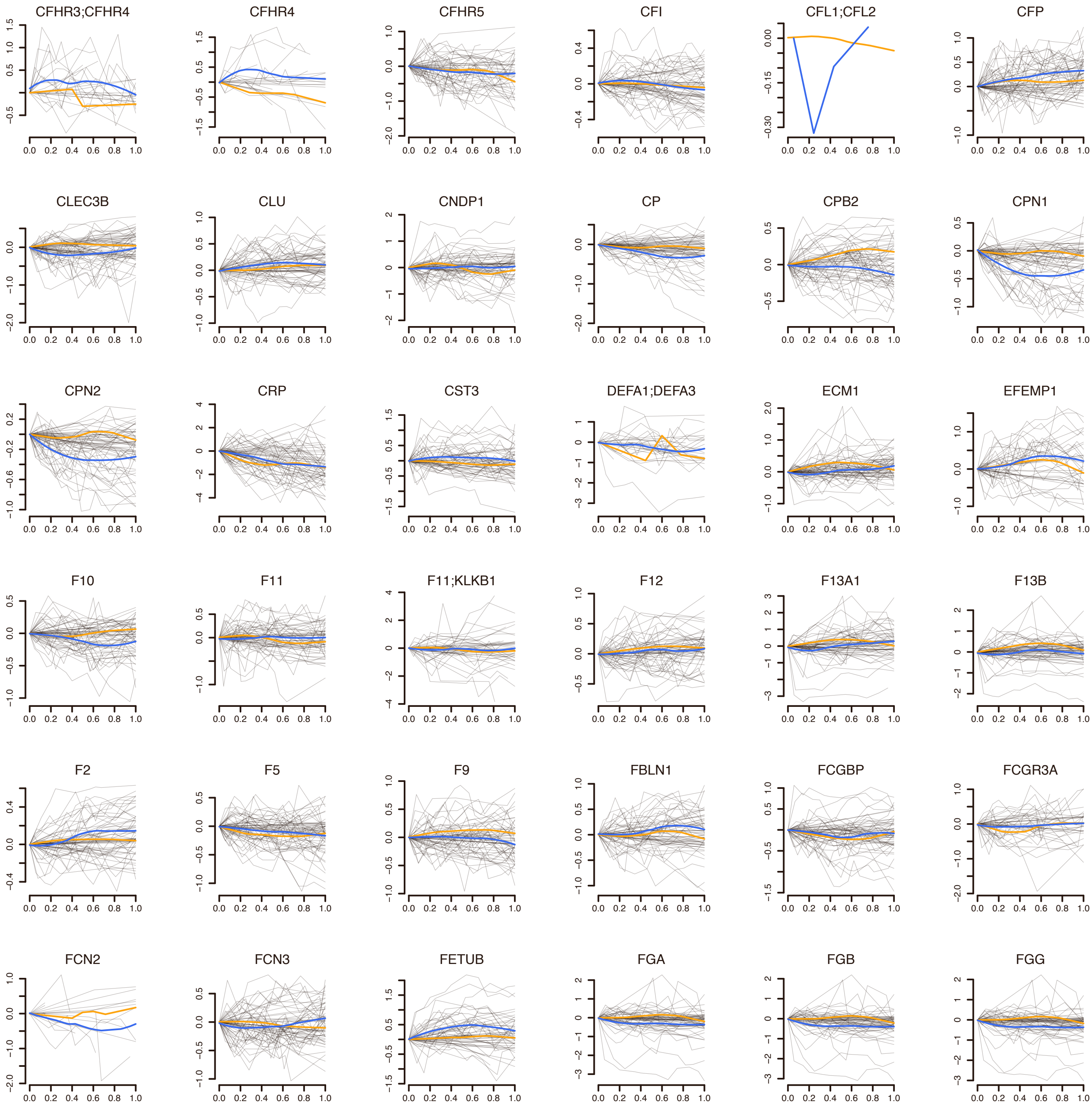
CFHR3;CFHR4
CFHR4
CFHR5
CFI
CFL1;CFL2
CFP
CLEC3B
CLU
CNDP1
CP
CPB2
CPN1
CPN2
CRP
CST3
DEFA1;DEFA3
ECM1
EFEMP1
F10
F11
F11;KLKB1
F12
F13A1
F13B
F2
F5
F9
FBLN1
FCGBP
FCGR3A
FCN2
FCN3
FETUB
FGA
FGB
FGG

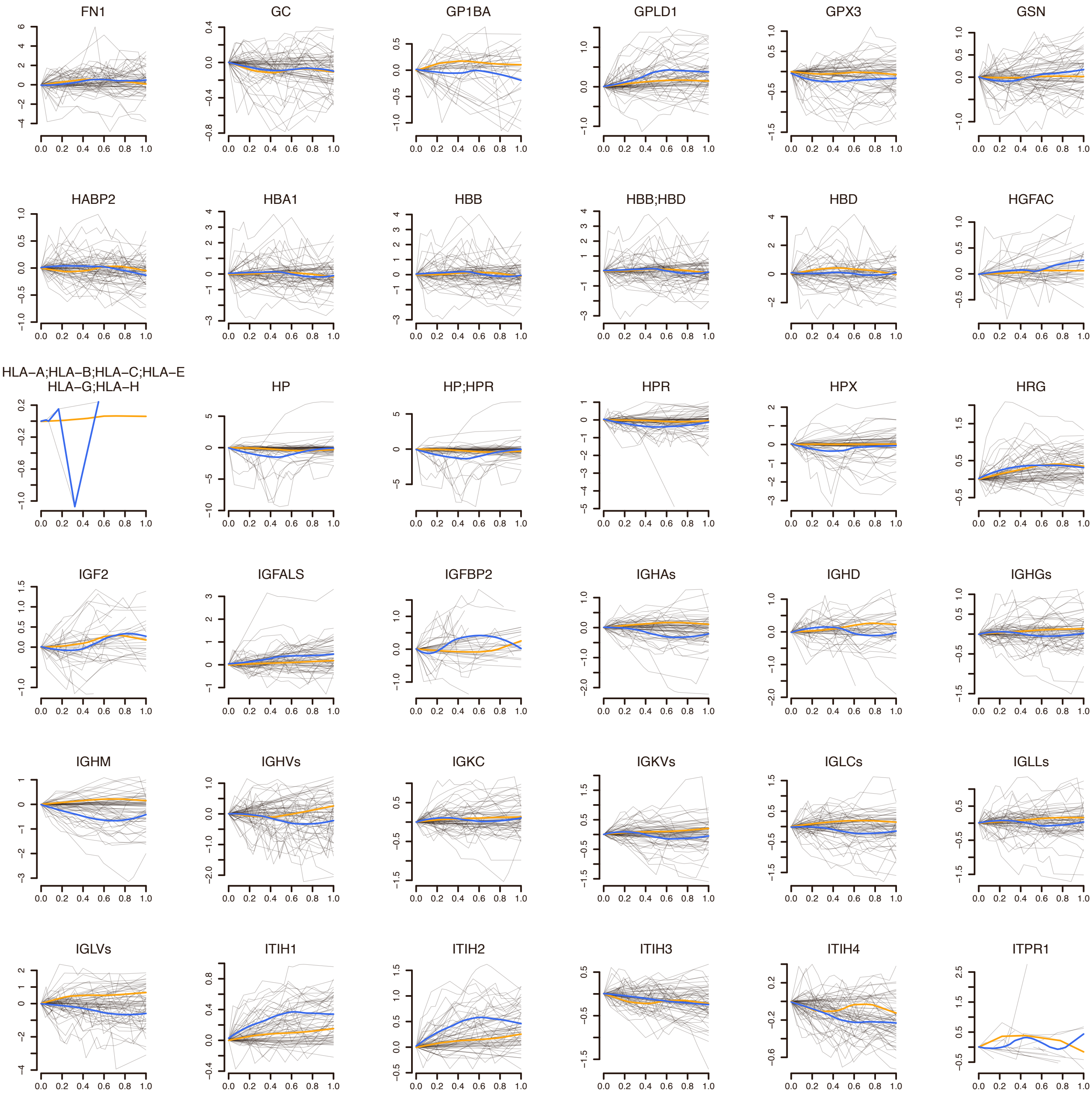
FN1
GC
GP1BA
GPLD1
GPX3
GSN
HABP2
HBA1
HBB
HBB;HBD
HBD
HGFAC
HLA−A;HLA−B;HLA−C;HLA−E
HLA−G;HLA−H
HP
HP;HPR
HPR
HPX
HRG
IGF2
IGFALS
IGFBP2
IGHAs
IGHD
IGHGs
IGHM
IGHVs
IGKC
IGKVs
IGLCs
IGLLs
IGLVs
ITIH1
ITIH2
ITIH3
ITIH4
ITPR1

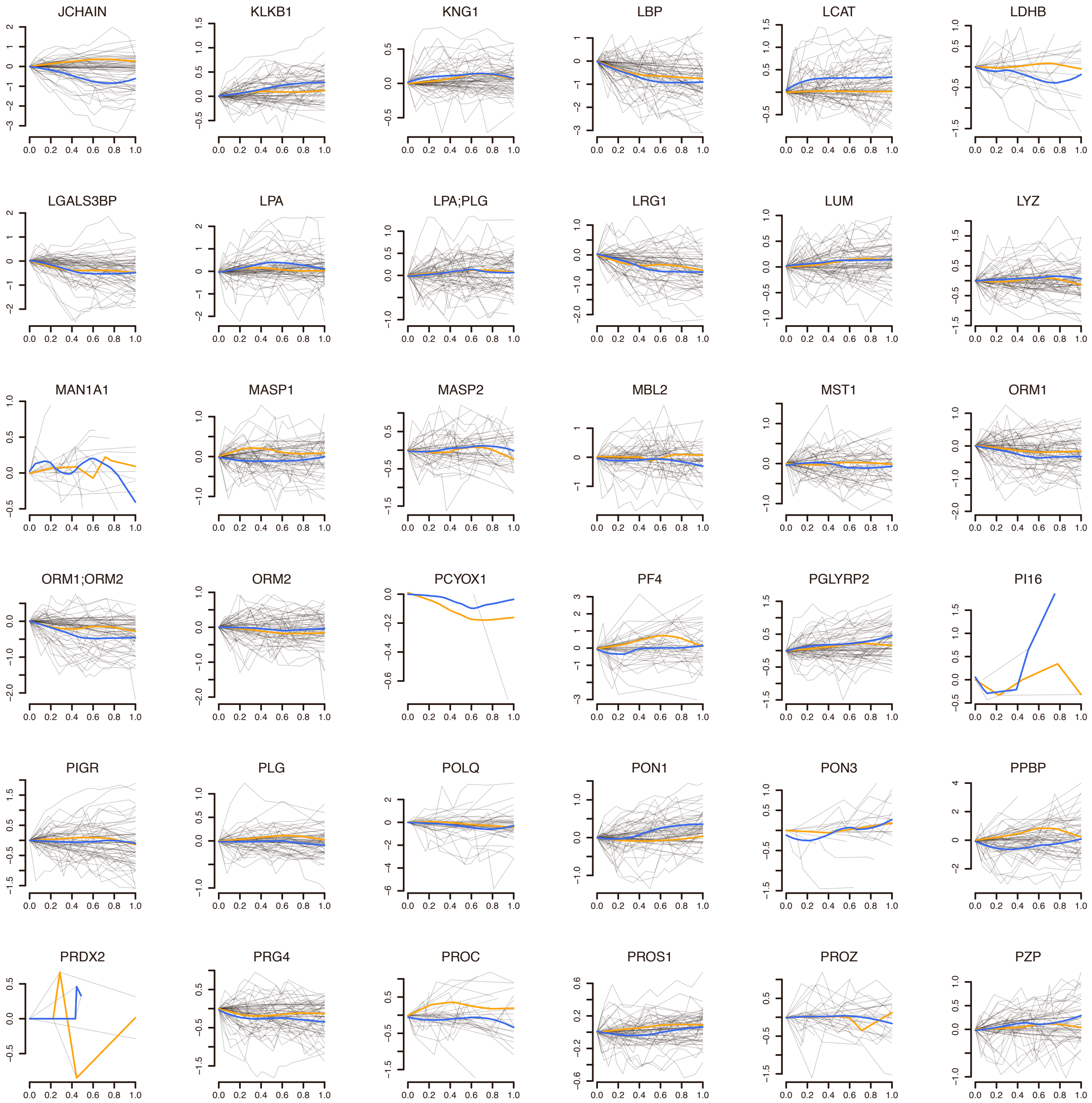
JCHAIN
KLKB1
KNG1
LBP
LCAT
LDHB
LGALS3BP
LPA
LPA;PLG
LRG1
LUM
LYZ
MAN1A1
MASP1
MASP2
MBL2
MST1
ORM1
ORM1;ORM2
ORM2
PCYOX1
PF4
PGLYRP2
PI16
PIGR
PLG
POLQ
PON1
PON3
PPBP
PRDX2
PRG4
PROC
PROS1
PROZ
PZP

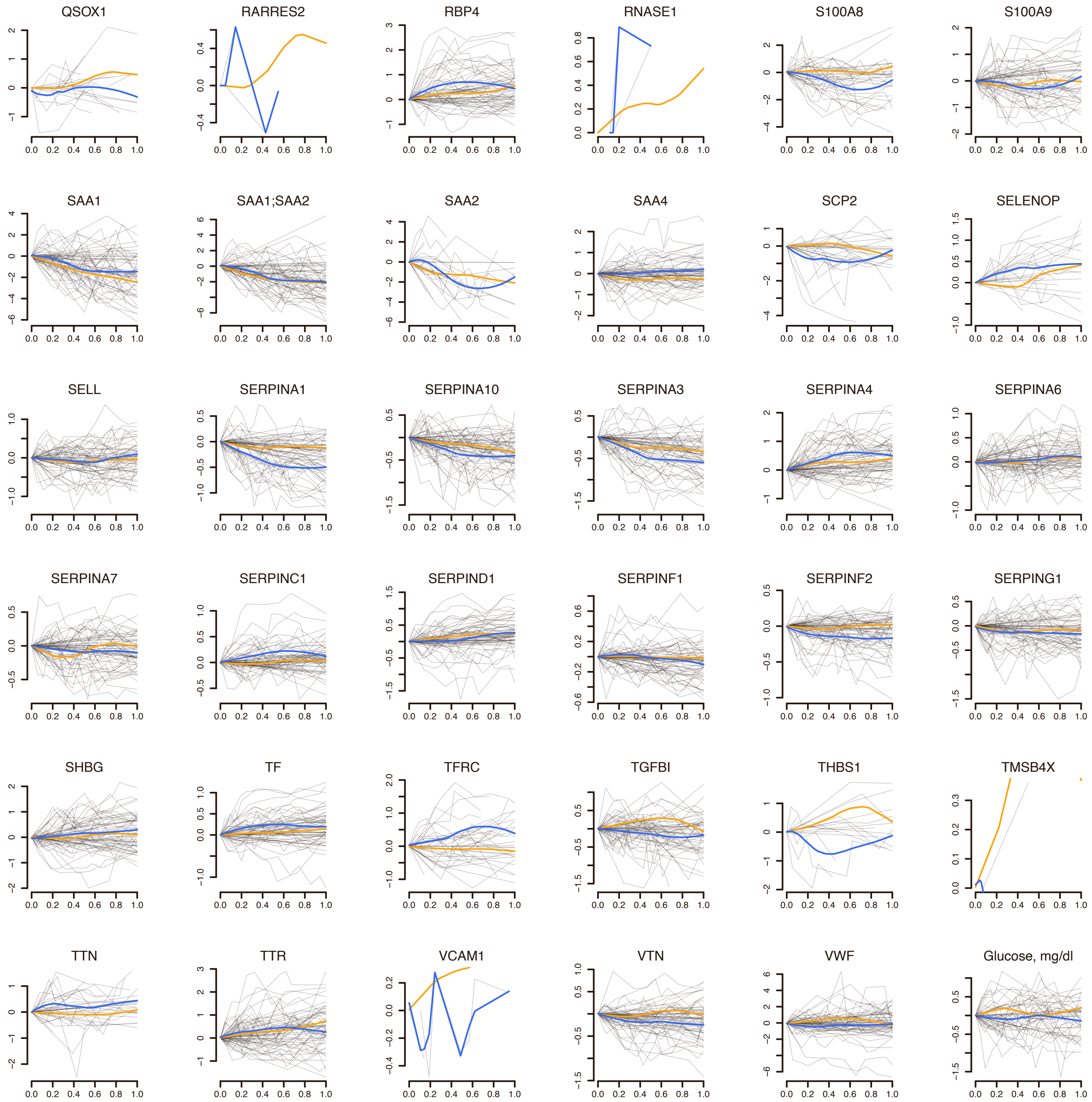
QSOX1
RARRES2
RBP4
RNASE1
S100A8
S100A9
SAA1
SAA1;SAA2
SAA2
SAA4
SCP2
SELENOP
SELL
SERPINA1
SERPINA10
SERPINA3
SERPINA4
SERPINA6
SERPINA7
SERPINC1
SERPIND1
SERPINF1
SERPINF2
SERPING1
SHBG
TF
TFRC
TGFBI
THBS1
TMSB4X
TTN
TTR
VCAM1
VTN
VWF
Glucose, mg/dl

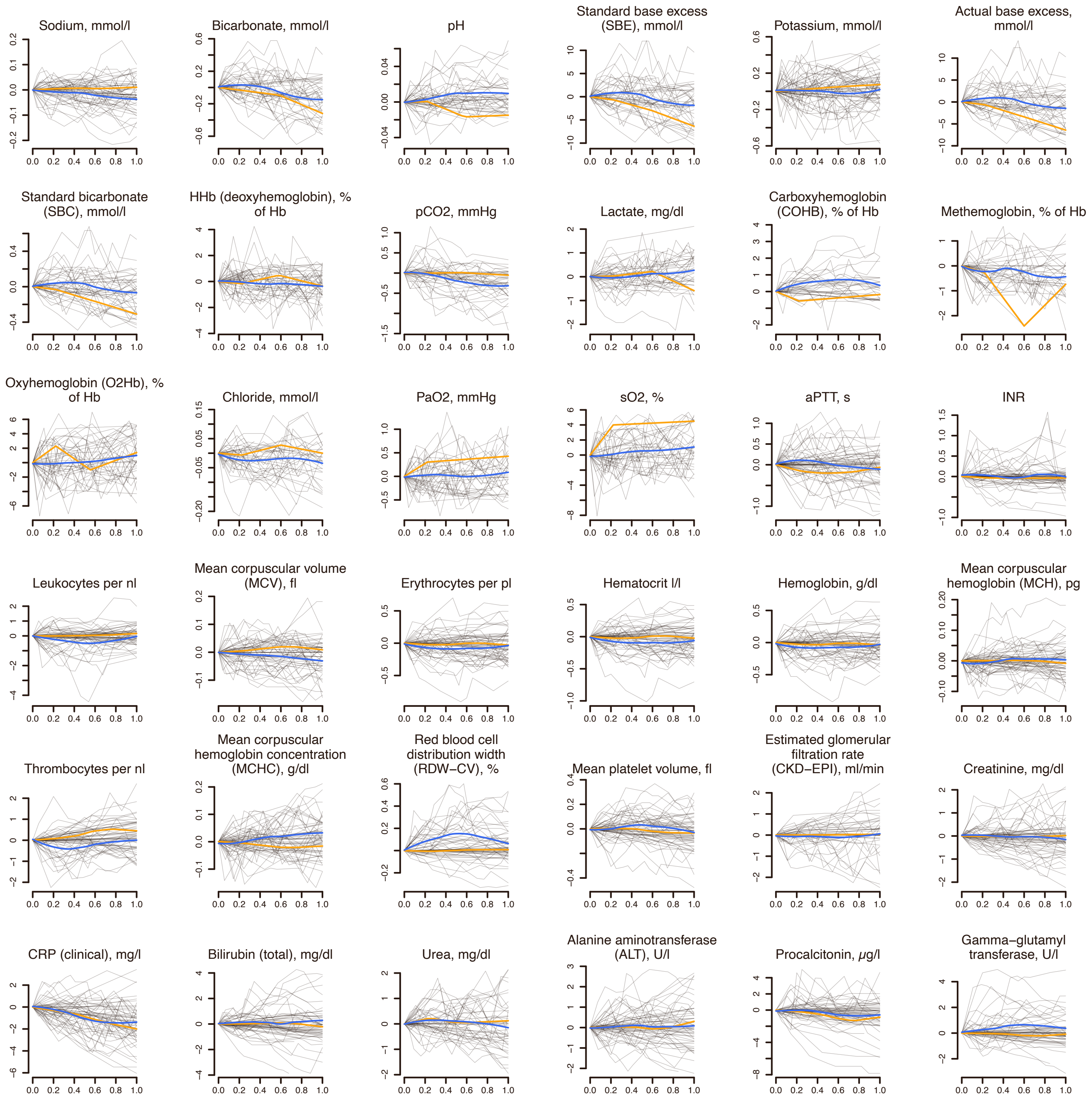
Sodium, mmol/l
Bicarbonate, mmol/l
pH
Standard base excess (SBE), mmol/l
Potassium, mmol/l
Actual base excess, mmol/l
Standard bicarbonate (SBC), mmol/l
HHb (deoxyhemoglobin), % of Hb
pCO2, mmHg
Lactate, mg/dl
Carboxyhemoglobin (COHB), % of Hb
Methemoglobin, % of Hb
Oxyhemoglobin (O2Hb), % of Hb
Chloride, mmol/l
PaO2, mmHg
sO2, %
aPTT, s
INR
Leukocytes per nl
Mean corpuscular volume (MCV), fl
Erythrocytes per pl
Hematocrit l/l
Hemoglobin, g/dl
Mean corpuscular hemoglobin (MCH), pg
Thrombocytes per nl
Mean corpuscular hemoglobin concentration (MCHC), g/dl
Red blood cell distribution width (RDW−CV), %
Mean platelet volume, fl
Estimated glomerular filtration rate (CKD−EPI), ml/min
Creatinine, mg/dl
CRP (clinical), mg/l
Bilirubin (total), mg/dl
Urea, mg/dl
Alanine aminotransferase (ALT), U/l
Procalcitonin, μg/l
Gamma−glutamyl transferase, U/l

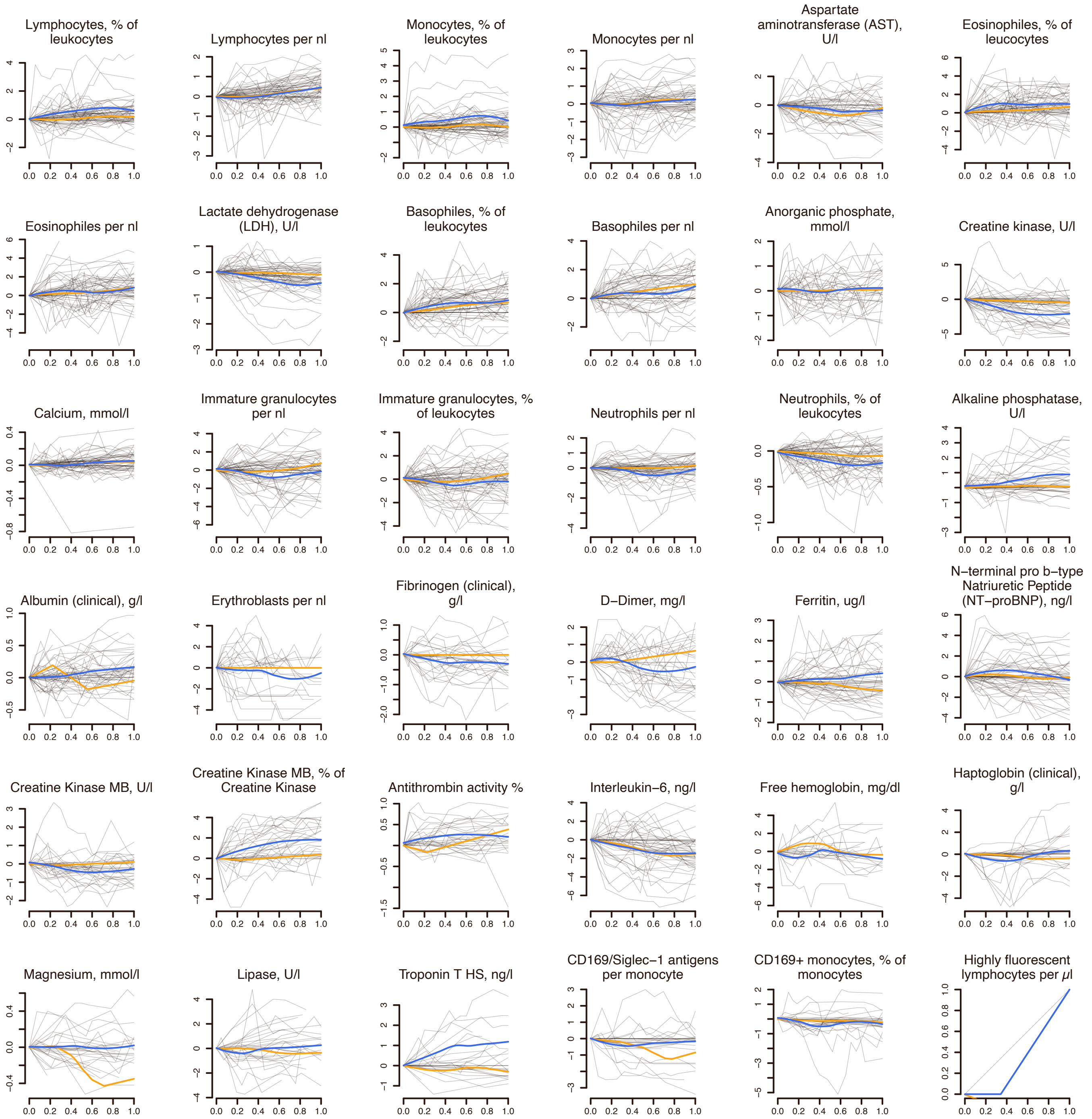
Lymphocytes, % of leukocytes
Lymphocytes per nl
Monocytes, % of leukocytes
Monocytes per nl
Aspartate aminotransferase (AST), U/l
Eosinophiles, % of leucocytes
Eosinophiles per nl
Lactate dehydrogenase (LDH), U/l
Basophiles, % of leukocytes
Basophiles per nl
Anorganic phosphate, mmol/l
Creatine kinase, U/l
Calcium, mmol/l
Immature granulocytes per nl
Immature granulocytes, % of leukocytes
Neutrophils per nl
Neutrophils, % of leukocytes
Alkaline phosphatase, U/l
Albumin (clinical), g/l
Erythroblasts per nl
Fibrinogen (clinical), g/l
D−Dimer, mg/l
Ferritin, ug/l
N−terminal pro b−type Natriuretic Peptide (NT−proBNP), ng/l
Creatine Kinase MB, U/l
Creatine Kinase MB, % of Creatine Kinase
Antithrombin activity %
Interleukin−6, ng/l
Free hemoglobin, mg/dl
Haptoglobin (clinical), g/l
Magnesium, mmol/l
Lipase, U/l
Troponin T HS, ng/l
CD169/Siglec−1 antigens per monocyte
CD169+ monocytes, % of monocytes
Highly fluorescent lymphocytes per μl

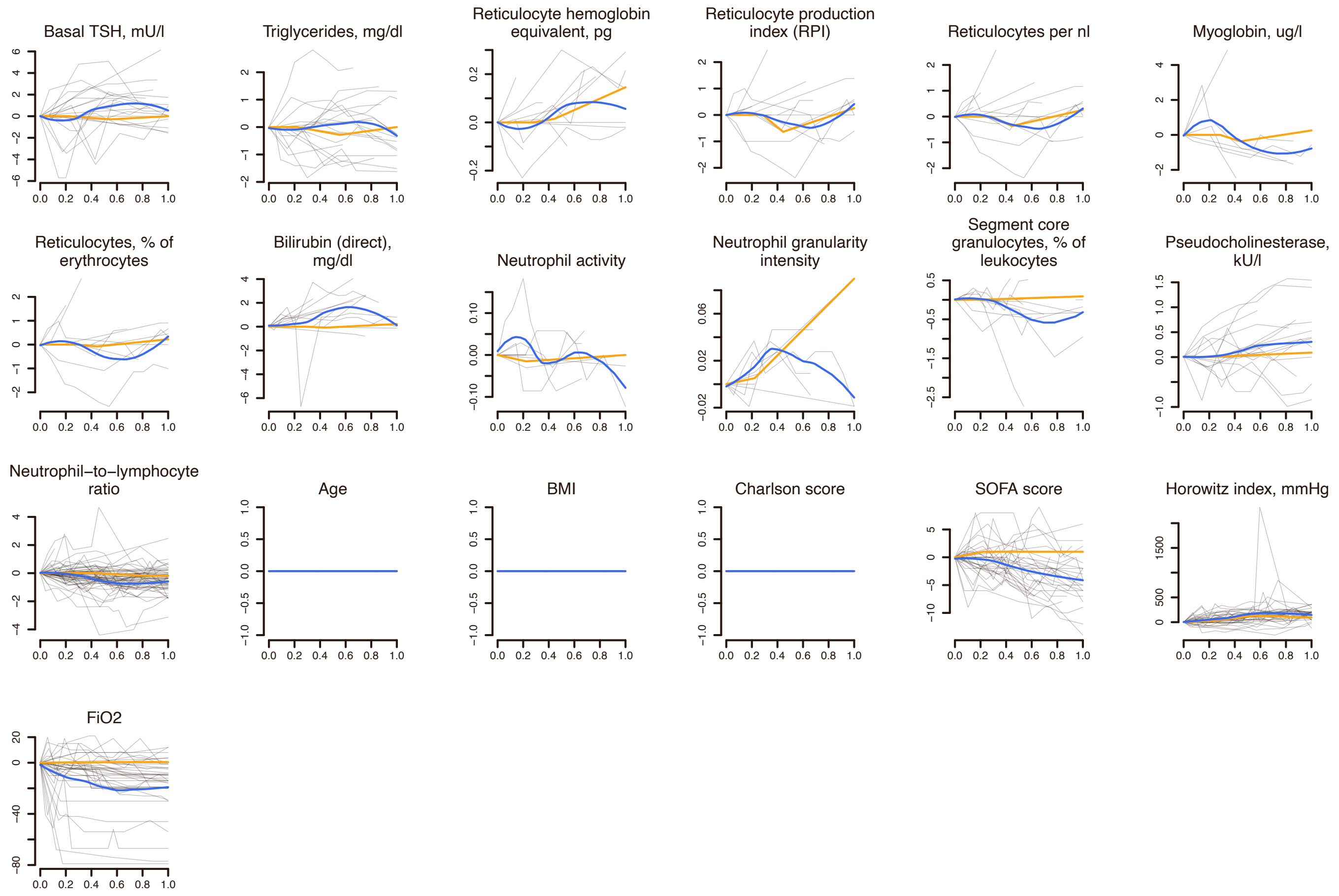

Basal TSH, mU/l
Triglycerides, mg/dl
Reticulocyte hemoglobin equivalent, pg
Reticulocyte production index (RPI)
Reticulocytes per nl
Myoglobin, ug/l
Reticulocytes, % of erythrocytes
Bilirubin (direct), mg/dl
Neutrophil activity
Neutrophil granularity intensity
Segment core granulocytes, % of leukocytes
Pseudocholinesterase, kU/l
Neutrophil−to−lymphocyte ratio
Age
BMI
Charlson score
SOFA score
Horowitz index, mmHg
FiO2

Figure S17. **Omics features predictive of future disease worsening**

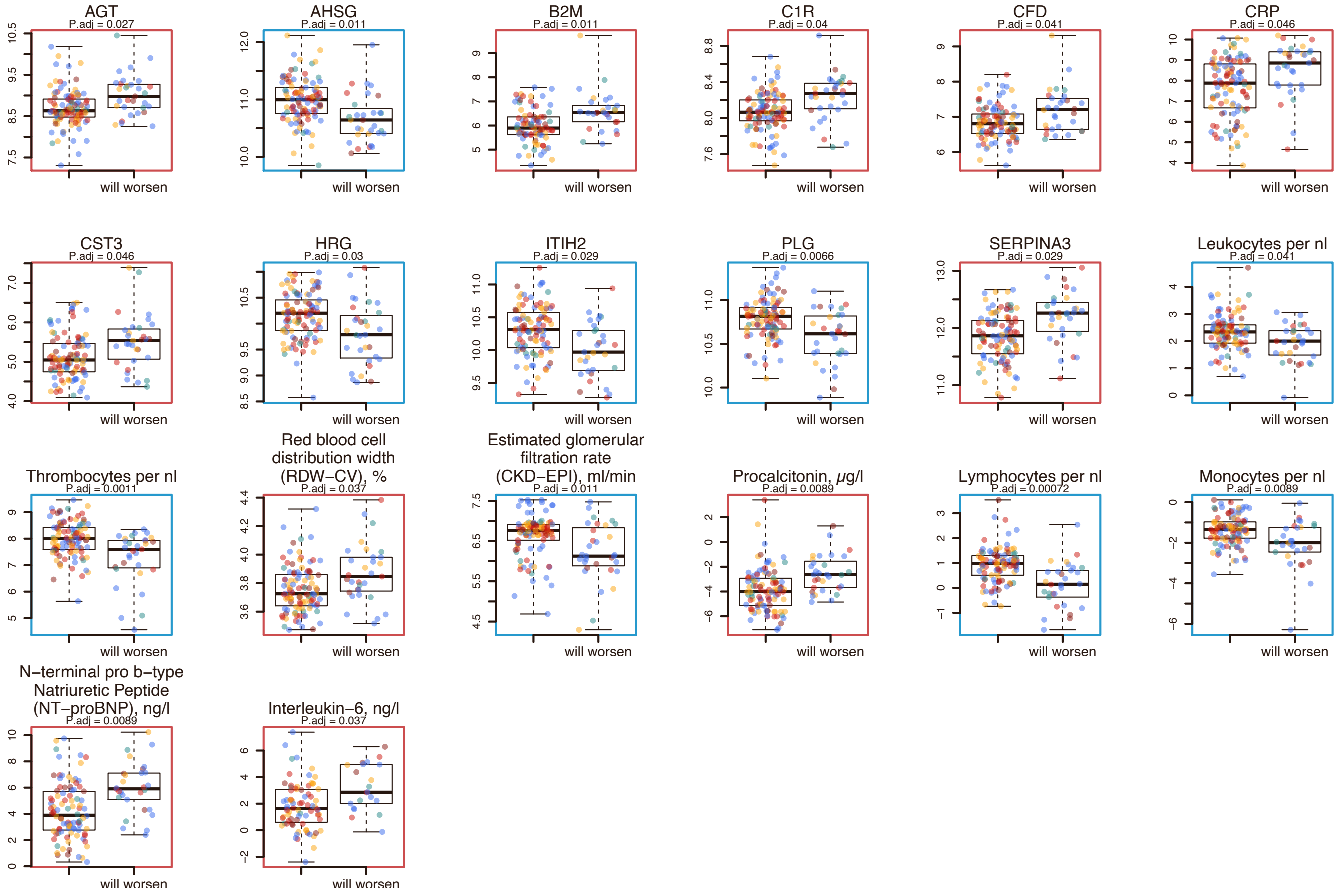

Figure S18 (following 2 pages). **Omics features predictive of the remaining time in hospital for patients at WHO = 3**

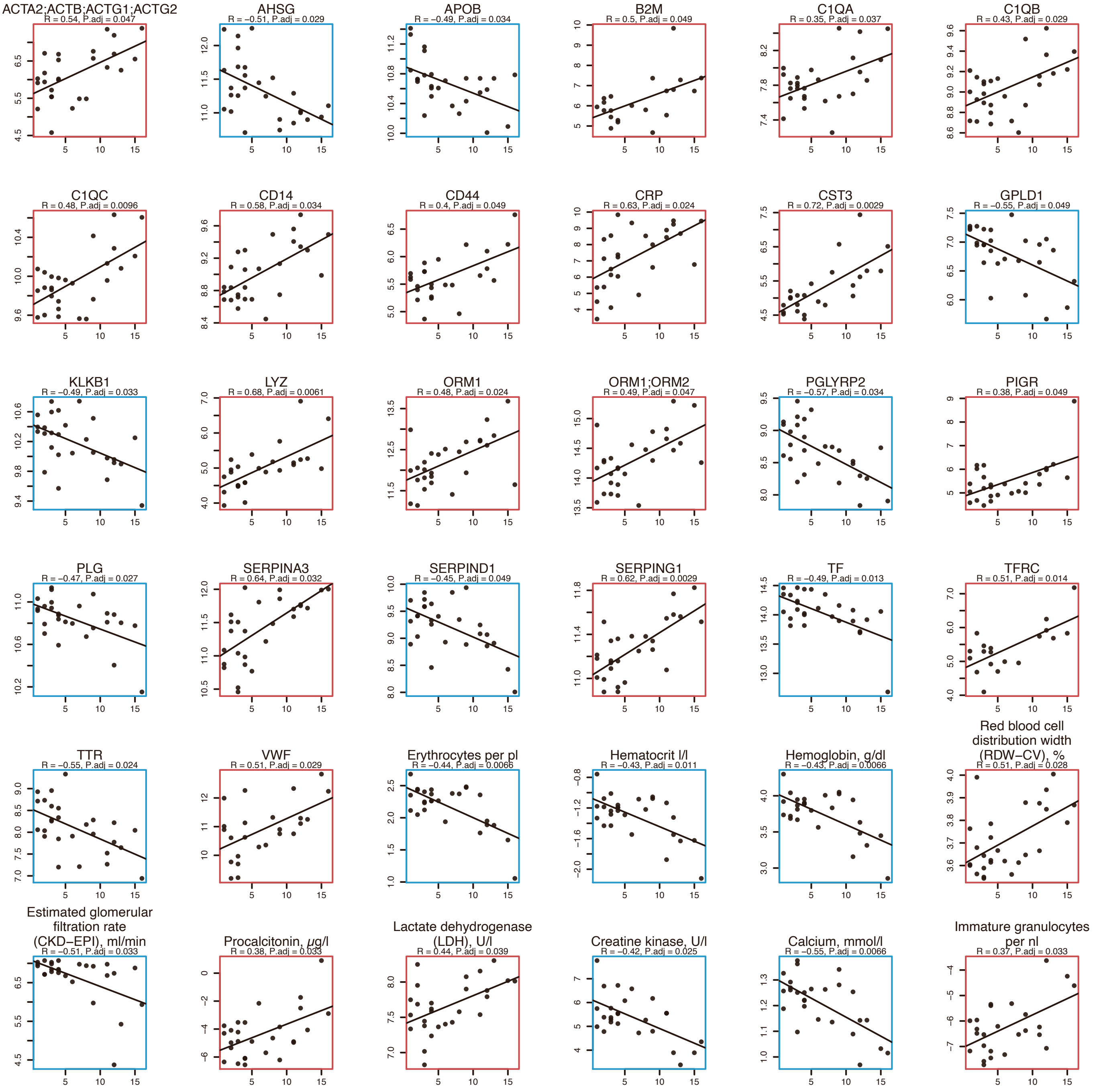

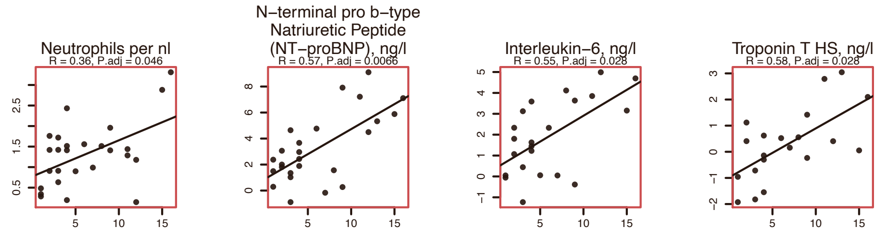

Neutrophils per nl
R = 0.36, P.adj = 0.046
N−terminal pro b−type Natriuretic Peptide (NT−proBNP), ng/l
R = 0.57, P.adj = 0.0066
Interleukin−6, ng/l
R = 0.55, P.adj = 0.028
Troponin T HS, ng/l
R = 0.58, P.adj = 0.028
