# Supplementary material for: A time-resolved proteomic and prognostic map of COVID-19
Source: Cell Syst. 2021 Aug 18;12(8):780–794.e7. doi: 10.1016/j.cels.2021.05.005 (PMC8201874; doi:10.1016/j.cels.2021.05.005)
Supplement: Data S1. Machine learning scripts, related to STAR methods [file mmc10.zip › Machine learning/Output/DaysUntilOutcome_WHO3_T_Proteome+Clinical.pdf]

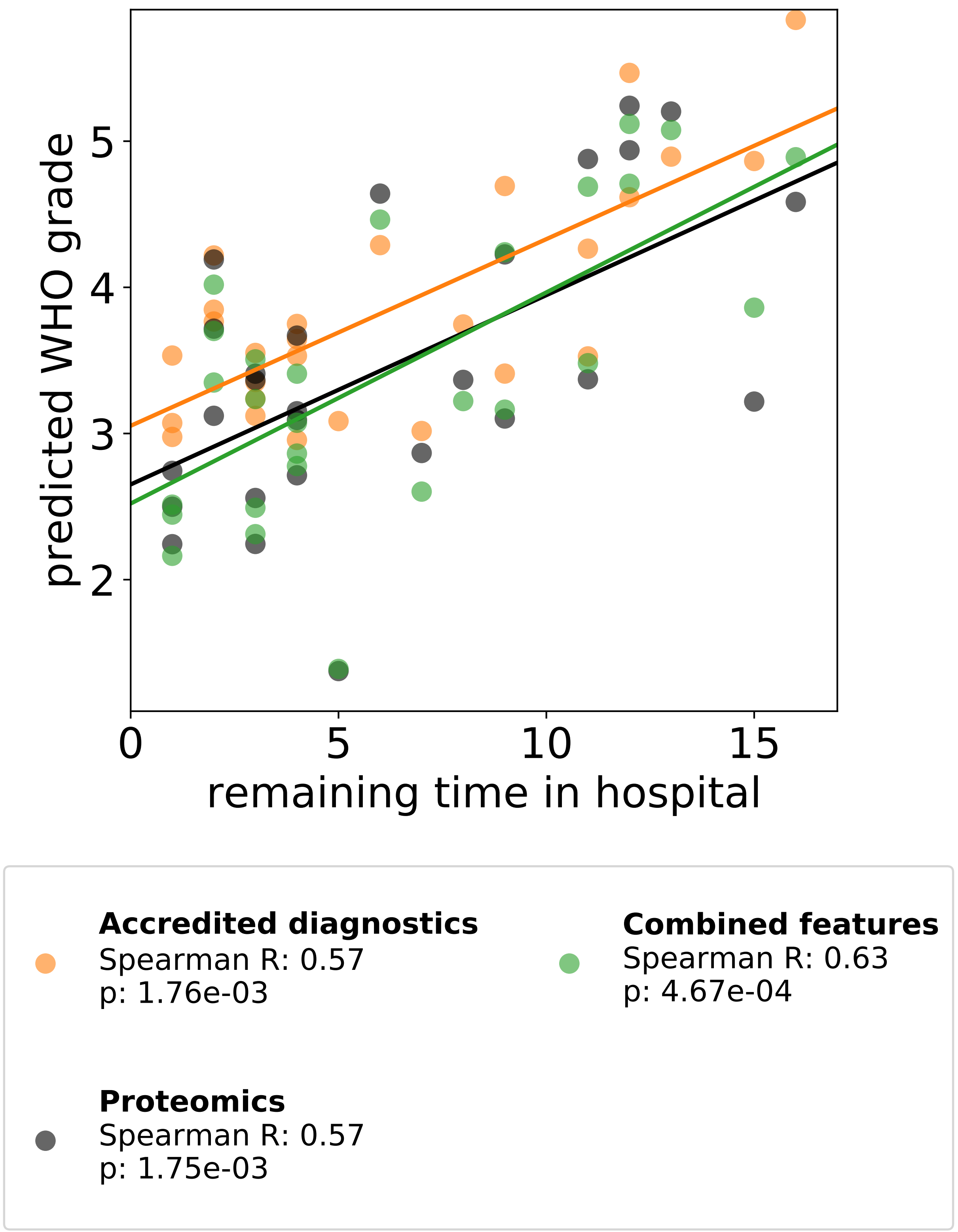

predicted WHO grade
2
3
4
5
0
5
10
15
remaining time in hospital
Accredited diagnostics
Spearman R: 0.57
p: 1.76e-03
Combined features
Spearman R: 0.63
p: 4.67e-04
Proteomics
Spearman R: 0.57
p: 1.75e-03
