# Supplementary material for: A time-resolved proteomic and prognostic map of COVID-19
Source: Cell Syst. 2021 Aug 18;12(8):780–794.e7. doi: 10.1016/j.cels.2021.05.005 (PMC8201874; doi:10.1016/j.cels.2021.05.005)
Supplement: Data S1. Machine learning scripts, related to STAR methods [file mmc10.zip › Machine learning/Output/WHO_prediction.pdf]

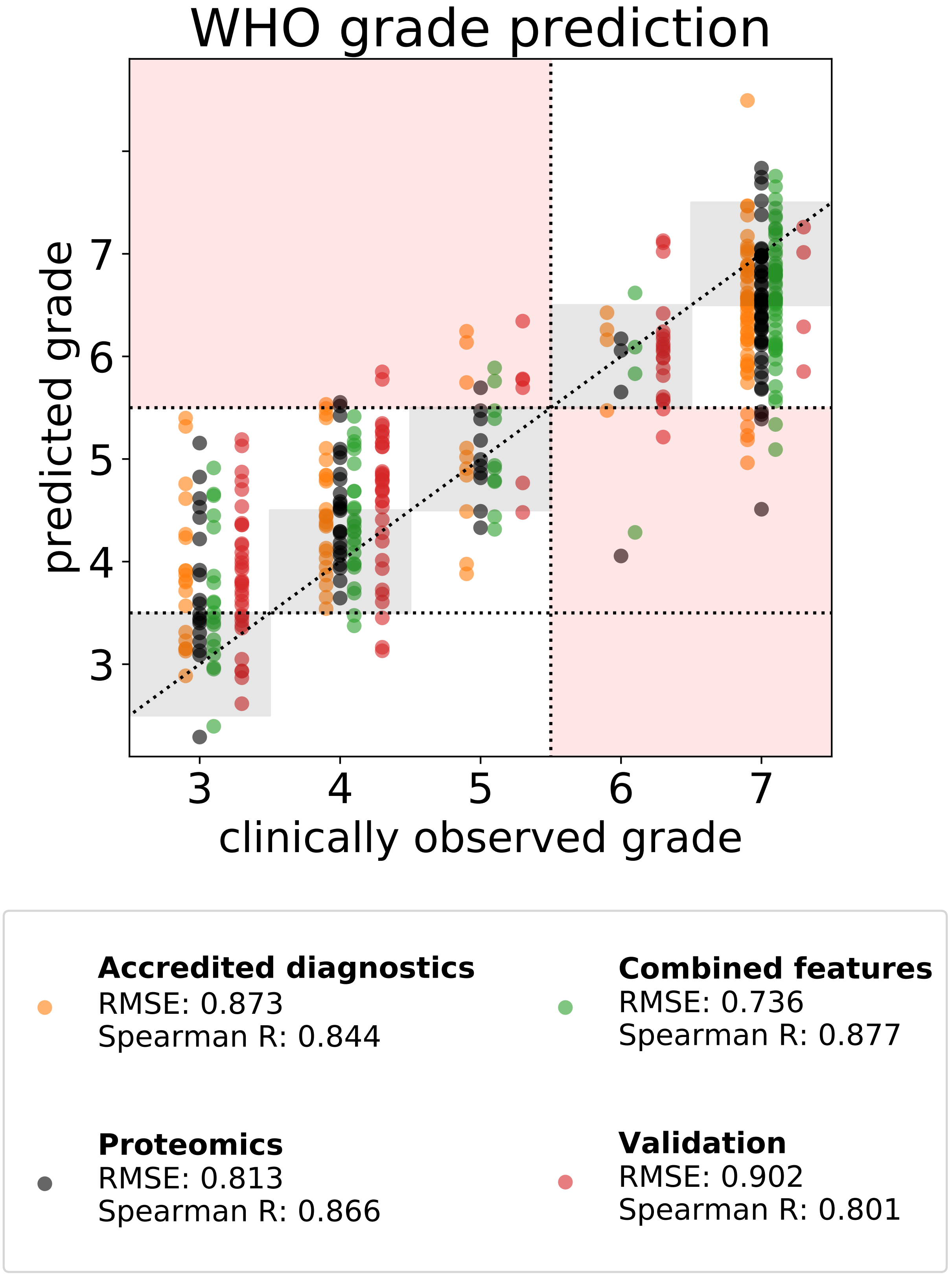
WHO grade prediction
predicted grade
3
4
5
6
7
clinically observed grade
Accredited diagnostics
RMSE: 0.873
Spearman R: 0.844
Combined features
RMSE: 0.736
Spearman R: 0.877
Proteomics
RMSE: 0.813
Spearman R: 0.866
Validation
RMSE: 0.902
Spearman R: 0.801
