# Supplementary figures and images for: A time-resolved proteomic and prognostic map of COVID-19
Source: Cell Syst. 2021 Aug 18;12(8):780–794.e7. doi: 10.1016/j.cels.2021.05.005 (PMC8201874; doi:10.1016/j.cels.2021.05.005)

# Proteomics

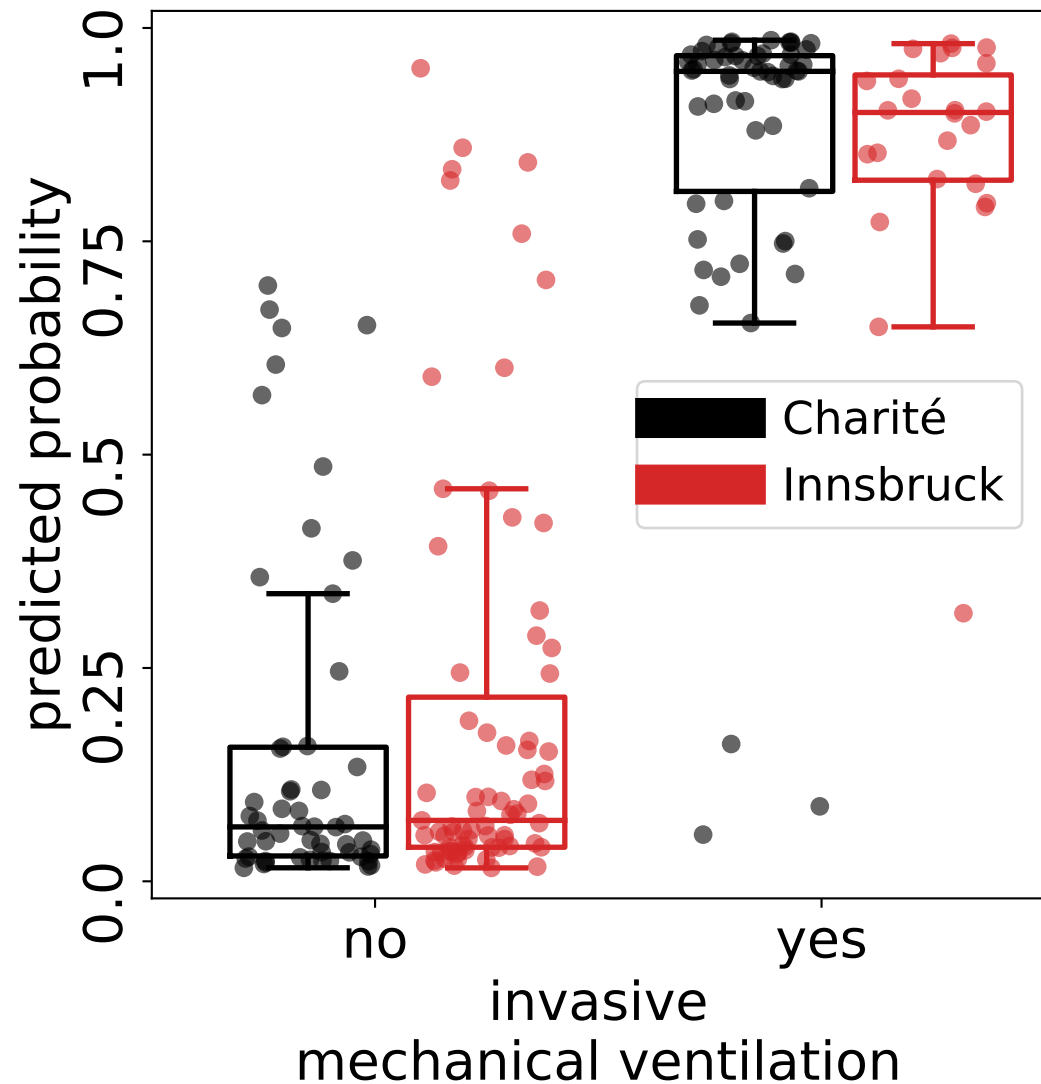

Supplement: Data S1. Machine learning scripts, related to STAR methods [file mmc10.zip › Machine learning/Output/Boxplot_Mechanical_Ventilation.pdf]

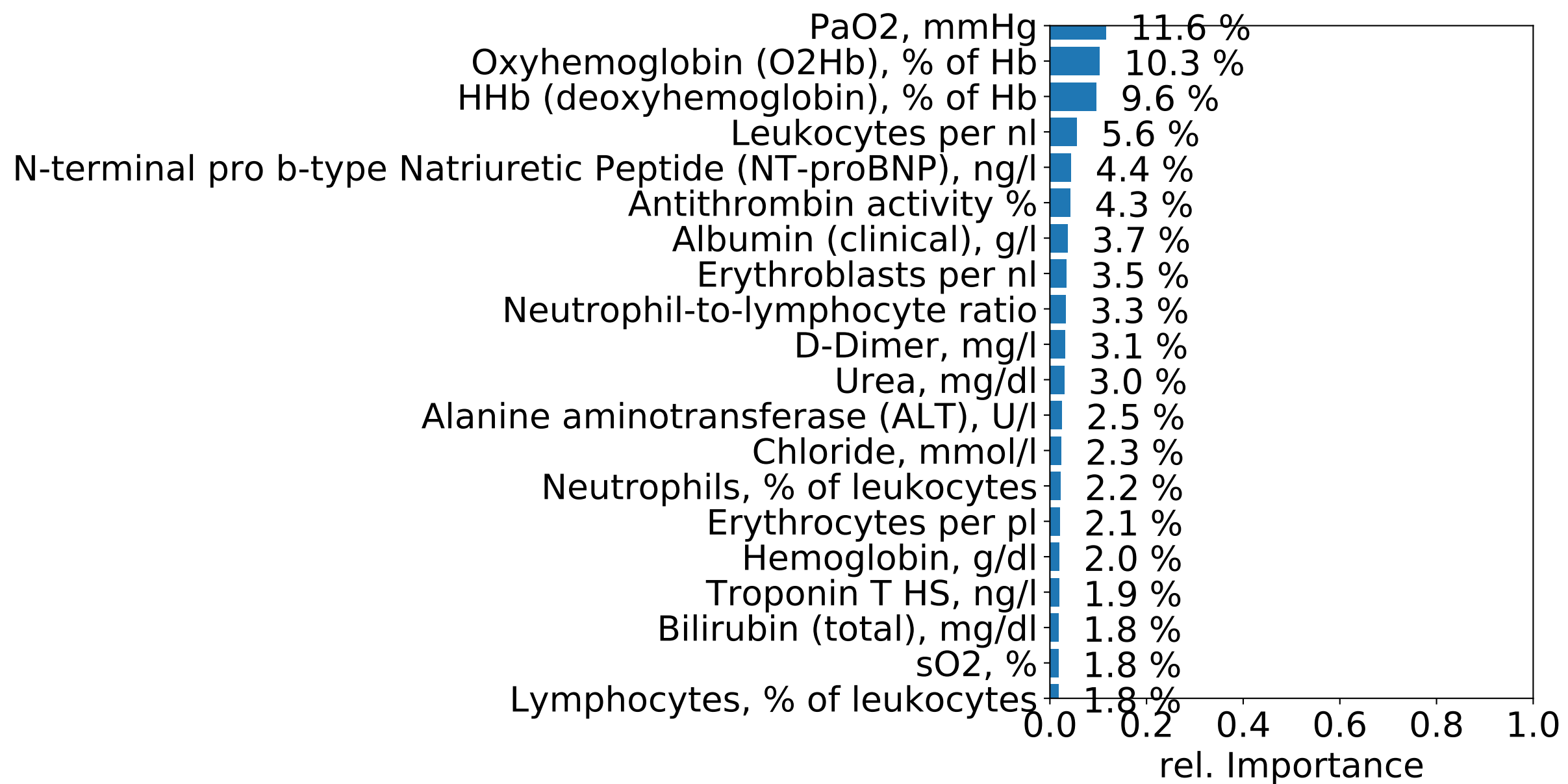

Supplement: Data S1. Machine learning scripts, related to STAR methods [file mmc10.zip › Machine learning/Output/Feature_Importance_Clinical.pdf]

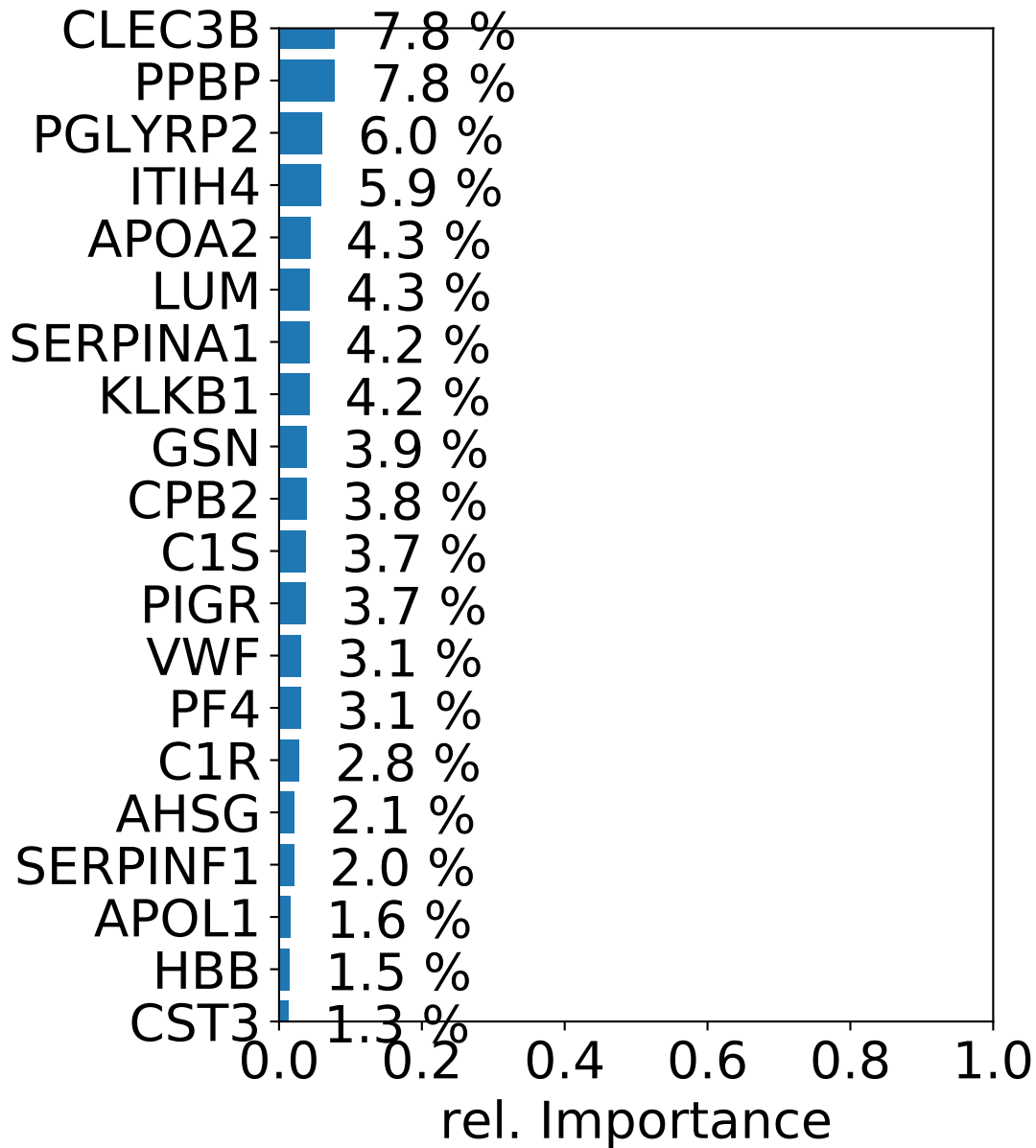

Supplement: Data S1. Machine learning scripts, related to STAR methods [file mmc10.zip › Machine learning/Output/Feature_Importance_Proteome.pdf]

# invasive mechanical ventilation

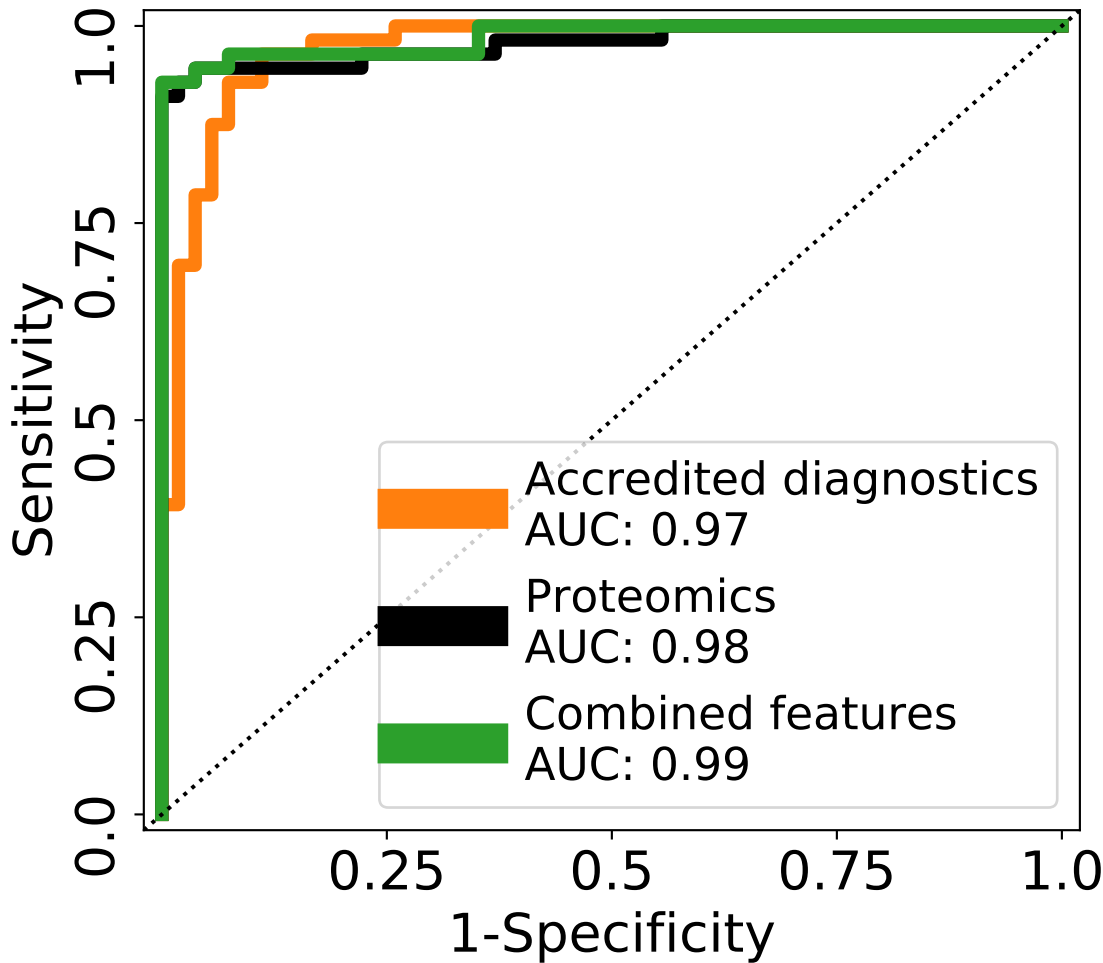

Supplement: Data S1. Machine learning scripts, related to STAR methods [file mmc10.zip › Machine learning/Output/ROC_Curve_Mechanical_Ventilation.pdf]

# invasive mechanical ventilation

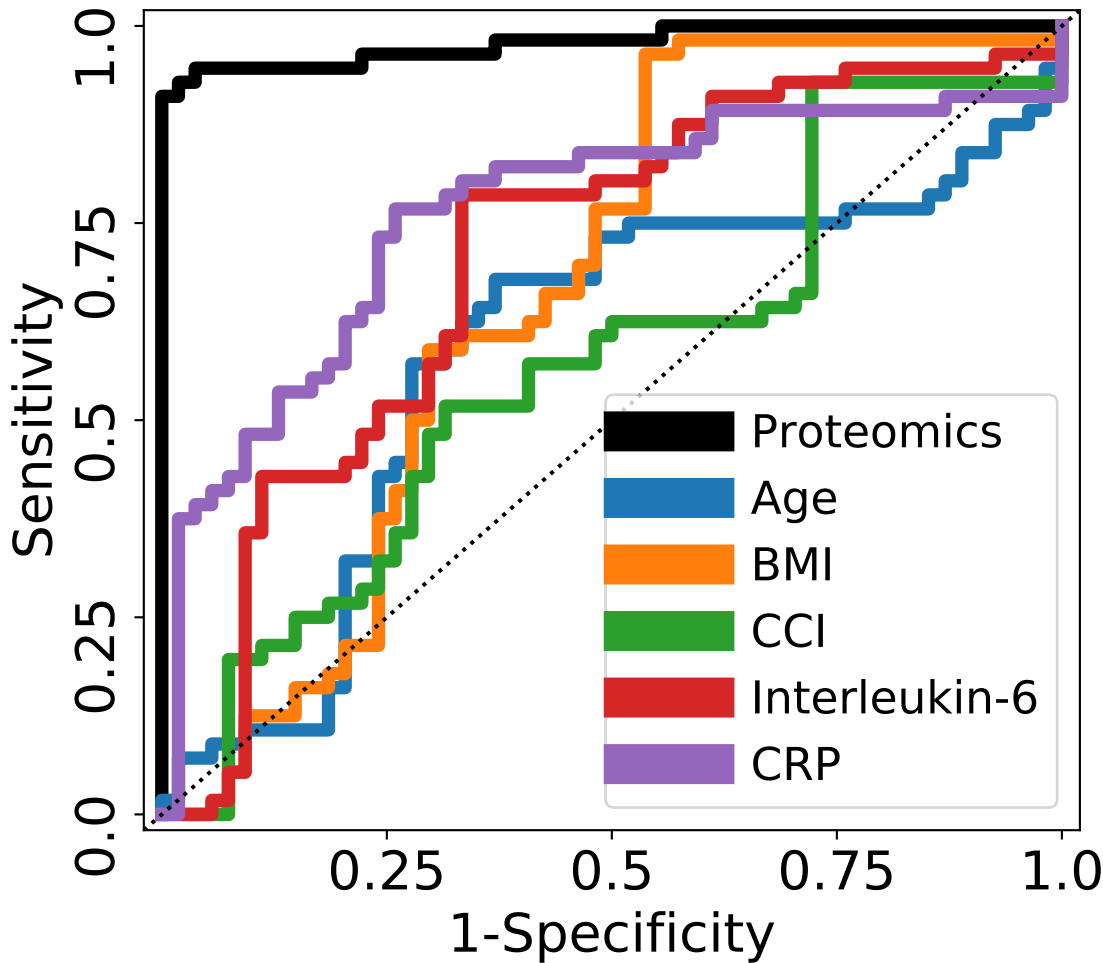

Supplement: Data S1. Machine learning scripts, related to STAR methods [file mmc10.zip › Machine learning/Output/ROC_Curve_Mechanical_Ventilation_single_predictor.pdf]

# validation

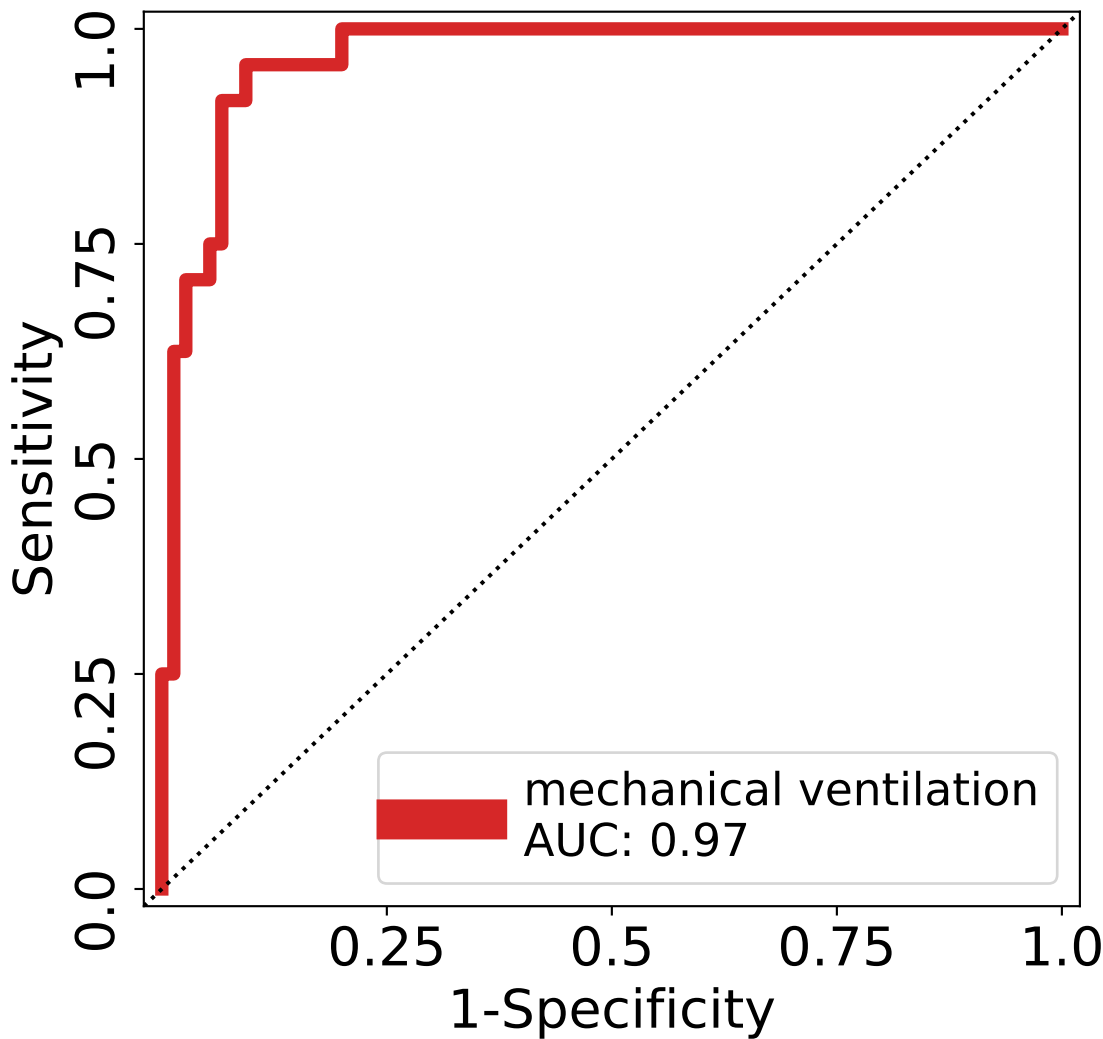

Supplement: Data S1. Machine learning scripts, related to STAR methods [file mmc10.zip › Machine learning/Output/ROC_Curve_Validation.pdf]
