# Supplementary material for: A time-resolved proteomic and prognostic map of COVID-19
Source: Cell Syst. 2021 Aug 18;12(8):780–794.e7. doi: 10.1016/j.cels.2021.05.005 (PMC8201874; doi:10.1016/j.cels.2021.05.005)
Supplement: Document S2. Article plus supplemental information [file mmc11.pdf]

Cell Systems

Article

A time-resolved proteomic and prognostic map of COVID-19

Graphical abstract

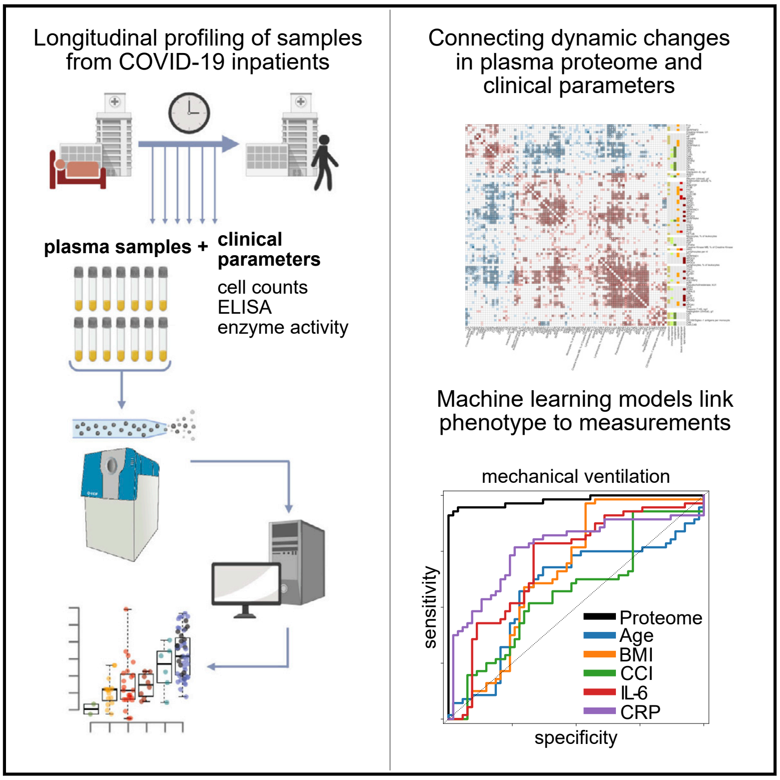

Highlights

- Plasma proteomes combined with clinical parameters characterize COVID-19 progression
- Machine learning models allow highly precise prediction of the disease phenotype
- The early molecular host response is predictive of COVID-19 progression
- The molecular response to COVID-19 is age specific

Authors

Vadim Demichev, Pinkus Tober-Lau, Oliver Lemke, ..., PA-COVID-19 Study group, Markus Ralser, Florian Kurth

Correspondence

markus.ralser@charite.de

In brief

Demichev, Tober-Lau et al., present a time-resolved molecular map of the COVID-19, measuring plasma proteomes of patients with COVID-19 along with an extensive panel of clinical diagnostic parameters at 687-time points. They describe the specificity and dynamics, as well as the predictive and prognostic power of the molecular signatures in COVID-19.

Demichev et al., 2021, Cell Systems *12*, 780–794
August 18, 2021 © 2021 The Authors. Published by Elsevier Inc.
https://doi.org/10.1016/j.cels.2021.05.005

CellPress

CellPress
OPEN ACCESS

Cell Systems

Article

A time-resolved proteomic and prognostic map of COVID-19

Vadim Demichev,[1,2,3,27] Pinkus Tober-Lau,[4,27] Oliver Lemke,[1] Tatiana Nazarenko,[8,11] Charlotte Thibeault,[4] Harry Whitwell,[9,10,26] Annika Röhl,[1] Anja Freiwald,[1] Lukasz Szyrwiel,[2] Daniela Ludwig,[1] Clara Correia-Melo,[2] Simran Kaur Aulakh,[2] Elisa T. Helbig,[4] Paula Stubbemann,[4] Lena J. Lippert,[4] Nana-Maria Grüning,[1] Oleg Blyuss,[10,12,13] Spyros Vernardis,[2] Matthew White,[2] Christoph B. Messner,[1,2] Michael Joannidis,[25] Thomas Sonnweber,[19] Sebastian J. Klein,[25] Alex Pizzini,[19] Yvonne Wohlfarter,[21] Sabina Sahanic,[19] Richard Hilbe,[19] Benedikt Schaefer,[20] Sonja Wagner,[20] Mirja Mittermaier,[4,22] Felix Machleidt,[4] Carmen Garcia,[4] Christoph Ruwwe-Glösenkamp,[4]

(Author list continued on next page)

[1]Charité Universitätsmedizin Berlin, Department of Biochemistry, 10117 Berlin, Germany
[2]The Francis Crick Institute, Molecular Biology of Metabolism Laboratory, London NW11AT, UK
[3]The University of Cambridge, Department of Biochemistry and Cambridge Centre for Proteomics, Cambridge CB21GA, UK
[4]Charité Universitätsmedizin Berlin, Department of Infectious Diseases and Respiratory Medicine, 10117 Berlin, Germany
[5]Charité Universitätsmedizin Berlin, Medical Department of Hematology, Oncology & Tumor Immunology, Virchow Campus & Molekulares Krebsforschungszentrum, 13353 Berlin, Germany
[6]Charité Universitätsmedizin Berlin, Department of Nephrology and Internal Intensive Care Medicine, 10117 Berlin, Germany
[7]Bernhard Nocht Institute for Tropical Medicine, Department of Tropical Medicine, and University Medical Center Hamburg-Eppendorf, Department of Medicine, 20359 Hamburg, Germany
[8]University College London, Department of Mathematics, London WC1E 6BT, UK
[9]National Phenome Centre and Imperial Clinical Phenotyping Centre, Department of Metabolism, Digestion and Reproduction, Imperial College London, London SW72AZ, UK
[10]Lobachevsky University, Department of Applied Mathematics, Nizhny Novgorod 603105, Russia
[11]University College London, Department of Women's Cancer, EGA Institute for Women'S Health, London WC1E 6BT, UK
[12]University of Hertfordshire, School of Physics, Astronomy and Mathematics, Hatfield AL10 9AB, UK
[13]Sechenov First Moscow State Medical University, Department of Paediatrics and Paediatric Infectious Diseases, Moscow 119435, Russia
[14]Lobachevsky University, Laboratory of Systems Medicine of Healthy Ageing, Nizhny Novgorod 603105, Russia
[15]Chalmers Tekniska Högskola, Department of Biology and Biological Engineering, SE-412 96 Gothenburg, Sweden
[16]University of Edinburgh, Centre for Genomic and Experimental Medicine, Institute of Genetics and Cancer, Edinburgh EH4 2XU, UK
[17]University of Edinburgh, Usher Institute, Edinburgh EH16 4UX, UK
[18]University of Edinburgh, MRC Human Genetics Unit, Institute of Genetics and Cancer, Edinburgh EH4 2XU, UK

(Affiliations continued on next page)

SUMMARY

COVID-19 is highly variable in its clinical presentation, ranging from asymptomatic infection to severe organ damage and death. We characterized the time-dependent progression of the disease in 139 COVID-19 inpatients by measuring 86 accredited diagnostic parameters, such as blood cell counts and enzyme activities, as well as untargeted plasma proteomes at 687 sampling points. We report an initial spike in a systemic inflammatory response, which is gradually alleviated and followed by a protein signature indicative of tissue repair, metabolic reconstitution, and immunomodulation. We identify prognostic marker signatures for devising risk-adapted treatment strategies and use machine learning to classify therapeutic needs. We show that the machine learning models based on the proteome are transferable to an independent cohort. Our study presents a map linking routinely used clinical diagnostic parameters to plasma proteomes and their dynamics in an infectious disease.

INTRODUCTION

The coronavirus disease 2019 (COVID-19) has created unprecedented societal challenges, particularly for public health and the global economy (Alwan et al., 2020; Blumenthal et al., 2020; Rosenbaum, 2020). Efficient management of these challenges is hampered by the variability of clinical manifestations, ranging from asymptomatic infection with severe acute respiratory syndrome coronavirus-2 (SARS-CoV-2) to death, despite maximum intensive care. Biomarkers and molecular signatures enabling accurate prognosis of future disease courses are needed to optimize resource allocation and personalize treatment strategies. Patients likely to progress to severe disease and organ failure and those likely to remain stable could be identified early, which

 © 2021 The Authors. Published by Elsevier Inc.
This is an open access article under the CC BY license (http://creativecommons.org/licenses/by/4.0/).

CellPress
OPEN ACCESS

Tilman Lingscheid,[4] Laure Bosquillon de Jarcy,[4] Miriam S. Stegemann,[4] Moritz Pfeiffer,[4] Linda Jürgens,[4] Sophy Denker,[5,22] Daniel Zickler,[6] Philipp Enghard,[6] Aleksej Zelezniak,[2,15] Archie Campbell,[16,17] Caroline Hayward,[18] David J. Porteous,[16,17] Riccardo E. Marioni,[16] Alexander Uhrig,[4] Holger Müller-Redetzky,[4] Heinz Zoller,[20] Judith Löffler-Ragg,[19] Markus A. Keller,[21] Ivan Tancevski,[19] John F. Timms,[11] Alexey Zaikin,[8,11,14] Stefan Hippenstiel,[4,24] Michael Ramharter,[7] Martin Witzenrath,[4,24] Norbert Suttorp,[4,24] Kathryn Lilley,[3] Michael Mülleder,[23] Leif Erik Sander,[4,24] PA-COVID-19 Study group, Markus Ralser,[1,2,28,*] and Florian Kurth[4,7]

[19]Medical University of Innsbruck, Department of Internal Medicine II, 6020 Innsbruck, Austria
[20]Medical University of Innsbruck, Christian Doppler Laboratory for Iron and Phosphate Biology, Department of Internal Medicine I, 6020 Innsbruck, Austria
[21]Medical University of Innsbruck, Institute of Human Genetics, 6020 Innsbruck, Austria
[22]Berlin Institute of Health, 10178 Berlin, Germany
[23]Charité – Universitätsmedizin Berlin, Core Facility - High-Throughput Mass Spectrometry, 10117 Berlin, Germany
[24]German Centre for Lung Research, 35392 Gießen, Germany
[25]Medical University Innsbruck, Division of Intensive Care and Emergency Medicine, Department of Internal Medicine, 6020 Innsbruck, Austria
[26]Imperial College London, Section of Bioanalytical Chemistry, Division of Systems Medicine, Department of Metabolism, Digestion and Reproduction, London SW7 2AZ, UK
[27]These authors contributed equally
[28]Lead contact
*Correspondence: markus.ralser@charite.de
https://doi.org/10.1016/j.cels.2021.05.005

is particularly valuable in scenarios where health care systems reach capacity limits. Prognostic panels would also optimize the monitoring of novel treatments, thereby accelerating clinical trials (Phua et al., 2020; Saxena, 2020; Wu et al., 2020). Knowledge of factors that differentiate recovery from deterioration throughout the disease will further enhance our understanding of the inflammatory host response as well as the underlying pathophysiology and provide new therapeutic targets.

A number of biomarkers that classify COVID-19 severity have recently been described. These are based on clinical chemistry, enzyme activities, immune profiling, single-cell sequencing, proteomics, and metabolomics (D'Alessandro et al., 2020; Laing et al., 2020; Liu et al., 2020b; Messner et al., 2020; Overmyer et al., 2020; Schulte-Schrepping et al., 2020; Shen et al., 2020; Shu et al., 2020; Wynants et al., 2020). As severity classifiers, the molecular signatures recorded in blood, serum, plasma, or immune cells characterize the COVID-19 pathology and host responses. Furthermore, markers of dysregulated coagulation, inflammation, and other organ dysfunction have been established as risk factors for severe illness, including low platelet count, elevated levels of D-dimer, C-reactive protein (CRP), interleukin 6 (IL-6), ferritin, troponin, and markers of kidney injury (Danwang et al., 2020; Henry et al., 2020). Proteomic investigations that characterize the comprehensive host response have revealed the activation of the complement cascade and acute phase response, both of which center around IL-6-driven pathways. In turn, these systematic studies have revealed that other common antiviral pathways, such as type I interferons (IFN), do not dominate the early response to COVID-19, probably reflecting evasion of the IFN system by SARS-CoV-2 and the subsequent activation of inflammatory cascades (Hadjadj et al., 2020; Yang et al., 2020). Furthermore, proteomic data and diagnostic parameters have pointed to underlying pathological mechanisms and possible therapeutic targets. For instance, using high-throughput proteomics, we reported a decline in plasma levels of gelsolin (GSN) in patients with severe COVID-19 in a previous study (Messner et al., 2020), and recombinant human GSN is currently undergoing clinical testing for COVID-19 pneumonia in a phase II trial (ClinicalTrials.gov identifier: NCT04358406).

The severity of the disease, and the biomarker signatures that indicate severity, correlate with the outcome, but the highest diagnostic need is to stratify within therapeutically homogeneous patients. For instance, to identify those among the mildly affected individuals with the highest risk for deterioration, or among the most severely affected, those with the highest chance to respond positively to an augmentation of therapy. Predicting future trajectories on an individualized basis would also help accelerate therapeutic developments to judge the impact of the treatment on an individual disease course. To obtain a comprehensive picture of how the molecular COVID-19 phenotype develops over time, we deeply phenotyped a group of 139 COVID-19 inpatients at 687 sampling points. On the one hand, we measured a compendium of 86 clinical parameters, routine diagnostic markers, and clinically established risk scores using gold standard accredited clinical tests. On the other hand, we captured the patient's molecular phenotype by measuring plasma proteomes in an untargeted fashion. For this, we made use of liquid chromatography coupled with tandem mass spectrometry, using a recently developed platform technology that utilizes analytical flow rate chromatography, data-independent acquisition mass spectrometry (SWATH-MS), and deep-neural network-based data processing (Demichev et al., 2020; Messner et al., 2020) (Figure S1). By combining the compendium of diagnostic parameters with the proteomes in a time- and patient-resolved fashion, we obtained a comprehensive molecular picture that captures changes in the patient's molecular phenotype as they depend on the severity, age, and disease progression. We identify prognostic biomarkers and depict their distinct trajectories. We exemplify the power of our resource by showing that the biomarker profiles and diagnostic parameters classify treatment requirements, in particular, the need for mechanical ventilation. Furthermore, we report the future prediction of recovery time in mildly ill patients as well as the individual risk of

clinical deterioration. Our study demonstrates the predictability of COVID-19 disease trajectories based on the molecular phenotype of the early disease stage.

RESULTS

Covariation of clinical diagnostic parameters and the plasma proteome characterizes the host response to COVID-19

We longitudinally phenotyped 139 patients admitted to Charité University Hospital, Berlin, Germany, between March 01, 2020, and June 30, 2020, due to PCR-confirmed SARS-CoV-2 infection (Figure S2). The patients exhibited highly variable disease courses, graded according to the World Health Organization (WHO) ordinal scale for clinical improvement (Table S1), which reflects the treatment that the patient is receiving as a measure of disease severity. The patients included in our study range from WHO grade 3, which includes patients who require inpatient care without supplemental oxygen therapy, to WHO grade 7, which includes patients with severe COVID-19 who require invasive mechanical ventilation and additional organ support therapies such as renal replacement therapy (RRT) and extracorporeal membrane oxygenation (ECMO) (WHO, 2020). In total, 23 out of 139 (17%) patients in the WHO grade 3 category were stable, without requiring supplemental oxygen therapy and could be discharged after a median of 7 days of inpatient care (Table S2 and Figure S2); 47 (34%) patients required either low-flow or high-flow supplemental oxygen therapy; 69 (50%) patients either presented with severe COVID-19 (WHO grade 6 or 7, i.e., requiring invasive mechanical ventilation) or deteriorated and required invasive mechanical ventilation during their hospitalization; 46 patients (33%) required RRT; and 22 (16%) were treated with ECMO. A total of 20 (13%) patients died, including three patients with *do not intubate/do not resuscitate (DNI/DNR)* orders in place and one patient who died due to a non-COVID-19-related cause. Common risk factors for severe COVID-19 were reflected in the outcomes: patients with a severe course of disease were older than those with mild disease (49 years [IQR 35–70] for WHO grade 3 versus 62 years [IQR 53–72] for WHO grade 7, p = 0.02), and an age of 65 years or older was associated with a higher risk of death -(OR 4.1 [95% CI 1.5–11.5]). Our cohort further reflected that men and individuals with a high BMI have an increased likelihood to be hospitalized upon a COVID-19 infection; 68% of the patients were men, and the median BMI was 27.8 (IQR 24.7–31.9). However, we noted that within the group of patients hospitalized with COVID-19, sex and BMI were not further associated with disease severity or an increased risk of death. The median duration of hospitalization was 20 days (IQR 9–48) and correlated with severity (7 days for WHO grade 3 versus 46 days for WHO grade 7). The median time from admission to death despite receiving maximum treatment was 28 days (IQR 16–46).

To capture the diverse disease trajectories on a molecular and biochemical level, we systematically collected 86 clinical and accredited diagnostic parameters as measured with certified tests. Moreover, we monitored the development of risk scores such as the "sequential organ failure assessment" (SOFA) score, blood gas analyses, blood cell counts, enzyme activities, and inflammation biomarkers (Table S3). To complement these parameters with an untargeted analysis, we employed a recently developed high-throughput proteomics platform (Messner et al., 2020). This platform makes use of the data-independent acquisition technique SWATH-MS (Gillet et al., 2012), a sample preparation pipeline designed to ISO13485 reporting standards, which is optimized for reducing batch effects, high-flow rate chromatography to provide highly consistent peptide separation in large sample series, and uses DIA-NN (Data-Independent Acquisition by Neural Networks) to analyze proteomics data recorded with 5-min chromatography (Messner et al., 2020; Demichev et al., 2020) (Figure S1 for a detailed overview of the proteomic workflow). In total, we measured 1,169 plasma proteome samples to determine 687 human proteomes, in which we quantified 321 plasma protein groups. Owing to the nature of the high-flow proteomics platform, data completeness was high; thus, we decided against the use of imputation strategies in the analysis of differential protein abundance. Total data completeness was 75%, with 200 proteins consistently quantified with 98% completeness, and 189 proteins with 99% completeness (Figure S1).

To identify interdependencies of the diagnostic parameters that are routinely used in clinical decision making and the plasma proteomes, we characterized their covariation and present a direct correlation map (Figures 1B and S3–S4; Tables S4, S5, and S6). We report a robust positive or negative correlation of IL-6 levels and other inflammatory markers (CRP, procalcitonin) with acute phase proteins (APPs) (APOA2, APOE, CD14, CRP, GSN, ITIH3, ITIH4, LYZ, SAA1, SAA2, SERPINA1, SERPINA3, and AHSG; the protein names corresponding to the gene identifiers are provided in Table S3), coagulation factors and related proteins (FGA, FGB, FGG, F2, F12, KLKB1, PLG, and SERPINC1), and the complement system (C1R, C1S, C8A, C9, CFB, CFD, and CFHR5). Our data, therefore, link the prominent role of the IL-6 response in COVID-19 (D'Alessandro et al., 2020) to coagulation and the complement cascade. Consistently, in our data, markers of cardiac (troponin T, NT-proBNP) and renal (creatinine, urea) function, as well as anemia and dyserythropoiesis (hemoglobin, hematocrit, erythrocytes, and red blood cell distribution width) correlate with various APPs (APOA2, APOE, CD14, GSN, LYZ, SAA1, SAA2, and SERPINA3; Figure 1B and Table S4) supporting the role of inflammation in COVID-19-related organ damage and its impact on erythropoiesis.

Increased levels of neutrophils and the occurrence of immature granulocyte precursors as markers of emergency myelopoiesis have been linked to severe COVID-19 (Schulte-Schrepping et al., 2020). Our data reveal covariation between neutrophil counts and the levels of two inhibitors of neutrophil serine proteases, SERPINA1 and SERPINA3 (Figure 1C). These two proteins show the highest correlation (0.72 and 0.79 Spearman R, respectively) with the neutrophil-to-lymphocyte ratio (NLR), a prognostic marker for COVID-19 (Lian et al., 2020; Liu et al., 2020a). We further report a strong correlation (Figure 1C) of alkaline phosphatase and gamma-glutamyl transferase activities, both characteristic of biliary disorders (Poynard and Imbert-Bismut, 2012), with plasma levels of the polymeric immunoglobulin receptor (PIGR). We notice that cholangiocytes (bile duct epithelium cells) express ACE-2 and can be directly infected with SARS-CoV-2 (Zhao et al., 2020), potentially leading to host viral

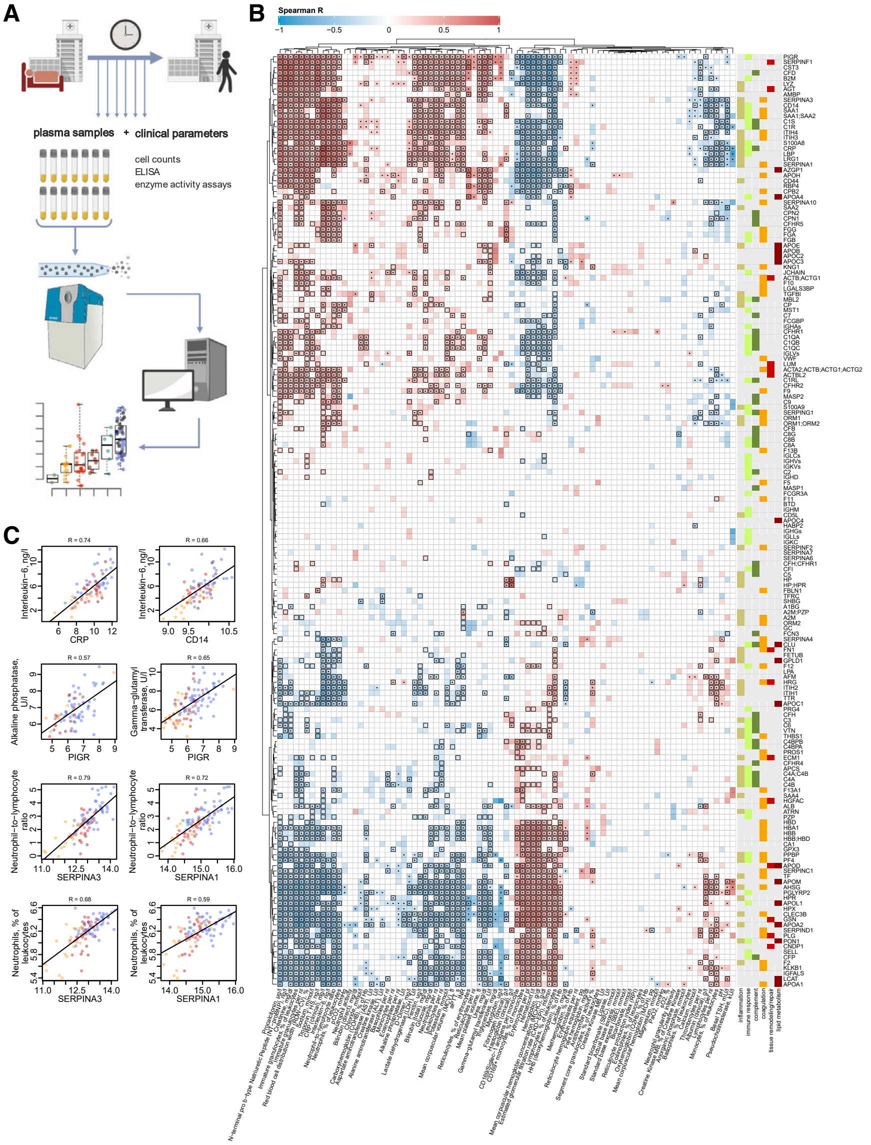

Figure 1. Interdependence of clinical, diagnostic, physiological and proteomic parameters during the clinical progression of COVID-19

(A) Study design. Schematic of the cohort of 139 patients with PCR-confirmed SARS-CoV-2 infection treated at Charité University Hospital Berlin. Plasma proteomics and accredited diagnostic tests were applied at 687 sampling points to generate high-resolution time series data for 86 routine diagnostic parameters and 321 protein quantities (see also Figures S1 and S2).

(B) Covariation map for plasma proteins and routine diagnostic and physiological parameters. Statistically significant correlations (Spearman; $p < 0.05$) are colored. Dots indicate statistical significance after row-wise multiple-testing correction (false discovery rate [FDR] < 0.05), black rectangles—column-wise. The panel on the right of the heatmap provides manual functional annotation for the proteins (see also Figures S3 and S4, and Tables S4, S5, and S6).

(C) Covariation of key diagnostic parameters and plasma protein markers (log_2-transformed) in COVID-19 (see also Tables S4, S5, and S6). Dots colors correspond to the WHO grade of the patient, see Figure 2B.

response-induced expression of PIGR and cell destruction (Schneeman et al., 2005; Turula and Wobus, 2018).

A map of plasma proteins and diagnostic parameters that depend on age and disease severity

113 proteins and 55 accredited diagnostic parameters responded in accordance to an increase in the WHO score as a measure of progressing COVID-19 severity (Figures 2, S5, and S6; STAR methods). To the best of our knowledge, more than 30 of these proteins have not been associated with COVID-19 severity previously (Table S3). The proteins that change dependent on disease severity include mediators of inflammation and immune response (CD44, B2M, PIGR, and A2M), components of the complement cascade (CFD, and CFHRs), and apolipoproteins (APOA2, APOC3, APOD, APOE, and APOL1). Furthermore, numerous markers of organ dysfunction (cardiac: NT-proBNP, troponin T; renal: creatinine, urea; liver: aspartate aminotransferase, alanine aminotransferase, gamma-glutamyl transferase, and total bilirubin) and, inversely, markers of anemia (hemoglobin, erythrocytes, and hematocrit) were correlated with the WHO grade of the patient. In order to further dissect the proteomic signatures of the most severely ill patients requiring maximum treatment (WHO grade 7), we specifically characterized the impact of organ support treatments (RRT and ECMO) on the patients' molecular phenotype (Figures S7 and S8). We showed, for instance, that HP and HPX are reduced in patients on RRT and ECMO as a sign of hemolysis in the extracorporeal circuit, whereas elevated SERPINC1 levels mirror substitution of antithrombin during ECMO. We discuss these findings in Note S1.

A total of 61 proteins and 18 diagnostic parameters varied with patients' age (Figure S9). Out of these, 37 proteins do not change with age in a pre-COVID-19 general population baseline (Generation Scotland cohort [Smith et al., 2006]), for which proteomes have been measured with the same proteomic technology (Messner et al., 2020) (Figure S10). We observed that a number of markers that increase with age in COVID-19 patients also correlated with a high WHO grade (Figures S6 and S9). To identify markers that are upregulated or downregulated in older patients in comparison with younger patients with a comparable therapy need, i.e. WHO grade, we tested the relationship between omics feature levels and age by accounting for WHO severity grade as a covariate using linear modeling

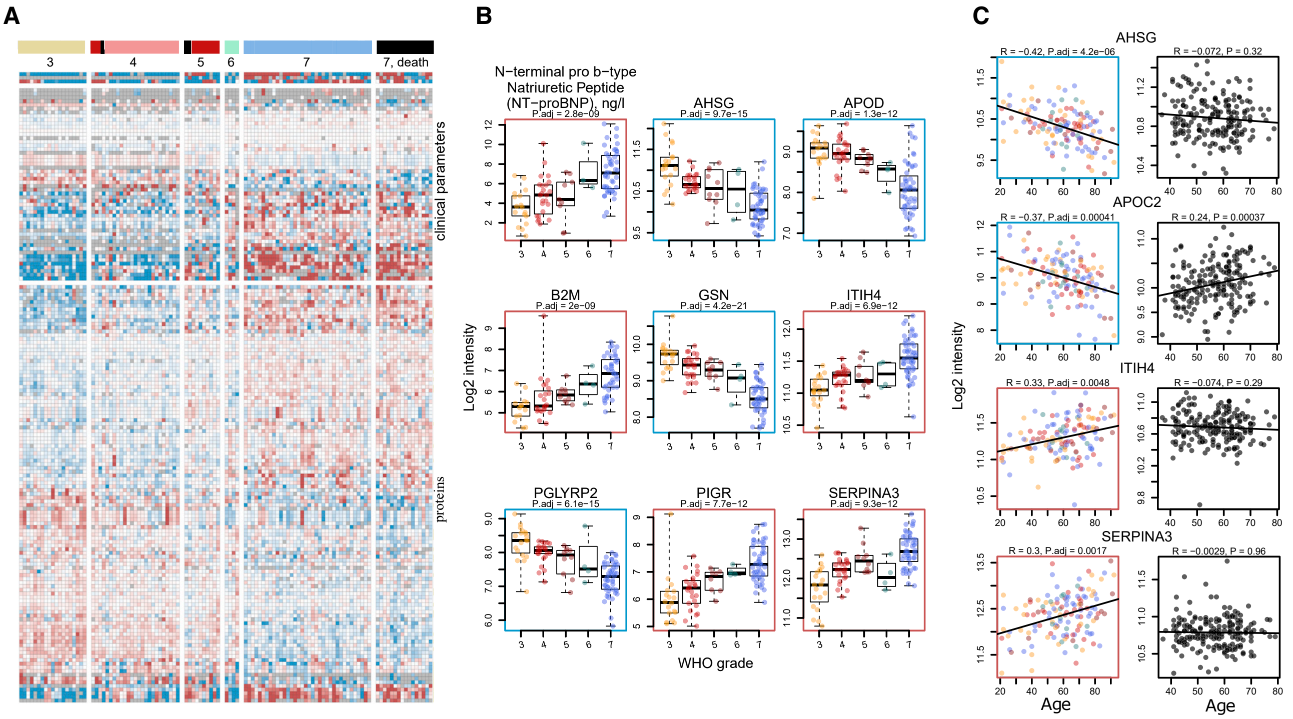

Figure 2. The molecular phenotype of patients with COVID-19 and its dependency on severity and age

(A) Plasma proteome and clinical diagnostic parameters in dependency of COVID-19 severity irrespective of age. The patients are grouped according to the maximum clinical treatment received (WHO ordinal scale), used as an indicator of disease severity (Table S1). 113 proteins and 55 routine diagnostic parameters vary significantly (FDR < 0.05) between patients of the different WHO groups upon accounting for age as a covariate using linear modeling (Ritchie et al., 2015). A fully annotated heatmap is provided in Figure S5 (see also Figure S6 and Table S3).

(B) Selected protein markers and routine diagnostic parameters (log_2-transformed) plotted against the WHO ordinal scale.

(C) Selected proteins differentially abundant depending on age (FDR < 0.05). Left, colored: this data set (log_2-transformed levels; statistical testing was performed by accounting for the WHO grade as a covariate Ritchie et al., 2015 and STAR methods; for visualization only, the data were corrected for the WHO grade); right, black: general population (log_2-transformed levels; Generation Scotland cohort).

(Ritchie et al., 2015) (STAR methods). This analysis identified 36 proteins and 12 clinical laboratory markers that are up- or are downregulated with age in COVID-19 patients within the same level of care, i.e., one WHO grade (Figures 2C and S11, summarized in Figure 5). Out of these, 20 proteins do not change with age in the pre-COVID-19 population baseline (Generation Scotland cohort proteome data, Messner et al., 2020; Figure S10), or show the opposite correlation with age in the general population (e.g., APOC2, Figure 2C). These proteins that only show an age-dependency in COVID-19 patients but not in the general population point toward age-dependent differences in host response patterns to SARS-CoV-2, and include markers involved in inflammation (SERPINA3, ITIH4, SAA1, SAA1, SAA2, ITIH3, CFB, C7, and AHSG), lipid metabolism (APOC1, APOC2, APOC3, APOB, and APOD), and coagulation (KLKB1, and FBLN1). We consider the implications of these findings in Note S2.

Time-dependent alleviation of severity indicators highlights the role of the early host response in COVID-19 progression

The time-resolved nature of our study facilitated a covariation analysis of protein levels and accredited diagnostic parameters along the patient trajectory over time (Figure S12; Table S7). Correlating the dynamics of omics features during the peak period of the disease (STAR methods), we noted covariation of inflammatory markers, APPs, fibrinogen precursor proteins, and the NLR. The correlation between APPs and the markers of cardiac and renal impairment observed across different patients at the earliest time points (Figure 1B; Table S4) was not reflected as a trend over time (Figure S13).

To further dissect the dynamics of the patients' molecular phenotype during the course of COVID-19, we determined the longitudinal trend for all protein and diagnostic parameters during the peak period of the disease (i.e., while receiving maximum treatment; STAR methods). In total, 89 proteins and 37 clinical parameters significantly changed over time (Figure 3B, trends across all time points at the maximum WHO grade are provided in Figure S14; STAR methods). In general, we found that most proteins and diagnostic parameters that correlate with disease severity return toward baseline during the peak period of the disease. Many of these were most prominently changed in the early samples (Figure S6) but alleviated with time, irrespective of the outcome (Figure S14; summarized in Figure 5). For example, components of the coagulation cascade with known acute phase activity, such as fibrinogen, and many complement factors, significantly decreased over time. Proteins indicative of inflammatory response (e.g., ORM1, SERPINA1 and SERPINA3, SAA1, SAA2 [Luo et al., 2015; Sack, 2018; Wu et al., 2015]) and markers of inflammation,

CellPress
OPEN ACCESS

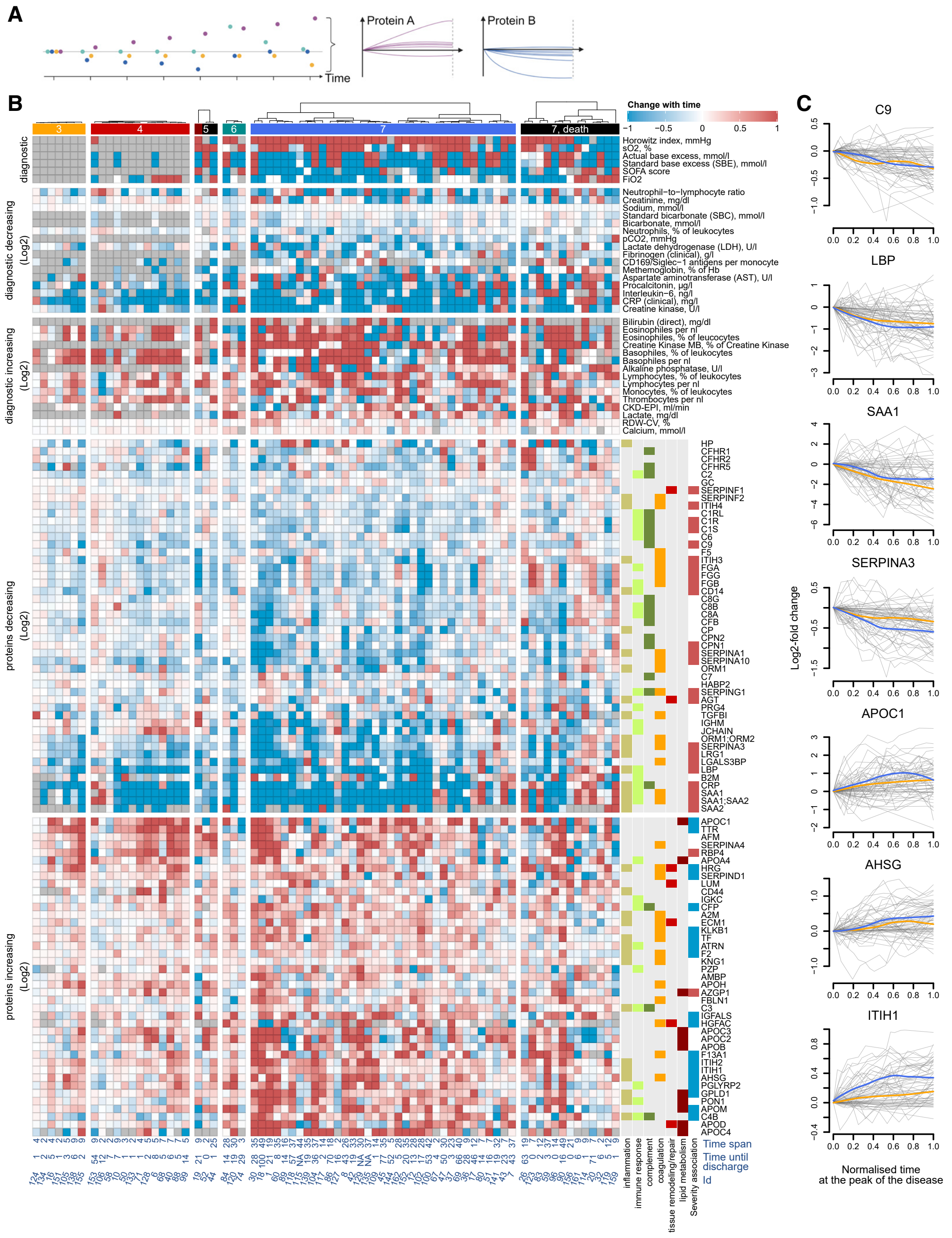

Figure 3. The progression of the COVID-19 molecular patient phenotype over time

(A) Schematic: each patient is followed during inpatient care by repetitive sampling, and the "trajectory" of each of the proteins and the routine diagnostic features is analyzed (points of different colors at each time point) (see also Figure S2).

(B) Protein levels and routine diagnostic parameters that change significantly (FDR < 0.05) over time during the peak of the disease, shown for individual patients stratified by their maximum treatment received (WHO grade): 89 proteins, 37 clinical diagnostic markers show time dependency during the disease course (illustrated as $\log_2$-fold changes or absolute value changes, as indicated). The panel to the right of the heatmap provides manual functional annotation for the proteins. Known associations with COVID-19 severity are indicated (blue - downregulated in severe COVID-19, and red - upregulated) (D'Alessandro et al., 2020; Laing et al., 2020; Messner et al., 2020; Shen et al., 2020). Below the heatmap, the time span between the first and the last sampling time point at the peak of the disease is indicated as well as the remaining time until the discharge (see also Figures S14 and S15, and Table S3).

(C) Trajectories (change of $\log_2$-transformed levels with time) for selected proteins. Sampling points during the peak period of the disease (STAR methods) are considered. x axis: 0 – first time point measured at the peak of the disease, 1 – last. The y axis reflects the change relative to the first valid measurement during the peak of the disease. Loess approximations are shown for patients, which did (blue), and did not (orange), require invasive mechanical ventilation. See also Figure S16.

such as CRP or IL-6, also declined over time. Conversely, extracellular matrix (ECM) proteins, such as ECM1, LUM, and immunoregulatory factors (e.g. AHSG, A2M Rehman et al., 2013, and HRG Wakabayashi, 2013) and proteins involved in lipid metabolism (e.g., APOC1, APOD, APOM, GPLD1, and PON1), and negative APPs (e.g., ITIH1, Figure 3C), which are downregulated in severe COVID-19 (Figure S6, summarized in Figure 5), increased over time, approaching the baseline. This general alleviation of the initial molecular phenotype of COVID-19 was consistently detected in both mildly and severely ill patients (outlier trajectories discussed in Note S3). Indeed, only 13 proteins showed differences in trend depending on the WHO score (Figure S15). We provide visualization of individual trajectories for all omics features measured between the first and the last time points sampled at the peak of the disease (Figures 3C and S16).

Overall, the molecular patient phenotype reflected an initial spike in the systemic inflammatory response, which alleviated gradually, followed by a protein signature indicative of tissue repair, metabolic reconstitution, and immunomodulation. This was observed in both mildly and severely ill patients, highlighting the early disease phase as a major molecular determinant of the COVID-19 phenotype.

Proteomes and diagnostic clinical markers allow for prediction of disease severity by machine learning

Using a machine learning algorithm based on gradient boosted trees (STAR methods), we first evaluated the extent to which diagnostic parameters and proteomes characterize treatment requirements, as reflected by the WHO grade. Both proteomes and clinical diagnostic parameters were highly discriminative of the patient receiving invasive mechanical ventilation (WHO grade 6 or 7, clinical laboratory values AUROC = 0.97, proteomic data AUROC = 0.98, combined data AUROC = 0.99; Figure 4C). The machine learning models significantly outperformed the predictive scores derived from established COVID-19 risk factors such as age, BMI, Charlson comorbidity index (CCI), or molecular predictors such as CRP or IL-6 levels (Figure 4C). In order to assess the transferability of the proteomic predictors, we tested our model in an independent cohort of 99 hospitalized patients with COVID-19 from another hospital in a different healthcare system (Innsbruck cohort, STAR methods). The proteomic model trained on the main Charité cohort demonstrated a comparably high patient stratification performance when applied to this validation cohort (Figure 4D; AUROC = 0.97). Scores reflecting the contribution of individual proteins and clinical parameters to the machine learning model are provided in Table S3. Of note, we were able to establish machine learning models that not merely classified patients based on severity but were able to predict the current WHO severity grade from the proteome, from clinical measurements, and both (Figure 4E). Again, combined proteomic and clinical laboratory data performed best.

Having observed clear time trajectories for many proteins and diagnostic parameters, we hypothesized that the molecular signature of the initial host response can be exploited for the prediction of the future disease course. We started by investigating the potential of using the levels of proteins and diagnostic parameters for prediction of future clinical worsening, defined as progression to a higher severity grade on the WHO scale, i.e., a requirement for supplemental low-flow oxygen therapy, high-flow oxygen therapy, or invasive mechanical ventilation. Upon using a linear model to account for current therapy (WHO grade) and age as covariates, 11 proteins and 9 clinical laboratory markers were identified as predictors of future worsening of the clinical condition, across all treatment groups (STAR methods) (Figures 4A and S17; Box 1). Increased or decreased plasma levels of these proteins functioning in inflammation (CRP, ITIH2, SERPINA3, AHSG, and B2M), coagulation (HRG, and PLG), and complement activation (C1R, and CFD), as well as levels of AGT and CST3, were predictive of future clinical deterioration.

Next, we investigated the predictability of the remaining time needed in the hospital for mildly ill patients with maximum WHO grade 3. We identified 26 protein biomarkers and 14 routine diagnostic markers (Figures 4B and S18) that correlate with the time between the first sampling point and discharge from inpatient care. The proteomic signature associated with a longer need for inpatient treatment is characterized by proteins of the complement system (C1QA, C1QB, and C1QC) and reflects altered coagulation (KLKB1, PLG, and SERPIND1) and inflammation (CD14, B2M, SERPINA3, CRP, GPLD1, PGLYRP2, and AHSG). As most of these proteins are also predictors of the required treatment (Figure S6; Table S3), we hypothesized that the time of inpatient care for mild (WHO grade 3) cases correlates with the severity of the disease in these patients. To test this hypothesis, we generated machine learning models for WHO grade prediction, similar to those shown in Figure 4E, but trained the model only on the first time point data measured for each patient (to avoid using any future information with respect to that time point). We observed that the predictions derived from the first time point data correlated with the remaining time in the hospital (Figure 4F). We conclude that machine learning allows us to finely distinguish between more and less severe patients within a single treatment group, i.e. WHO grade.

DISCUSSION

Upfront clinical decision making is essential for optimum treatment allocation to patients as well as for efficient resource management within the hospital. For instance, early referral to intensive care treatment units has been shown to improve prognosis and outcome for patients with severe COVID-19 (Sun et al., 2020). One of the peculiarities of COVID-19 is that the examinable clinical conditions of patients often do not reflect the true severity of the disease, e.g., with respect to respiratory insufficiency. In contrast to patients with severe bacterial pneumonia, patients with COVID-19 often clinically appear to be only slightly affected, despite being in severe respiratory failure, a phenomenon termed ‘‘happy hypoxemia’’ (Stawicki et al., 2020). Clinical decisions therefore need to be supported by objective, molecular diagnostics. These diagnostic analyses help further in the monitoring of therapies and clinical trials as they allow for determining the extent to which a given patient has deviated from the disease trajectory that would be achieved without therapy.

Several recent investigations have identified protein biomarkers and clinical parameters that classify patients with COVID-19 according to disease severity and/or received treatment (D'Alessandro et al., 2020; Laing et al., 2020; Liu et al., 2020b; Messner et al., 2020; Overmyer et al., 2020; Schulte-Schrepping et al., 2020; Shen et al., 2020; Shu et al., 2020;

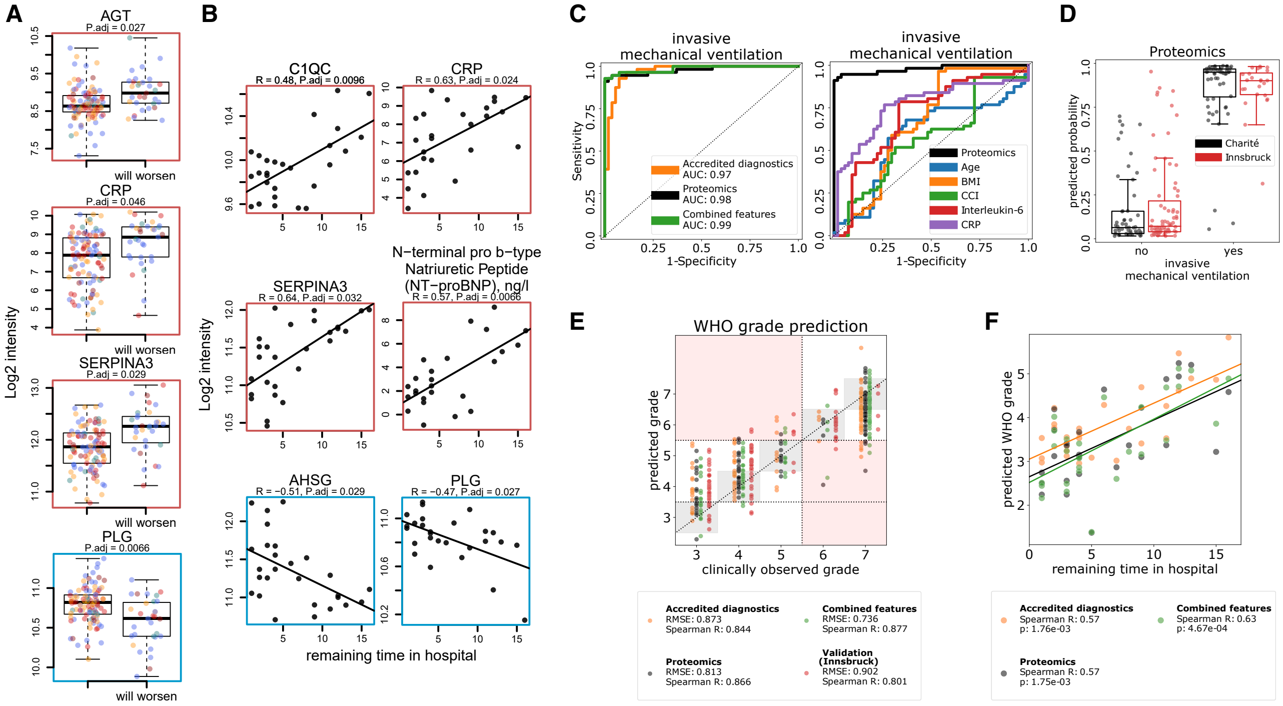

Figure 4. Predicting COVID-19 treatment requirement and future disease progression from the early molecular phenotype by using machine learning.

(A) Selected proteins that are predictive (FDR < 0.05) of the future clinical deterioration of the disease (that is progression to a higher WHO grade in the future; STAR methods). Illustrated are the log_2-transformed levels of the proteins at the first sampling point upon correction (for visualization only) for the impact of the WHO grade and age as covariates (Ritchie et al., 2015) (see also Figure S17).

(B) Selected proteins and routine diagnostic parameters predictive (FDR < 0.05) of the remaining time in hospital for patients receiving mild treatment (WHO grade 3). Statistical testing was performed by including patient's age as a covariate (STAR methods). Illustrated are the log_2-transformed levels of the proteins (upon correction for age as a covariate, for visualization only) at the first sampling point, plotted against the remaining time in hospital (days) (see also Figure S18).

(C) Left: performance of a machine learning model characterizing the need for invasive mechanical ventilation, based on either the proteomic data, accredited diagnostic parameters, or both. Right: comparison of the performance of a machine learning model characterising the need for invasive mechanical ventilation based on proteomic data to established prognostic parameters.

(D) Prediction performance, based on the proteome, visualized as boxplots. Cross-validation predictions on the Charité cohort are shown in black, predictions of a model trained on the Charité data and then applied to an independent cohort from another hospital (Innsbruck cohort) are shown in red.

(E) Prediction of the WHO grade itself using machine learning (cross-validated, first time point at the maximum treatment level for each patient is used, STAR methods), based on either the proteome, clinical diagnostic parameters, or both. The performance of the proteomic model trained on the Charité cohort and applied to the Innsbruck cohort is also shown.

(F) A machine learning model was trained to predict the level of necessary treatment (WHO grade) using the data (proteomics, clinical, or both) from the first time point measured for each patient. Derived predictions for patients who did not receive supplemental oxygen at this time point (WHO = 3) were plotted against the remaining time (days) in hospital for these patients.

Wynants et al., 2020). In other studies, the potential prognostic value of several established and newly discovered markers for predicting the future course of the disease was investigated, e.g., for IL-6, ferritin, or resistin (Grifoni et al., 2020; Meizlish et al., 2020). Yet, it remained challenging so far, to put their prognostic value in relation to patient age and current level of care, the two most important apparent characteristics for prognosis in COVID-19. For instance, a patient at WHO grade 5 who requires high-flow oxygen therapy is significantly more likely to progress to mechanical ventilation and subsequently die than an inpatient at WHO grade 3 that does not require oxygen support. Likewise, a 90-year-old patient at WHO grade 3 is significantly more likely to progress to more severe disease and to stay in the hospital for a longer period of time than a 20-year-old patient at the same WHO grade.

To identify (1) which proteomic markers and laboratory parameters correlate with each other by being attributed to a common biological or physiological response, and (2) which markers reflect disease trajectories, we longitudinally phenotyped 139 individuals admitted at Charité University Hospital, Berlin, Germany, between March 01, 2020, and June 30, 2020, due to PCR-confirmed SARS-CoV-2 infection (Figure S2). We recorded a large panel of 86 parameters with accredited diagnostic tests. These tests capture the compendium of analytical parameters that are available for the clinical routine. In parallel, we record plasma proteomes using a recently developed mass spectrometry platform. This platform introduced the use of analytical (high-flow rate) chromatography to routine proteomics in order to increase throughput and measurement precision to the scale of clinical trials (Messner et al., 2020). The platform reaches a

Box 1. Proteins predictive of future worsening, i.e., disease progression to higher WHO grade

HIGH LEVELS INDICATIVE OF POOR PROGNOSIS

AGT: Angiotensinogen: Conversion via angiotensin-converting enzymes ACE and ACE2 produces AngI/AngII (pro-inflammatory, vasoconstrictive, pro-fibrotic) and Ang1-7/Ang1-9 (anti-inflammatory, vasodilative, anti-fibrotic), respectively (Turner, 2015; Zhang et al., 2020). Increased AGT likely reflects increased AngI/AngII due to SARS-CoV-2 mediated inactivation of ACE2 (Tay et al., 2020) and subsequently predominant conversion of AGT to AngII. AngII correlates with viral load (Liu et al., 2020c) and has tissue damaging effects (Zhang et al., 2020).
B2M: Beta-2-microglobulin: Component of major histocompatibility complex (MHC I) on all nucleated cells and platelets. Released abundantly by activated platelets leading to pro-inflammatory M1-like macrophage polarization (Hilt et al., 2019). Increase of B2M has been associated with death in patients with chronic kidney disease (Makridakis et al., 2020).
C1R: Complement C1r: Initiator of the classical complement pathway (Hajishengallis et al., 2017).
CFD: Complement Factor D: Initiator of the alternative complement pathway by cleaving Factor B (CFB) to form the C3bBb alternative pathway convertase (Volanakis and Narayana, 1996).
CRP: C-reactive protein: Acute phase protein, strongly upregulated in inflammation and infection, including COVID-19.
CST3: Cystatin C: Biomarker of kidney function (Peralta et al., 2011).
SERPINA3: Alpha-1-antichymotrypsin: Protease inhibitor of neutrophil cathepsin G (Benarafa, 2015). When cleaved at reactive site loop, it becomes stable to degradation and becomes a strong neutrophil chemoattractant (Banda et al., 1988; Potempa et al., 1991)

LOW LEVELS INDICATIVE OF POOR PROGNOSIS

AHSG: Alpha-2-HS glycoprotein (Fetuin-A): Negative acute phase protein attenuating macrophage activation and neutrophil degranulation (Ombrellino et al., 2001).
HRG: Histidine-rich glycoprotein: Negative acute phase protein, regulator of inflammation and immune response, clearance of pathogens and cell detritus as well as coagulation and fibrinolysis through a variety of interactions (Poon et al., 2011; Wakabayashi, 2013).
ITIH2: Inter-alpha-trypsin inhibitor heavy chain H2: Covalently linked to bikunin (AMBP), the complex binds to hyalarunan (HA) to form serum-derived hyaluronan-associated protein (SHAP) which has matrix-stabilizing and immunomodulatory effects (Fries and Blom, 2000; Zhuo et al., 2004).
PLG: Plasminogen, Plasmin: Mediator of fibrinolysis (Chapin and Hajjar, 2015). More recently, immunological functions including neutrophil attenuation as well as macrophage efferocytosis and polarization from pro-inflammatory M1 to tissue-repairing M2 phenotype have been identified (Heissig et al., 2020).

similar proteomic depth as other contemporary mass spectrometry technologies that address undepleted human plasma that is constrained by its huge dynamic range (Anderson and Anderson, 2002) (Box 2 for the resources generated).

The comprehensive and time-resolved molecular phenotyping of this patient cohort is complemented by a comparison with a healthy population baseline (Generation Scotland [Smith et al., 2006]) measured with the same proteomic platform (Messner et al., 2020), and the characterization of an independent cohort from an unrelated health care system (Innsbruck cohort, Austria) for validating the created predictors. The measurements were performed on samples collected during the early period of COVID-19, i.e., before immunomodulatory treatments such as dexamethasone became standard of care for severe COVID-19 (RECOVERY Collaborative Group, 2020). Our data thus reflect treatment-naive trajectories, which are of major value as baseline data for future studies.

We report an initial spike in the early inflammatory host response as a determinant for the future course of the disease. As our results indicate, the patients in our cohort showed molecular marker signatures of higher basal inflammation with increasing age, which might be partially responsible for the higher risk of severe COVID-19 in older individuals. While several approaches of targeted anti-inflammatory treatment have not been successful in preventing clinical deterioration in COVID-19 so far (Stone et al., 2020), our study indicates that this special population of older patients might benefit particularly from treatments that mitigate the inflammatory host response. We report numerous interdependencies between clinical laboratory markers and alterations in proteomes, linking, for example, clinical inflammatory markers to components of the complement cascade and the coagulation system. Using machine learning, we show that both plasma proteomes and the compendium of established diagnostic parameters can be used for accurate characterization of disease severity, significantly outperforming established individual risk markers, such as CRP or IL-6 levels. Of note, the combination of proteomic features and clinical laboratory markers repeatedly showed the best performance in the machine learning models. Furthermore, the models generated could be transferred for prediction in an independent cohort from another hospital and healthcare system, highlighting the robustness of this approach and its translational potential.

We observed a considerable overlap between prognostic markers and those that classify treatment according to COVID-19 severity (Figure 5). Out of 49 prognostic markers, 41 correlated with the WHO severity score. As an example, SERPINA3

CellPress
OPEN ACCESS

Box 2. Overview of resources generated

We provide deep and time-resolved resources that characterize COVID-19 at the level of plasma proteomes and established diagnostic parameters. We demonstrate the extent to which proteomes and diagnostic parameters interdepend, in initial response to the disease and in dynamics during the disease course. We show how they change with age, differ depending on the disease severity, reflect the therapy received and evolve over time. Our data have been acquired for COVID-19 patients' samples and analyzed in the context of general population proteomics (Generation Scotland) for which samples have been measured with the same proteomic technology (Messner et al., 2020), but we also expect it to be of high value as a reference for studies of other types of viral pneumonia as well as any investigations involving both routine clinical phenotyping and plasma proteomics.

Summary of the resource data generated in the study.

1. Covariation maps. We provide a covariation map between plasma proteins measured with at least 3 peptides and clinical laboratory measurements (Figure 1B; Table S4). In addition, we provide a full covariation map between all features measured in the study (Figure S3; Table S5) as well as a COVID-19 specific protein-protein covariation map (Figure S4; Table S6). Finally, we also provide a correlation map for the changes of different omics features with time (Figure S12; Table S7).
2. A map of plasma protein levels and clinical laboratory measurements depending on disease severity (Figures S5 and S6; Table S3).
3. Characterization of age-dependency of plasma protein levels and clinical laboratory measurements in COVID-19, and in comparison with the general population (Figures 2C and S9–S11; Table S3).
4. Characterization of the dynamics of plasma protein levels and clinical laboratory measurements during the course of COVID-19 (Figures 3B, S14, and S15; Table S3).
5. Characterization of the predictive power of plasma protein levels and clinical laboratory measurements in COVID-19 (Figures 4, S17, and S18; Table S3).
6. Proteomic and clinical signatures observed in severe COVID-19 patients undergoing RRT and ECMO (Figures S7 and S8; Table S3).

(Alpha-1 antichymotrypsin) can be used for both the classification of severity and prediction of future disease course. Both SERPINA3 and SERPINA1, another classifier of severity, possess anti-inflammatory properties and are involved in the protection of tissues from neutrophil elastase- and cathepsin G-mediated tissue damage (Benarafa, 2015). Our data show a strong correlation of both serpins with levels of neutrophils and NLR in peripheral blood. SERPINA1 is mainly produced by the liver but it is also produced in epithelial cells, pulmonary alveolar cells, tissue macrophages, blood monocytes, and granulocytes. Hence, this finding presumably reflects a systemic response to the increased NLR. After binding to effector enzymes, SERPIN-proteinase complexes are normally rapidly cleared from the blood but become resistant to degradation when cleaved at the reactive site loop (Gettins and Olson, 2016). Cleaved SERPINA1 and SERPINA3 have been shown to act as strong neutrophil chemoattractants (Banda et al., 1988; Potempa et al., 1991). The observed increase in levels of SERPINA1 and SERPINA3 might therefore partly reflect the more stable, chemoattractant, pro-inflammatory cleaved forms, rather than the short-lived tissue-protective proteins in severe COVID-19. Given the prominent role of neutrophil activation in severe COVID-19 (Schulte-Schrepping et al., 2020), this finding merits further investigation.

Our data also highlight angiotensinogen (AGT) as a marker for future worsening. Activation of angiotensinogen occurs via the protease renin and the endogenous angiotensin-converting enzymes ACE or ACE2. ACE converts angiotensin I (AngI) to pro-inflammatory, vasoconstrictive, and pro-fibrotic angiotensin II (AngII) (Zhang et al., 2020). ACE2, in contrast, mediates conversion of angiotensins I and II to anti-inflammatory, vasodilative, anti-fibrotic, and anti-oxidant angiotensins 1–9 (Ang1-9) and 1–7 (Ang1-7) (Turner, 2015). SARS-CoV-2 invades host cells of the lung, heart, kidneys, and other organs via ACE2, resulting in the internalization and downregulation of ACE2 (Hoffmann et al., 2020; Tay et al., 2020; Zhang et al., 2020). Subsequently, angiotensinogen is converted predominantly via ACE to AngII and is less degraded by ACE2, resulting in AngII accumulation (Batlle et al., 2012; Silhol et al., 2020). We can thus assume that the higher plasma levels of AGT gene products in severely ill patients, as measured in our study, mainly reflect the higher levels of AngII. Importantly, we observed a strong correlation of AGT with markers of acute kidney injury (AKI; creatinine, urea; Figures S3 and S13; Table S5), a frequent complication of COVID-19 and a risk factor for poor prognosis and fatal outcome (Fu et al., 2020). Aggravated by the absence of tissue-protective Ang1-7, elevated levels of AngII lead to activation of the renin-angiotensin-system (RAS) and contribute to hypoxic kidney injury (Kasal et al., 2020). Of note, apart from tissue damaging effects, AngII has been shown to linearly correlate with viral load and lung injury in SARS-CoV-2 infection (Liu et al., 2020c).

Overall, many of the markers that are both classifiers and predictors of the future disease course are initiators of the inflammatory response. This group includes some of the key initiators of the complement cascade: C1QA, C1QB, C1QC, C1R, and CFD. In contrast, severity markers without prognostic value largely include downstream effectors of inflammation-associated damage, such as GSN and the circulating actins ACTBL2 and ACTB, ACTG1, and ECM1. Thus, this high-precision, high-throughput approach can help us understand mechanisms of immune-mediated organ damage on a molecular basis.

Despite the high resolution and high throughput of the mass spectrometry platform deployed in our study, the direct translation of our results into clinical practice will require the development of a clinical assay according to FDA or EMA standards.

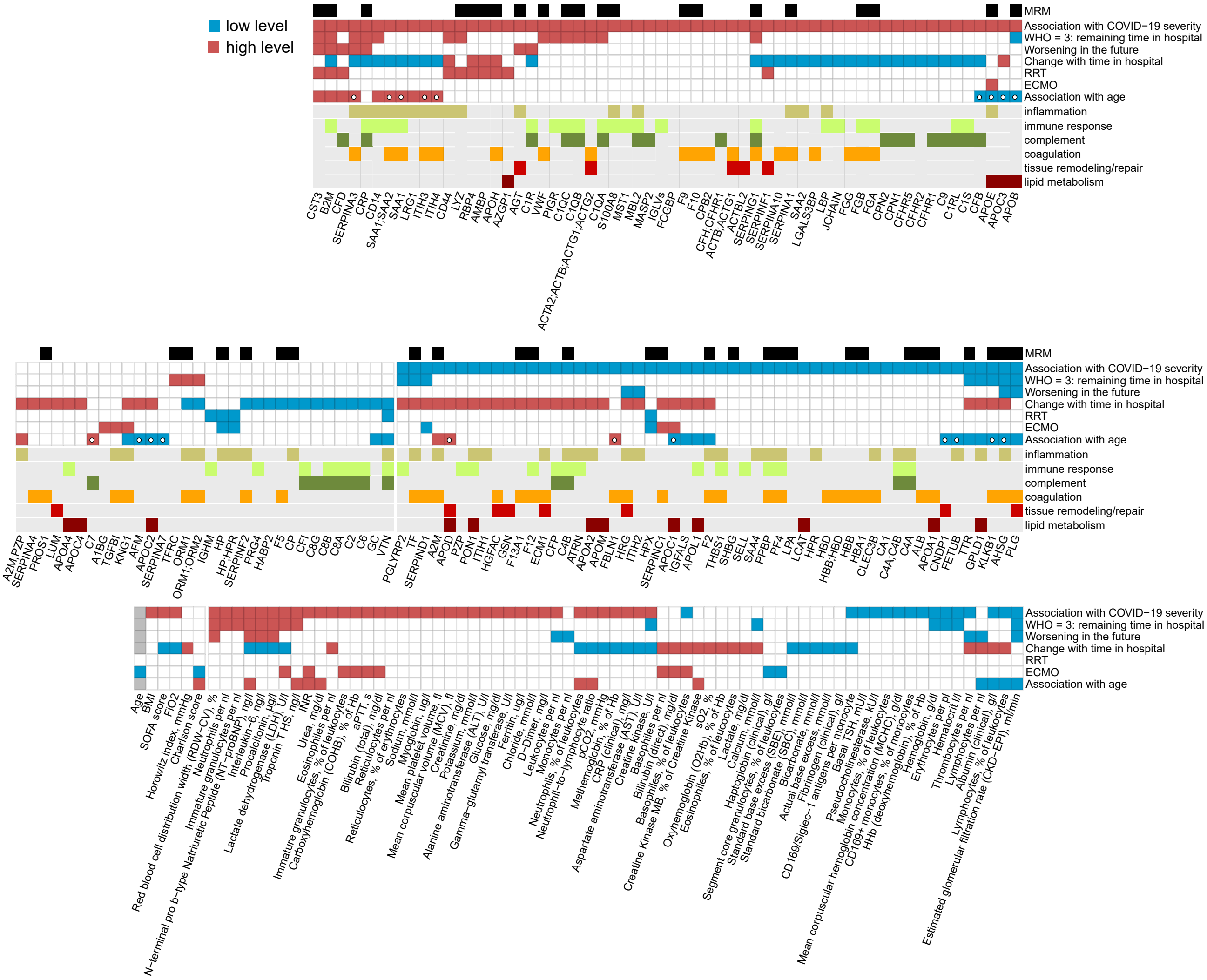

Figure 5. Summary: association of individual plasma proteins, routine diagnostic and physiological parameters with severity, necessary therapy, and progression of COVID-19.

For each statistical test considered (association with WHO grade, prediction of the remaining time in hospital for patients at WHO grade 3, prediction of worsening, i.e., progression to a higher WHO grade in the future, the trend during the peak period of the disease, association with RRT, association with ECMO and association with higher patient age), measurements, which show significant differences are highlighted, with the color indicating the trend, e.g., red for CST3 in the "Association with COVID-19 severity" test indicates higher levels of CST3 in severely ill patients. Proteins for which MRMAssayDB (Bhowmick et al., 2018) lists that a targeted proteomic assay has been developed are indicated with a black bar at the top. Proteins which change significantly with age in the Charité COVID-19 cohort (FDR < 0.05) but do not change significantly (p < 0.05) with age in the general population (Generation Scotland cohort), are highlighted with a white circle in the 7th row ("Association with age"). See also Figures S6–S8, S10, S11, S14, S17, and S18, and Table S3.

We further note that the use of machine learning is currently not a certified method to inform clinical decisions. However, in addition to multiple works that have successfully used machine learning for clinical prognosis previously (see recent reviews [Kelly et al., 2019; Lee and Lee, 2020; Nagendran et al., 2020; Shah et al., 2019; Vollmer et al., 2020]), our results bear a strong implication of the future potential of machine learning for clinical applications, including personalized medicine. This calls for a worldwide effort aimed at developing procedures, which would allow reliable clinical validation of machine learning predictors, their approval, and their routine deployment in the clinic.

In summary, by following a deeply phenotyped COVID-19 patient cohort over time at the level of the proteome and established diagnostic biomarkers and physiological parameters, we have created a rich data resource for understanding the extent and progression of COVID-19. We have shown that an early spike in the inflammatory response is a key determinant of COVID-19, and that future disease progression is predictable by using panels of accredited diagnostic parameters as well as proteomic measurements from early time point samples. By using machine learning, we demonstrated that the proteome allows to precisely characterize the patients' phenotype and that the resulting machine learning models are robust and perform accurately when applied to samples from a different hospital and healthcare system. Our study provides comprehensive information about the key determinants of the varying COVID-19

trajectories as well as marker panels for early prognosis that can be exploited for clinical decision making, to devise personalized therapies, as well as for monitoring the development of much needed COVID-19 treatments.

CONSORTIA

Malte Kleinschmidt, Katrin M. Heim, Belén Millet, Lil Meyer-Arndt, Ralf H. Hübner, Tim Andermann, Jan M. Doehn, Bastian Opitz, Birgit Sawitzki, Daniel Grund, Peter Radünzel, Mariana Schürmann, Thomas Zoller, Florian Alius, Philipp Knape, Astrid Breitbart, Yaosi Li, Felix Bremer, Panagiotis Pergantis, Dirk Schürmann, Bettina Temmesfeld-Wollbrück, Daniel Wendisch, Sophia Brumhard, Sascha S. Haenel, Claudia Conrad, Philipp Georg, Kai-Uwe Eckardt, Lukas Lehner, Jan M. Kruse, Carolin Ferse, Roland Körner, Claudia Spies, Andreas Edel, Steffen Weber-Carstens, Alexander Krannich, Saskia Zvorc, Linna Li, Uwe Behrens, Sein Schmidt, Maria Rönnefarth, Chantip Dang-Heine, Robert Röhle, Emma Lieker, Lucie Kretzler, Isabelle Wirsching, Christian Wollboldt, Yinan Wu, Georg Schwanitz, David Hillus, Stefanie Kasper, Nadine Olk, Alexandra Horn, Dana Briesemeister, Denise Treue, Michael Hummel, Victor M. Corman, Christian Drosten, and Christof von Kalle

STAR★METHODS

Detailed methods are provided in the online version of this paper and include the following:

- KEY RESOURCES TABLE
- RESOURCE AVAILABILITY
  - Lead contact
  - Materials availability
  - Data and code availability
  - Experimental model and subject details
  - Innsbruck Patient cohort and clinical data
- METHOD DETAILS
  - Materials
  - Mass spectrometry
- QUANTIFICATION AND STATISTICAL ANALYSIS
  - Data analysis
  - Markers of the disease severity
  - Markers varying with age in COVID-19
  - Markers of RRT and ECMO
  - Markers predictive of time in hospital
  - Markers predictive of disease worsening
  - Peak period of the disease definition
  - Markers changing during the peak of disease
  - Correlation maps
  - Prediction of current mechanical ventilation
  - WHO grade prediction
  - Prediction of the remaining time in hospital
  - Supplementary Note 1. Diagnostic parameters and Proteome signatures that indicate therapeutic interventions
  - Supplementary Note 2. Age-specific response to COVID-19 in the context of severity markers
  - Supplementary Note 3. Diverging trends at the proteome level during the disease peak in individual patients

SUPPLEMENTAL INFORMATION

Supplemental information can be found online at https://doi.org/10.1016/j.cels.2021.05.005.

ACKNOWLEDGMENTS

We thank Robert Lane, Jean-Baptiste Vincedent and Nick Morrice (Sciex) for help with the TripleTOF 6600. This work was supported by the Berlin University Alliance (501_Massenspektrometrie, 501_Linklab, 112_PreEP_Corona_Ralser), by UKRI/NIHR through the UK Coronavirus Immunology Consortium (UK-CIC), the BMBF/DLR Projektträger (01KI20160A, 01ZX1604B, 01KI20337, 01KX2021), Charité-BIH Centrum für Therapieforschung (BIH_PA_covid-19_Ralser), the BBSRC (BB/N015215/1, BB/N015282/1), the Francis Crick Institute, which receives its core funding from Cancer Research UK (FC001134), the UK Medical Research Council (FC001134), and the Wellcome Trust (FC001134 and IA 200829/Z/16/Z), as well as the European Research Council (SyG 951475 to M.R.). This work was further supported by the Ministry of Education and Research (BMBF), as a part of the National Research Node 'mass spectrometry in Systems Medicine (MSCoresys), under grant agreement 031L0220A. This study was further supported by the German Federal Ministry of Education and Research (NaFoUniMedCovid19 – NUM-NAPKON, NUM-COVIM, FKZ: 01KX2021 and PROVID—FKZ: 01KI20160A) to F.K., L.E.S., M.W., N.S., and S.H.; L.E.S. is supported by the German Research Foundation (DFG, SFB-TR84 114933180) and by the Berlin Institute of Health (BIH), which receives funding from the Ministry of Education and Research (BMBF). M.W. is supported by grants from the German Research Foundation, SFB-TR84 C06 and C09, by the German Ministry of Education and Research (BMBF) in the framework of the CAPSyS (01ZX1304B), CAPSyS-COVID (01ZX1604B), SYMPATH (01ZX1906A) and PROVID project (01KI20160A) and by the Berlin Institute of Health (CM-COVID). S.H. is supported by the German Research Foundation (DFG, SFB-TR84 A04 and B06), and the BMBF (PROVID, and project 01KI2082). N.S. is supported by grants from the German Research Foundation, SFB-TR84 C09 und Z02, by the German Ministry of Education and Research (BMBF) in the framework of the PROGRESS 01KI07114. This study was further supported by Wellcome Trust (200829/Z/16/Z). The Generation Scotland study received core support from the Chief Scientist Office of the Scottish Government Health Directorates (CZD/16/6) and the Scottish Funding Council (HR03006), and is now supported by the Wellcome Trust (216767/Z/19/Z). A.C. is funded by HDR UK and the Wellcome Trust (216767/Z/19/Z). C.H. is supported by an MRC University Unit Programme grant (MC_UU_00007/10) (QTL in Health and Disease). R.M. is supported by an Alzheimer's Research UK project grant (ARUK-PG2017B-10). H.W., J.F.T., A.Z., and T.N. are supported by a Medical Research Council grant (MR/R02524X/1) and H.W., A.Z., and O.B. by the Ministry of Science and Higher Education agreement no. 075-15-2020-808. H.W. is supported by the National Institute for Health Research (NIHR) Imperial Biomedical Research Centre (BRC). J.F.T is supported by the National Institute for Health Research (NIHR) UCLH/UCL Biomedical Research Centre. M.M. is a participant in the Bih-Charité Digital Clinician Scientist Program funded by the Charité – Universitätsmedizin Berlin, the Berlin Institute of Health, and the German Research Foundation (DFG). M.A.K. is supported by the Austrian Science Funds (FWF; P33333) and the Austrian Research Promotion Agency (FFG, #878654). Figures were created with biorender.com

AUTHOR CONTRIBUTIONS

L.E.S., M.R., and F.K. designed the study. A.F., D.L., and M.M. conducted experiments. P.T.-L., C.T., A.F., D.L., E.T.H., P.S., C.B.M., M.J., T.S., S.J.K., A.P., Y.W., S.S., R.H., B.S., S.W., M.M., F.M., C.G., C.R.-G., T.L., L.B.J., M.S.S., M.P., L.J., S.D., L.J.L., D.Z., P.E., A.U., H.Z., J.L.-R., M.A.K., I.T., H.M.-R., M.W., N.S., L.E.S., and F.K. acquired clinical samples and data. V.D., P.T.-L., O.L., T.N., C.T., H.W., S.K.A., A.R., N.-M.G., L.S., S.V., M.W., C.B.M., O.B., A.Z., A.C., C.H., D.J.P., R.E.M., J.F.T., A.Z., K.L., S.H., M.M., M.R., and F.K. analyzed the data. V.D., P.T.-L., C.T., C.C.-M., E.T.H., P.S., S.H., M.R., M.M., T.L., L.J.L., K.L., L.E.S., M.R., and F.K. interpreted the data. V.D., P.T.-L., M.R., F.K., and L.E.S. wrote the first draft of the manuscript. All authors contributed to finalizing the manuscript.

DECLARATION OF INTERESTS

The authors declare no competing interests.

Received: November 11, 2020
Revised: March 24, 2021
Accepted: May 7, 2021
Published: June 14, 2021

REFERENCES

Ağirbaşli, M., Song, J., Lei, F., Wang, S., Kunselman, A.R., Clark, J.B., Myers, J.L., and Ündar, A. (2015). Apolipoprotein E levels in pediatric patients undergoing cardiopulmonary bypass. Artif. Organs *39*, 28–33.

Alwan, N.A., Burgess, R.A., Ashworth, S., Beale, R., Bhadelia, N., Bogaert, D., Dowd, J., Eckerle, I., Goldman, L.R., Greenhalgh, T., et al. (2020). Scientific consensus on the COVID-19 pandemic: we need to act now. Lancet *396*, e71–e72.

Anderson, N.L., and Anderson, N.G. (2002). The human plasma proteome: history, character, and diagnostic prospects. Mol. Cell. Proteomics *1*, 845–867.

ARDS Definition Task Force, Ranieri, V.M., Rubenfeld, G.D., Thompson, B.T., Ferguson, N.D., Caldwell, E., Fan, E., Camporota, L., and Slutsky, A.S. (2012). Acute respiratory distress syndrome: the Berlin definition. JAMA *307*, 2526–2533.

Banda, M.J., Rice, A.G., Griffin, G.L., and Senior, R.M. (1988). Alpha 1-proteinase inhibitor is a neutrophil chemoattractant after proteolytic inactivation by macrophage elastase. J. Biol. Chem. *263*, 4481–4484.

Batlle, D., Wysocki, J., Soler, M.J., and Ranganath, K. (2012). Angiotensin-converting enzyme 2: enhancing the degradation of angiotensin II as a potential therapy for diabetic nephropathy. Kidney Int *81*, 520–528.

Benarafa, C. (2015). Regulation of neutrophil serine proteases by intracellular serpins. In The Serpin Family: Proteins with Multiple Functions in Health and Disease, M. Geiger, F. Wahlmüller, and M. Furtmüller, eds. (Springer International Publishing)), pp. 59–76.

Benjamini, Y., and Hochberg, Y. (1995). Controlling the false discovery rate: a practical and powerful approach to multiple testing. Journal of the Royal Statistical Society: Series B (Methodological) *57*, 289–300.

Bhowmick, P., Mohammed, Y., and Borchers, C.H. (2018). MRMAssayDB: an integrated resource for validated targeted proteomics assays. Bioinformatics *34*, 3566–3571.

Blumenthal, D., Fowler, E.J., Abrams, M., and Collins, S.R. (2020). Covid-19 - implications for the health care system. N. Engl. J. Med. *383*, 1483–1488.

Chapin, J.C., and Hajjar, K.A. (2015). Fibrinolysis and the control of blood coagulation. Blood Rev *29*, 17–24.

Chen, T., and Guestrin, C. (2016). XGBoost: A scalable tree boosting system. In Proceedings of the 22nd ACM SIGKDD international conference on Knowledge Discovery and Data Mining, pp. 785–794.

Chung, H.Y., Kim, D.H., Lee, E.K., Chung, K.W., Chung, S., Lee, B., Seo, A.Y., Chung, J.H., Jung, Y.S., Im, E., et al. (2019). Redefining chronic inflammation in aging and age-related diseases: proposal of the Senoinflammation concept. Aging Dis *10*, 367–382.

Cox, J., Hein, M.Y., Luber, C.A., Paron, I., Nagaraj, N., and Mann, M. (2014). Accurate proteome-wide label-free quantification by delayed normalization and maximal peptide ratio extraction, termed MaxLFQ. Mol. Cell. Proteomics *13*, 2513–2526.

D'Alessandro, A., Thomas, T., Dzieciatkowska, M., Hill, R.C., Francis, R.O., Hudson, K.E., Zimring, J.C., Hod, E.A., Spitalnik, S.L., and Hansen, K.C. (2020). Serum proteomics in COVID-19 patients: altered coagulation and complement status as a function of IL-6 level. J. Proteome Res. *19*, 4417–4427.

Danwang, C., Endomba, F.T., Nkeck, J.R., Wouna, D.L.A., Robert, A., and Noubiap, J.J. (2020). A meta-analysis of potential biomarkers associated with severity of coronavirus disease 2019 (COVID-19). Biomark. Res. *8*, 37.

Dassati, S., Waldner, A., and Schweigreiter, R. (2014). Apolipoprotein D takes center stage in the stress response of the aging and degenerative brain. Neurobiol. Aging *35*, 1632–1642.

Demichev, V., Messner, C.B., Vernardis, S.I., Lilley, K.S., and Ralser, M. (2020). DIA-NN: neural networks and interference correction enable deep proteome coverage in high throughput. Nat. Methods *17*, 41–44.

Do Carmo, S., Jacomy, H., Talbot, P.J., and Rassart, E. (2008). Neuroprotective effect of apolipoprotein D against human coronavirus OC43-induced encephalitis in mice. J. Neurosci. *28*, 10330–10338.

Ferrucci, L., Corsi, A., Lauretani, F., Bandinelli, S., Bartali, B., Taub, D.D., Guralnik, J.M., and Longo, D.L. (2005). The origins of age-related proinflammatory state. Blood *105*, 2294–2299.

Figueroa, D.M., Gordon, E.M., Yao, X., and Levine, S.J. (2019). Apolipoproteins as context-dependent regulators of lung inflammation. In Mechanisms and Manifestations of Obesity in Lung Disease, R.A. Johnston and B.T. Suratt, eds. (Academic Press), pp. 301–326.

Franceschi, C., Garagnani, P., Parini, P., Giuliani, C., and Santoro, A. (2018). Inflammaging: a new immune-metabolic viewpoint for age-related diseases. Nat. Rev. Endocrinol. *14*, 576–590.

Fries, E., and Blom, A.M. (2000). Bikunin–not just a plasma proteinase inhibitor. Int. J. Biochem. Cell Biol. *32*, 125–137.

Fu, E.L., Janse, R.J., de Jong, Y., van der Endt, V.H.W., Milders, J., van der Willik, E.M., de Rooij, E.N.M., Dekkers, O.M., Rotmans, J.I., and van Diepen, M. (2020). Acute kidney injury and kidney replacement therapy in COVID-19: a systematic review and meta-analysis. Clin. Kidney J. *13*, 550–563.

Gettins, P.G.W., and Olson, S.T. (2016). Inhibitory serpins. New insights into their folding, polymerization, regulation and clearance. Biochem. J. *473*, 2273–2293.

Gillet, L.C., Navarro, P., Tate, S., Röst, H., Selevsek, N., Reiter, L., Bonner, R., and Aebersold, R. (2012). Targeted data extraction of the MS/MS spectra generated by data- independent acquisition: a new concept for consistent and accurate proteome analysis. Mol. Cell. Proteomics *11*, O111.016717.

Gordon, S.M. (2014). Proteomic diversity in HDL: a driving force for particle function and target for therapeutic intervention. In The HDL Handbook, Second Edition, T. Komoda, ed. (Academic Press), pp. 293–322.

Goronzy, J.J., and Weyand, C.M. (2013). Understanding immunosenescence to improve responses to vaccines. Nat. Immunol. *14*, 428–436.

Grifoni, E., Valoriani, A., Cei, F., Lamanna, R., Gelli, A.M.G., Ciambotti, B., Vannucchi, V., Moroni, F., Pelagatti, L., Tarquini, R., et al. (2020). Interleukin-6 as prognosticator in patients with COVID-19. J. Infect. *81*, 452–482.

Gu, Z., Eils, R., and Schlesner, M. (2016). Complex heatmaps reveal patterns and correlations in multidimensional genomic data. Bioinformatics *32*, 2847–2849.

Hadjadj, J., Yatim, N., Barnabei, L., Corneau, A., Boussier, J., Smith, N., Péré, H., Charbit, B., Bondet, V., Chenevier-Gobeaux, C., et al. (2020). Impaired type I interferon activity and inflammatory responses in severe COVID-19 patients. Science *369*, 718–724.

Hajishengallis, George, Reis, E.S., Mastellos, D.C., Ricklin, D., and Lambris, J.D. (2017). Novel mechanisms and functions of complement. Nature Immunology *18*, 1288–1298. https://doi.org/10.1038/ni.3858.

Han, S., Yang, K., Zhu, H., Liu, J., Zhang, L., and Zhao, J. (2018). Proteomics investigation of the changes in serum proteins after high- and low-flux hemodialysis. Ren. Fail. *40*, 506–513.

Harris, S.E., Riggio, V., Evenden, L., Gilchrist, T., McCafferty, S., Murphy, L., Wrobel, N., Taylor, A.M., Corley, J., Pattie, A., et al. (2017). Age-related gene expression changes, and transcriptome wide association study of physical and cognitive aging traits, in the Lothian Birth Cohort 1936. Aging *9*, 2489–2503.

Heissig, B., Salama, Y., Takahashi, S., Osada, T., and Hattori, K. (2020). The multifaceted role of plasminogen in inflammation. Cell Signal *75*. https://doi.org/10.1016/j.cellsig.2020.109761.

Henry, B.M., de Oliveira, M.H.S., Benoit, S., Plebani, M., and Lippi, G. (2020). Hematologic, biochemical and immune biomarker abnormalities associated with severe illness and mortality in coronavirus disease 2019 (COVID-19): a meta-analysis. Clin. Chem. Lab. Med. *58*, 1021–1028.

Hilt, Z.T., Pariser, D.N., Ture, S.K., Mohan, A., Quijada, P., Asante, A.A., Cameron, S.J., Sterling, J.A., Merkel, A.R., Johanson, A.L., et al. (2019).

CellPress
OPEN ACCESS

Platelet-derived β2M regulates monocyte inflammatory responses. JCI Insight *4*. https://doi.org/10.1172/jci.insight.122943.

Hoffmann, M., Kleine-Weber, H., Schroeder, S., Krüger, N., Herrler, T., Erichsen, S., Schiergens, T.S., Herrler, G., Wu, N.-H., Nitsche, A., et al. (2020). SARS-CoV-2 cell entry depends on ACE2 and TMPRSS2 and is blocked by a clinically proven protease inhibitor. Cell *181*, 271–280.e8.

Kasal, D.A., De Lorenzo, A., and Tibiriçá, E. (2020). COVID-19 and microvascular disease: pathophysiology of SARS-CoV-2 infection with focus on the renin-angiotensin system. Heart Lung Circ *29*, 1596–1602.

Kelly, C.J., Karthikesalingam, A., Suleyman, M., Corrado, G., and King, D. (2019). Key challenges for delivering clinical impact with artificial intelligence. BMC Med *17*, 195.

Kurth, F., Roenneferth, M., Thibeault, C., Corman, V.M., Müller-Redetzky, H., Mittermaier, M., Ruwwe-Glösenkamp, C., Heim, K.M., Krannich, A., Zvorc, S., et al. (2020). Studying the pathophysiology of coronavirus disease 2019: a protocol for the Berlin prospective COVID-19 patient cohort (Pa-COVID-19). Infection *48*, 619–626.

Laing, A.G., Lorenc, A., Del Molino Del Barrio, I., Das, A., Fish, M., Monin, L., Muñoz-Ruiz, M., McKenzie, D.R., Hayday, T.S., Francos-Quijorna, I., et al. (2020). A dynamic COVID-19 immune signature includes associations with poor prognosis. Nat. Med. *26*, 1623–1635.

Lee, C.S., and Lee, A.Y. (2020). Clinical applications of continual learning machine learning. Lancet Digit. Health *2*, e279–e281.

Lian, J., Jin, C., Hao, S., Zhang, X., Yang, M., Jin, X., Lu, Y., Hu, J., Zhang, S., Zheng, L., et al. (2020). High neutrophil-to-lymphocyte ratio associated with progression to critical illness in older patients with COVID-19: a multicenter retrospective study. Aging *12*, 13849–13859.

Liu, J., Liu, Y., Xiang, P., Pu, L., Xiong, H., Li, C., Zhang, M., Tan, J., Xu, Y., Song, R., et al. (2020a). Neutrophil-to-lymphocyte ratio predicts critical illness patients with 2019 coronavirus disease in the early stage. J. Transl. Med. *18*, 206.

Liu, Y., Gao, W., Guo, W., Guo, Y., Shi, M., Dong, G., Ge, Q., Zhu, J., and Lu, J. (2020b). Prominent coagulation disorder is closely related to inflammatory response and could be as a prognostic indicator for ICU patients with COVID-19. J. Thromb. Thrombolysis *50*, 825–832.

Liu, Y., Yang, Y., Zhang, C., Huang, F., Wang, F., Yuan, J., Wang, Z., Li, J., Li, J., Feng, C., et al. (2020c). Clinical and biochemical indexes from 2019-nCoV infected patients linked to viral loads and lung injury. Sci. China Life Sci. *63*, 364–374.

Liu, Y.C., Zou, X.B., Chai, Y.F., and Yao, Y.M. (2014). Macrophage polarization in inflammatory diseases. Int. J. Biol. Sci. *10*, 520–529.

Luo, Z., Lei, H., Sun, Y., Liu, X., and Su, D.F. (2015). Orosomucoid, an acute response protein with multiple modulating activities. J. Physiol. Biochem. *71*, 329–340.

Makridakis, M., Kontostathi, G., Petra, E., Stroggilos, R., Lygirou, V., Filip, S., Duranton, F., Mischak, H., Argiles, A., Zoidakis, J., and Vlahou, A. (2020). Multiplexed MRM-based protein quantification of putative prognostic biomarkers for chronic kidney disease progression in plasma. Sci. Rep. *10*, 4815.

McDonnell, T., Wincup, C., Buchholz, I., Pericleous, C., Giles, I., Ripoll, V., Cohen, H., Delcea, M., and Rahman, A. (2020). The role of beta-2-glycoprotein I in health and disease associating structure with function: more than just APS. Blood Rev *39*, 100610.

Meizlish, M.L., Pine, A.B., Bishai, J.D., Goshua, G., Nadelmann, E.R., Simonov, M., Chang, C.-H., Zhang, H., Shallow, M., Bahel, P., et al. (2020). A neutrophil activation signature predicts critical illness and mortality in COVID-19. medRxiv. https://doi.org/10.1182/bloodadvances.2020003568.

Messner, C.B., Demichev, V., Wendisch, D., Michalick, L., White, M., Freiwald, A., Textoris-Taube, K., Vernardis, S.I., Egger, A.-S., Kreidl, M., et al. (2020). Ultra-high-throughput clinical proteomics reveals classifiers of COVID-19 infection. Cell Syst *11*, 11–24.e4.

Millard, S.P. (2014). EnvStats, an RPackage for environmental statistics. In Wiley StatsRef: Statistics Reference Online, N. Balakrishnan, T. Colton, B. Everitt, W. Piegorsch, F. Ruggeri, and J.L. Teugels, eds. (Wiley).

Muffat, J., and Walker, D.W. (2010). Apolipoprotein D: an overview of its role in aging and age-related diseases. Cell Cycle *9*, 269–273.

Murphy, A.J., Akhtari, M., Tolani, S., Pagler, T., Bijl, N., Kuo, C.L., Wang, M., Sanson, M., Abramowicz, S., Welch, C., et al. (2011). ApoE regulates hematopoietic stem cell proliferation, monocytosis, and monocyte accumulation in atherosclerotic lesions in mice. J. Clin. Invest. *121*, 4138–4149.

Nagendran, M., Chen, Y., Lovejoy, C.A., Gordon, A.C., Komorowski, M., Harvey, H., Topol, E.J., Ioannidis, J.P.A., Collins, G.S., and Maruthappu, M. (2020). Artificial intelligence versus clinicians: systematic review of design, reporting standards, and claims of deep learning studies. BMJ *368*, m689.

WHO. (2020). R&D Blueprint-Novel Coronavirus COVID-19 Therapeutic Trial Synopsis (World Health Organization).

Ombrellino, M., Wang, H., Yang, H., Zhang, M., Vishnubhakat, J., Frazier, A., Scher, L.A., Friedman, S.G., and Tracey, K.J. (2001). Fetuin, a negative acute phase protein, attenuates TNF synthesis and the innate inflammatory response to carrageenan. Shock *15*, 181–185.

Overmyer, K.A., Shishkova, E., Miller, I.J., Balnis, J., Bernstein, M.N., Peters-Clarke, T.M., Meyer, J.G., Quan, Q., Muehlbauer, L.K., Trujillo, E.A., et al. (2020). Large-scale multi-omic analysis of COVID-19 severity. medRxiv. https://doi.org/10.1101/2020.07.17.20156513.

Page, C., Goicochea, L., Matthews, K., Zhang, Y., Klover, P., Holtzman, M.J., Hennighausen, L., and Frieman, M. (2012). Induction of alternatively activated macrophages enhances pathogenesis during severe acute respiratory syndrome coronavirus infection. J. Virol. *86*, 13334–13349.

Pascual, M., Steiger, G., Estreicher, J., Macon, K., Volanakis, J.E., and Schifferli, J.A. (1988). Metabolism of complement factor D in renal failure. Kidney Int *34*, 529–536.

Patricio, P., Paiva, J.A., and Borrego, L.M. (2019). Immune response in bacterial and candida sepsis. Eur. J. Microbiol. Immunol. (Bp) *9*, 105–113.

Pedregosa, F., Varoquaux, G., Gramfort, A., Michel, V., Thirion, B., Grisel, O., Blondel, M., Prettenhofer, P., Weiss, R., Dubourg, V., et al. (2011). Scikit-learn: machine learning in Python. J. Mach. Learn. Res. *12*, 2825–2830.

Peralta, C.A., Shlipak, M., Judd, S., Cushman, M., McClellan, W., Zakai, N.A., Safford, M.M., Zhang, X., Muntner, P., and Warnock, D. (2011). Detection of chronic kidney disease with creatinine, cystatin C, and urine albumin-to-creatinine ratio and association with progression to end-stage renal disease and mortality. JAMA *305*, 1545–1552.

Perez-Riverol, Y., Csordas, A., Bai, J., Bernal-Llinares, M., Hewapathirana, S., Kundu, D.J., Inuganti, A., Griss, J., Mayer, G., Eisenacher, M., et al. (2019). The PRIDE database and related tools and resources in 2019: improving support for quantification data. Nucleic Acids Res *47*, D442–D450.

Peters, M.J., Joehanes, R., Pilling, L.C., Schurmann, C., Conneely, K.N., Powell, J., Reinmaa, E., Sutphin, G.L., Zhernakova, A., Schramm, K., et al. (2015). The transcriptional landscape of age in human peripheral blood. Nat. Commun. *6*, 8570.

Pham, T.V., Henneman, A.A., and Jimenez, C.R. (2020). iq: an R package to estimate relative protein abundances from ion quantification in DIA-MS-based proteomics. Bioinformatics *36*, 2611–2613.

Phua, J., Weng, L., Ling, L., Egi, M., Lim, C.M., Divatia, J.V., Shrestha, B.R., Arabi, Y.M., Ng, J., Gomersall, C.D., et al. (2020). Intensive care management of coronavirus disease 2019 (COVID-19): challenges and recommendations. Lancet Respir. Med. *8*, 506–517.

Poon, I.K.H., Patel, K.K., Davis, D.S., Parish, C.R., and Hulett, M.D. (2011). Histidine-rich glycoprotein: the Swiss Army knife of mammalian plasma. Blood *117*, 2093–2101.

Potempa, J., Fedak, D., Dubin, A., Mast, A., and Travis, J. (1991). Proteolytic inactivation of alpha-1-anti-chymotrypsin. Sites of cleavage and generation of chemotactic activity. J. Biol. Chem. *266*, 21482–21487.

Poynard, T., and Imbert-Bismut, F. (2012). Laboratory testing for liver disease. In Zakim and Boyer's Hepatology, Sixth Edition, T.D. Boyer, M.P. Manns, and A.J. Sanyal, eds. (W.B. Saunders), pp. 201–215.

Ramasamy, I. (2014). Recent advances in physiological lipoprotein metabolism. Clin. Chem. Lab. Med. *52*, 1695–1727.

Rea, I.M., Gibson, D.S., McGilligan, V., McNerlan, S.E., Alexander, H.D., and Ross, O.A. (2018). Age and age-related diseases: role of inflammation triggers and cytokines. Front. Immunol. *9*, 586.

RECOVERY Collaborative Group (2020). Dexamethasone in hospitalized patients with Covid-19 - preliminary report. N. Engl. J. Med. *384*, 693–704.

Rehman, A.A., Ahsan, H., and Khan, F.H. (2013). α-2-macroglobulin: a physiological guardian. J. Cell. Physiol. *228*, 1665–1675.

Ritchie, M.E., Phipson, B., Wu, D., Hu, Y., Law, C.W., Shi, W., and Smyth, G.K. (2015). limma powers differential expression analyses for RNA-sequencing and microarray studies. Nucleic Acids Res *43*, e47.

Rosenbaum, L. (2020). The untold toll - the pandemic's effects on patients without Covid-19. N. Engl. J. Med. *382*, 2368–2371.

Sack, G.H., Jr. (2018). Serum amyloid A - a review. Mol. Med. *24*, 46.

Saxena, S.K. (2020). Coronavirus Disease 2019 (COVID-19): Epidemiology, Pathogenesis, Diagnosis, And Therapeutics (Springer nature).

Schneeman, T.A., Bruno, M.E.C., Schjerven, H., Johansen, F.E., Chady, L., and Kaetzel, C.S. (2005). Regulation of the polymeric Ig receptor by signaling through TLRs 3 and 4: linking innate and adaptive immune responses. J. Immunol *175*, 376–384.

Schulte-Schrepping, J., Reusch, N., Paclik, D., Baßler, K., Schlickeiser, S., Zhang, B., Krämer, B., Krammer, T., Brumhard, S., Bonaguro, L., et al. (2020). Severe COVID-19 is marked by a dysregulated myeloid cell compartment. Cell *182*, 1419–1440.e23.

Shah, P., Kendall, F., Khozin, S., Goosen, R., Hu, J., Laramie, J., Ringel, M., and Schork, N. (2019). Artificial intelligence and machine learning in clinical development: a translational perspective. NPJ Digit. Med. *2*, 69.

Shao, B., de Boer, I., Tang, C., Mayer, P.S., Zelnick, L., Afkarian, M., Heinecke, J.W., and Himmelfarb, J. (2015). A cluster of proteins implicated in kidney disease is increased in high-density lipoprotein isolated from hemodialysis subjects. J. Proteome Res. *14*, 2792–2806.

Sharma, N.K., Tashima, A.K., Brunialti, M.K.C., Ferreira, E.R., Torquato, R.J.S., Mortara, R.A., Machado, F.R., Assuncao, M., Rigato, O., and Salomao, R. (2017). Proteomic study revealed cellular assembly and lipid metabolism dysregulation in sepsis secondary to community-acquired pneumonia. Sci. Rep. *7*, 15606.

Shen, B., Yi, X., Sun, Y., Bi, X., Du, J., Zhang, C., Quan, S., Zhang, F., Sun, R., Qian, L., et al. (2020). Proteomic and metabolomic characterization of COVID-19 patient sera. Cell *182*, 59–72.e15.

Shu, T., Ning, W., Wu, D., Xu, J., Han, Q., Huang, M., Zou, X., Yang, Q., Yuan, Y., Bie, Y., et al. (2020). Plasma proteomics identify biomarkers and pathogenesis of COVID-19. Immunity *53*, 1108–1122.e5.

Silhol, F., Sarlon, G., Deharo, J.C., and Vaïsse, B. (2020). Downregulation of ACE2 induces overstimulation of the renin-angiotensin system in COVID-19: should we block the renin-angiotensin system? Hypertens. Res. *43*, 854–856.

Singer, M., Deutschman, C.S., Seymour, C.W., Shankar-Hari, M., Annane, D., Bauer, M., Bellomo, R., Bernard, G.R., Chiche, J.D., Coopersmith, C.M., et al. (2016). The third international consensus definitions for sepsis and septic shock (Sepsis-3). JAMA *315*, 801–810.

Smith, B.H., Campbell, H., Blackwood, D., Connell, J., Connor, M., Deary, I.J., Dominiczak, A.F., Fitzpatrick, B., Ford, I., Jackson, C., et al. (2006). Generation Scotland: the Scottish Family Health Study; a new resource for researching genes and heritability. BMC Med. Genet. *7*, 74.

Smyth, G.K. (2004). Linear models and empirical bayes methods for assessing differential expression in microarray experiments. Stat. Appl. Genet. Mol. Biol. *3*, article3.

Soysal, P., Stubbs, B., Lucato, P., Luchini, C., Solmi, M., Peluso, R., Sergi, G., Isik, A.T., Manzato, E., Maggi, S., et al. (2016). Inflammation and frailty in the elderly: a systematic review and meta-analysis. Ageing Res. Rev. *31*, 1–8.

Stawicki, S.P., Jeanmonod, R., Miller, A.C., Paladino, L., Gaieski, D.F., Yaffee, A.Q., De Wulf, A., Grover, J., Papadimos, T.J., Bloem, C., et al. (2020). The 2019–2020 novel coronavirus (severe acute respiratory syndrome coronavirus 2) pandemic: a joint American College of Academic International Medicine-world academic council of emergency medicine multidisciplinary COVID-19 working group consensus paper. J. Glob. Infect. Dis. *12*, 47–93.

Stone, J.H., Frigault, M.J., Serling-Boyd, N.J., Fernandes, A.D., Harvey, L., Foulkes, A.S., Horick, N.K., Healy, B.C., Shah, R., Bensaci, A.M., et al. (2020). Efficacy of tocilizumab in patients hospitalized with Covid-19. N. Engl. J. Med. *383*, 2333–2344.

Sun, Q., Qiu, H., Huang, M., and Yang, Y. (2020). Lower mortality of COVID-19 by early recognition and intervention: experience from Jiangsu Province. Ann. Intensive Care *10*, 33.

Tavazoie, M.F., Pollack, I., Tanqueco, R., Ostendorf, B.N., Reis, B.S., Gonsalves, F.C., Kurth, I., Andreu-Agullo, C., Derbyshire, M.L., Posada, J., et al. (2018). LXR/ApoE activation restricts innate immune suppression in cancer. Cell *172*, 825–840.e18.

Tay, M.Z., Poh, C.M., Rénia, L., MacAry, P.A., and Ng, L.F.P. (2020). The trinity of COVID-19: immunity, inflammation and intervention. Nat. Rev. Immunol. *20*, 363–374.

Turner, A.J. (2015). ACE2 cell biology, regulation, and physiological functions. In The Protective Arm of the Renin Angiotensin System (RAS), T. Unger, U.M. Steckelings, and R.A.S. dos Santos, eds. (Academic Press), pp. 185–189.

Turula, H., and Wobus, C.E. (2018). The role of the polymeric immunoglobulin receptor and secretory immunoglobulins during mucosal infection and immunity. Viruses *10*, 237.

Virtanen, P., Gommers, R., Oliphant, T.E., Haberland, M., Reddy, T., Cournapeau, D., Burovski, E., Peterson, P., Weckesser, W., Bright, J., et al. (2020). SciPy 1.0: fundamental algorithms for scientific computing in Python. Nat. Methods *17*, 261–272.

Volanakis, J.E., and Narayana, S.V. (1996). Complement factor D, a novel serine protease. Protein Sci *5*, 553–564.

Vollmer, S., Mateen, B.A., Bohner, G., Király, F.J., Ghani, R., Jonsson, P., Cumbers, S., Jonas, A., McAllister, K.S.L., Myles, P., et al. (2020). Machine learning and artificial intelligence research for patient benefit: 20 critical questions on transparency, replicability, ethics, and effectiveness. BMJ *368*, l6927.

Wakabayashi, S. (2013). New insights into the functions of histidine-rich glycoprotein. In International Review of Cell and Molecular Biology, K.W. Jeon, ed. (Academic Press), pp. 467–493.

Wermuth, P.J., and Jimenez, S.A. (2015). The significance of macrophage polarization subtypes for animal models of tissue fibrosis and human fibrotic diseases. Clin. Transl. Med. *4*, 2.

Wu, D., Koganti, R., Lambe, U.P., Yadavalli, T., Nandi, S.S., and Shukla, D. (2020). Vaccines and therapies in development for SARS-CoV-2 infections. J. Clin. Med. *9*, 1885.

Wu, Y., Potempa, L.A., El Kebir, D., and Filep, J.G. (2015). C-reactive protein and inflammation: conformational changes affect function. Biol. Chem. *396*, 1181–1197.

Wynants, L., Van Calster, B., Collins, G.S., Riley, R.D., Heinze, G., Schuit, E., Bonten, M.M.J., Dahly, D.L., Damen, J.A.A., Debray, T.P.A., et al. (2020). Prediction models for diagnosis and prognosis of covid-19 infection: systematic review and critical appraisal. BMJ *369*, m1328.

Yang, L., Liu, S., Liu, J., Zhang, Z., Wan, X., Huang, B., Chen, Y., and Zhang, Y. (2020). COVID-19: immunopathogenesis and immunotherapeutics. Signal Transduct. Target. Ther. *5*, 128.

Zhang, H., Penninger, J.M., Li, Y., Zhong, N., and Slutsky, A.S. (2020). Angiotensin-converting enzyme 2 (ACE2) as a SARS-CoV-2 receptor: molecular mechanisms and potential therapeutic target. Intensive Care Med *46*, 586–590.

Zhao, B., Ni, C., Gao, R., Wang, Y., Yang, L., Wei, J., Lv, T., Liang, J., Zhang, Q., Xu, W., et al. (2020). Recapitulation of SARS-CoV-2 infection and cholangiocyte damage with human liver ductal organoids. Protein Cell *11*, 771–775.

Zhuo, L., Itano, N., Nonogaki, T., Shen, L., Wu, J., Watanabe, H., and Kimata, K. (2004). Chapter 9 - Biological Function of SHAP–Hyaluronan Covalent Complex. Chemistry and Biology of Hyaluronan, 205–222. https://doi.org/10.1016/B978-008044382-9/50040-6.

STAR★METHODS

KEY RESOURCES TABLE

REAGENT or RESOURCE	SOURCE	IDENTIFIER
**Biological samples**		
Human Serum	Sigma-Aldrich	Cat# S7023-50MB
Human Plasma (EDTA, Pooled Donor)	Genetex	Cat# GTX73265
**Chemicals, peptides, and recombinant proteins**		
Water for chromatography (LC-MS Grade) LiChrosolv®	Merck	Cat# 115333
Acetonitrile (Acetonitrile, Optima™ LC/MS Grade, Fisher Chemical™)	Fisher Scientific	Cat# A955-212
Methanol (Optima LC-MS Grade, Fisher Chemical)	Fisher Scientific	Cat# A456-212
DL-Dithiothreitol (BioUltra)	Sigma-Aldrich	Cat# 43815
Iodoacetamide (BioUltra)	Sigma-Aldrich	Cat# I1149
Ammonium Bicarbonate (Eluent additive for LC-MS)	Sigma-Aldrich	Cat# 40867
Urea (puriss. P.a., reag. Ph. Eur.)	Honeywell Research Chemicals	Cat# 33247H
Formic Acid, LC-MS Grade (Eluent additive for LC-MS)	Thermo Scientific™ Pierce™	Cat# 85178
Trypsin (Sequence grade)	Promega	Cat# V511X
Mass Spec-Compatible Human Extract	Promega	Cat# V6951
Retention time peptides Biognosys iRT kit	Biognosys	Cat# Ki-30002-b
MS synthetic peptide calibration kit	SCIEX	Cat# 5045759
**Deposited Data**		
Raw mass spectrometry proteomics data (commercial plasma and serum control samples)	This study	PXD025752
**Software and algorithms**		
Proteomics data analysis via Deep Neural Networks, DIA-NN	Demichev et al., 2020	https://github.com/vdemichev/DiaNN
DIA-NN R package	Demichev et al., 2020	https://github.com/vdemichev/diann-rpackage
ComplexHeatmap R package	(Gu et al., 2016)	https://github.com/jokergoo/ComplexHeatmap
EnvStats R package	(Millard, 2014)	https://CRAN.R-project.org/package=EnvStats
Limma R package	(Ritchie et al., 2015)	https://bioconductor.org/packages/limma/
eBayes R package	(Smyth, 2004)	https://github.com/cran/limma/blob/master/R/ebayes.R
XGBoost 1.2.0 Python package	(Chen and Guestrin, 2016)	https://pypi.org/project/xgboost/1.2.0/
scikit-learn 0.23.2 Python package	(Pedregosa et al., 2011)	https://scikit-learn.org/0.23/
scipy 1.5.2 Python package	(Virtanen et al., 2020)	https://pypi.org/project/scipy/1.5.2/
**Other**		
Zorbax RRHD Eclipse Plus 95A C18, 2.1 x 50mm, 1.8 um, 1200 bar	Agilent	Cat# 959757-902
Infinitylab Poroshell 120 EC-C18, 2.1x50mm 1.9um	Agilent	Cat# 699675-902
BioPureSPE Macro 96-Well, 100mg PROTO 300 C18	The Nest Group, Inc.	HNS S18V-L

RESOURCE AVAILABILITY

Lead contact

Further information and requests for resources and reagents should be directed to and will be fulfilled by the lead contact, Markus Ralser (markus.ralser@charite.de).

Materials availability

This study did not generate new unique reagents.

Data and code availability

- The processed proteomic and clinical source data is available in this paper's supplemental information.
- The raw mass spectrometry proteomics source data for the quality control plasma and serum acquisitions has been deposited to the ProteomeXchange Consortium via the PRIDE partner repository (Perez-Riverol et al., 2019) with the dataset identifier PXD025752.
- This paper does not report original code.
- The machine learning scripts used to generate the figures reported in this paper are available in this paper's supplemental information.
- Any additional information required to reproduce this work is available from the Lead Contact.

Experimental model and subject details

Charité patient cohort and clinical data

Patients were recruited within the Pa-COVID-19 study conducted at Charité - Universitätsmedizin Berlin, a prospective observational cohort study on the pathophysiology of COVID-19. The study protocol has been described in detail before (Kurth et al., 2020). All patients with PCR-confirmed SARS-CoV-2 infection were eligible for inclusion. Refusal to provide informed consent by the patient or a legal representative and any condition prohibiting supplemental blood collection for serial biosampling were exclusion criteria. Patients were treated according to current national and international guidelines. Three patients had *Do Not Intubate and Do Not Resuscitate* (DNI/DNR) orders in place, declining mechanical ventilation and other organ support or cardiopulmonary resuscitation. In 4 further cases, limitation of therapy was decided at a later time point according to the patient's presumed wish ("secondary DNR") and predictably unfavorable outcome. All other patients received maximum intensive care treatment including organ replacement therapies at the discretion of the responsible physicians.

Biosampling for proteome measurement was performed 3 times per week after inclusion. The WHO ordinal scale for clinical improvement (Table S1) was used to assess disease severity. ARDS was defined according to the Berlin ARDS criteria (ARDS Definition Task Force et al., 2012). Sepsis was defined according to sepsis-3 criteria (Singer et al., 2016). The study was approved by the ethics committee of Charité - Universitätsmedizin Berlin (EA2/066/20) and conducted in accordance with the Declaration of Helsinki and guidelines of Good Clinical Practice (ICH 1996). The study is registered in the German and the WHO international registry for clinical studies (DRKS00021688). Clinical data was captured in a purpose built electronic case report form data using the capture system SecuTrial®. All routine laboratory parameters were analyzed in accredited laboratories at Charité - Universitätsmedizin Berlin. Pseudonymized data exported from SecuTrial® were processed using JMP Pro 14 (SAS Institute Inc., Cary, NC, USA). If a laboratory value was missing for a given day, values from up to two preceding days were used for the analysis.

Innsbruck Patient cohort and clinical data

Serum samples from 99 patients admitted to the intensive care unit at the Department of Medicine, University Hospital of Innsbruck for the treatment of respiratory failure due to severe COVID-19 were collected within the first days (median 7.5, IQR 5-12) after admission. Written informed consent was either obtained before sampling or retrospectively after recovery, if patients were mechanically ventilated at the time of sampling. COVID-19 was diagnosed on the basis of a (i) positive SARS-CoV2 PCR within the last 7 days prior to study inclusion, (ii) respiratory failure defined as a partial pressure of oxygen < 60 mmHg on arterial blood gas analysis or a peripheral oxygen saturation of < 90% and (iii) typical infiltrates on computed tomography scanning of the chest. Patients were treated according to national guidelines. The study was approved by the local ethics research committee EK-Nr. 1107/2020, and EK-Nr. 1103/2020 for follow-up.

METHOD DETAILS

Materials

Water for chromatography (LC-MS Grade, LiChrosolv®, Merck; 115333), Acetonitrile (LC-MS Grade Optima; A955-212) and Methanol (LC-MS Grade, Optima; A456-212) were purchased from Fisher Chemicals. DL-Dithiothreitol (BioUltra, 43815), Iodoacetamide (BioUltra, I1149) and Ammonium Bicarbonate (Eluent additive for LC-MS, 40867) were purchased from Sigma Aldrich. Urea (puriss. P.a., reag. Ph. Eur., 33247H) and Formic Acid (Eluent additive for LC-MS, 85178) were purchased from Thermo Scientific. Trypsin

(Sequence grade, V511X) was purchased from Promega. Control samples were prepared from Human Serum (Sigma Aldrich, S7023-50MB) and Human Plasma (EDTA, Pooled Donor, Genetex GTX73265).

Mass spectrometry

Mass spectrometry-based proteomics analysis was performed as described previously (Messner et al., 2020) with minor adjustments to the workflow (Figure S1). Semi-automated sample preparation was performed in 96-well format, using in advance prepared stock solution plates stored at -80°C. Briefly, 5μl of thawed plasma samples were transferred to the pre-made denaturation/reduction stock solution plates (55μl 8M Urea, 100mM ammonium bicarbonate (ABC), 50mM dithiothreitol) resuspended and incubated at 30°C for 60 minutes. 5μl was then transferred from the iodoacetamide stock solution plate (100mM) to the sample plate and incubated in the dark at 23°C for 30 minutes before dilution with 100mM ABC buffer (340μl). 220μl of this solution was transferred to the pre-made trypsin stock solution plate (12.5μl, 0.1μg/μl) and incubated at 37°C for 17 h (Benchmark Scientific Incu-Mixer MP4). The digestion was quenched by addition of formic acid (10% v/v, 25μl). The digestion mixture was cleaned-up using C18 96-well plates (BioPureSPE Macro 96-Well, 100mg PROTO C18, The Nest Group) and redissolved in 60μl 0.1% formic acid with shaking. Insoluble particles were removed by centrifugation and the samples transferred to a new plate.

Each 96-well plate contained 8 plasma and 4 serum sample preparation controls, and the acquisition workflow included a pooled quality control sample every ~10 injections. Liquid chromatography was performed using the Agilent 1290 Infinity II system coupled to a TripleTOF 6600 mass spectrometer (SCIEX) equipped with IonDrive Turbo V Source (Sciex). A total of 5μl was injected, and the peptides were separated in reversed phase mode using a C18 ZORBAX Rapid Resolution High Definition (RRHD) column 2.1mm x 50mm, 1.8μm particles or Infinitylab Poroshell 120 EC-C18, 2.1 x 50mm 1.9 μm particles. A linear gradient was applied which ramps from 1% B to 40% B in 5 minutes (Buffer A: 0.1% FA; Buffer B: ACN/0.1% FA) with a flow rate of 800μl/min. For washing the column, the organic solvent was increased to 80% B in 0.5 minutes and was kept for 0.2 minutes at this composition before going back to 1% B in 0.1 min. The mass spectrometer was operated in the high sensitivity mode. The DIA/SWATH method consisted of an MS1 scan from m/z 100 to m/z 1500 (20 ms accumulation time) and 25 MS2 scans (25ms accumulation time) with variable precursor isolation width covering the mass range from m/z 450 to m/z 850 (Messner et al., 2020). An IonDrive Turbo V Source (Sciex) was used with ion source gas 1 (nebulizer gas), ion source gas 2 (heater gas) and curtain gas set to 50, 40 and 25, respectively. The source temperature was set to 450 and the ion spray voltage to 5500V. System suitability was evaluated using synthetic peptides (Sciex 5045759, Biognosys Ki-30002-b) and human protein extracts (Promega V6951).

QUANTIFICATION AND STATISTICAL ANALYSIS

Data analysis

The data were processed with DIA-NN (Demichev et al., 2020), an open-source software suite for DIA / SWATH data processing (https://github.com/vdemichev/DiaNN, commit 4498bd7) using a two-step spectral library refinement procedure as described previously (Messner et al., 2020), with filtering at precursor level q-value (1%), library q-value (0.5%) and gene group q-value (1%). Highly hydrophobic peptides (reference retention time > 110 on the iRT scale) were discarded. Batch correction was performed at the precursor level as described previously (Messner et al., 2020), using linear regression for intra-batch correction (for each MS batch) and control samples for inter-plate correction. Protein quantification was subsequently carried out using the MaxLFQ algorithm (Cox et al., 2014; Pham et al., 2020) as implemented in the DIA-NN R package (https://github.com/vdemichev/diann-rpackage). One of the 96-well plates (#12) featured technical replicates of a number of samples that were also analysed on other plates: in an extra batch correction step, the median $\log_2$-protein levels across these replicates on plate 12 were matched to the respective median $\log_2$-levels (across the same biological samples) throughout other plates, to correct protein levels on plate 12. Further batch correction was performed for Innsbruck data, to match the mean $\log_2$-transformed protein levels in the respective control samples to $\log_2$-transformed protein levels in control samples acquired for the Charité cohort. The Generation Scotland cohort proteomics raw data, which we described previously (Messner et al., 2020), have been reanalyzed using the updated software pipeline, to ensure comparability. Exclusion of precursors or proteins based on the data completeness was not performed.

Statistical testing was performed in the R environment for statistical computing, version 3.6.0 (R core team, www.R-project.org). All protein and clinical laboratory measurements (except for standard and actual base excess, oxyhemoglobin and sO2) were first $\log_2$-transformed, to ensure optimal performance of linear models assuming Gaussian errors, as well as to reduce the impact of outliers. Imputation of the data was not performed, as all the statistical tests applied can accommodate missing values. Likewise, no data filtering based on missing value rates was applied. For differential abundance testing, only protein groups matched to at least three different unmodified peptide sequences were considered. Significance testing for a zero median (for analysing trajectories) or against binary variables (worsening, death) was performed using the Wilcoxon W test or Mann-Whitney U test, respectively, as implemented in the "wilcox.test" function of the "stats" R package. Testing against a continuous variable (e.g. when determining significance of pairwise correlations) was performed using the Kendall Tau test, with the slope estimated using the Theil-Sen method, as implemented in the "kendallTrendTest" function of the "EnvStats" (Millard, 2014) package. When covariates had to be taken into account, we used linear modelling with the "limma" (Ritchie et al., 2015) R package, with P-values obtained using "eBayes" (Smyth, 2004). Modelling with "limma" was likewise used to correct for these covariates for visualisation purposes. WHO grade was considered as a "factor-type" covariate (resulting in a "limma" design matrix with one-hot encoding for different WHO grades). Multiple-testing correction was performed using the Benjamini-Hochberg false discovery rate controlling procedure (Benjamini and Hochberg, 1995)

as implemented in the "p.adjust" function of the "stats" R package. The adjusted p-values below 0.05 were considered significant. Multiple-testing correction for differential abundance analysis was performed separately for proteins, for which MRMAssayDB lists a targeted assay (Bhowmick et al., 2018), the rest of proteins measured, the clinical laboratory measurements and the clinical factors (age, Charlson score, BMI, Horowitz index and FiO_2, SOFA score), to ensure that the false discovery rate stayed below 0.05 for each of these categories of features. Likewise, when determining the significance of correlations in correlation matrices, correction was performed for each row or each column separately, to ensure less than 5% false discoveries in each row or column, respectively. For correlation map visualisations, black points were used to indicate row-wise significant correlations, and black rectangles at the border of the respective cell - column-wise significant correlations.

Quantities of gene products corresponding to open reading frames named IGxx (i.e. different types of immunoglobulin chains) were summed together to generate quantities representative of the overall levels of immunoglobulin classes (IGHVs, IGLVs, etc). This does not affect any conclusions of this work and was done purely to improve visualization and simplify the interpretation of the heatmaps and correlation maps. Full protein level tables, including levels of individual immunoglobulin gene products, are provided in supplementary materials. For visualisation, different WHO grades were color-coded throughout the manuscript (see Figure S2).

Markers of the disease severity

The first time point measured at the maximum WHO grade was chosen for each patient. For each omics feature, its values (log_2-transformed when necessary, as described above) were tested for a trend depending on the WHO grade. Age was included as a covariate in the linear model as described above.

Markers varying with age in COVID-19

The first sampling time point measured was chosen per patient. For each omics feature, its values (log_2-transformed when necessary, as described above) were tested for a trend depending on age. The test was performed either using the Kendall Tau test (as described above; Figures S9 and S10), or by accounting for WHO grade as a covariate in the linear model (as described above; Figures S11 and 5).

Markers of RRT and ECMO

For each omics feature, the P-value was calculated using the Mann-Whitney test, comparing between the median levels (log_2-transformed when necessary, as described above) across all sampling time points at WHO grade 7 in patients who did not receive the therapy and the median levels (log_2-transformed when necessary, as described above) across all sampling time points at WHO grade 7 after initiation of the respective therapy in patients who did.

Markers predictive of time in hospital

Patients, for which the first sampling time point before the outcome corresponded to the WHO = 3 severity grade (that is the patient did not require supplemental oxygen on that day), were considered. Thus, no correction for disease severity was necessary. Testing of levels (log_2-transformed when necessary, as described above) of each omics feature (measured for the first sampling time point) vs the remaining time in hospital (days) was performed by including age as a covariate in the linear model as described above.

Markers predictive of disease worsening

The first sampling time point measured was chosen per patient. Future disease worsening was defined as a future increase in the WHO grade (for patients at WHO grade < 7) or death (for patients at WHO grade 7). For each omics feature, its levels (log_2-transformed when necessary, as described above) were compared between patients who did not worsen and patients who did, with age and current WHO grade (as factor) included as covariates in the linear model as described above.

Peak period of the disease definition

When studying the dynamic changes in omics values during the disease course, we focused on the time points sampled when the disease was the most severe for a particular patient. This allowed us to look at molecular changes over time without the need to take into account the potential impact of changes in disease severity and the level of treatment. For each patient, we thus defined the "peak period of the disease" as the time when the patient was receiving the most intensive treatment during their stay in hospital, that is the time when the patient was at WHO grade 6 or 7, for patients who received invasive mechanical ventilation at some point, or otherwise at their maximum WHO grade (3, 4 or 5).

Markers changing during the peak of disease

Only patients with at least two days between the first and last sampling time points at the peak of the disease (as defined above) were considered. For each omics feature, a linear regression model was fitted for its levels (log_2-transformed when necessary, as described above) vs the day number (with the slope estimated using the nonparametric Theil-Sen method, as implemented in the "kendallTrendTest" function of the "EnvStats" (Millard, 2014) R package), and the quantity $slope_{adj}$ = (regression slope) * (number of days between first and last time points) was calculated. A non-parametric approach was chosen because of its superior robustness to outliers. A Wilcoxon W test was then applied to compare the median of $slope_{adj}$ to zero. The values of $slope_{adj}$ for each feature are visualised in Figure S14. The non-parametric approach was chosen here due to its robustness with respect to outliers.

CellPress
OPEN ACCESS

Correlation maps

General correlation maps were generated using the values (log_2-transformed when necessary, as described above) of features at the first time point measured at the maximum WHO grade for each patient. The correlation map between feature changes during the peak of the disease (as defined above) was generated by correlating the $slope_{adj}$ values (as defined above). The map of significant protein correlations not detected in the general population was generated by excluding all correlations which were either significant ($P <= 0.05$, without multiple-testing correction) with the same trend in the Generation Scotland cohort, or could not be calculated reliably therein (less than 20 valid points).

Prediction of current mechanical ventilation

To reflect the power of omics measurements in characterising the phenotype, a classifier was constructed to predict mechanical ventilation (WHO grade > 5) at the present time point using the proteomic and/or accredited diagnostic data. For the proteomic data only proteins characterized by at least 3 peptides were taken into account. The first time point measured at the maximum WHO grade was selected per patient. We used a gradient boosted tree algorithm implemented in the XGBoost 1.2.0 (Chen and Guestrin, 2016) under Python 3.8.1. The classifier was constructed using leave-one-out cross-validation. To circumvent overfitting a subsampling of 0.5 of the training data per boosting step and an L2 regularization term ‘‘lambda’’ of 20 were applied.

For the assessment of classifier performance, the leave-one-out method was applied in the following way: the prediction was made for each sample separately, by excluding (withholding) this sample from the dataset, training the classifier on the remaining (independent) samples and then predicting the withheld sample using the trained model. The source code is provided in supplementary materials. For the determination of the feature importances, one classifier was trained on all data points using the same setup as described above. The feature importances were then extracted directly from the trained classifier.

For the validation of the trained models, samples from an independent cohort (Innsbruck) were used. A model was trained on the data collected at the Charité using the same setup and parameters as described above and the proteins that were characterized in both cohorts. The evaluation was performed on the Innsbruck cohort that was not used for training. ROC-curves and AUC were calculated using scikit-learn 0.23.2 (Pedregosa et al., 2011). The machine learning scripts are provided in Data S1.

WHO grade prediction

For the prediction of the WHO grade an elastic net was applied as implemented in scikit-learn 0.23.2. The WHO grade was predicted for the first time point at maximum WHO grade per patient using a leave-one-out cross-validation procedure. A training/prediction based on proteomic (proteins with at least 3 peptides) and/or accredited diagnostic data from the Charité cohort was performed. Additionally, the proteomic model was validated using proteomic data set from the Innsbruck cohort that was not included in the training. Features with more than 10% missing values were removed. All data were log_2-transformed when necessary (as described above), standardized and kNN-imputed (5 neighbors). The latter two steps were fitted on the training data only. For the elastic net an ‘‘l1_ratio’’ of 0.05 was used coupled to a 5-fold cross-validated recursive feature-elimination algorithm (‘‘step’’ = 10, ‘‘min_features’’ = 20). Calculations of metrics were performed using scikit-learn 0.23.2 and scipy 1.5.2 (Virtanen et al., 2020). The machine learning scripts are provided in Data S1.

Prediction of the remaining time in hospital

For the prediction of the remaining time in hospital a WHO grade predictor as described above was trained on the first data points for every patient. The predicted WHO grades for every patient at WHO grade 3 who stayed in hospital for at least 1 day after sample time were correlated to the remaining time in hospital. The Spearman correlation was calculated using scipy 1.5.2. The machine learning scripts are provided in Data S1.

Supplementary Note 1. Diagnostic parameters and Proteome signatures that indicate therapeutic interventions

We investigated to what extent specific organ replacement therapies in severely ill patients, (renal replacement therapy (RRT) and extracorporeal membrane oxygenation (ECMO)) were reflected in the proteome and at the level of accredited diagnostic parameters. HP and HPX were reduced in patients on RRT and ECMO, reflecting hemolysis in the extracorporeal circuits (Figures S7 and S8). Elevated SERPINC1 (Antithrombin III) levels mirror substitution of antithrombin during ECMO. The reason for elevated levels of APOE in patients with ECMO is unclear, but is in line with reports on increased levels of APOE in pediatric patients after cardiopulmonary bypass (Aĝirbaşli et al., 2015). The proteins increased in patients receiving RRT mainly reflect impaired kidney function and have been associated with RRT before (AMBP, B2M, CST3, LYZ, RBP4, Figure S7) (Shao et al., 2015). Of note, increased levels of AMBP, B2M and LYZ have been associated with death in chronic kidney disease (Makridakis et al., 2020). Levels of CFD and APOH, both involved in the complement system, were also increased (McDonnell et al., 2020; Volanakis and Narayana, 1996). CFD is eliminated renally and accumulates in end stage renal disease, possibly leading to enhanced complement activation via the alternative pathway (Pascual et al., 1988). In contrast, levels of APOH have even been reported to be slightly lower following high-flux hemodialysis (Han et al., 2018).

We note that the analysis of the effect of treatments on the proteome has two limitations. First, some of the markers identified might be prognostic for the treatment rather than reflect its effect. Age and the Charlson comorbidity index belong to this category: patients receiving ECMO were significantly younger and had a lower number of pre-existing chronic conditions than those who did not.

Second, the results might be partially confounded by the time elapsed from the onset of the disease, as we have shown (Figure 3) that omics signature changes with time in COVID-19 patients while on invasive mechanical ventilation.

Supplementary Note 2. Age-specific response to COVID-19 in the context of severity markers

Older age is one of the most significant risk factors for severe disease and adverse outcome in COVID-19. Enhanced understanding of underlying mechanisms for the age-specific response to SARS-CoV-2 infection is therefore important and needed for the development of effective age-specific strategies for prevention and treatment. Furthermore, dissecting the age-specific components of the host response will improve our knowledge of the pathogenicity of similar viruses, making the world better prepared for future pandemics. Current theories characterizing the link between the higher age and risk for severe disease include immunosenescence, elevated baseline inflammation, or altered protein glycosylation landscape leading to impaired antiviral response or reduced immune tolerance (Franceschi et al., 2018; Goronzy and Weyand, 2013; Rea et al., 2018; Tay et al., 2020). However, a detailed and mechanistic understanding of the relation between COVID-19 and aging is lacking. In this work, we leverage the large size and high precision of the proteomic data acquired to map the age-related response to COVID-19, to provide a reference dataset (Figures 2C, 5, and S11) for future studies addressing this problem.

We report elevation of several inflammatory and acute phase proteins such as SERPINA3, ITIH4, SAA1, and ITIH3 in older patients with COVID-19. SAA1 has been shown to induce macrophage polarization to the M2-type which promotes tissue repair but also possesses pro-fibrotic properties involved in the pathogenesis of pulmonary fibrosis (Liu et al., 2014; Page et al., 2012; Wermuth and Jimenez, 2015). Moreover, SAA1 mediates displacement of APOA1 from HDL leading to loss of the cardio- and vasoprotective properties of high density lipoprotein (HDL) (Gordon, 2014). SERPINA3, as discussed above, has an ambivalent role as a neutrophil proteinase inhibitor but also a powerful neutrophil chemoattractant. Upregulation of SERPINA3 with age in COVID-19, along with the higher neutrophil-to-lymphocyte ratio, suggests that excessive neutrophil response is one of the aggravating factors in older COVID-19 patients. Taken together, our findings point toward a disproportionately dysregulated inflammatory response to SARS-CoV-2 with age, which may be explained by an increased baseline inflammation and immunosenescence in older patients (Chung et al., 2019; Ferrucci et al., 2005; Soysal et al., 2016). Age-dependent increase of FBLN1 and decrease of KLKB1 reflect alterations in blood coagulation which may aggravate this effect by predisposing older patients to thromboembolic events, one of the key clinical characteristics of severe COVID-19.

Interestingly, a number of apolipoproteins displayed a strong age-specific signature in COVID-19. For instance, APOC2, a component of chylomicrons, very low density lipoprotein (VLDL) and high density lipoprotein (HDL), and activator of lipoprotein lipase involved in triglyceride metabolism (Ramasamy, 2014), was downregulated with age in COVID-19, but upregulated with age in the general population (Harris et al., 2017; Peters et al., 2015) (Figure 2C). Dysregulation of apolipoproteins has been observed in community acquired pneumonia and associated with unfavourable outcome (Sharma et al., 2017). Remarkably, contrary to the general trend, APOD, APOC3 and APOE show opposite trends in older COVID-19 patients and in severe disease (Figure 5). APOD is expressed by many tissues, including the brain (Dassati et al., 2014). An increase in APOD has been previously observed in ischemic stroke and CNS inflammation and may reflect (subclinical) involvement of the central nervous system especially in older patients with more severe inflammation and more comorbidities (Muffat and Walker, 2010). Conversely, high levels of APOD have been shown to temper coronavirus-mediated encephalitis in mice, indicating its role as a marker of CNS damage as well as tissue protection and repair (Carmo et al., 2008). APOE, involved in inflammation, immune response and lipid metabolism, is upregulated in severe COVID-19 but downregulated with age in this cohort. APOE typically mediates anti-inflammatory effects by downregulation of NFκB and inhibition of macrophage response to IFNγ and TLR3, both mediators of viral immune response. Moreover, it neutralizes bacterial LPS and enhances the adaptive immune response by facilitating antigen presentation (Figueroa et al., 2019). Downregulation with age may reflect a compromised immune response leading to over-activation of NFκB and insufficient pathogen clearance in older patients. Finally, APOE has been described to reduce proliferation of myeloid progenitor cells (Murphy et al., 2011) and to reduce myeloid derived suppressor cell (MDSC) survival in mice (Tavazoie et al., 2018). Thus, lower levels of APOE in the elderly may favor expansion of immature and dysfunctional neutrophils that have been described as a hallmark in severe COVID-19 (Schulte-Schrepping et al., 2020). This broad involvement of APOE merits further investigation in future studies.

Supplementary Note 3. Diverging trends at the proteome level during the disease peak in individual patients

Some patients (59, 90, 96, 123) who died exhibited protein concentration trajectories distinctly similar to "typical" survivors (Figure 3B). Two of them (59, 90) had a prolonged ICU stay with repeated septic episodes and finally defined limitations of therapy according to presumed patients' wishes ("secondary DNR"). Their protein signatures probably reflect the phenomenon of immune paralysis that can follow bacterial sepsis associated with a prolonged ICU treatment (Patricio et al., 2019). One patient (96) was receiving ongoing immunosuppressive therapy for an autoimmune disorder, and a fourth patient (123) had a history of kidney transplantation, both died of septic shock. Whether the particular group of solid organ recipients shows a distinct protein signature associated with the outcome requires further investigation.

We also note that some surviving patients do not show a trajectory characteristic of the typical ‘alleviation’ of the proteomic phenotype (WHO = 4: 58, 106, 153; WHO = 6 or 7: 43, 80). Specifically, the proteomic response in patients 106, 153 and 141 was indicative of overall ‘worsening’ of the proteome (Figure 3B). In contrast, patients 43 and 80 exhibited the overall ‘alleviation’ of the proteome, except for the spike in the levels of CRP and serum amyloid (Figure 3B). Shorter time spans between sampling days may explain these observations in four of these patients (43, 58, 80, 106), indicating that the host inflammatory response requires a certain time to resolve, especially in more severely ill patients, and some of the markers of systemic inflammation might linger, whereas a typical alleviation of the proteomic signature can be observed even within a few days in moderate disease courses. The unusual pattern of patient 153 was likely confounded by a skin infection that subsequently required antibiotic treatment.

Cell Systems, Volume *12*

Supplemental information

A time-resolved proteomic

and prognostic map of COVID-19

Vadim Demichev, Pinkus Tober-Lau, Oliver Lemke, Tatiana Nazarenko, Charlotte Thibeault, Harry Whitwell, Annika Röhl, Anja Freiwald, Lukasz Szyrwiel, Daniela Ludwig, Clara Correia-Melo, Simran Kaur Aulakh, Elisa T. Helbig, Paula Stubbemann, Lena J. Lippert, Nana-Maria Grüning, Oleg Blyuss, Spyros Vernardis, Matthew White, Christoph B. Messner, Michael Joannidis, Thomas Sonnweber, Sebastian J. Klein, Alex Pizzini, Yvonne Wohlfarter, Sabina Sahanic, Richard Hilbe, Benedikt Schaefer, Sonja Wagner, Mirja Mittermaier, Felix Machleidt, Carmen Garcia, Christoph Ruwwe-Glösenkamp, Tilman Lingscheid, Laure Bosquillon de Jarcy, Miriam S. Stegemann, Moritz Pfeiffer, Linda Jürgens, Sophy Denker, Daniel Zickler, Philipp Enghard, Aleksej Zelezniak, Archie Campbell, Caroline Hayward, David J. Porteous, Riccardo E. Marioni, Alexander Uhrig, Holger Müller-Redetzky, Heinz Zoller, Judith Löffler-Ragg, Markus A. Keller, Ivan Tancevski, John F. Timms, Alexey Zaikin, Stefan Hippenstiel, Michael Ramharter, Martin Witzenrath, Norbert Suttorp, Kathryn Lilley, Michael Mülleder, Leif Erik Sander, PA-COVID-19 Study group, Markus Ralser, and Florian Kurth

Patient state	Descriptor	Score
Uninfected	No clinical or virological evidence of infection	0
Ambulatory	No limitation of activities	1
	Limitation of activities	2
Hospitalised - mild disease	No oxygen therapy	3
	Oxygen by mask or nasal prongs	4
Hospitalised - severe disease	Non-invasive ventilation or high-flow oxygen	5
	Intubation and mechanical ventilation	6
	Ventilation + additional organ support (vasopressors, RRT, ECMO)	7
Dead	Death	8

Supplementary Table 1. **WHO ordinal scale for clinical improvement in COVID-19 as used in the study** (World Health Organisation 2020).

	All patients		No invasive mechanical ventilation						Invasive mechanical ventilation				Deceased**	
	All patients		Max WHO 3 No supplemental oxygen		Max WHO 4 Supplemental low flow oxygen		Max WHO 5 Supplemental high flow oxygen		Max WHO 6 Invasive mechanical ventilation		Max WHO 7 additional organ replacement therapy			
**Number of patients**	139	100%	23	17%	33	24%	14	10%	6	4%	63	45%	17	12%
**Sex**														
Female	44	32%	13	57%	8	24%	3	21%	1	17%	19	30%	3	18%
Male	95	68%	10	43%	25	76%	11	79%	5	83%	44	70%	14	82%
**Age, years** (Median, IQR)	61	50 - 71	49	35 - 70	63	48 - 71	62	49 - 85	64	59 - 71	62	53 - 72	69	55 - 77
≥65	57	41%	6	26%	16	48%	6	43%	3	50%	26	41%	11	65%
**BMI** (kg/m^2, Median, IQR)	27.8	24.7 - 31.9	25.4	23.0 - 30.6	27.2	23.3 - 30.3	27.1	23.3 - 34.9	26.6	24.6 - 28.1	29.4	25.7 - 34.1	29.0	24.8 - 31.0
< 25 kg/m^2	40	29%	10	43%	13	39%	3	21%	2	33%	12	20%	4	24%
≥ 25 kg/m^2	97	71%	13	57%	20	61%	11	79%	4	66%	49	80%	13	76%
**Pre-existing conditions**														
CCI (Median, IQR)	3	1 - 4	1	0 - 3	4	1 - 5	3	1 - 6	2.5	2 - 5	3	1 - 4	3	3 - 6
<3	65	47%	16	70%	13	39%	7	50%	3	50%	26	41%	2	12%
≥3	74	53%	7	30%	20	61%	7	50%	3	50%	37	59%	15	88%
Hypertension	78	56%	6	26%	21	64%	7	50%	3	50%	41	65%	15	88%
Congestive heart failure	7	5%	0	0%	3	9%	0	0%	1	17%	3	5%	1	6%
h/o myocardial infarction	13	9%	1	4%	2	6%	1	7%	1	17%	8	13%	1	6%
Chronic lung disease	22	16%	2	9%	6	18%	1	7%	2	33%	11	17%	3	18%
Asthma	7	5%	1	4%	2	6%	0	0%	0	0%	4	6%	1	6%
COPD	15	11%	1	4%	5	15%	0	0%	1	17%	8	13%	2	12%
on home oxygen therapy	6	4%	0	0%	1	3%	0	0%	1	17%	4	6%	2	12%
Diabetes	31	22%	1	4%	9	27%	4	29%	1	17%	16	25%	3	18%
Type II diabetes (% of diabetes)	30	97%	1	100%	8	89%	4	100%	1	100%	16	100%	3	100%
Oral hypoglycaemic drugs	16	12%	0	0%	5	15%	2	14%	0	0%	9	14%	2	12%
Insulin therapy	11	8%	0	0%	6	18%	2	14%	0	0%	3	5%	0	0%
Chronic kidney disease	19	14%	2	9%	8	24%	3	21%	0	0%	6	10%	2	12%
Requiring RRT	2	2%	0	0%	1	3%	0	0%	0	0%	1	2%	1	6%
Chronic liver disease	8	6%	2	9%	3	9%	1	7%	0	0%	2	3%	1	6%
Lipid metabolism disorder	21	15%	4	17%	9	27%	1	7%	1	17%	6	10%	3	18%
h/o stroke	7	5%	0	0%	1	3%	1	7%	1	17%	4	6%	1	6%
Ischemic (% of stroke)	6	86%	0	0%	1	100%	1	100%	1	100%	3	75%	0	0%
Active malignancy	3	2%	0	0%	0	0%	0	0%	1	17%	2	3%	1	6%
h/o solid organ transplant	3	2%	0	0%	2	6%	0	0%	0	0%	1	2%	1	6%
h/o immunosuppressive therapy within past 3 months	13	9%	2	9%	4	12%	2	14%	0	0%	5	8%	3	18%
h/o smoking	33	24%	6	26%	14	42%	2	14%	2	33%	9	14%	2	12%
Current smoker	5	4%	1	4%	2	6%	0	0%	1	17%	1	2%	0	0%
**Outpatient medication**														
Total amount (Median, IQR)	2	1 - 3	1	0 - 2	3	2 - 5	1	0 - 2	5	2 - 8	2	1 - 4	2	1 - 3
ARB	24	17%	2	9%	6	18%	2	14%	0	0%	14	22%	2	12%
ACE-I	22	22%	3	13%	8	24%	1	7%	0	0%	10	16%	2	12%
Lipid lowering agents	25	25%	2	9%	11	33%	0	0%	1	17%	11	17%	3	18%

**Duration of hospital course* (days)**	20	9 - 48	7	4 - 10	14	9 - 17	20	13 - 28	38	24 - 71	46	32 - 75	28	16 - 46
Proning**	51	38%	-	-	-	-	-	-	2	33%	49	78%	13	76%
Mechanical ventilation**	69	50%	-	-	-	-	-	-	6	100%	63	100%	17	100%
RRT	46	33%	0	0%	1	3%	0	0%	1	17%	44	70%	15	88%
ECMO**	22	16%	-	-	-		-	-	-	-	22	35%	8	47%
ARDS	76	55%	0	0%	3	9%	7	50%	4	67%	62	98%	17	100%
Sepsis	41	29%	0	0%	2	6%	3	21%	2	33%	34	54%	12	71%
Thromboembolic event	32	23%	0	0%	1	3%	2	14%	1	17%	28	44%	8	47%
Cardiopulmonary resuscitation**	7	6%	0	0%	0	0%	0	0%	0	0%	7	12%	2	12%
**Outcome**														
Deceased (incl. DNI/DNR)	20***	14%	0	0%	1	3%	2	14%	0	0%	17	27%	-	-
DNI/DNR	3	2%	0	0%	1	3%	2	14%	0	0%	0	0%	-	-
Secondary DNI/DNR	4	3%	0	0%	0	0%	0	0%	0	0%	4	6%	4	24%
Requiring new oxygen therapy after discharge*	12	10%	0	0%	0	0%	1	10%	0	0%	11	24%	-	-
Requiring new RRT after discharge*	5	4%	0	0%	0	0%	0	0%	0	0%	5	11%	-	-

Data are shown in n (%) unless otherwise indicated. IQR – interquartile range, CCI – Charlson's comorbidity index; CKD – chronic kidney disease; ACE-I – angiotensin-converting enzyme inhibitor, ARB - Angiotensin II receptor blockers, RRT – renal replacement therapy, ECMO – extracorporeal membrane oxygenation, DNI/DNR – do not intubate/do not resuscitate, i.e. patient's wish not to receive mechanical ventilation, organ replacement therapy, or cardiopulmonary resuscitation - secondary DNR - secondary limitation of therapy in situation of probable unfavourable outcome and according to the presumed patient's wish

* deceased patients not included

** patients with DNI/DNR not included

*** one patient died of non-COVID-19 related cause

Supplementary Table 2. **Baseline, treatment and outcome characteristics of patient cohort with COVID-19 at Charité - University hospital Berlin**. Patients are stratified according to the maximum grade on WHO ordinal scale.

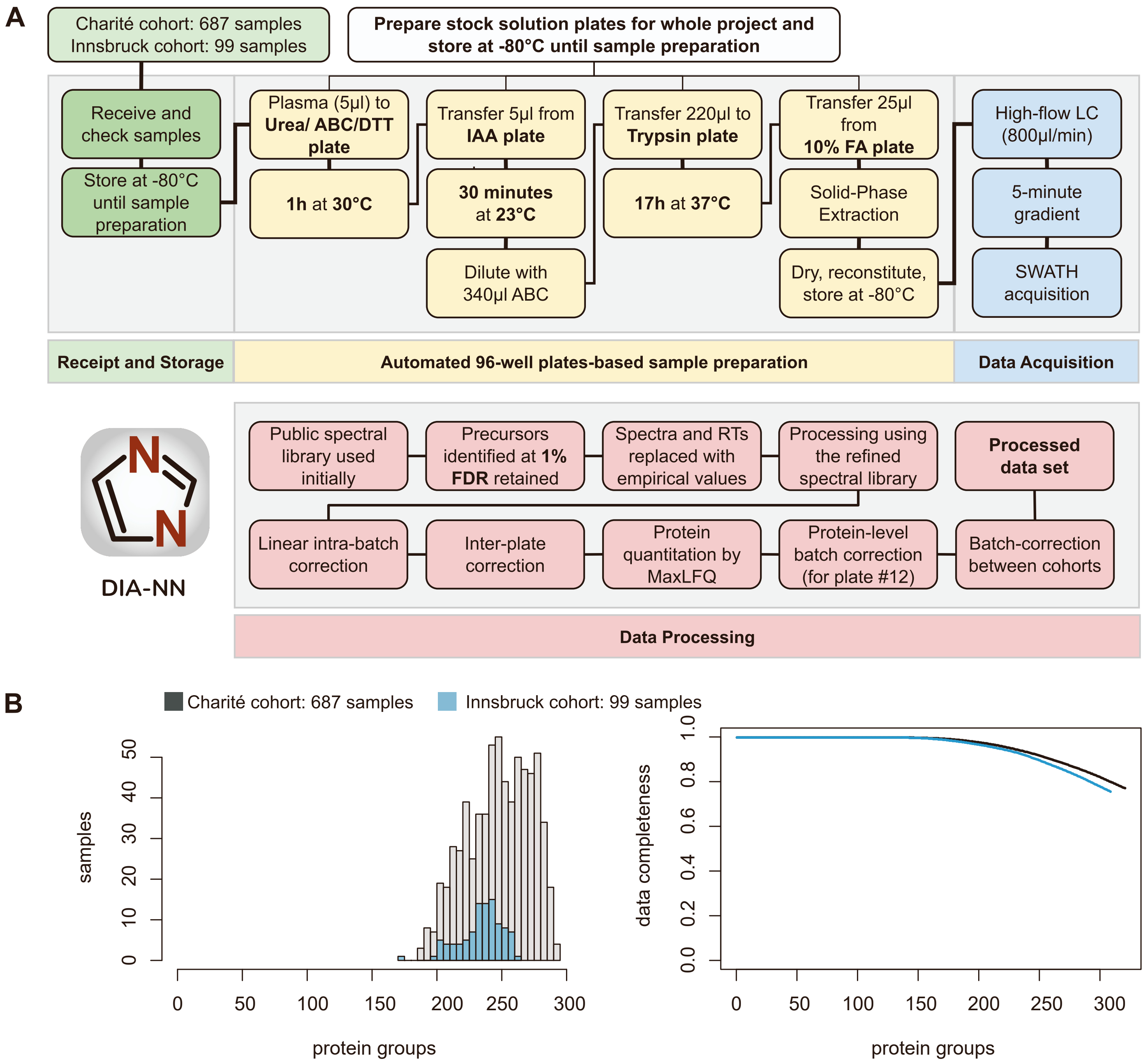

Figure S1. **Plasma proteomics workflow. A** The schematic of the 96-well plates-based sample preparation workflow (Messner et al., 2020), followed by mass spectrometry and raw data processing. The workflow results in normalised and batch-corrected protein quantities. Imputation of missing values is not performed per se, but might be required for certain downstream analyses. All statistical tests in the present work were applied without imputation, while imputation was performed for machine learning (Methods). **B** Distribution of the numbers of quantified protein groups across different samples in two cohorts (left) and the data completeness plotted against the number of protein groups (right).

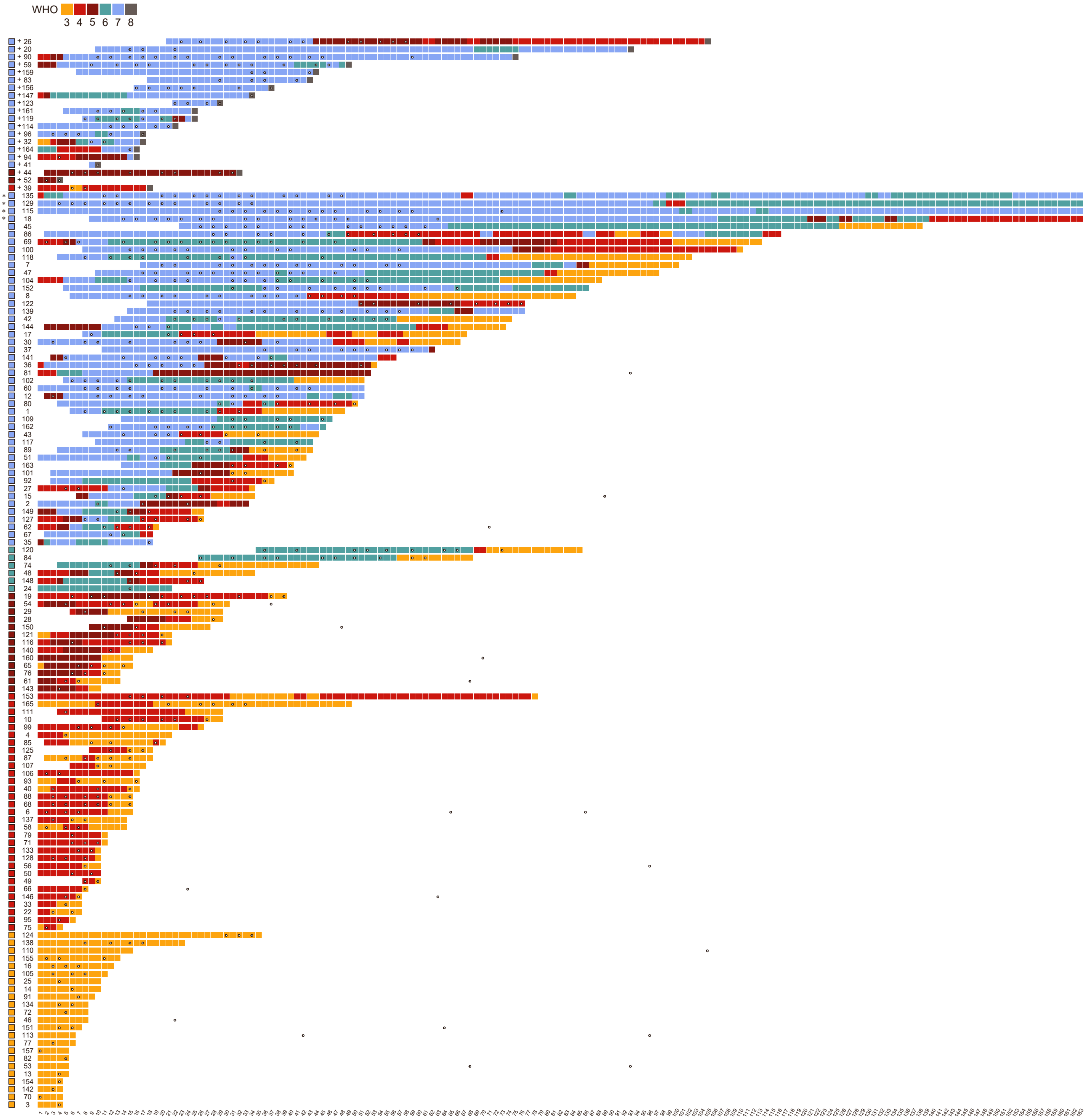

Figure S2. **Disease trajectories from hospital admission onward**. Patient IDs are given on the y-axis, number of days since admission on the x-axis, WHO severity grade is color-coded, starting with the day the patient was admitted to Charite or transferred from another hospital. Proteomic samples (including follow-up visits after discharge) are indicated with white points.

+ deceased

* still in hospital on 30 August 2020

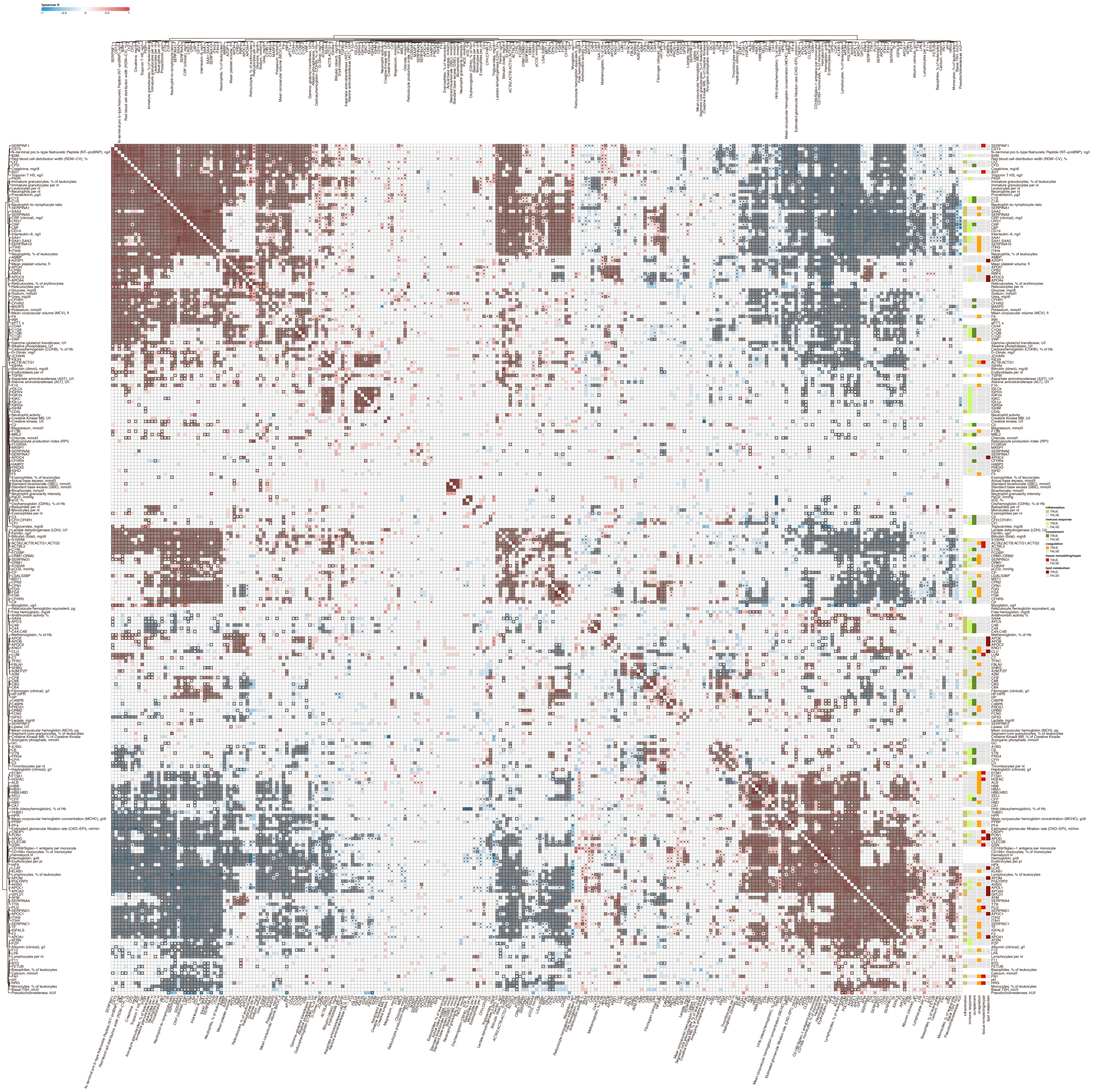

Figure S3. **Map of all correlations between omics measurements**

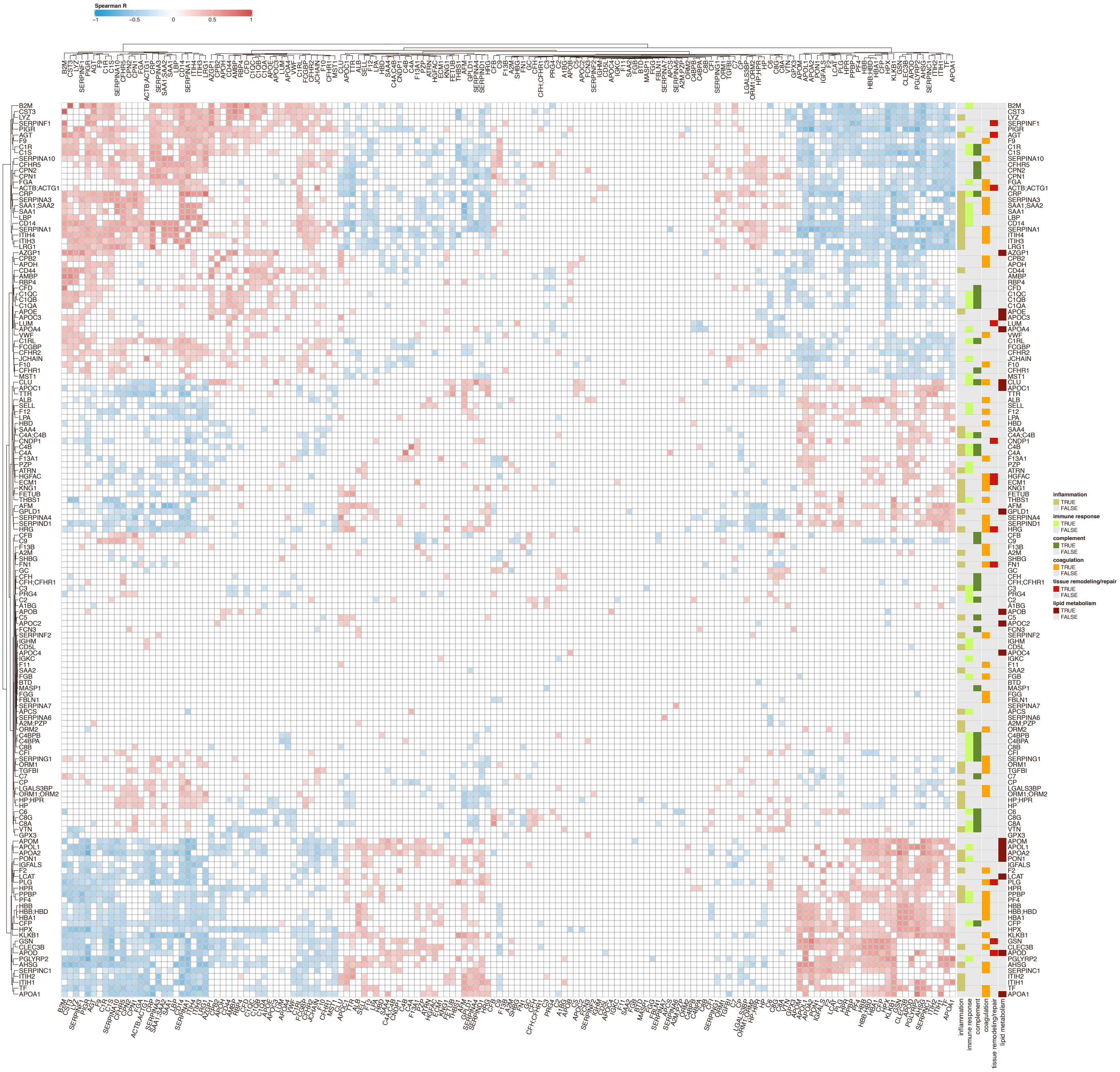

Figure S4. **Map of COVID-19-specific protein-protein correlations**

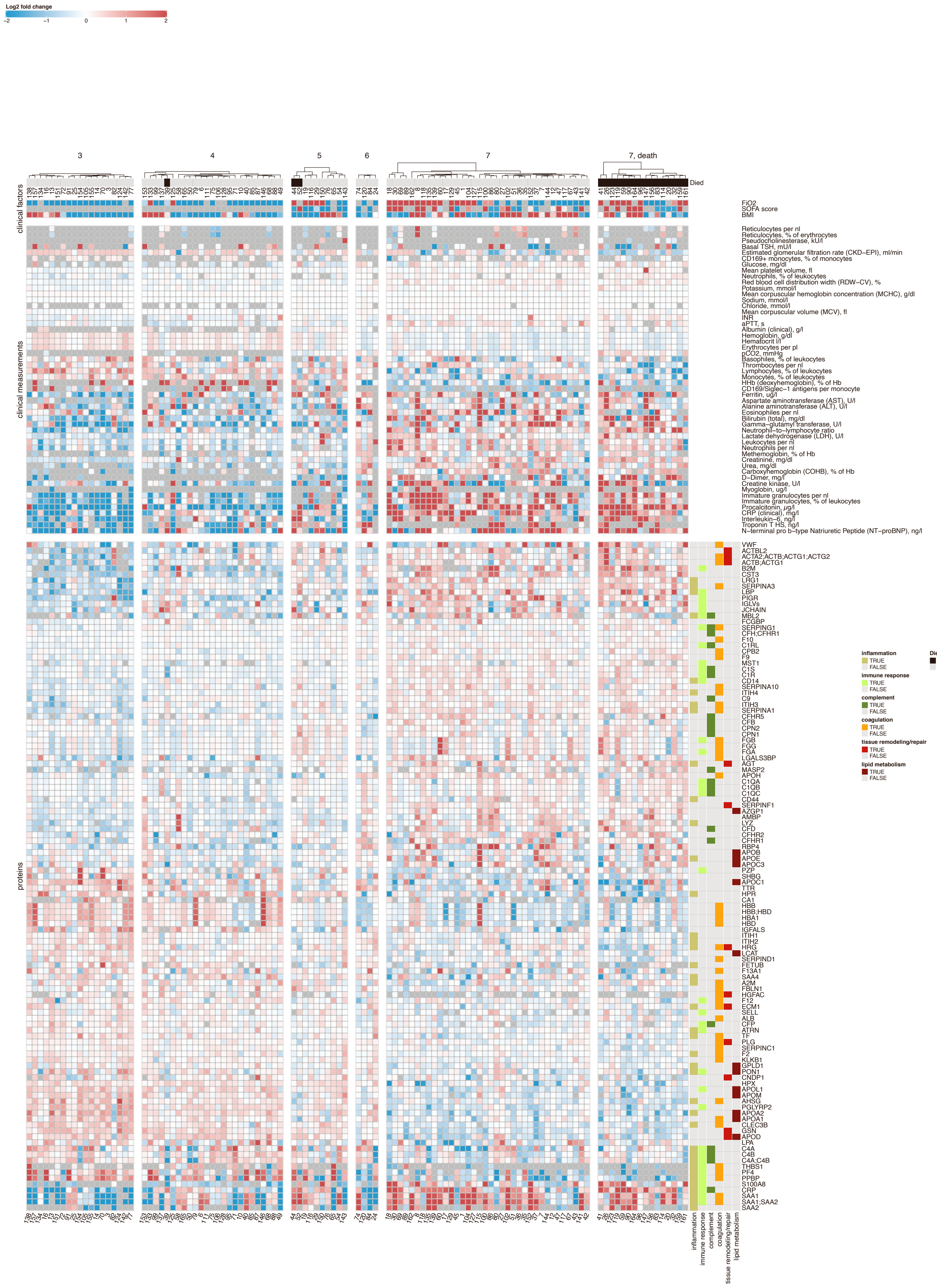

Figure S5. **Map of omics response depending on COVID-19 severity**

Figure S6 (following 5 pages). **Omics features significantly regulated depending on COVID-19 severity**

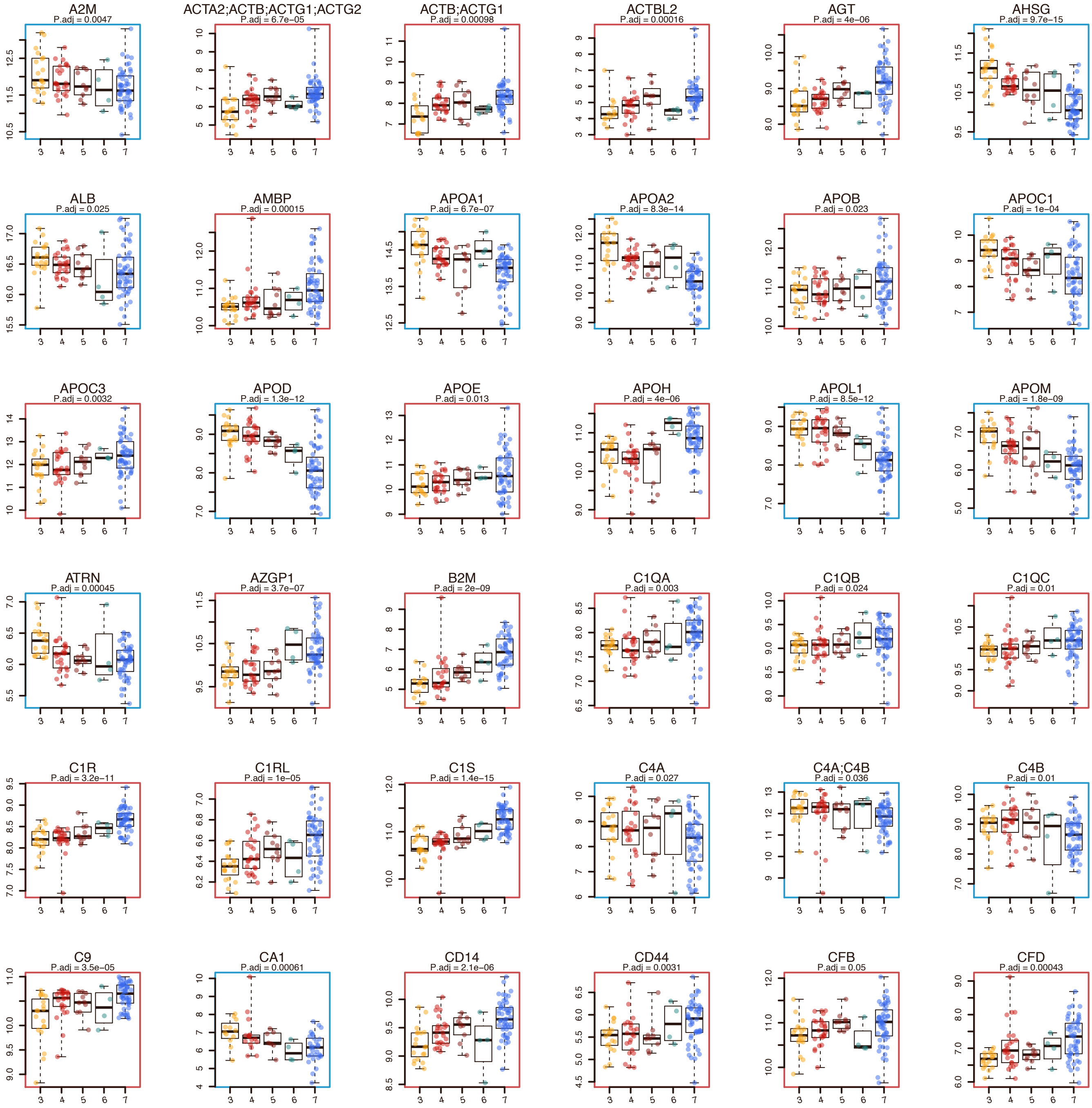

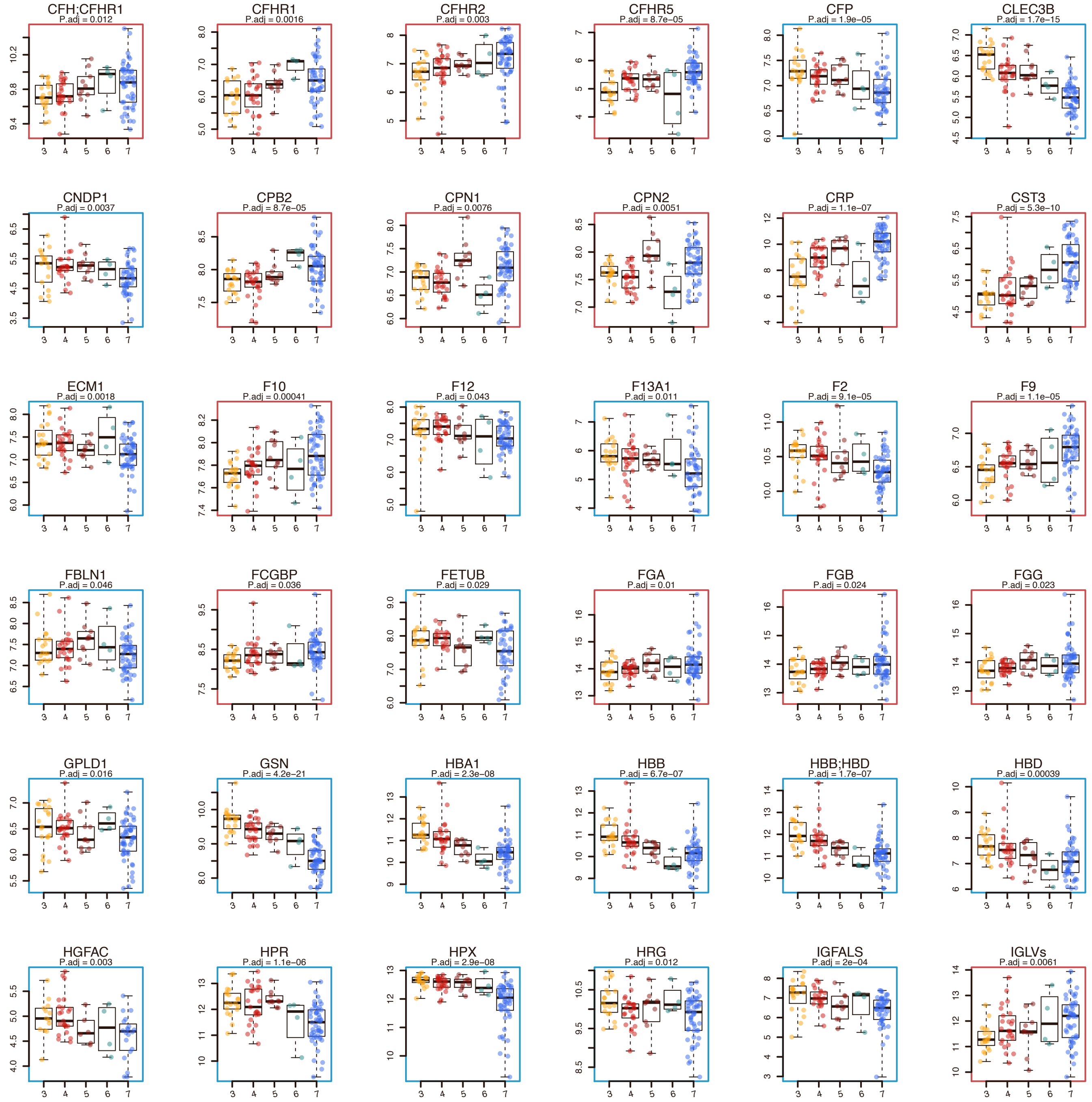
CFH;CFHR1
P.adj = 0.012
CFHR1
P.adj = 0.0016
CFHR2
P.adj = 0.003
CFHR5
P.adj = 8.7e−05
CFP
P.adj = 1.9e−05
CLEC3B
P.adj = 1.7e−15
CNDP1
P.adj = 0.0037
CPB2
P.adj = 8.7e−05
CPN1
P.adj = 0.0076
CPN2
P.adj = 0.0051
CRP
P.adj = 1.1e−07
CST3
P.adj = 5.3e−10
ECM1
P.adj = 0.0018
F10
P.adj = 0.00041
F12
P.adj = 0.043
F13A1
P.adj = 0.011
F2
P.adj = 9.1e−05
F9
P.adj = 1.1e−05
FBLN1
P.adj = 0.046
FCGBP
P.adj = 0.036
FETUB
P.adj = 0.029
FGA
P.adj = 0.01
FGB
P.adj = 0.024
FGG
P.adj = 0.023
GPLD1
P.adj = 0.016
GSN
P.adj = 4.2e−21
HBA1
P.adj = 2.3e−08
HBB
P.adj = 6.7e−07
HBB;HBD
P.adj = 1.7e−07
HBD
P.adj = 0.00039
HGFAC
P.adj = 0.003
HPR
P.adj = 1.1e−06
HPX
P.adj = 2.9e−08
HRG
P.adj = 0.012
IGFALS
P.adj = 2e−04
IGLVs
P.adj = 0.0061
3 4 5 6 7

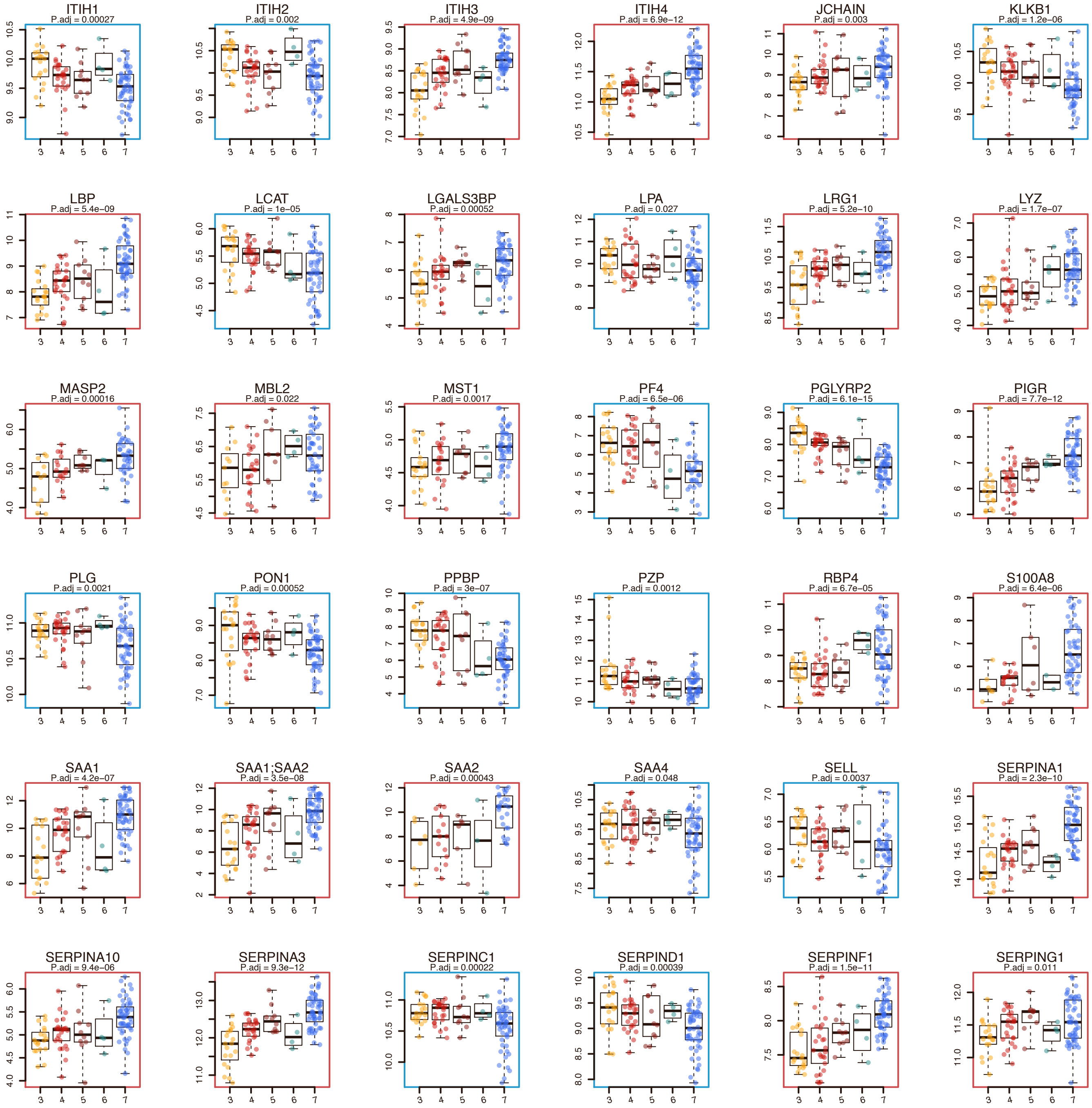
ITIH1
P.adj = 0.00027
ITIH2
P.adj = 0.002
ITIH3
P.adj = 4.9e−09
ITIH4
P.adj = 6.9e−12
JCHAIN
P.adj = 0.003
KLKB1
P.adj = 1.2e−06
LBP
P.adj = 5.4e−09
LCAT
P.adj = 1e−05
LGALS3BP
P.adj = 0.00052
LPA
P.adj = 0.027
LRG1
P.adj = 5.2e−10
LYZ
P.adj = 1.7e−07
MASP2
P.adj = 0.00016
MBL2
P.adj = 0.022
MST1
P.adj = 0.0017
PF4
P.adj = 6.5e−06
PGLYRP2
P.adj = 6.1e−15
PIGR
P.adj = 7.7e−12
PLG
P.adj = 0.0021
PON1
P.adj = 0.00052
PPBP
P.adj = 3e−07
PZP
P.adj = 0.0012
RBP4
P.adj = 6.7e−05
S100A8
P.adj = 6.4e−06
SAA1
P.adj = 4.2e−07
SAA1;SAA2
P.adj = 3.5e−08
SAA2
P.adj = 0.00043
SAA4
P.adj = 0.048
SELL
P.adj = 0.0037
SERPINA1
P.adj = 2.3e−10
SERPINA10
P.adj = 9.4e−06
SERPINA3
P.adj = 9.3e−12
SERPINC1
P.adj = 0.00022
SERPIND1
P.adj = 0.00039
SERPINF1
P.adj = 1.5e−11
SERPING1
P.adj = 0.011

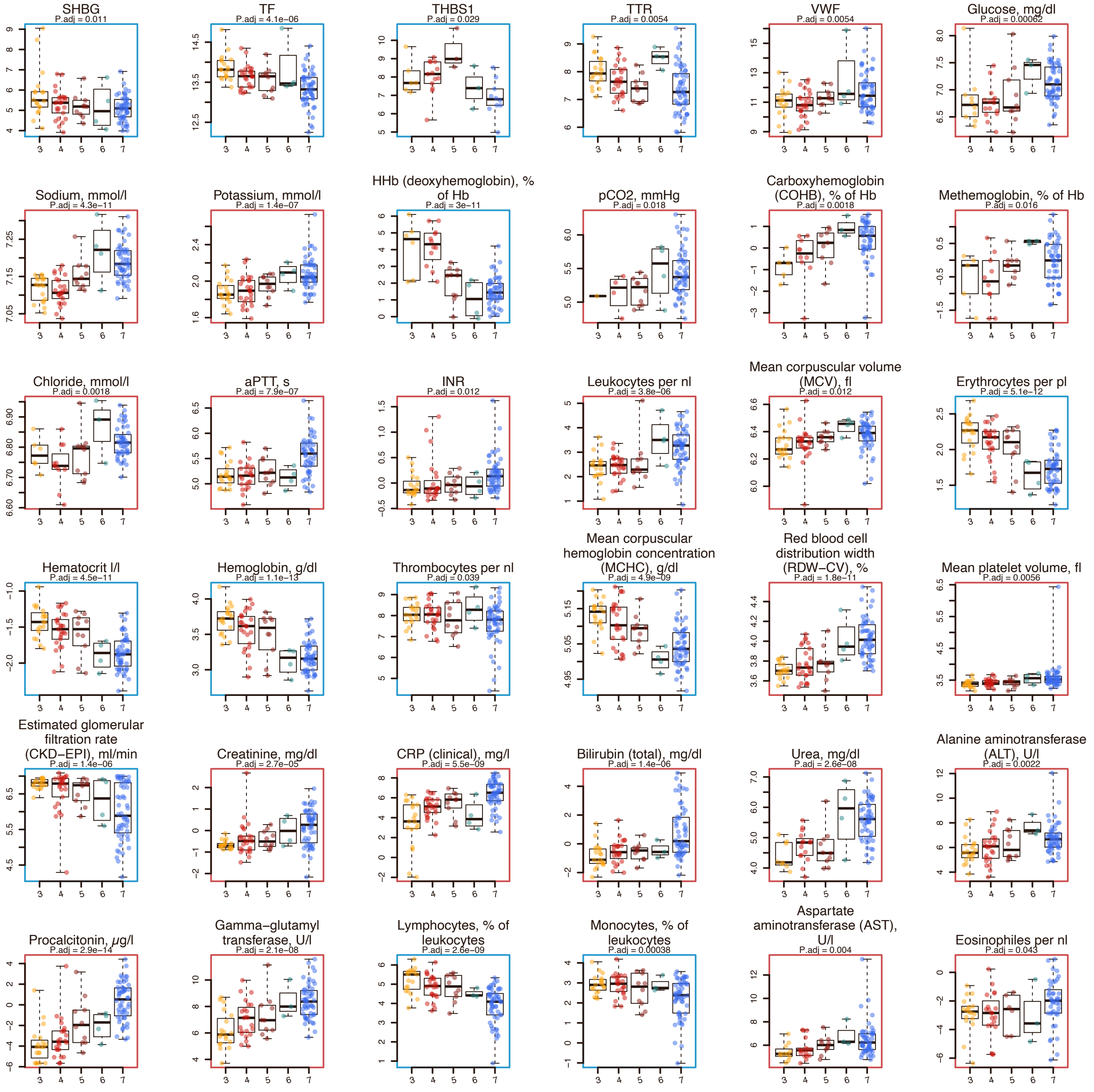
SHBG
P.adj = 0.011
TF
P.adj = 4.1e−06
THBS1
P.adj = 0.029
TTR
P.adj = 0.0054
VWF
P.adj = 0.0054
Glucose, mg/dl
P.adj = 0.00062
Sodium, mmol/l
P.adj = 4.3e−11
Potassium, mmol/l
P.adj = 1.4e−07
HHb (deoxyhemoglobin), % of Hb
P.adj = 3e−11
pCO2, mmHg
P.adj = 0.018
Carboxyhemoglobin (COHB), % of Hb
P.adj = 0.0018
Methemoglobin, % of Hb
P.adj = 0.016
Chloride, mmol/l
P.adj = 0.0018
aPTT, s
P.adj = 7.9e−07
INR
P.adj = 0.012
Leukocytes per nl
P.adj = 3.8e−06
Mean corpuscular volume (MCV), fl
P.adj = 0.012
Erythrocytes per pl
P.adj = 5.1e−12
Hematocrit l/l
P.adj = 4.5e−11
Hemoglobin, g/dl
P.adj = 1.1e−13
Thrombocytes per nl
P.adj = 0.039
Mean corpuscular hemoglobin concentration (MCHC), g/dl
P.adj = 4.9e−09
Red blood cell distribution width (RDW−CV), %
P.adj = 1.8e−11
Mean platelet volume, fl
P.adj = 0.0056
Estimated glomerular filtration rate (CKD−EPI), ml/min
P.adj = 1.4e−06
Creatinine, mg/dl
P.adj = 2.7e−05
CRP (clinical), mg/l
P.adj = 5.5e−09
Bilirubin (total), mg/dl
P.adj = 1.4e−06
Urea, mg/dl
P.adj = 2.6e−08
Alanine aminotransferase (ALT), U/l
P.adj = 0.0022
Procalcitonin, μg/l
P.adj = 2.9e−14
Gamma−glutamyl transferase, U/l
P.adj = 2.1e−08
Lymphocytes, % of leukocytes
P.adj = 2.6e−09
Monocytes, % of leukocytes
P.adj = 0.00038
Aspartate aminotransferase (AST), U/l
P.adj = 0.004
Eosinophiles per nl
P.adj = 0.043

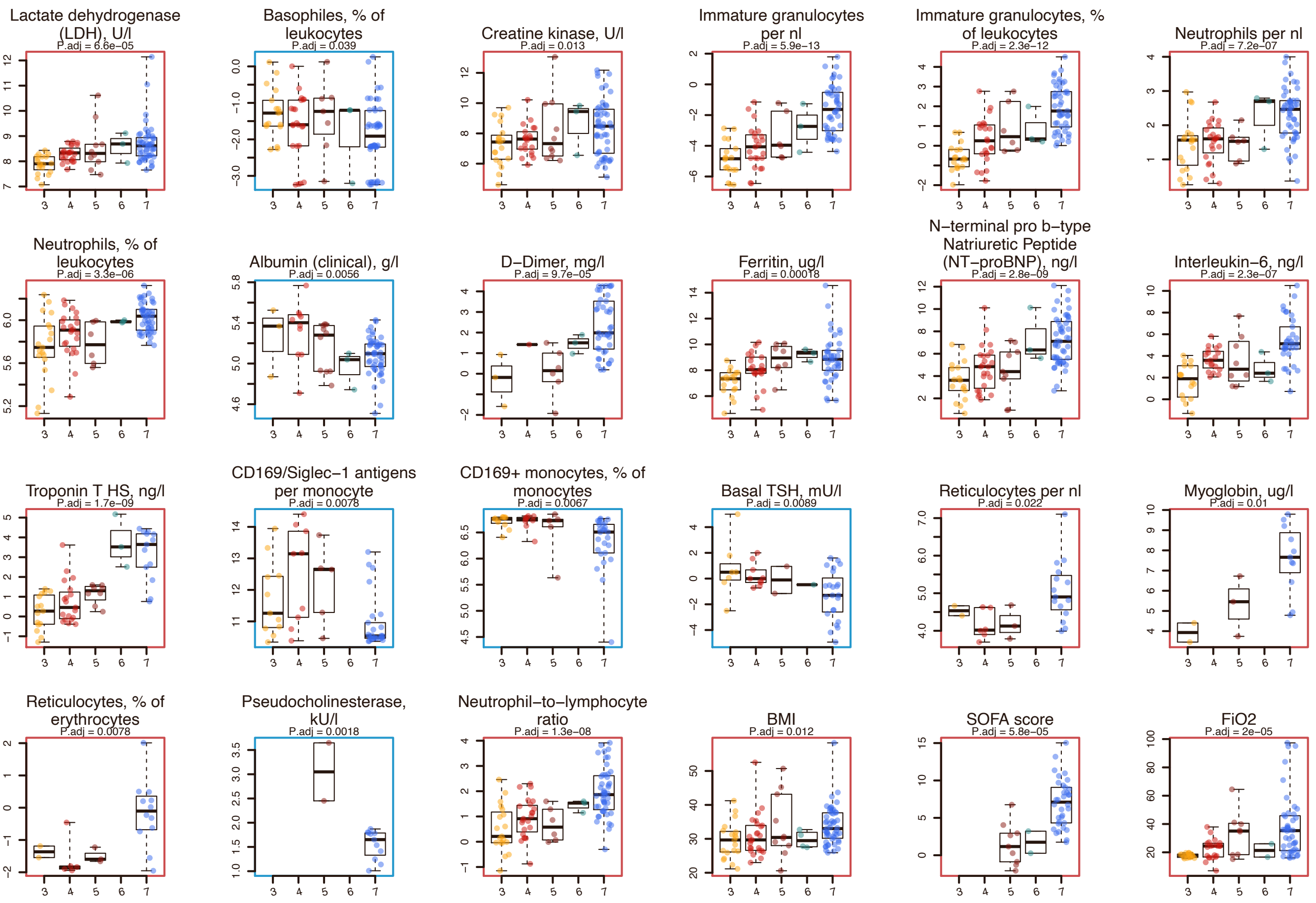
Lactate dehydrogenase (LDH), U/l
P.adj = 6.6e−05
Basophiles, % of leukocytes
P.adj = 0.039
Creatine kinase, U/l
P.adj = 0.013
Immature granulocytes per nl
P.adj = 5.9e−13
Immature granulocytes, % of leukocytes
P.adj = 2.3e−12
Neutrophils per nl
P.adj = 7.2e−07
Neutrophils, % of leukocytes
P.adj = 3.3e−06
Albumin (clinical), g/l
P.adj = 0.0056
D−Dimer, mg/l
P.adj = 9.7e−05
Ferritin, ug/l
P.adj = 0.00018
N−terminal pro b−type Natriuretic Peptide (NT−proBNP), ng/l
P.adj = 2.8e−09
Interleukin−6, ng/l
P.adj = 2.3e−07
Troponin T HS, ng/l
P.adj = 1.7e−09
CD169/Siglec−1 antigens per monocyte
P.adj = 0.0078
CD169+ monocytes, % of monocytes
P.adj = 0.0067
Basal TSH, mU/l
P.adj = 0.0089
Reticulocytes per nl
P.adj = 0.022
Myoglobin, ug/l
P.adj = 0.01
Reticulocytes, % of erythrocytes
P.adj = 0.0078
Pseudocholinesterase, kU/l
P.adj = 0.0018
Neutrophil−to−lymphocyte ratio
P.adj = 1.3e−08
BMI
P.adj = 0.012
SOFA score
P.adj = 5.8e−05
FiO2
P.adj = 2e−05

Figure S7. **Omics features significantly regulated upon RRT**

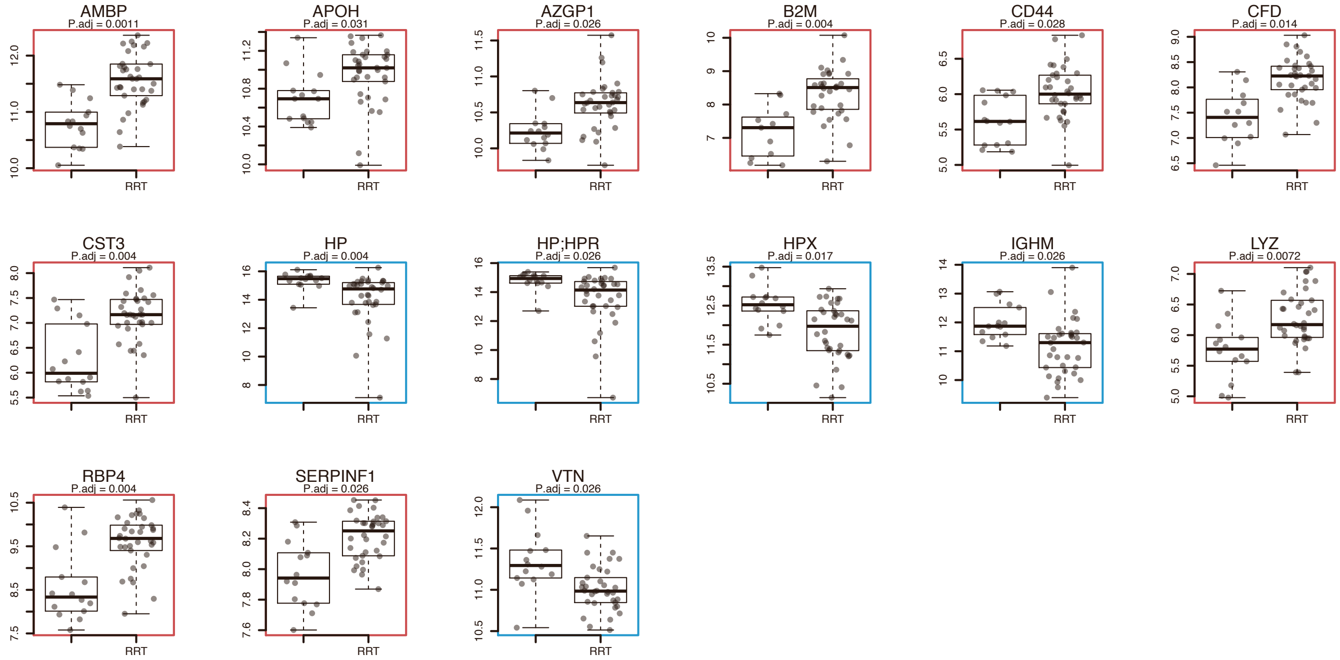

Figure S8. **Omics features significantly regulated upon ECMO**

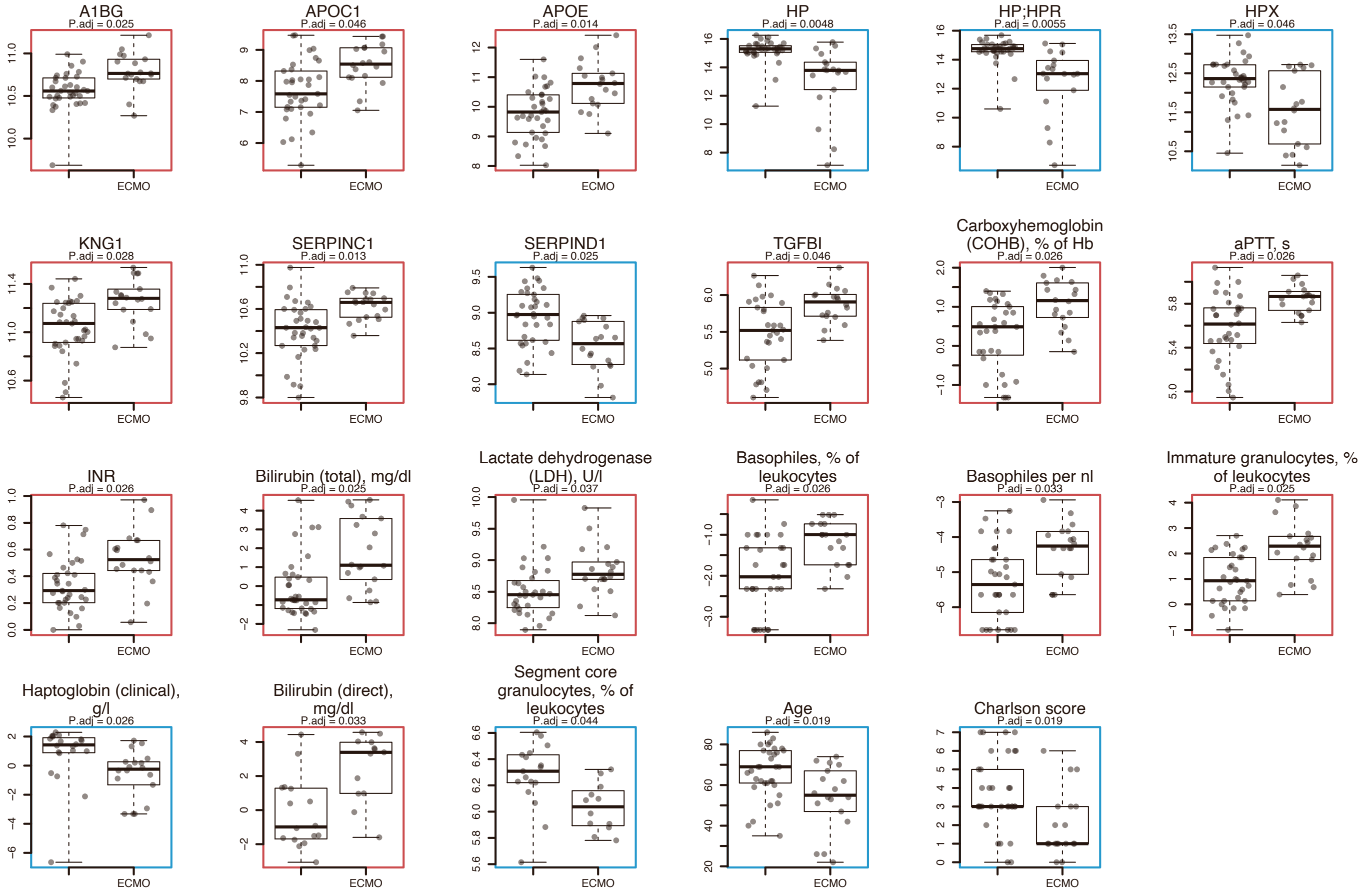

Figure S9 (following 3 pages). **Omics features changing with age - test without accounting for the WHO grade as a covariate**

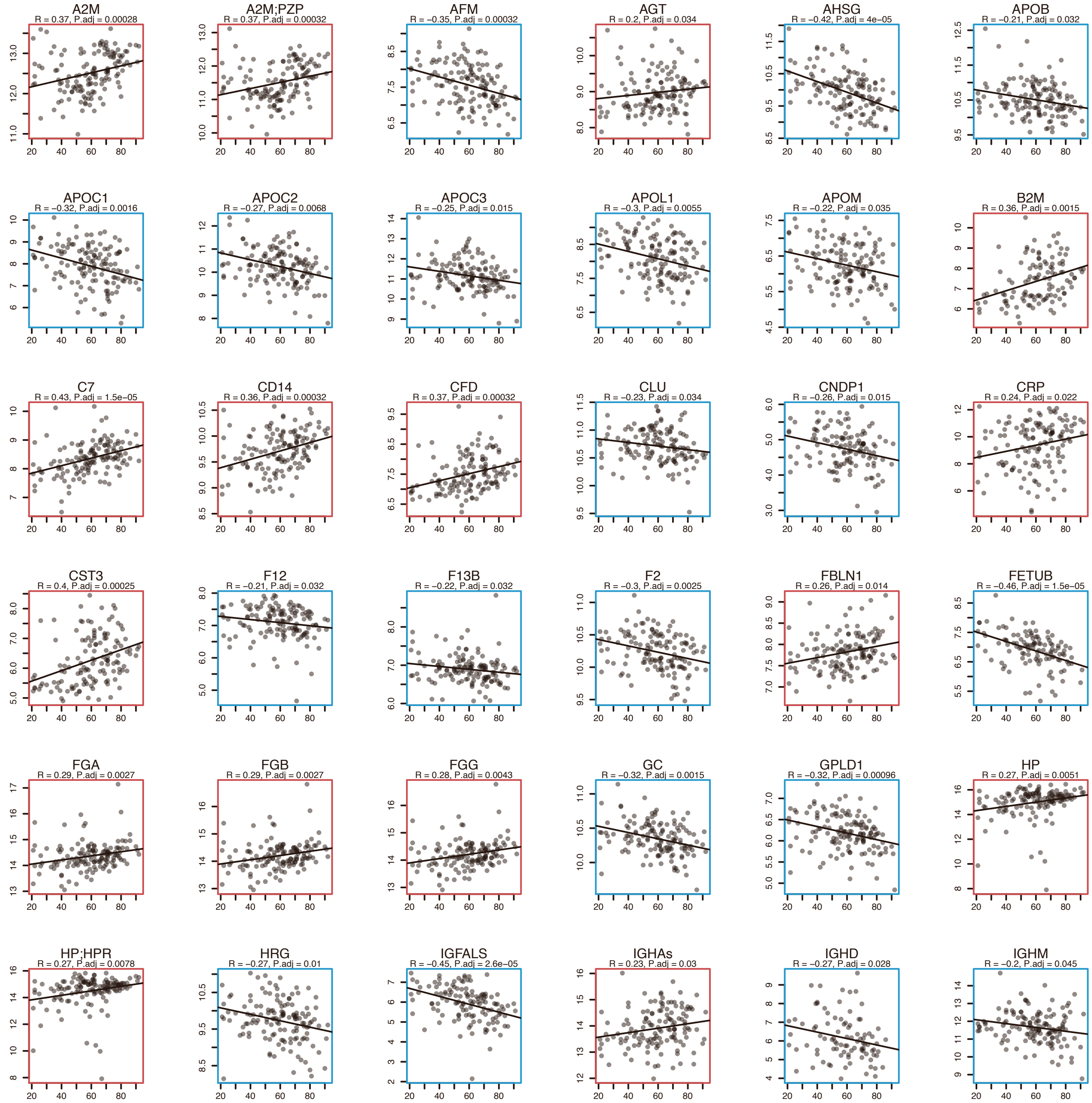

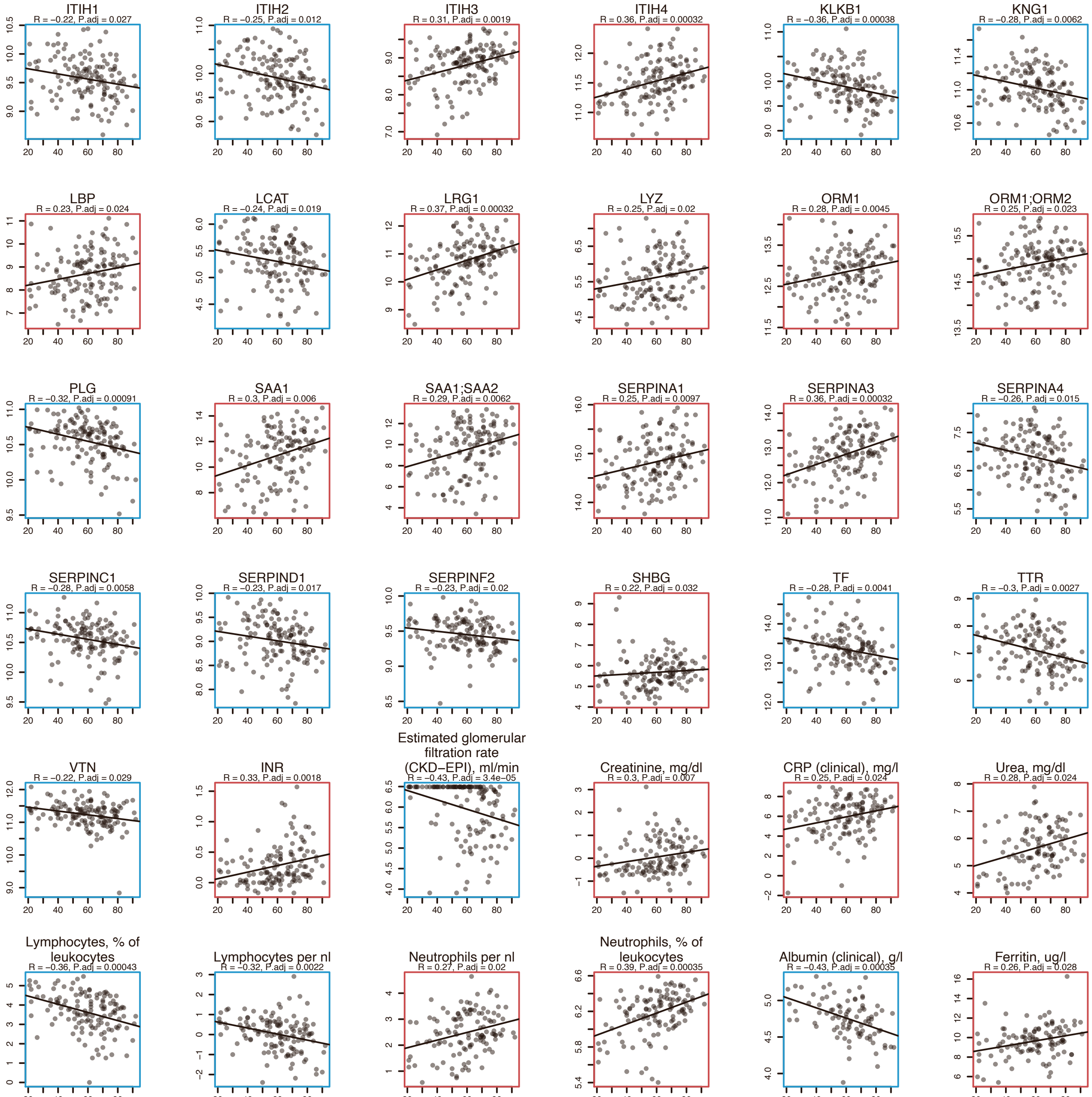
ITIH1
R = −0.22, P.adj = 0.027
ITIH2
R = −0.25, P.adj = 0.012
ITIH3
R = 0.31, P.adj = 0.0019
ITIH4
R = 0.36, P.adj = 0.00032
KLKB1
R = −0.36, P.adj = 0.00038
KNG1
R = −0.28, P.adj = 0.0062
LBP
R = 0.23, P.adj = 0.024
LCAT
R = −0.24, P.adj = 0.019
LRG1
R = 0.37, P.adj = 0.00032
LYZ
R = 0.25, P.adj = 0.02
ORM1
R = 0.28, P.adj = 0.0045
ORM1;ORM2
R = 0.25, P.adj = 0.023
PLG
R = −0.32, P.adj = 0.00091
SAA1
R = 0.3, P.adj = 0.006
SAA1;SAA2
R = 0.29, P.adj = 0.0062
SERPINA1
R = 0.25, P.adj = 0.0097
SERPINA3
R = 0.36, P.adj = 0.00032
SERPINA4
R = −0.26, P.adj = 0.015
SERPINC1
R = −0.28, P.adj = 0.0058
SERPIND1
R = −0.23, P.adj = 0.017
SERPINF2
R = −0.23, P.adj = 0.02
SHBG
R = 0.22, P.adj = 0.032
TF
R = −0.28, P.adj = 0.0041
TTR
R = −0.3, P.adj = 0.0027
VTN
R = −0.22, P.adj = 0.029
INR
R = 0.33, P.adj = 0.0018
Estimated glomerular filtration rate (CKD−EPI), ml/min
R = −0.43, P.adj = 3.4e−05
Creatinine, mg/dl
R = 0.3, P.adj = 0.007
CRP (clinical), mg/l
R = 0.25, P.adj = 0.024
Urea, mg/dl
R = 0.28, P.adj = 0.024
Lymphocytes, % of leukocytes
R = −0.36, P.adj = 0.00043
Lymphocytes per nl
R = −0.32, P.adj = 0.0022
Neutrophils per nl
R = 0.27, P.adj = 0.02
Neutrophils, % of leukocytes
R = 0.39, P.adj = 0.00035
Albumin (clinical), g/l
R = −0.43, P.adj = 0.00035
Ferritin, ug/l
R = 0.26, P.adj = 0.028

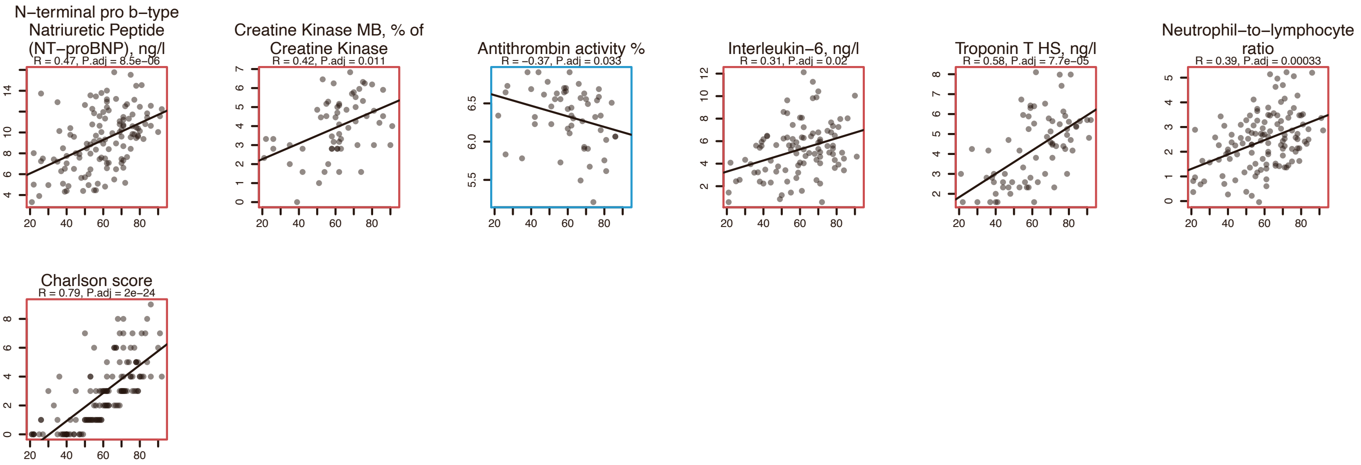
N−terminal pro b−type Natriuretic Peptide (NT−proBNP), ng/l
R = 0.47, P.adj = 8.5e−06
Creatine Kinase MB, % of Creatine Kinase
R = 0.42, P.adj = 0.011
Antithrombin activity %
R = −0.37, P.adj = 0.033
Interleukin−6, ng/l
R = 0.31, P.adj = 0.02
Troponin T HS, ng/l
R = 0.58, P.adj = 7.7e−05
Neutrophil−to−lymphocyte ratio
R = 0.39, P.adj = 0.00033
Charlson score
R = 0.79, P.adj = 2e−24

Figure S10 (following 9 pages). **Plasma protein levels vs age in general population based on Generation Scotland cohort**

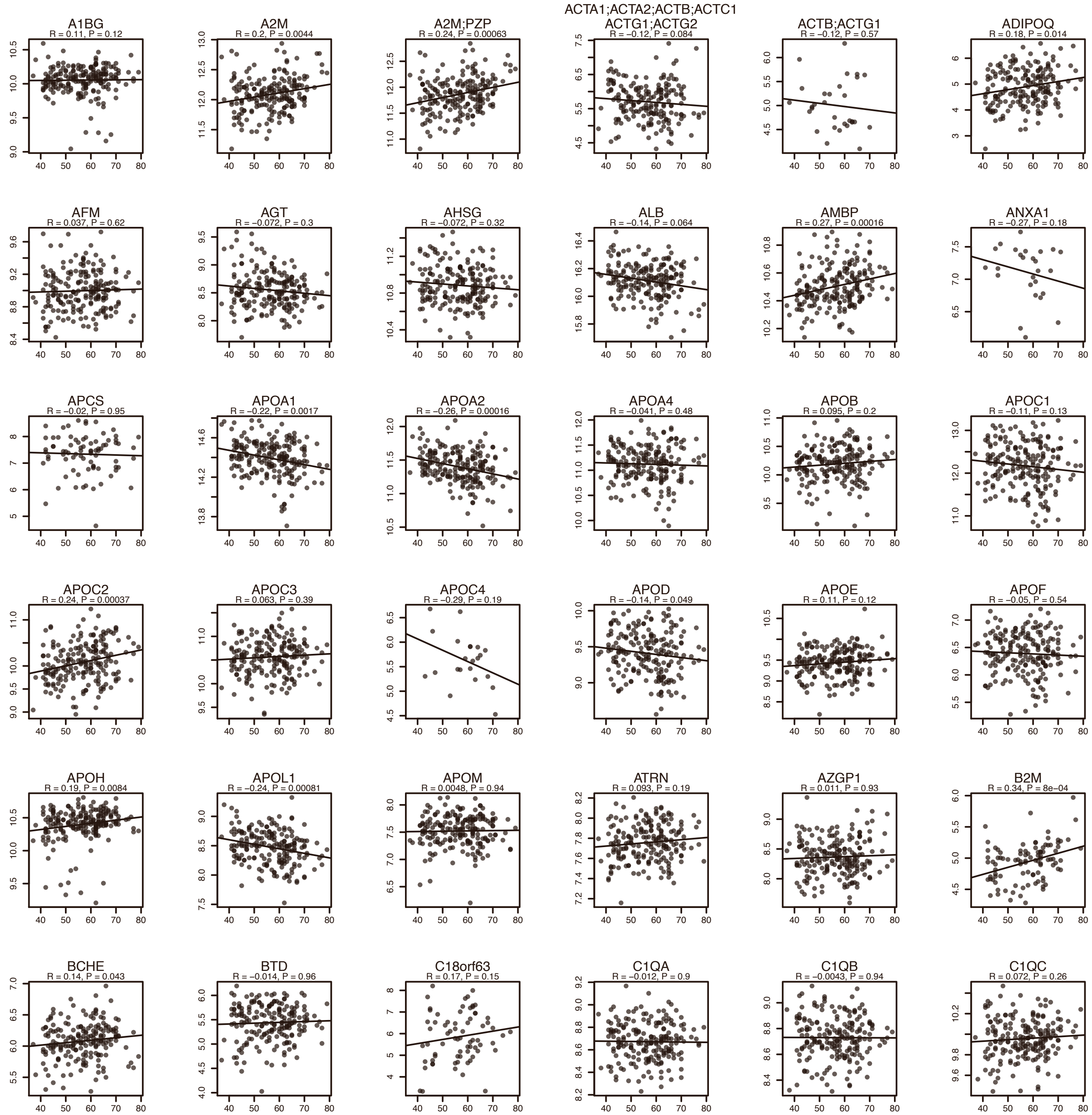

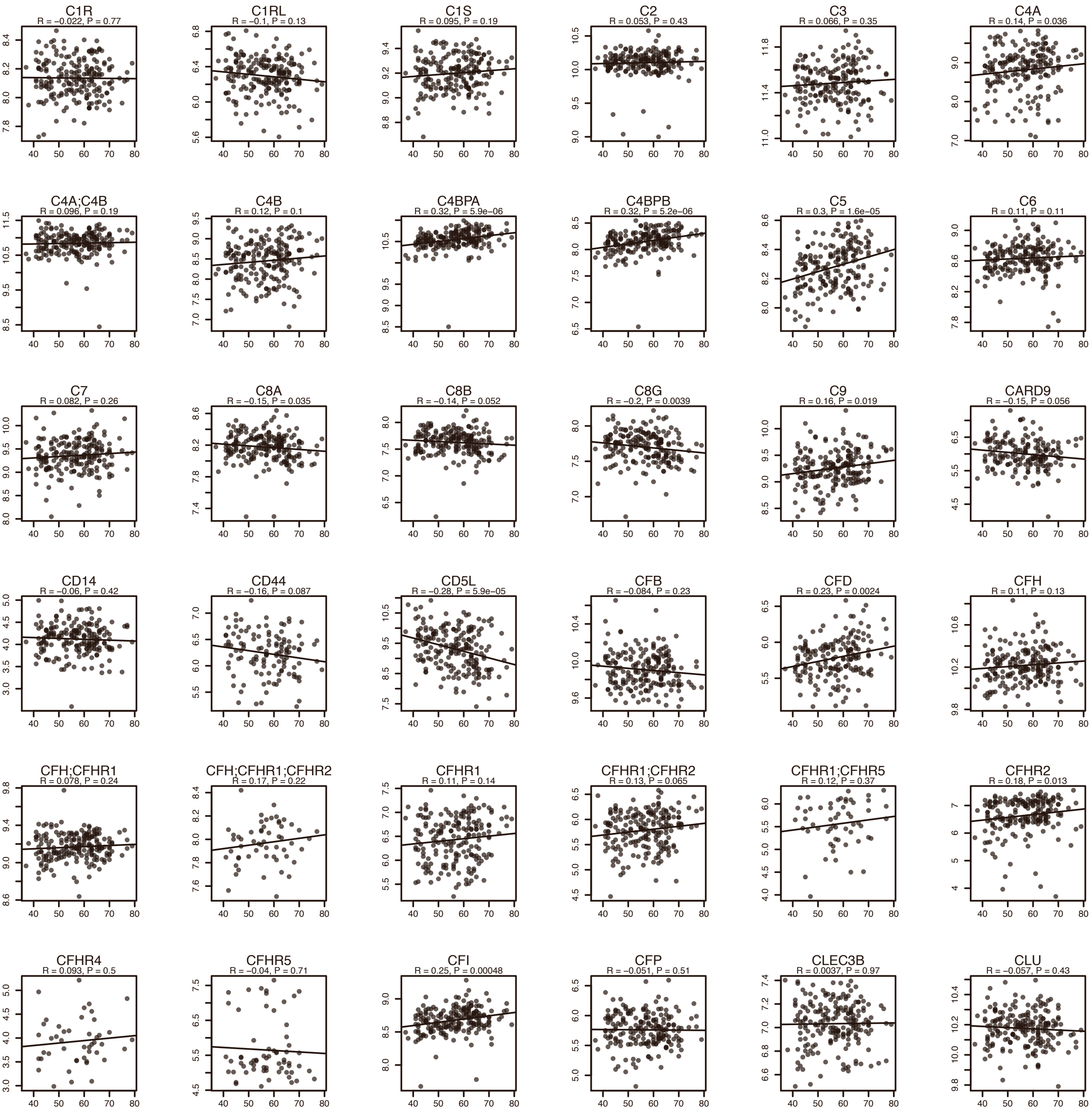
C1R
R = −0.022, P = 0.77
C1RL
R = −0.1, P = 0.13
C1S
R = 0.095, P = 0.19
C2
R = 0.053, P = 0.43
C3
R = 0.066, P = 0.35
C4A
R = 0.14, P = 0.036
C4A;C4B
R = 0.096, P = 0.19
C4B
R = 0.12, P = 0.1
C4BPA
R = 0.32, P = 5.9e−06
C4BPB
R = 0.32, P = 5.2e−06
C5
R = 0.3, P = 1.6e−05
C6
R = 0.11, P = 0.11
C7
R = 0.082, P = 0.26
C8A
R = −0.15, P = 0.035
C8B
R = −0.14, P = 0.052
C8G
R = −0.2, P = 0.0039
C9
R = 0.16, P = 0.019
CARD9
R = −0.15, P = 0.056
CD14
R = −0.06, P = 0.42
CD44
R = −0.16, P = 0.087
CD5L
R = −0.28, P = 5.9e−05
CFB
R = −0.084, P = 0.23
CFD
R = 0.23, P = 0.0024
CFH
R = 0.11, P = 0.13
CFH;CFHR1
R = 0.078, P = 0.24
CFH;CFHR1;CFHR2
R = 0.17, P = 0.22
CFHR1
R = 0.11, P = 0.14
CFHR1;CFHR2
R = 0.13, P = 0.065
CFHR1;CFHR5
R = 0.12, P = 0.37
CFHR2
R = 0.18, P = 0.013
CFHR4
R = 0.093, P = 0.5
CFHR5
R = −0.04, P = 0.71
CFI
R = 0.25, P = 0.00048
CFP
R = −0.051, P = 0.51
CLEC3B
R = 0.0037, P = 0.97
CLU
R = −0.057, P = 0.43

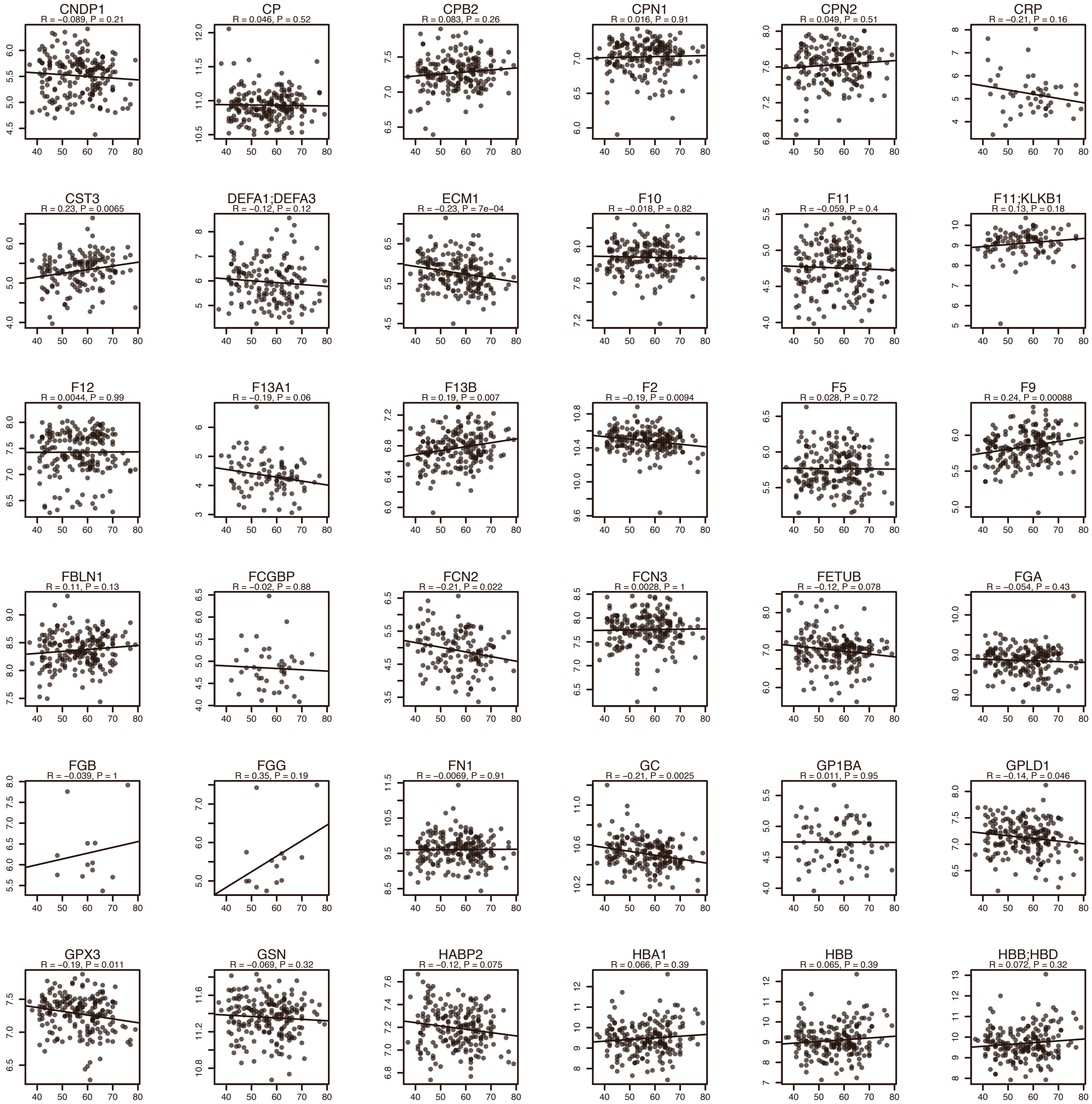
CNDP1
R = −0.089, P = 0.21
CP
R = 0.046, P = 0.52
CPB2
R = 0.083, P = 0.26
CPN1
R = 0.016, P = 0.91
CPN2
R = 0.049, P = 0.51
CRP
R = −0.21, P = 0.16
CST3
R = 0.23, P = 0.0065
DEFA1;DEFA3
R = −0.12, P = 0.12
ECM1
R = −0.23, P = 7e−04
F10
R = −0.018, P = 0.82
F11
R = −0.059, P = 0.4
F11;KLKB1
R = 0.13, P = 0.18
F12
R = 0.0044, P = 0.99
F13A1
R = −0.19, P = 0.06
F13B
R = 0.19, P = 0.007
F2
R = −0.19, P = 0.0094
F5
R = 0.028, P = 0.72
F9
R = 0.24, P = 0.00088
FBLN1
R = 0.11, P = 0.13
FCGBP
R = −0.02, P = 0.88
FCN2
R = −0.21, P = 0.022
FCN3
R = 0.0028, P = 1
FETUB
R = −0.12, P = 0.078
FGA
R = −0.054, P = 0.43
FGB
R = −0.039, P = 1
FGG
R = 0.35, P = 0.19
FN1
R = −0.0069, P = 0.91
GC
R = −0.21, P = 0.0025
GP1BA
R = 0.011, P = 0.95
GPLD1
R = −0.14, P = 0.046
GPX3
R = −0.19, P = 0.011
GSN
R = −0.069, P = 0.32
HABP2
R = −0.12, P = 0.075
HBA1
R = 0.066, P = 0.39
HBB
R = 0.065, P = 0.39
HBB;HBD
R = 0.072, P = 0.32

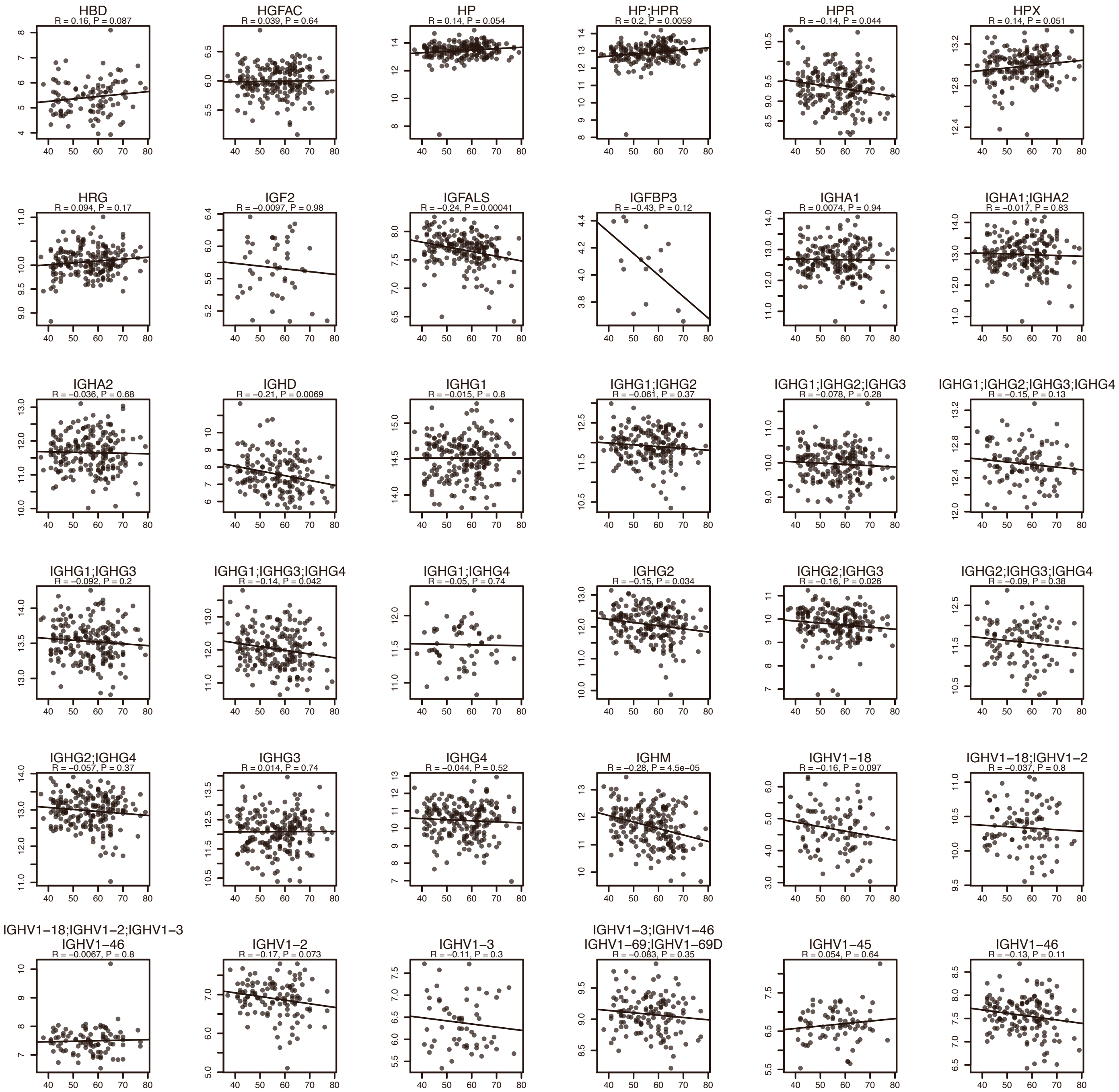
HBD
R = 0.16, P = 0.087
HGFAC
R = 0.039, P = 0.64
HP
R = 0.14, P = 0.054
HP;HPR
R = 0.2, P = 0.0059
HPR
R = −0.14, P = 0.044
HPX
R = 0.14, P = 0.051
HRG
R = 0.094, P = 0.17
IGF2
R = −0.0097, P = 0.98
IGFALS
R = −0.24, P = 0.00041
IGFBP3
R = −0.43, P = 0.12
IGHA1
R = 0.0074, P = 0.94
IGHA1;IGHA2
R = −0.017, P = 0.83
IGHA2
R = −0.036, P = 0.68
IGHD
R = −0.21, P = 0.0069
IGHG1
R = −0.015, P = 0.8
IGHG1;IGHG2
R = −0.061, P = 0.37
IGHG1;IGHG2;IGHG3
R = −0.078, P = 0.28
IGHG1;IGHG2;IGHG3;IGHG4
R = −0.15, P = 0.13
IGHG1;IGHG3
R = −0.092, P = 0.2
IGHG1;IGHG3;IGHG4
R = −0.14, P = 0.042
IGHG1;IGHG4
R = −0.05, P = 0.74
IGHG2
R = −0.15, P = 0.034
IGHG2;IGHG3
R = −0.16, P = 0.026
IGHG2;IGHG3;IGHG4
R = −0.09, P = 0.38
IGHG2;IGHG4
R = −0.057, P = 0.37
IGHG3
R = 0.014, P = 0.74
IGHG4
R = −0.044, P = 0.52
IGHM
R = −0.28, P = 4.5e−05
IGHV1−18
R = −0.16, P = 0.097
IGHV1−18;IGHV1−2
R = −0.037, P = 0.8
IGHV1−18;IGHV1−2;IGHV1−3
IGHV1−46
R = −0.0067, P = 0.8
IGHV1−2
R = −0.17, P = 0.073
IGHV1−3
R = −0.11, P = 0.3
IGHV1−3;IGHV1−46
IGHV1−69;IGHV1−69D
R = −0.083, P = 0.35
IGHV1−45
R = 0.054, P = 0.64
IGHV1−46
R = −0.13, P = 0.11

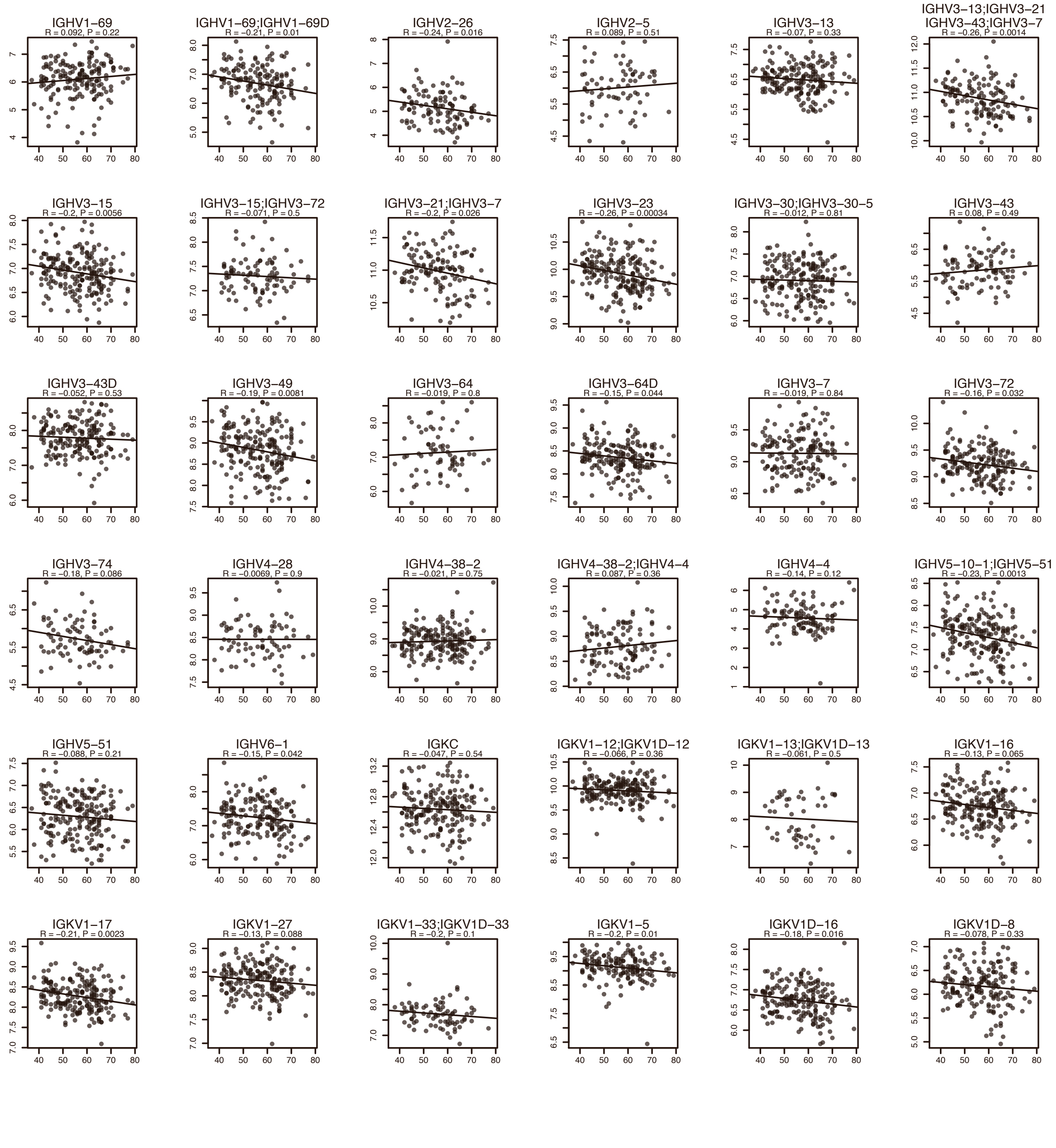
IGHV1−69
R = 0.092, P = 0.22
IGHV1−69;IGHV1−69D
R = −0.21, P = 0.01
IGHV2−26
R = −0.24, P = 0.016
IGHV2−5
R = 0.089, P = 0.51
IGHV3−13
R = −0.07, P = 0.33
IGHV3−13;IGHV3−21
IGHV3−43;IGHV3−7
R = −0.26, P = 0.0014
IGHV3−15
R = −0.2, P = 0.0056
IGHV3−15;IGHV3−72
R = −0.071, P = 0.5
IGHV3−21;IGHV3−7
R = −0.2, P = 0.026
IGHV3−23
R = −0.26, P = 0.00034
IGHV3−30;IGHV3−30−5
R = −0.012, P = 0.81
IGHV3−43
R = 0.08, P = 0.49
IGHV3−43D
R = −0.052, P = 0.53
IGHV3−49
R = −0.19, P = 0.0081
IGHV3−64
R = −0.019, P = 0.8
IGHV3−64D
R = −0.15, P = 0.044
IGHV3−7
R = −0.019, P = 0.84
IGHV3−72
R = −0.16, P = 0.032
IGHV3−74
R = −0.18, P = 0.086
IGHV4−28
R = −0.0069, P = 0.9
IGHV4−38−2
R = −0.021, P = 0.75
IGHV4−38−2;IGHV4−4
R = 0.087, P = 0.36
IGHV4−4
R = −0.14, P = 0.12
IGHV5−10−1;IGHV5−51
R = −0.23, P = 0.0013
IGHV5−51
R = −0.088, P = 0.21
IGHV6−1
R = −0.15, P = 0.042
IGKC
R = −0.047, P = 0.54
IGKV1−12;IGKV1D−12
R = −0.066, P = 0.36
IGKV1−13;IGKV1D−13
R = −0.061, P = 0.5
IGKV1−16
R = −0.13, P = 0.065
IGKV1−17
R = −0.21, P = 0.0023
IGKV1−27
R = −0.13, P = 0.088
IGKV1−33;IGKV1D−33
R = −0.2, P = 0.1
IGKV1−5
R = −0.2, P = 0.01
IGKV1D−16
R = −0.18, P = 0.016
IGKV1D−8
R = −0.078, P = 0.33

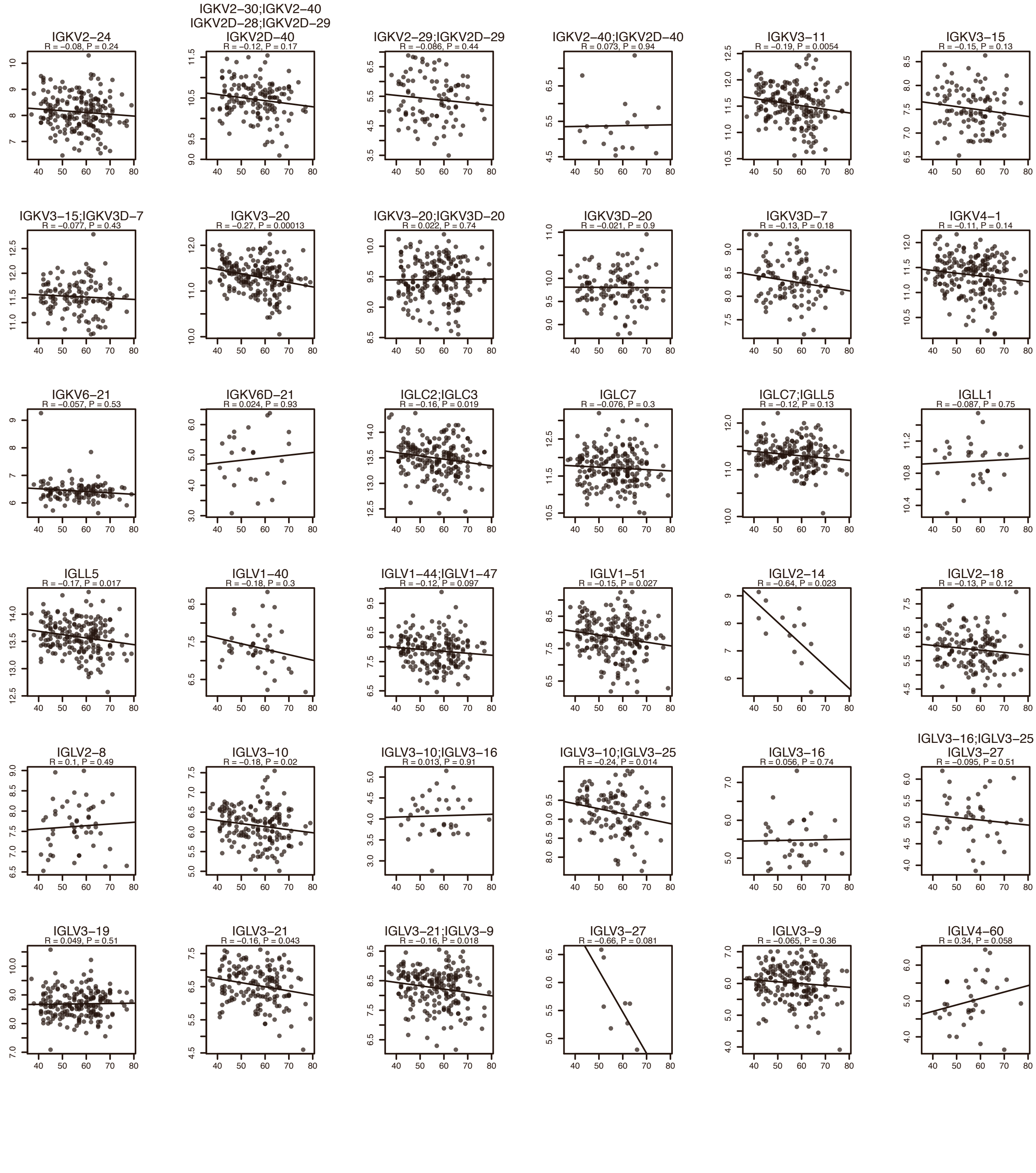
IGKV2−24
R = −0.08, P = 0.24
IGKV2−30;IGKV2−40
IGKV2D−28;IGKV2D−29
IGKV2D−40
R = −0.12, P = 0.17
IGKV2−29;IGKV2D−29
R = −0.086, P = 0.44
IGKV2−40;IGKV2D−40
R = 0.073, P = 0.94
IGKV3−11
R = −0.19, P = 0.0054
IGKV3−15
R = −0.15, P = 0.13
IGKV3−15;IGKV3D−7
R = −0.077, P = 0.43
IGKV3−20
R = −0.27, P = 0.00013
IGKV3−20;IGKV3D−20
R = 0.022, P = 0.74
IGKV3D−20
R = −0.021, P = 0.9
IGKV3D−7
R = −0.13, P = 0.18
IGKV4−1
R = −0.11, P = 0.14
IGKV6−21
R = −0.057, P = 0.53
IGKV6D−21
R = 0.024, P = 0.93
IGLC2;IGLC3
R = −0.16, P = 0.019
IGLC7
R = −0.076, P = 0.3
IGLC7;IGLL5
R = −0.12, P = 0.13
IGLL1
R = −0.087, P = 0.75
IGLL5
R = −0.17, P = 0.017
IGLV1−40
R = −0.18, P = 0.3
IGLV1−44;IGLV1−47
R = −0.12, P = 0.097
IGLV1−51
R = −0.15, P = 0.027
IGLV2−14
R = −0.64, P = 0.023
IGLV2−18
R = −0.13, P = 0.12
IGLV2−8
R = 0.1, P = 0.49
IGLV3−10
R = −0.18, P = 0.02
IGLV3−10;IGLV3−16
R = 0.013, P = 0.91
IGLV3−10;IGLV3−25
R = −0.24, P = 0.014
IGLV3−16
R = 0.056, P = 0.74
IGLV3−16;IGLV3−25
IGLV3−27
R = −0.095, P = 0.51
IGLV3−19
R = 0.049, P = 0.51
IGLV3−21
R = −0.16, P = 0.043
IGLV3−21;IGLV3−9
R = −0.16, P = 0.018
IGLV3−27
R = −0.66, P = 0.081
IGLV3−9
R = −0.065, P = 0.36
IGLV4−60
R = 0.34, P = 0.058

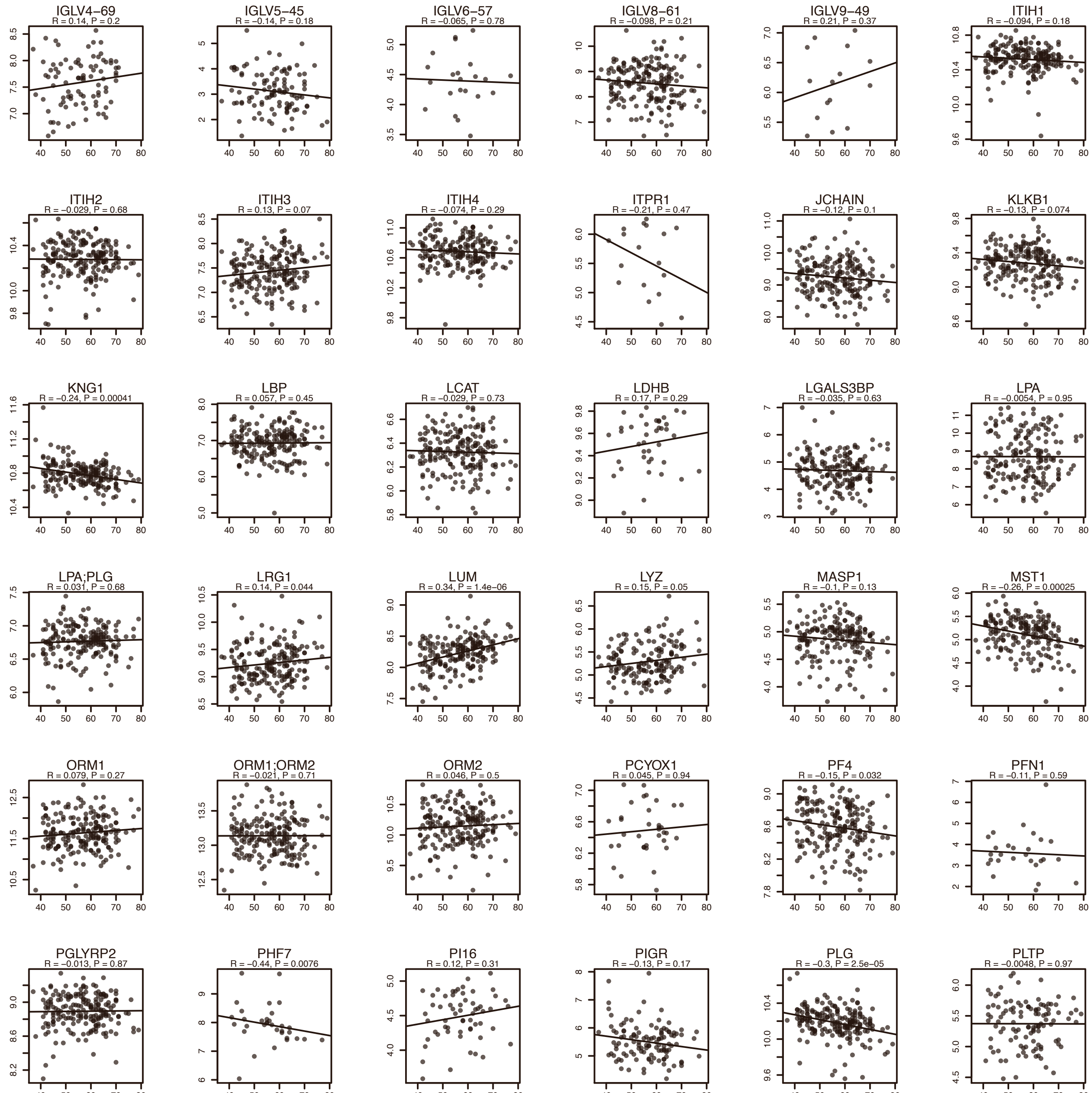
IGLV4−69
R = 0.14, P = 0.2
IGLV5−45
R = −0.14, P = 0.18
IGLV6−57
R = −0.065, P = 0.78
IGLV8−61
R = −0.098, P = 0.21
IGLV9−49
R = 0.21, P = 0.37
ITIH1
R = −0.094, P = 0.18
ITIH2
R = −0.029, P = 0.68
ITIH3
R = 0.13, P = 0.07
ITIH4
R = −0.074, P = 0.29
ITPR1
R = −0.21, P = 0.47
JCHAIN
R = −0.12, P = 0.1
KLKB1
R = −0.13, P = 0.074
KNG1
R = −0.24, P = 0.00041
LBP
R = 0.057, P = 0.45
LCAT
R = −0.029, P = 0.73
LDHB
R = 0.17, P = 0.29
LGALS3BP
R = −0.035, P = 0.63
LPA
R = −0.0054, P = 0.95
LPA;PLG
R = 0.031, P = 0.68
LRG1
R = 0.14, P = 0.044
LUM
R = 0.34, P = 1.4e−06
LYZ
R = 0.15, P = 0.05
MASP1
R = −0.1, P = 0.13
MST1
R = −0.26, P = 0.00025
ORM1
R = 0.079, P = 0.27
ORM1;ORM2
R = −0.021, P = 0.71
ORM2
R = 0.046, P = 0.5
PCYOX1
R = 0.045, P = 0.94
PF4
R = −0.15, P = 0.032
PFN1
R = −0.11, P = 0.59
PGLYRP2
R = −0.013, P = 0.87
PHF7
R = −0.44, P = 0.0076
PI16
R = 0.12, P = 0.31
PIGR
R = −0.13, P = 0.17
PLG
R = −0.3, P = 2.5e−05
PLTP
R = −0.0048, P = 0.97

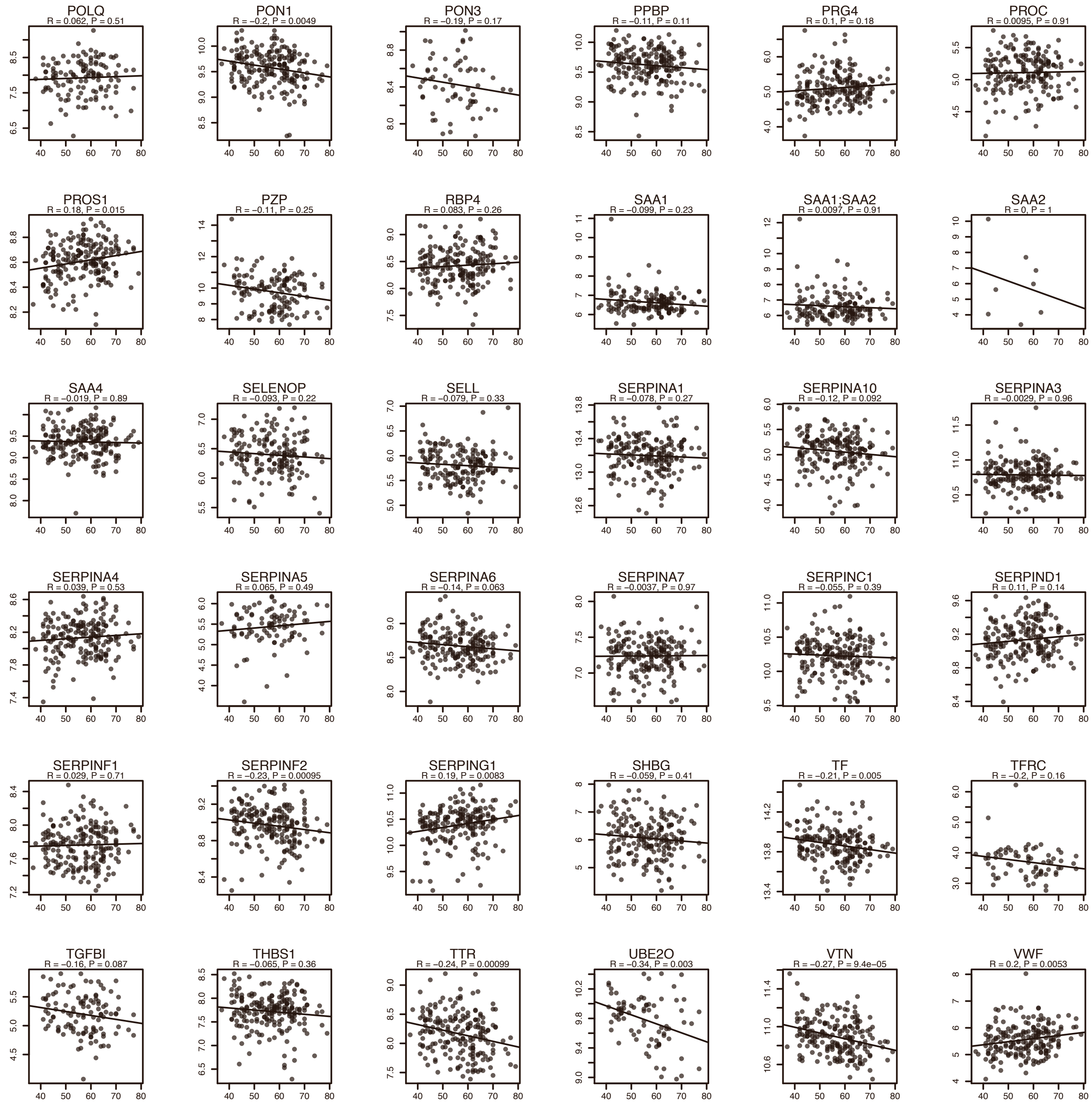
POLQ
R = 0.062, P = 0.51
PON1
R = −0.2, P = 0.0049
PON3
R = −0.19, P = 0.17
PPBP
R = −0.11, P = 0.11
PRG4
R = 0.1, P = 0.18
PROC
R = 0.0095, P = 0.91
PROS1
R = 0.18, P = 0.015
PZP
R = −0.11, P = 0.25
RBP4
R = 0.083, P = 0.26
SAA1
R = −0.099, P = 0.23
SAA1;SAA2
R = 0.0097, P = 0.91
SAA2
R = 0, P = 1
SAA4
R = −0.019, P = 0.89
SELENOP
R = −0.093, P = 0.22
SELL
R = −0.079, P = 0.33
SERPINA1
R = −0.078, P = 0.27
SERPINA10
R = −0.12, P = 0.092
SERPINA3
R = −0.0029, P = 0.96
SERPINA4
R = 0.039, P = 0.53
SERPINA5
R = 0.065, P = 0.49
SERPINA6
R = −0.14, P = 0.063
SERPINA7
R = −0.0037, P = 0.97
SERPINC1
R = −0.055, P = 0.39
SERPIND1
R = 0.11, P = 0.14
SERPINF1
R = 0.029, P = 0.71
SERPINF2
R = −0.23, P = 0.00095
SERPING1
R = 0.19, P = 0.0083
SHBG
R = −0.059, P = 0.41
TF
R = −0.21, P = 0.005
TFRC
R = −0.2, P = 0.16
TGFBI
R = −0.16, P = 0.087
THBS1
R = −0.065, P = 0.36
TTR
R = −0.24, P = 0.00099
UBE2O
R = −0.34, P = 0.003
VTN
R = −0.27, P = 9.4e−05
VWF
R = 0.2, P = 0.0053

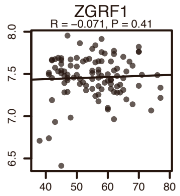
ZGRF1
R = −0.071, P = 0.41
8.0
7.5
7.0
6.5
40
50
60
70
80

Figure S11 (following 2 pages). **Omics features significantly regulated depending on age**

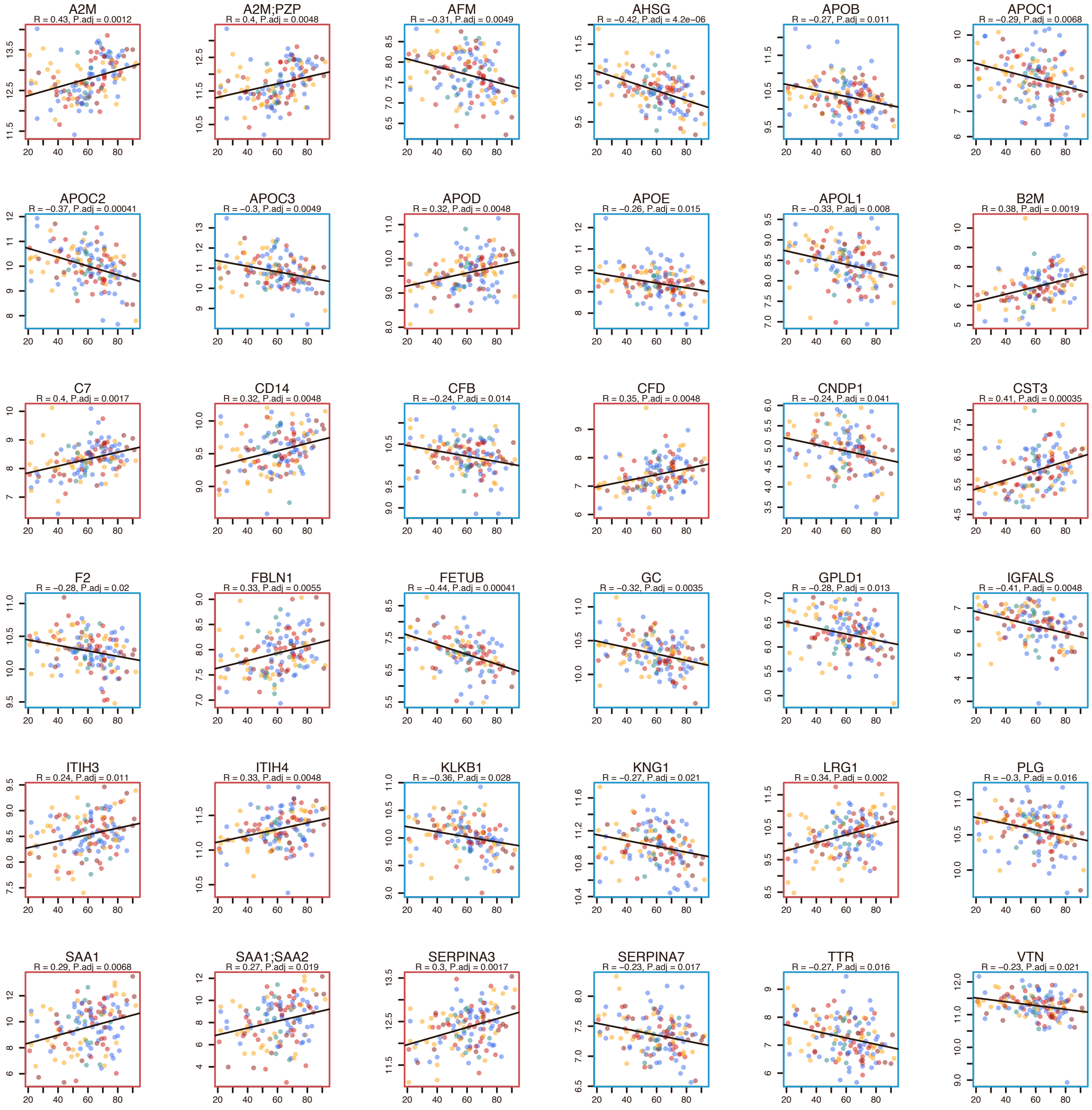

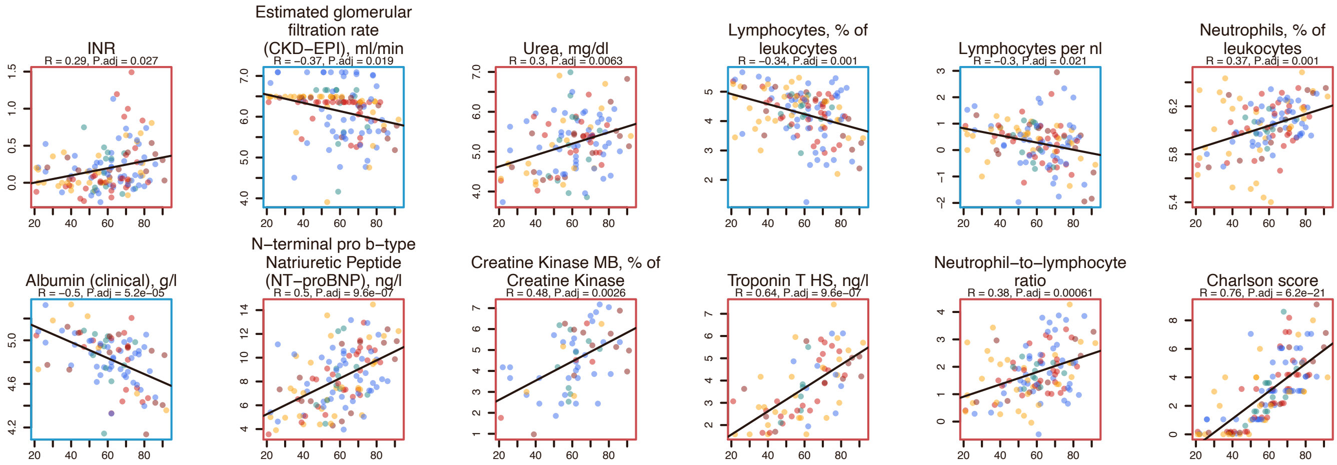
INR
R = 0.29, P.adj = 0.027
Estimated glomerular filtration rate (CKD−EPI), ml/min
R = −0.37, P.adj = 0.019
Urea, mg/dl
R = 0.3, P.adj = 0.0063
Lymphocytes, % of leukocytes
R = −0.34, P.adj = 0.001
Lymphocytes per nl
R = −0.3, P.adj = 0.021
Neutrophils, % of leukocytes
R = 0.37, P.adj = 0.001
Albumin (clinical), g/l
R = −0.5, P.adj = 5.2e−05
N−terminal pro b−type Natriuretic Peptide (NT−proBNP), ng/l
R = 0.5, P.adj = 9.6e−07
Creatine Kinase MB, % of Creatine Kinase
R = 0.48, P.adj = 0.0026
Troponin T HS, ng/l
R = 0.64, P.adj = 9.6e−07
Neutrophil−to−lymphocyte ratio
R = 0.38, P.adj = 0.00061
Charlson score
R = 0.76, P.adj = 6.2e−21

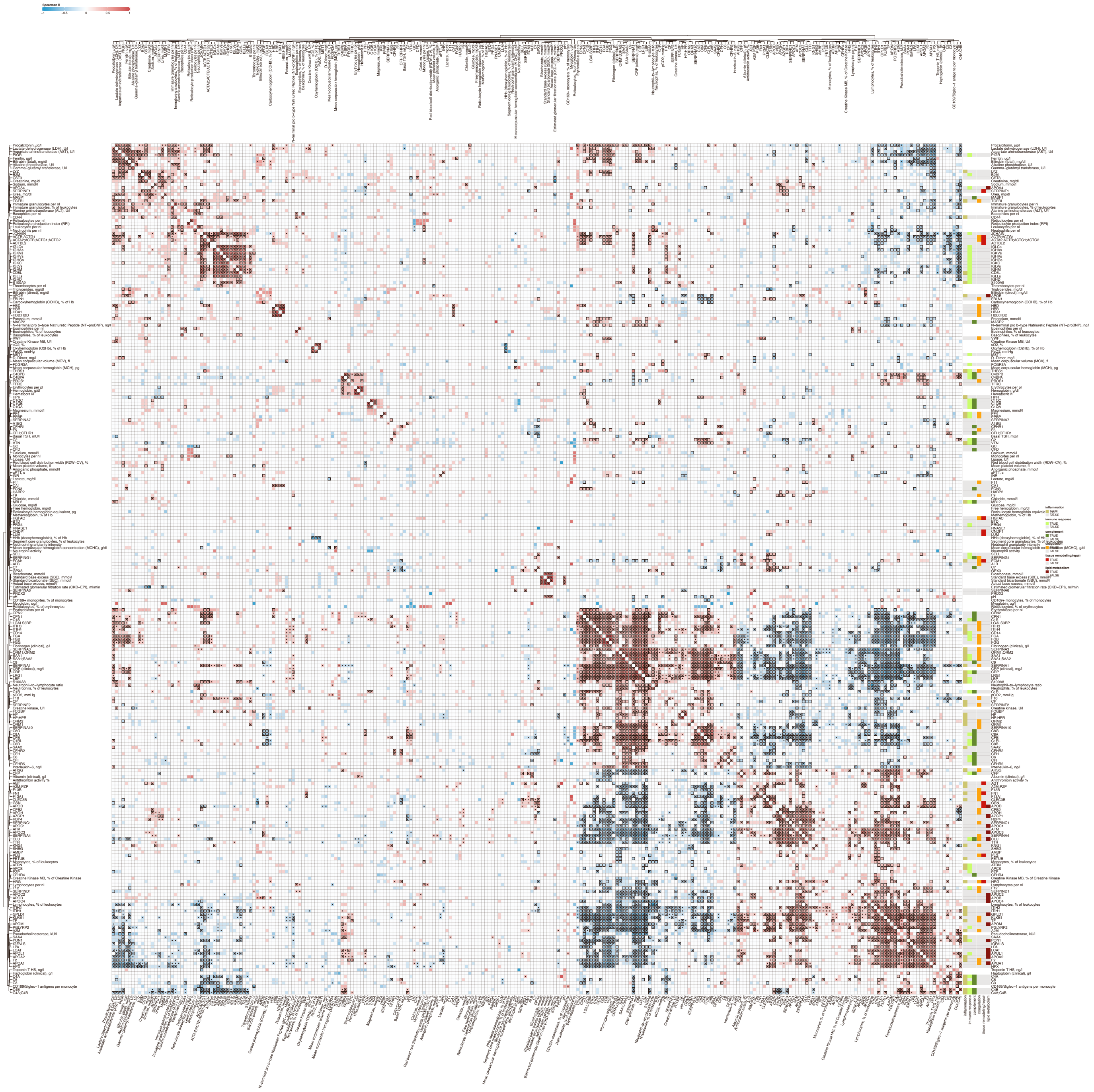

Figure S12. **Map of correlations between dynamic changes in omics measurements during the peak period of the disease**

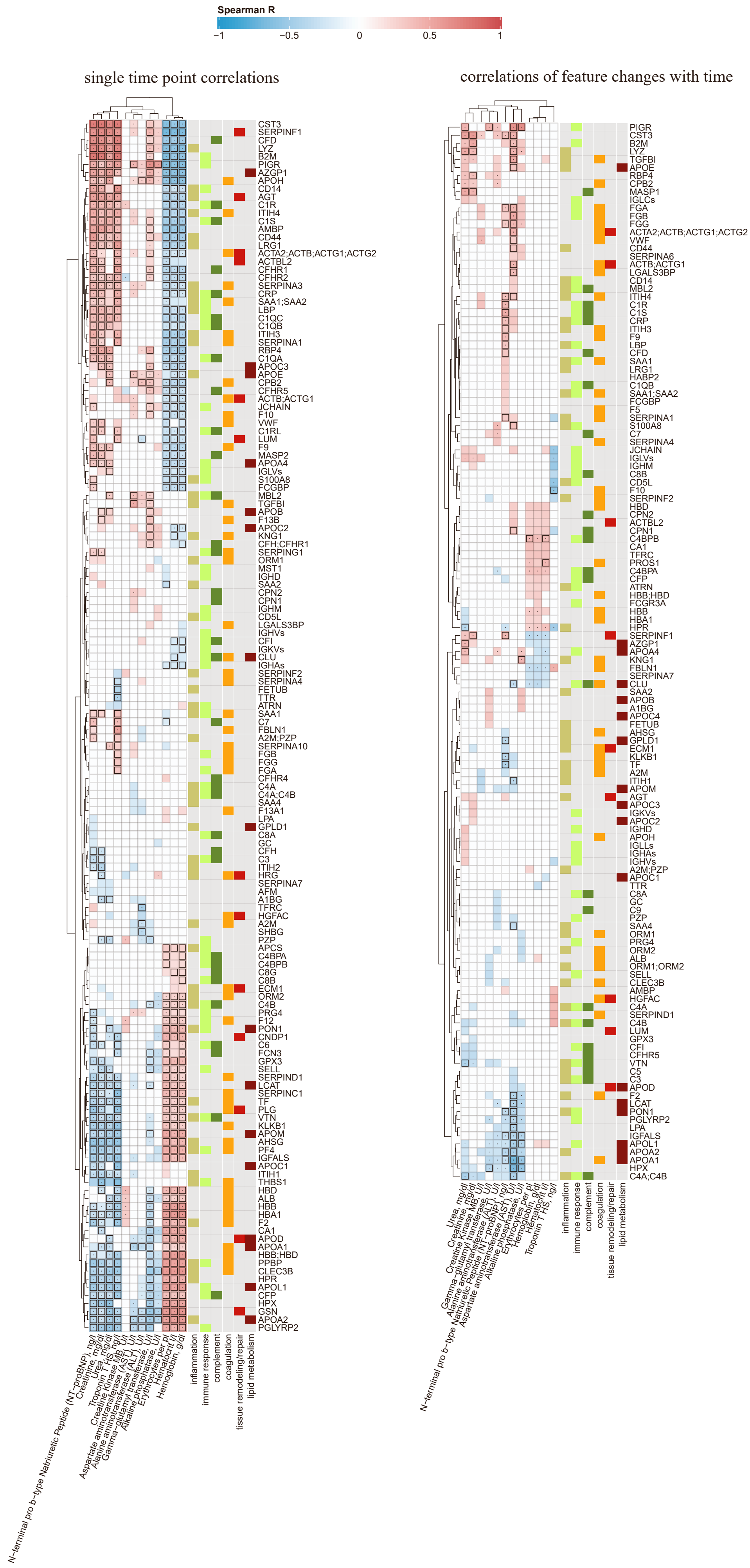

Figure S13. **Covariation of organ function markers and plasma proteins**

Figure S14 (following 4 pages). **Omics features changing during the peak of the disease**

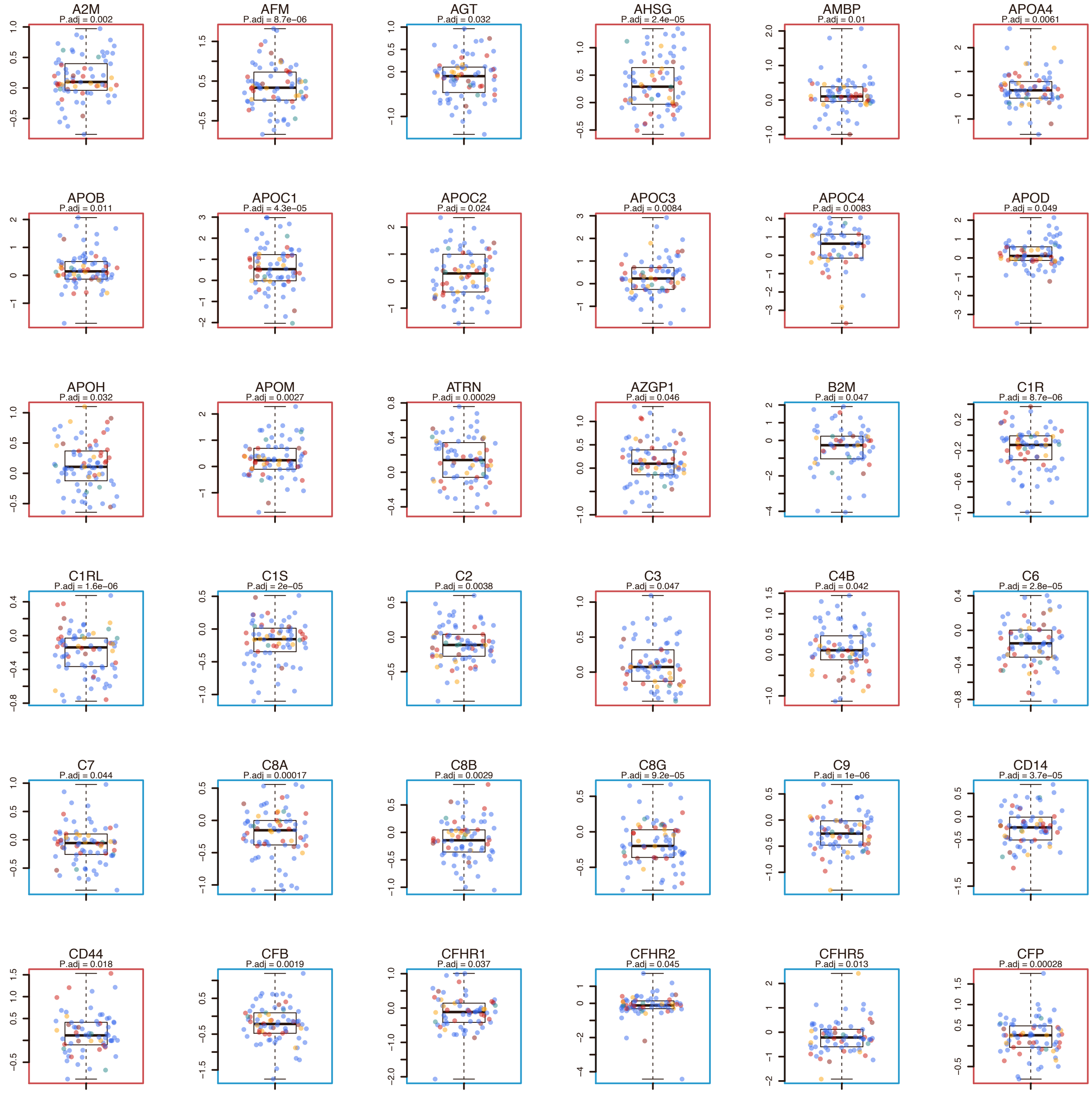

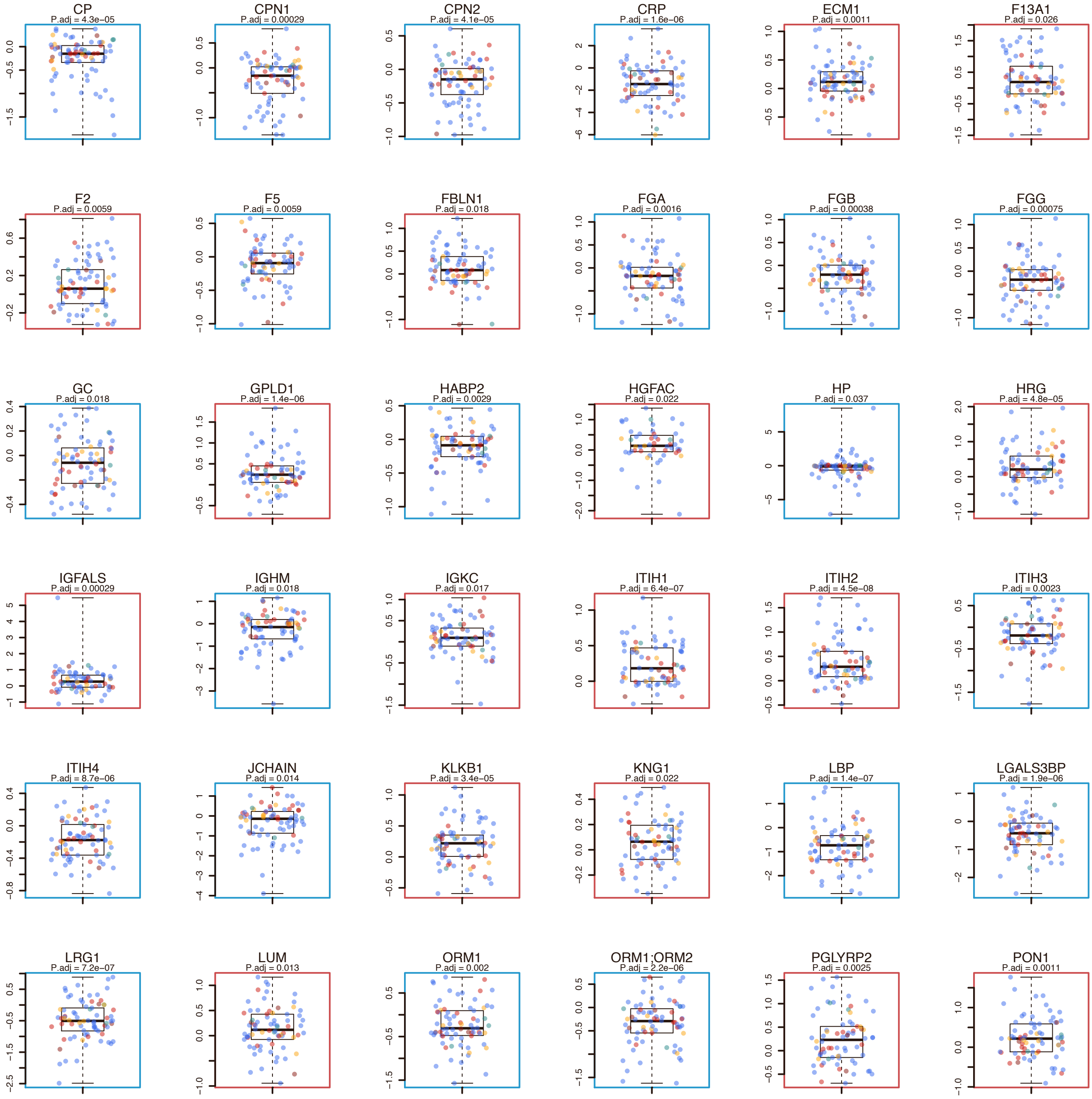
CP
P.adj = 4.3e−05
CPN1
P.adj = 0.00029
CPN2
P.adj = 4.1e−05
CRP
P.adj = 1.6e−06
ECM1
P.adj = 0.0011
F13A1
P.adj = 0.026
F2
P.adj = 0.0059
F5
P.adj = 0.0059
FBLN1
P.adj = 0.018
FGA
P.adj = 0.0016
FGB
P.adj = 0.00038
FGG
P.adj = 0.00075
GC
P.adj = 0.018
GPLD1
P.adj = 1.4e−06
HABP2
P.adj = 0.0029
HGFAC
P.adj = 0.022
HP
P.adj = 0.037
HRG
P.adj = 4.8e−05
IGFALS
P.adj = 0.00029
IGHM
P.adj = 0.018
IGKC
P.adj = 0.017
ITIH1
P.adj = 6.4e−07
ITIH2
P.adj = 4.5e−08
ITIH3
P.adj = 0.0023
ITIH4
P.adj = 8.7e−06
JCHAIN
P.adj = 0.014
KLKB1
P.adj = 3.4e−05
KNG1
P.adj = 0.022
LBP
P.adj = 1.4e−07
LGALS3BP
P.adj = 1.9e−06
LRG1
P.adj = 7.2e−07
LUM
P.adj = 0.013
ORM1
P.adj = 0.002
ORM1;ORM2
P.adj = 2.2e−06
PGLYRP2
P.adj = 0.0025
PON1
P.adj = 0.0011

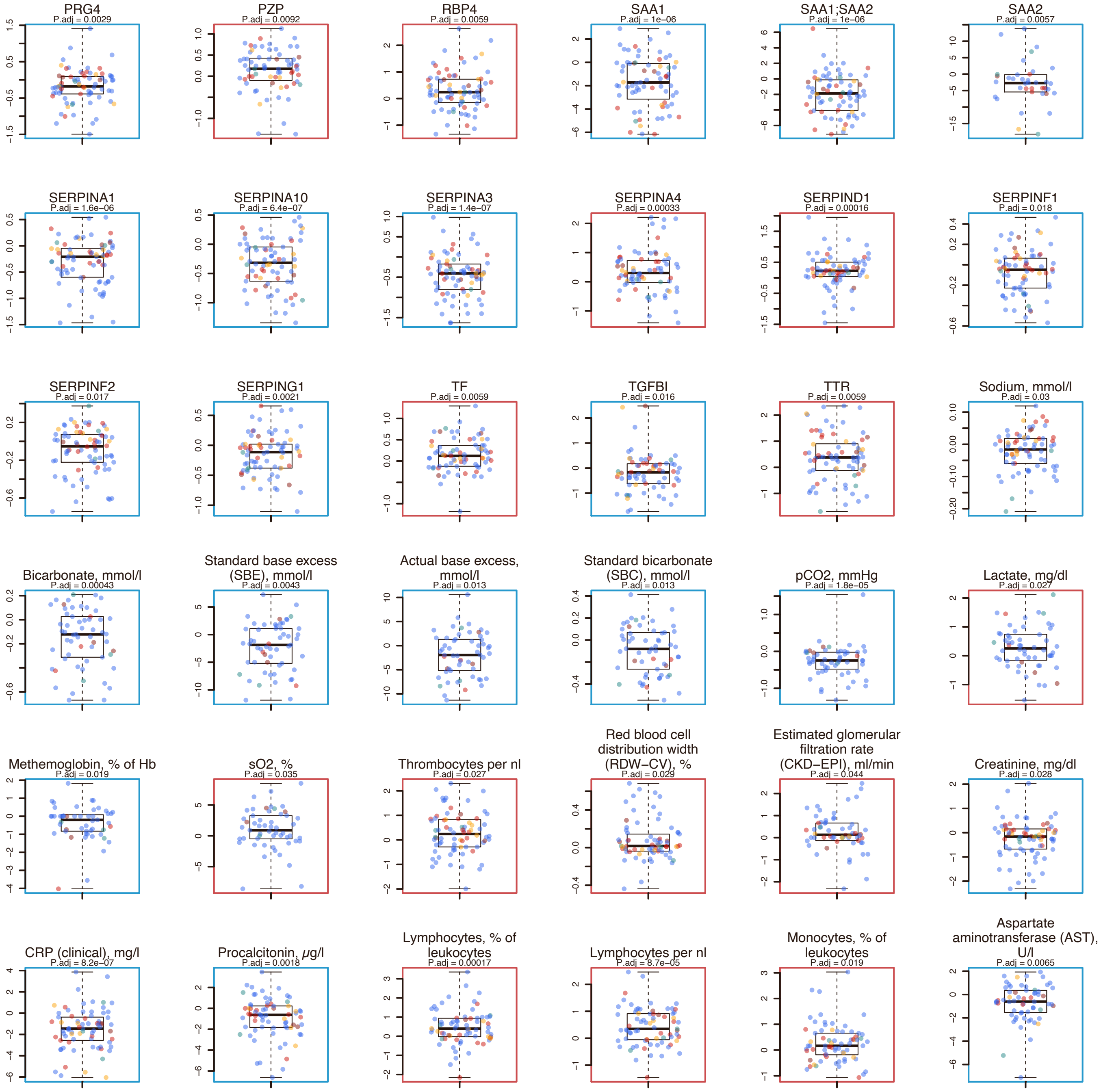
PRG4
P.adj = 0.0029
PZP
P.adj = 0.0092
RBP4
P.adj = 0.0059
SAA1
P.adj = 1e−06
SAA1;SAA2
P.adj = 1e−06
SAA2
P.adj = 0.0057
SERPINA1
P.adj = 1.6e−06
SERPINA10
P.adj = 6.4e−07
SERPINA3
P.adj = 1.4e−07
SERPINA4
P.adj = 0.00033
SERPIND1
P.adj = 0.00016
SERPINF1
P.adj = 0.018
SERPINF2
P.adj = 0.017
SERPING1
P.adj = 0.0021
TF
P.adj = 0.0059
TGFBI
P.adj = 0.016
TTR
P.adj = 0.0059
Sodium, mmol/l
P.adj = 0.03
Bicarbonate, mmol/l
P.adj = 0.00043
Standard base excess (SBE), mmol/l
P.adj = 0.0043
Actual base excess, mmol/l
P.adj = 0.013
Standard bicarbonate (SBC), mmol/l
P.adj = 0.013
pCO2, mmHg
P.adj = 1.8e−05
Lactate, mg/dl
P.adj = 0.027
Methemoglobin, % of Hb
P.adj = 0.019
sO2, %
P.adj = 0.035
Thrombocytes per nl
P.adj = 0.027
Red blood cell distribution width (RDW−CV), %
P.adj = 0.029
Estimated glomerular filtration rate (CKD−EPI), ml/min
P.adj = 0.044
Creatinine, mg/dl
P.adj = 0.028
CRP (clinical), mg/l
P.adj = 8.2e−07
Procalcitonin, μg/l
P.adj = 0.0018
Lymphocytes, % of leukocytes
P.adj = 0.00017
Lymphocytes per nl
P.adj = 8.7e−05
Monocytes, % of leukocytes
P.adj = 0.019
Aspartate aminotransferase (AST), U/l
P.adj = 0.0065

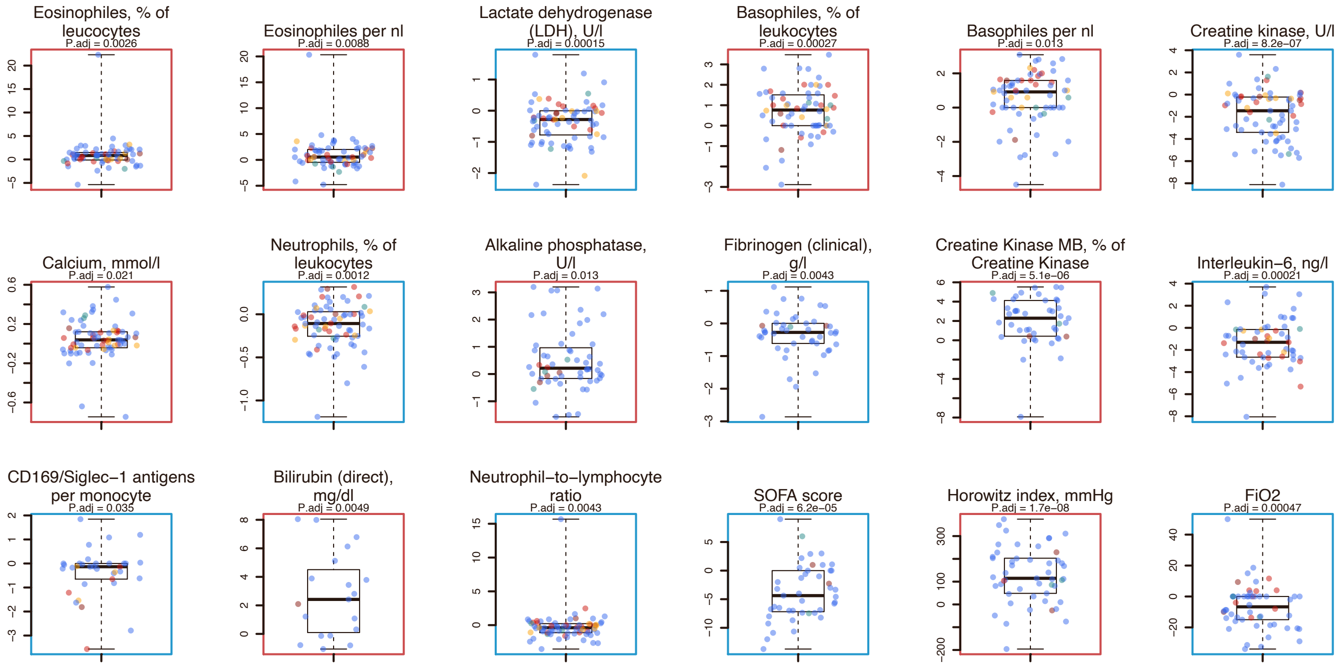
Eosinophiles, % of leucocytes
P.adj = 0.0026
Eosinophiles per nl
P.adj = 0.0088
Lactate dehydrogenase (LDH), U/l
P.adj = 0.00015
Basophiles, % of leukocytes
P.adj = 0.00027
Basophiles per nl
P.adj = 0.013
Creatine kinase, U/l
P.adj = 8.2e−07
Calcium, mmol/l
P.adj = 0.021
Neutrophils, % of leukocytes
P.adj = 0.0012
Alkaline phosphatase, U/l
P.adj = 0.013
Fibrinogen (clinical), g/l
P.adj = 0.0043
Creatine Kinase MB, % of Creatine Kinase
P.adj = 5.1e−06
Interleukin−6, ng/l
P.adj = 0.00021
CD169/Siglec−1 antigens per monocyte
P.adj = 0.035
Bilirubin (direct), mg/dl
P.adj = 0.0049
Neutrophil−to−lymphocyte ratio
P.adj = 0.0043
SOFA score
P.adj = 6.2e−05
Horowitz index, mmHg
P.adj = 1.7e−08
FiO2
P.adj = 0.00047

Figure S15. **Omics features differentially changing during the peak of the disease depending on the severity**

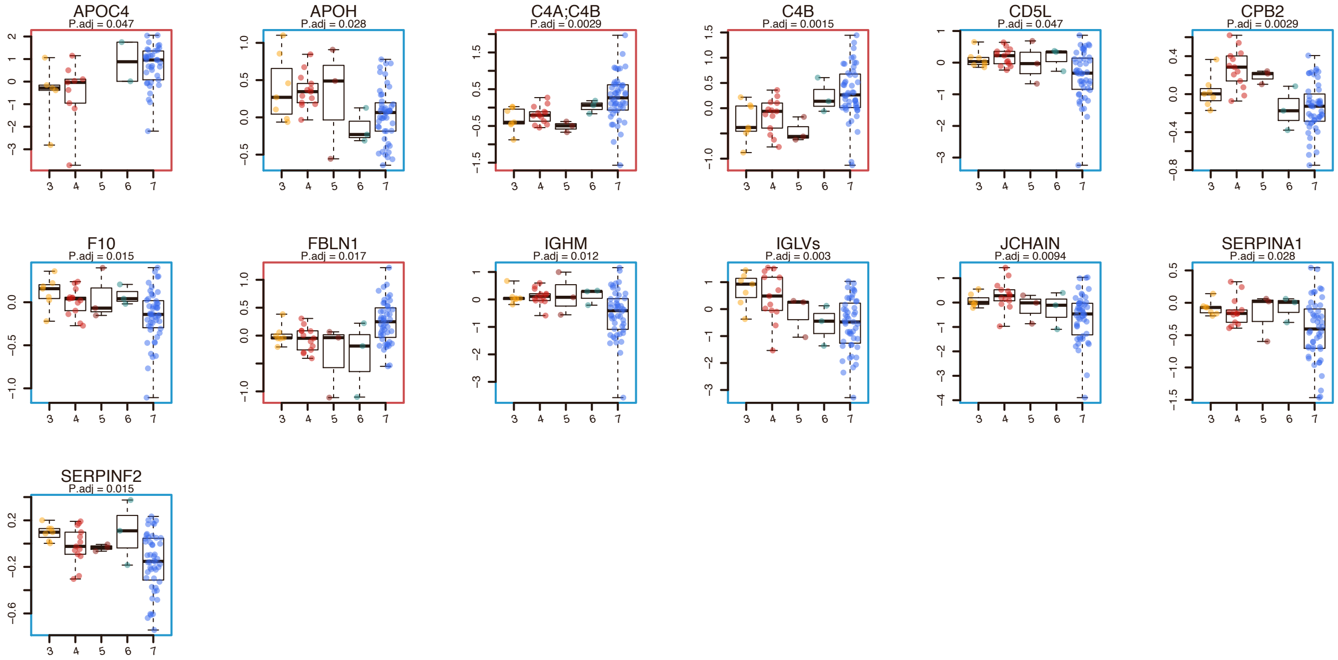

Figure S16 (following 9 pages). **Trajectories at the peak of the disease**

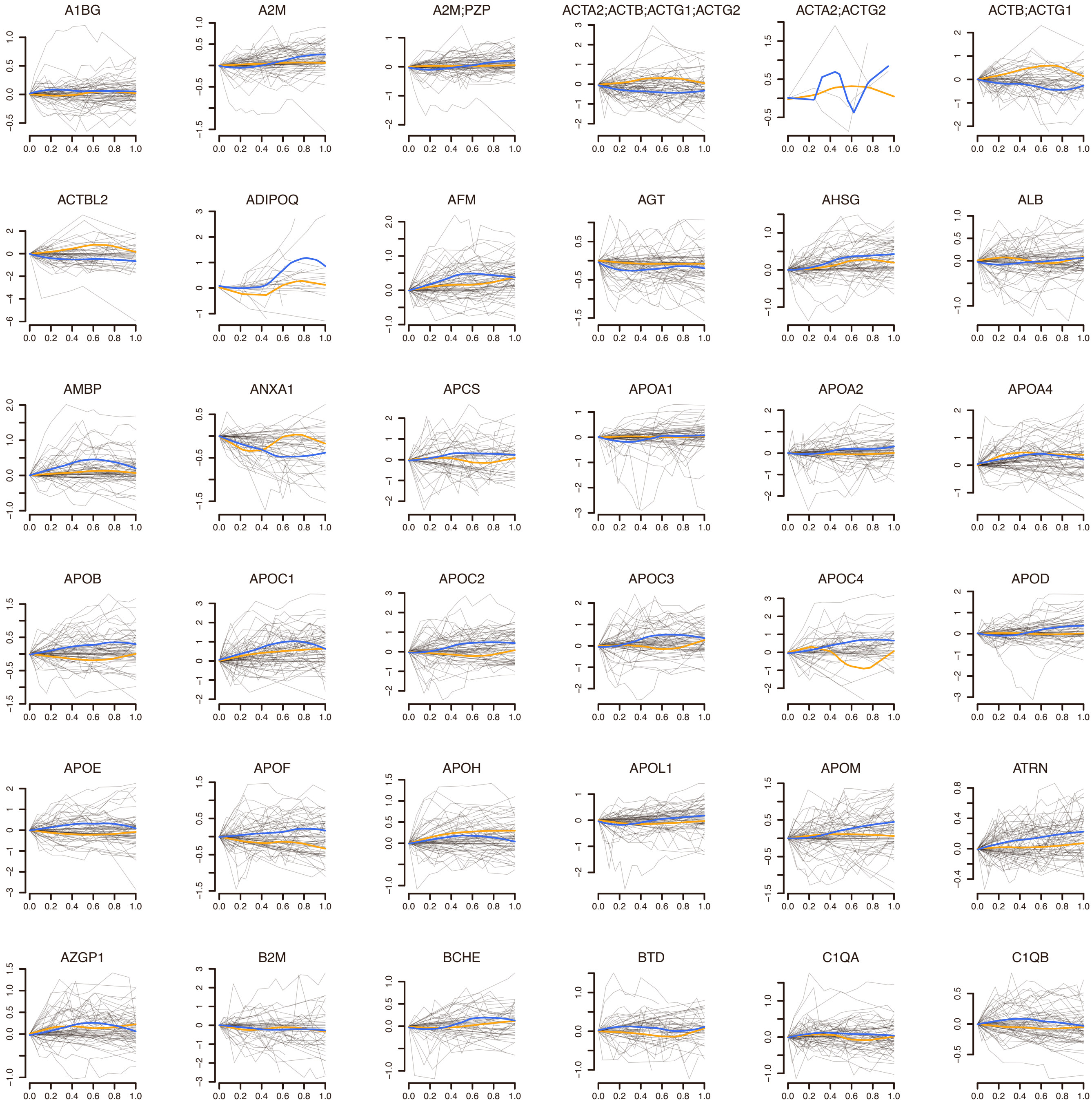

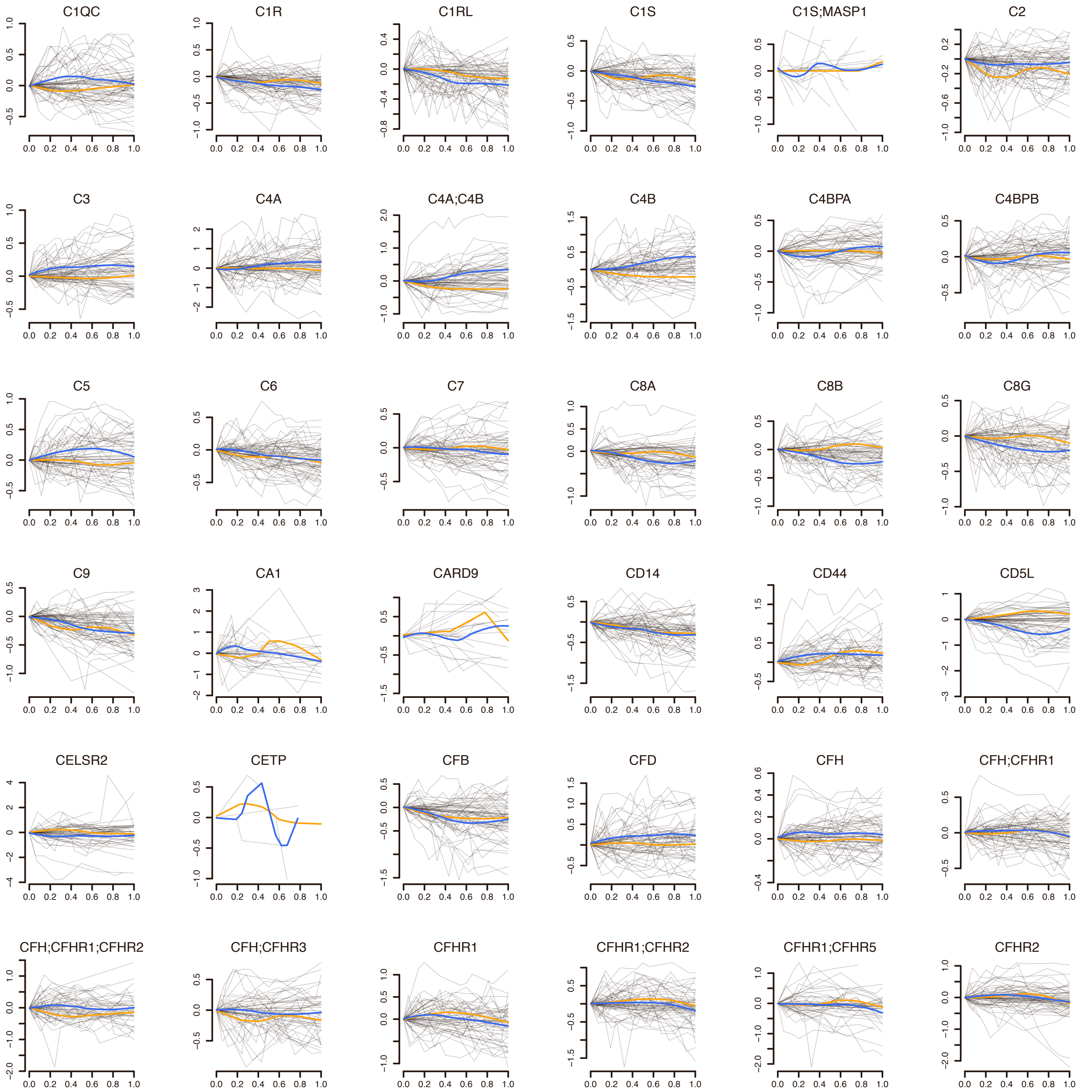
C1QC
C1R
C1RL
C1S
C1S;MASP1
C2
C3
C4A
C4A;C4B
C4B
C4BPA
C4BPB
C5
C6
C7
C8A
C8B
C8G
C9
CA1
CARD9
CD14
CD44
CD5L
CELSR2
CETP
CFB
CFD
CFH
CFH;CFHR1
CFH;CFHR1;CFHR2
CFH;CFHR3
CFHR1
CFHR1;CFHR2
CFHR1;CFHR5
CFHR2

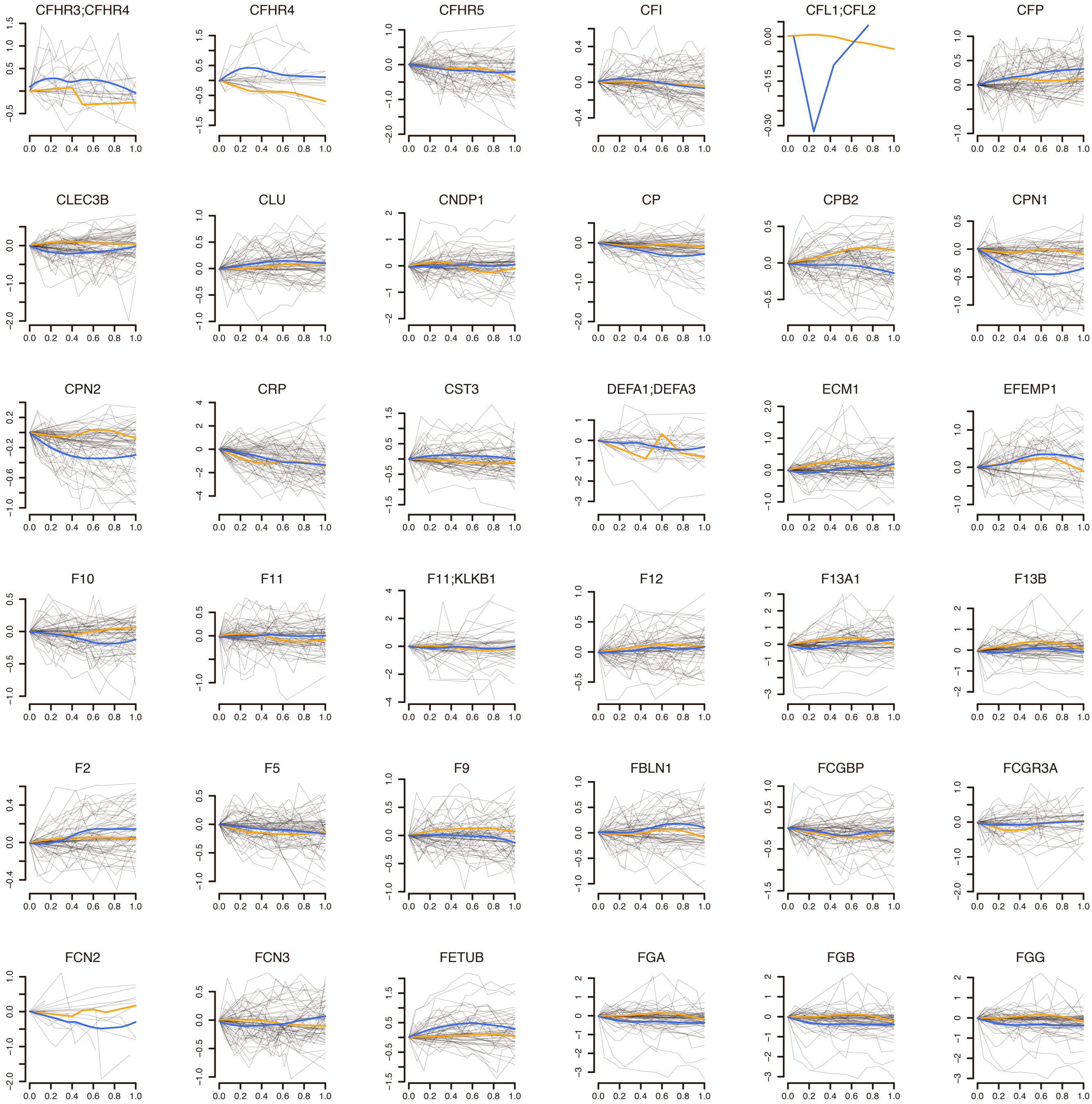
CFHR3;CFHR4
CFHR4
CFHR5
CFI
CFL1;CFL2
CFP
CLEC3B
CLU
CNDP1
CP
CPB2
CPN1
CPN2
CRP
CST3
DEFA1;DEFA3
ECM1
EFEMP1
F10
F11
F11;KLKB1
F12
F13A1
F13B
F2
F5
F9
FBLN1
FCGBP
FCGR3A
FCN2
FCN3
FETUB
FGA
FGB
FGG

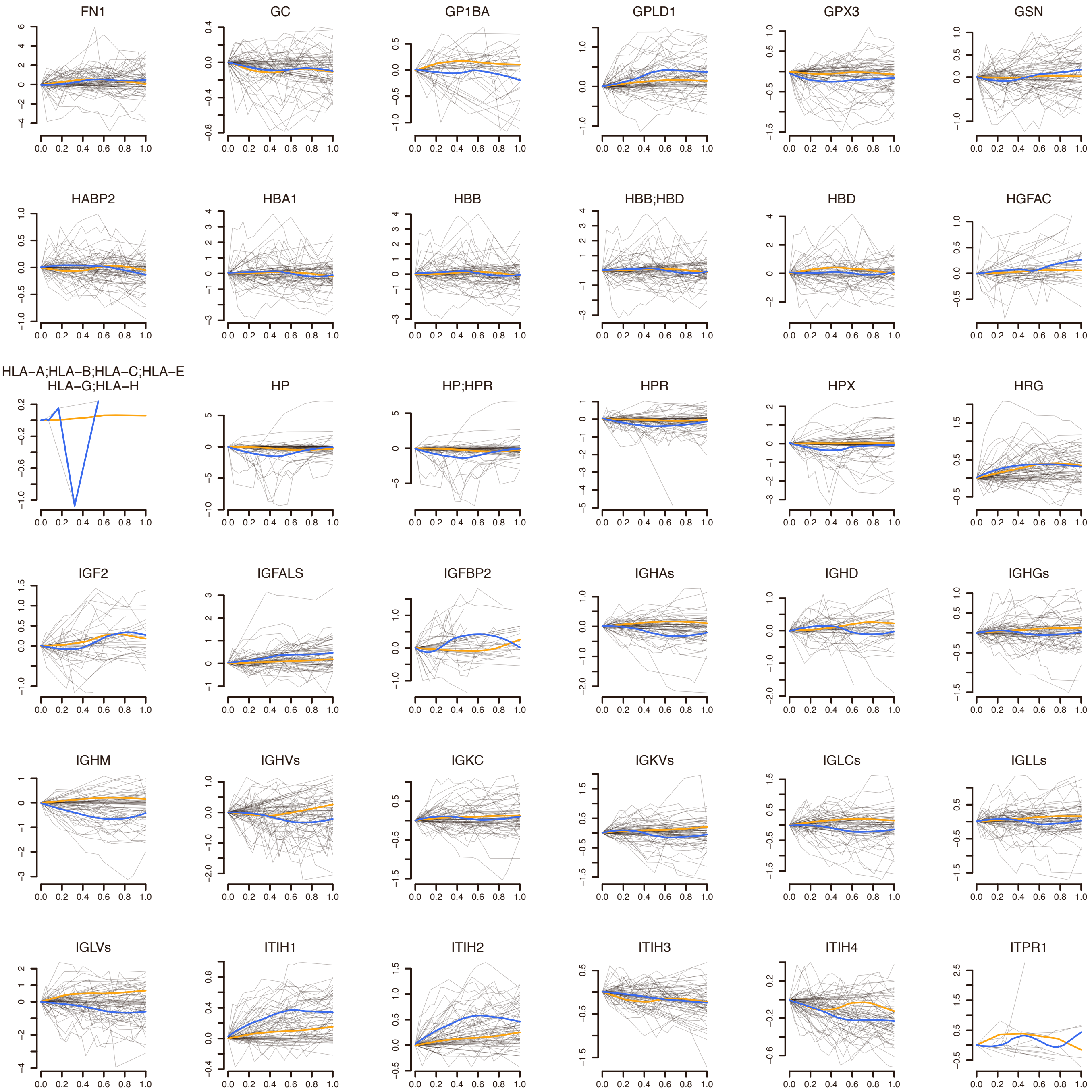
FN1
GC
GP1BA
GPLD1
GPX3
GSN
HABP2
HBA1
HBB
HBB;HBD
HBD
HGFAC
HLA−A;HLA−B;HLA−C;HLA−E
HLA−G;HLA−H
HP
HP;HPR
HPR
HPX
HRG
IGF2
IGFALS
IGFBP2
IGHAs
IGHD
IGHGs
IGHM
IGHVs
IGKC
IGKVs
IGLCs
IGLLs
IGLVs
ITIH1
ITIH2
ITIH3
ITIH4
ITPR1

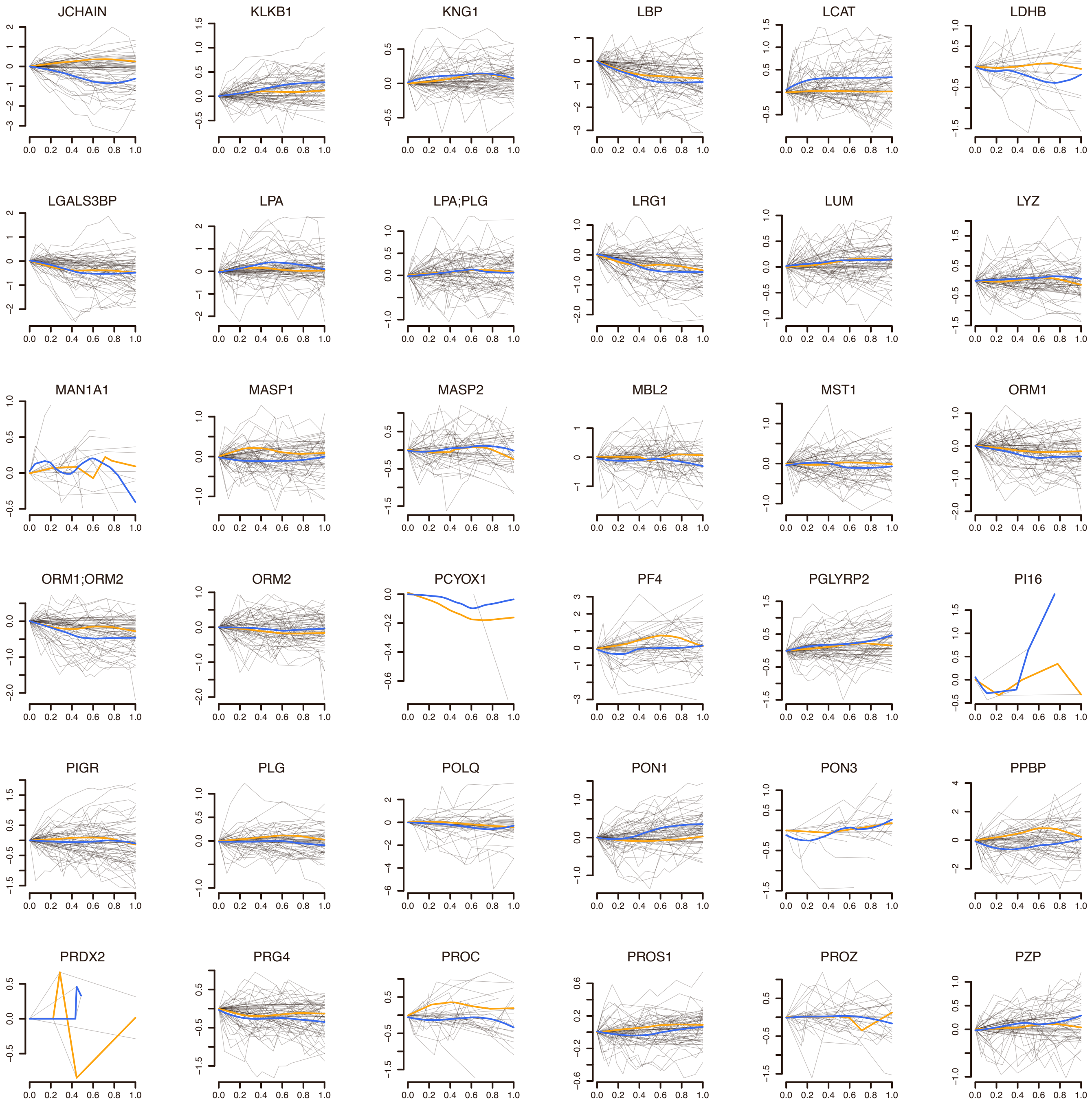
JCHAIN
KLKB1
KNG1
LBP
LCAT
LDHB
LGALS3BP
LPA
LPA;PLG
LRG1
LUM
LYZ
MAN1A1
MASP1
MASP2
MBL2
MST1
ORM1
ORM1;ORM2
ORM2
PCYOX1
PF4
PGLYRP2
PI16
PIGR
PLG
POLQ
PON1
PON3
PPBP
PRDX2
PRG4
PROC
PROS1
PROZ
PZP

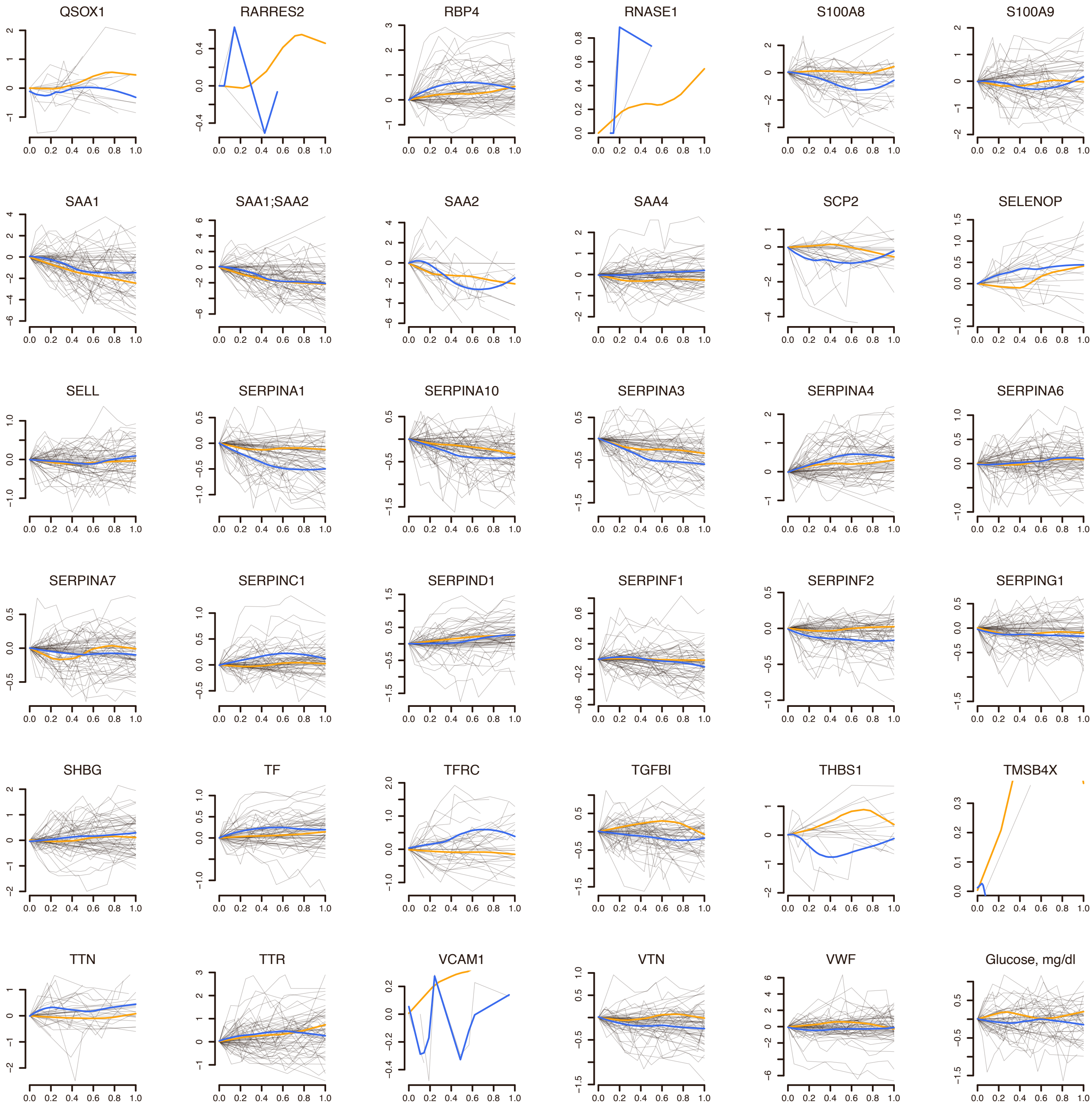
QSOX1
RARRES2
RBP4
RNASE1
S100A8
S100A9
SAA1
SAA1;SAA2
SAA2
SAA4
SCP2
SELENOP
SELL
SERPINA1
SERPINA10
SERPINA3
SERPINA4
SERPINA6
SERPINA7
SERPINC1
SERPIND1
SERPINF1
SERPINF2
SERPING1
SHBG
TF
TFRC
TGFBI
THBS1
TMSB4X
TTN
TTR
VCAM1
VTN
VWF
Glucose, mg/dl

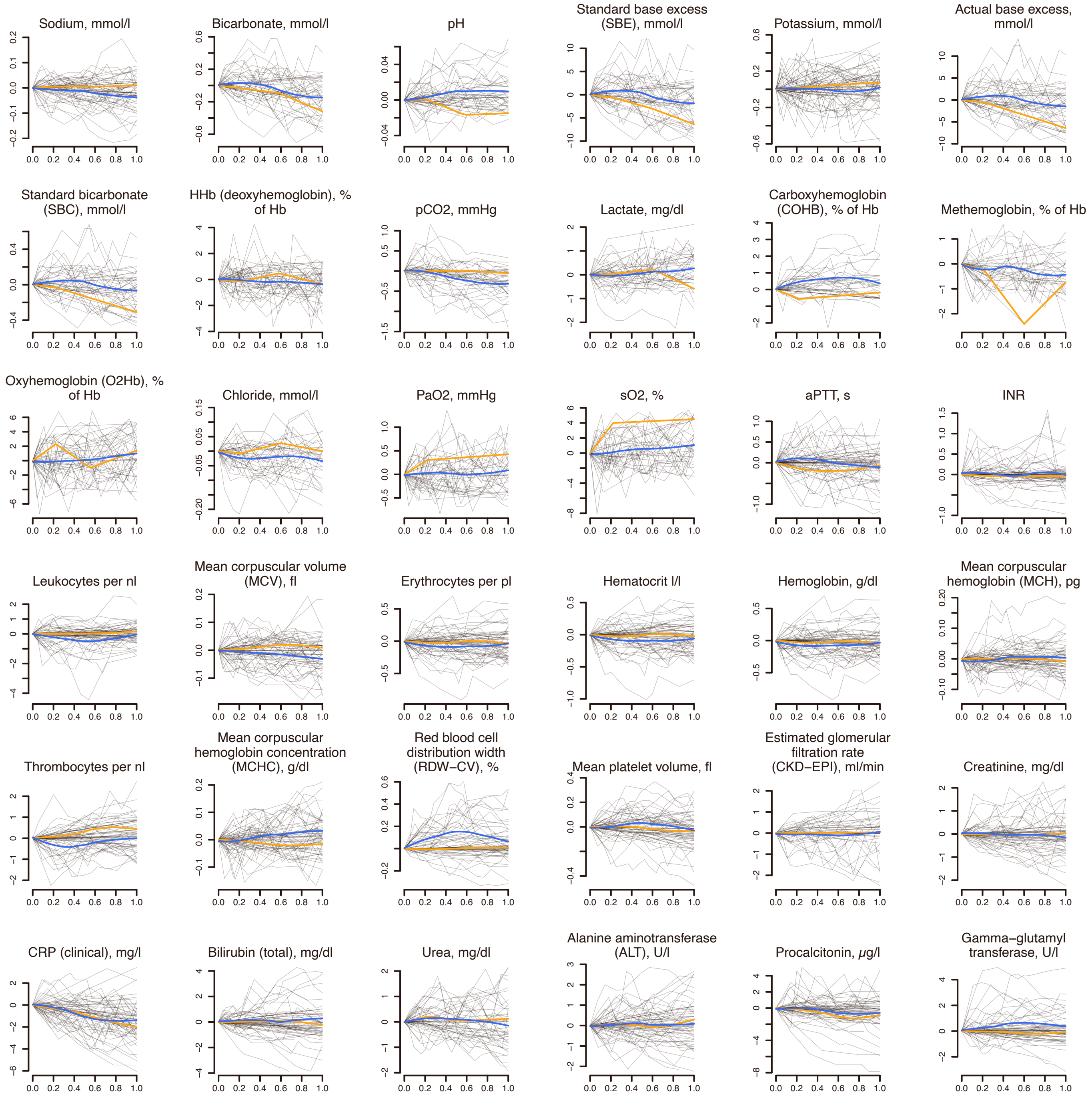
Sodium, mmol/l
Bicarbonate, mmol/l
pH
Standard base excess (SBE), mmol/l
Potassium, mmol/l
Actual base excess, mmol/l
Standard bicarbonate (SBC), mmol/l
HHb (deoxyhemoglobin), % of Hb
pCO2, mmHg
Lactate, mg/dl
Carboxyhemoglobin (COHB), % of Hb
Methemoglobin, % of Hb
Oxyhemoglobin (O2Hb), % of Hb
Chloride, mmol/l
PaO2, mmHg
sO2, %
aPTT, s
INR
Leukocytes per nl
Mean corpuscular volume (MCV), fl
Erythrocytes per pl
Hematocrit l/l
Hemoglobin, g/dl
Mean corpuscular hemoglobin (MCH), pg
Thrombocytes per nl
Mean corpuscular hemoglobin concentration (MCHC), g/dl
Red blood cell distribution width (RDW−CV), %
Mean platelet volume, fl
Estimated glomerular filtration rate (CKD−EPI), ml/min
Creatinine, mg/dl
CRP (clinical), mg/l
Bilirubin (total), mg/dl
Urea, mg/dl
Alanine aminotransferase (ALT), U/l
Procalcitonin, μg/l
Gamma−glutamyl transferase, U/l

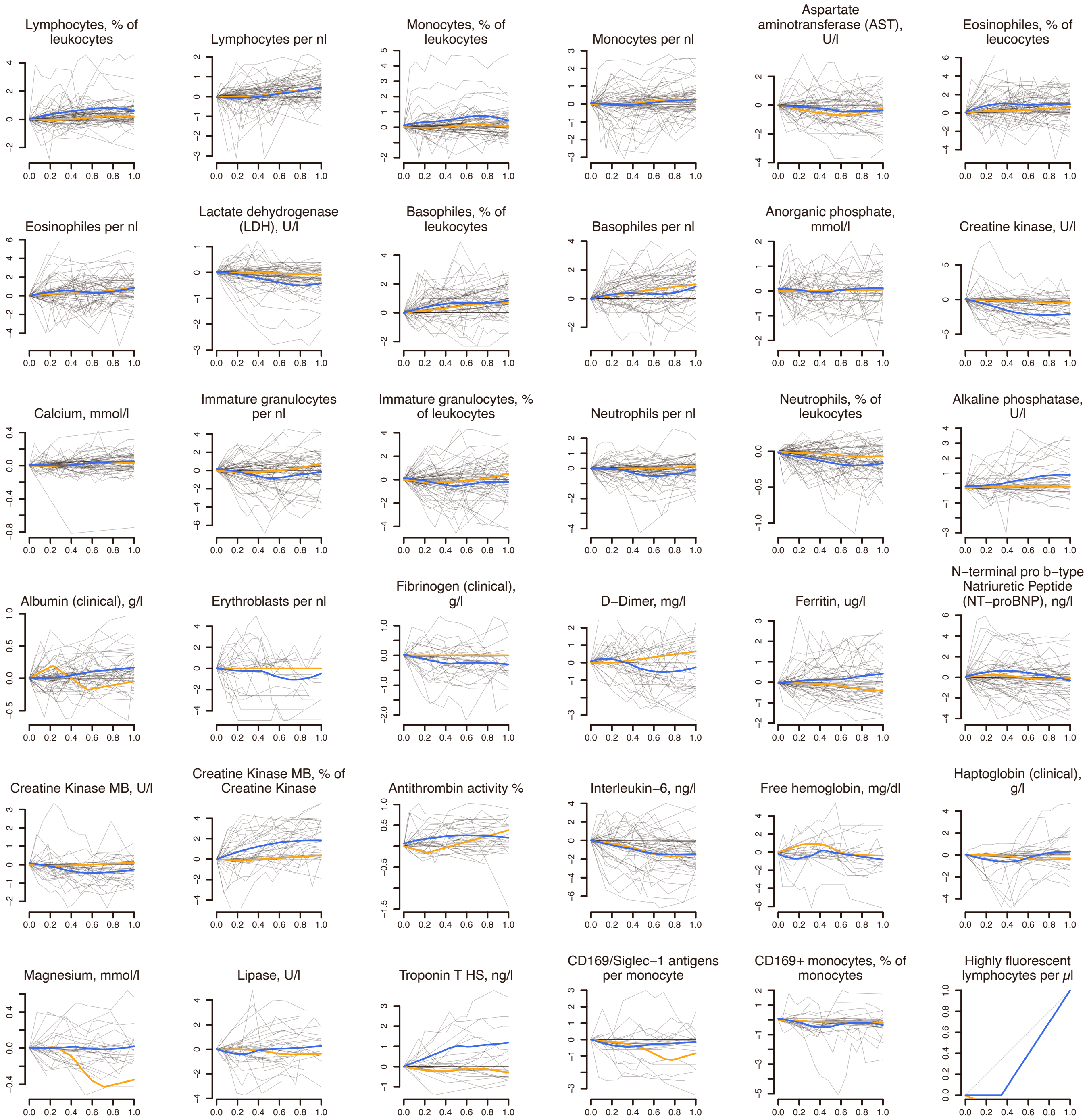
Lymphocytes, % of leukocytes
Lymphocytes per nl
Monocytes, % of leukocytes
Monocytes per nl
Aspartate aminotransferase (AST), U/l
Eosinophiles, % of leucocytes
Eosinophiles per nl
Lactate dehydrogenase (LDH), U/l
Basophiles, % of leukocytes
Basophiles per nl
Anorganic phosphate, mmol/l
Creatine kinase, U/l
Calcium, mmol/l
Immature granulocytes per nl
Immature granulocytes, % of leukocytes
Neutrophils per nl
Neutrophils, % of leukocytes
Alkaline phosphatase, U/l
Albumin (clinical), g/l
Erythroblasts per nl
Fibrinogen (clinical), g/l
D−Dimer, mg/l
Ferritin, ug/l
N−terminal pro b−type Natriuretic Peptide (NT−proBNP), ng/l
Creatine Kinase MB, U/l
Creatine Kinase MB, % of Creatine Kinase
Antithrombin activity %
Interleukin−6, ng/l
Free hemoglobin, mg/dl
Haptoglobin (clinical), g/l
Magnesium, mmol/l
Lipase, U/l
Troponin T HS, ng/l
CD169/Siglec−1 antigens per monocyte
CD169+ monocytes, % of monocytes
Highly fluorescent lymphocytes per μl

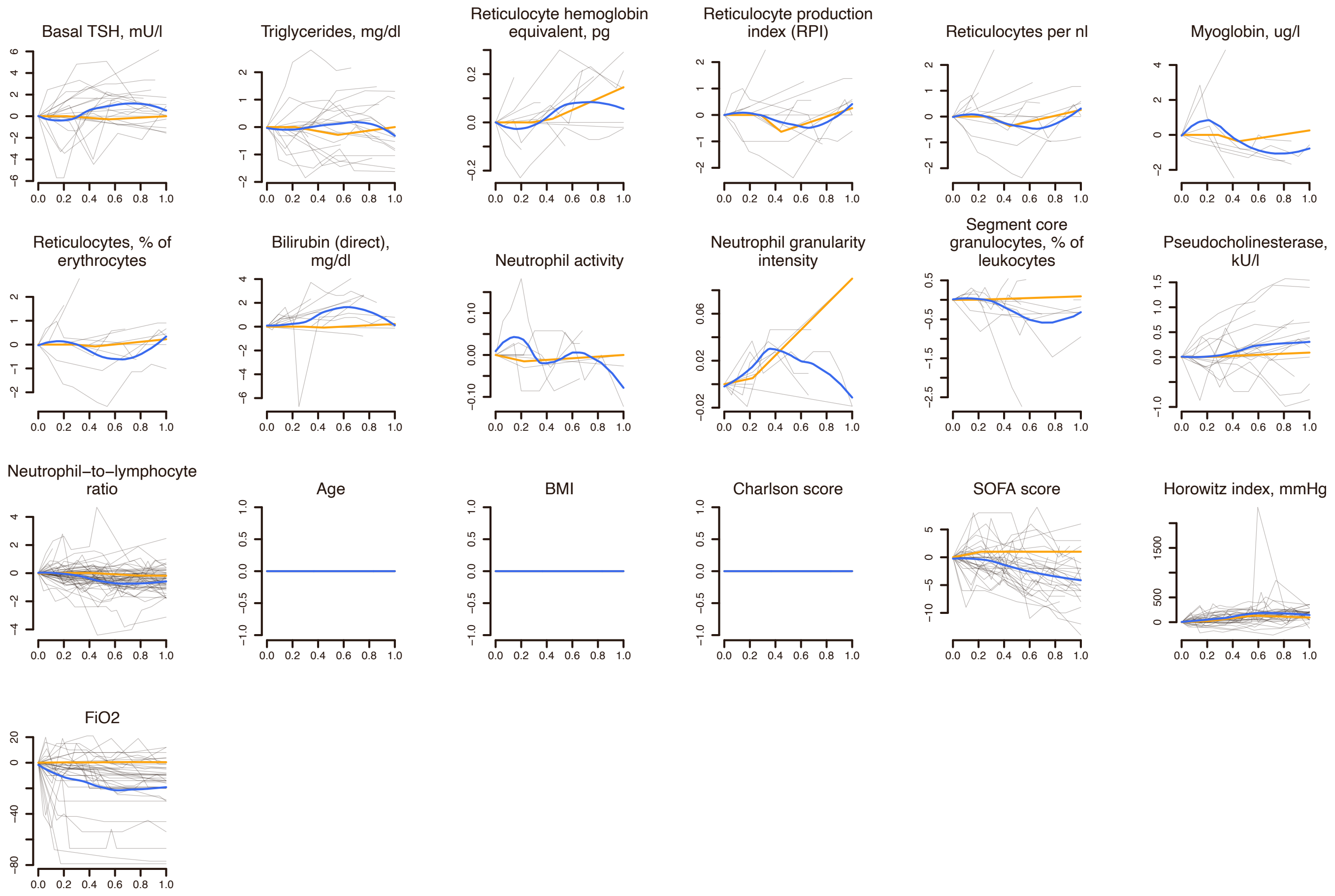

Basal TSH, mU/l
Triglycerides, mg/dl
Reticulocyte hemoglobin equivalent, pg
Reticulocyte production index (RPI)
Reticulocytes per nl
Myoglobin, ug/l
Reticulocytes, % of erythrocytes
Bilirubin (direct), mg/dl
Neutrophil activity
Neutrophil granularity intensity
Segment core granulocytes, % of leukocytes
Pseudocholinesterase, kU/l
Neutrophil−to−lymphocyte ratio
Age
BMI
Charlson score
SOFA score
Horowitz index, mmHg
FiO2

Figure S17. **Omics features predictive of future disease worsening**

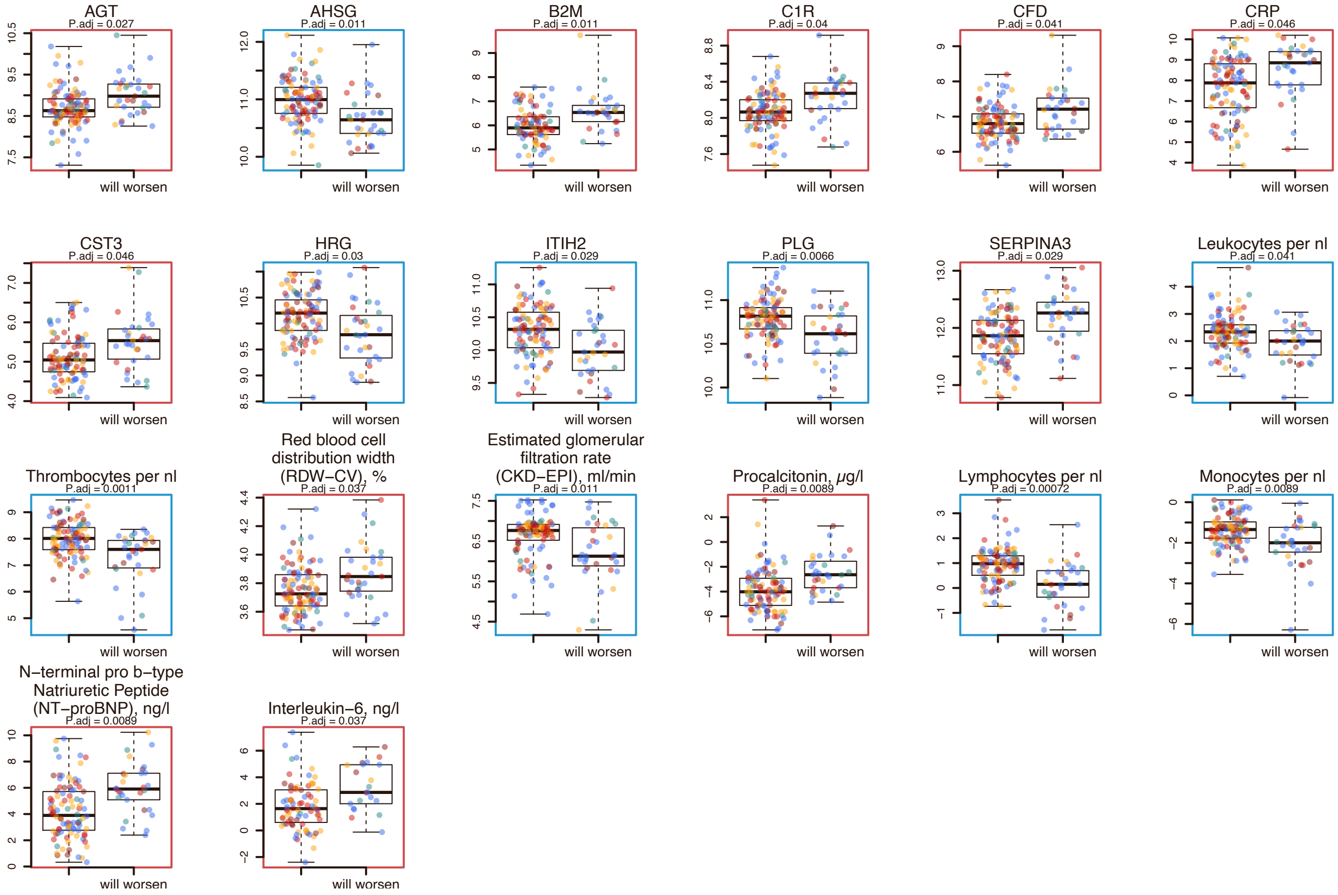

Figure S18 (following 2 pages). **Omics features predictive of the remaining time in hospital for patients at WHO = 3**

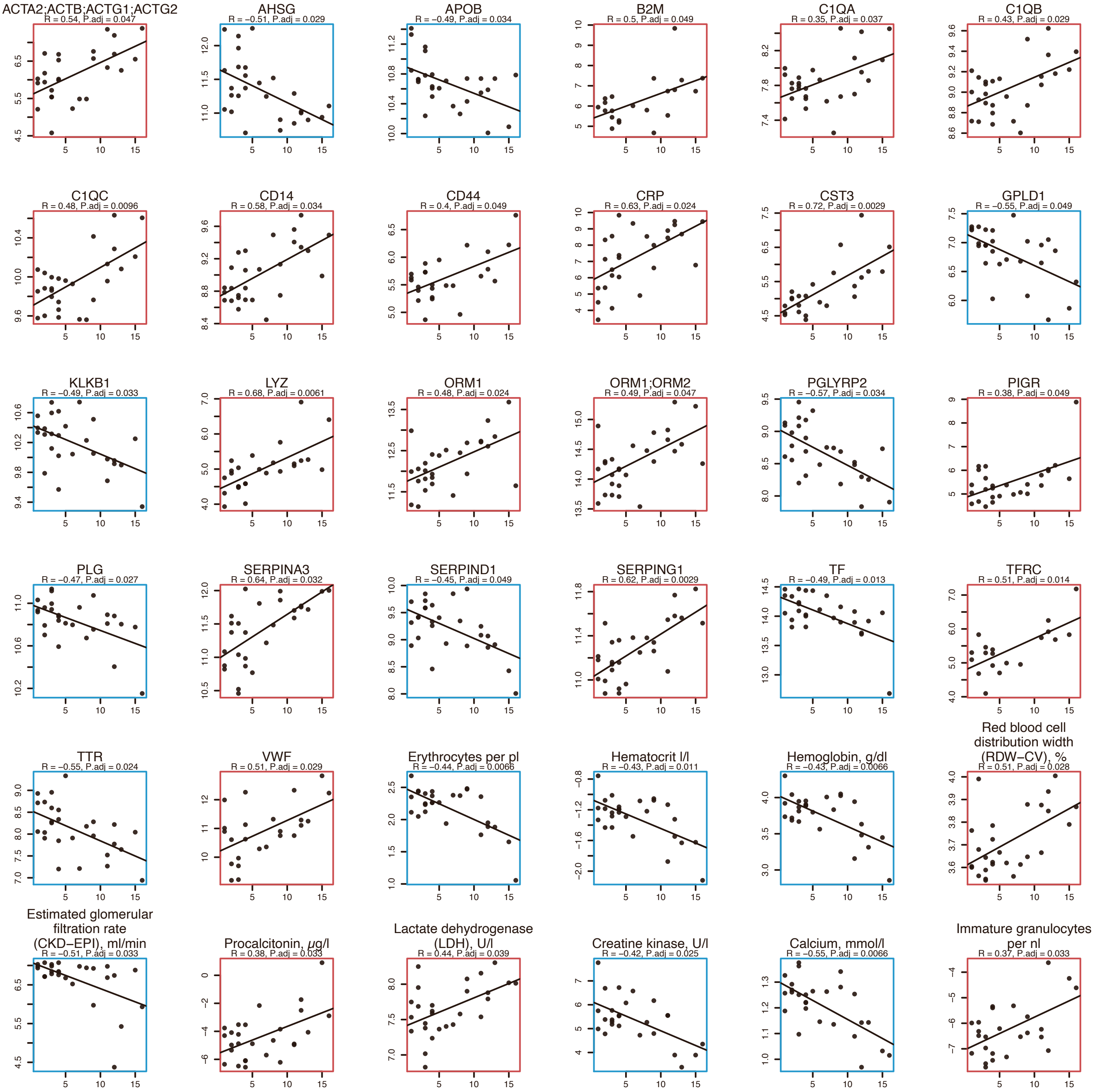

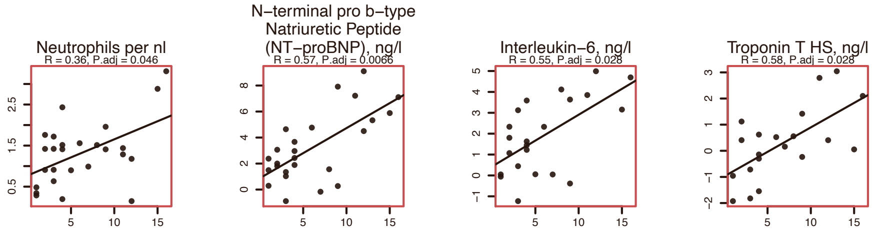
Neutrophils per nl
R = 0.36, P.adj = 0.046
N−terminal pro b−type
Natriuretic Peptide
(NT−proBNP), ng/l
R = 0.57, P.adj = 0.0066
Interleukin−6, ng/l
R = 0.55, P.adj = 0.028
Troponin T HS, ng/l
R = 0.58, P.adj = 0.028
